# SARs for the Antiparasitic Plant Metabolite Pulchrol. Part 2: B- and C-Ring Substituents

**DOI:** 10.3390/molecules25194510

**Published:** 2020-10-01

**Authors:** Paola Terrazas, Sophie Manner, Olov Sterner, Marcelo Dávila, Alberto Giménez, Efrain Salamanca

**Affiliations:** 1 Department of Chemistry, Centre for Analysis and Synthesis, Lund University, 22100 Lund, Sweden; paola.terrazas_villarroel@chem.lu.se (P.T.); sophie.manner@chem.lu.se (S.M.); 2 Centre of Agroindustrial Technology, San Simón University, Cochabamba 3299, Bolivia; marcelodavila@fcyt.umss.edu.bo; 3 Institute for Pharmacological and Biochemical Sciences, San Andrés University, La Paz 3299, Bolivia; agimenez@megalink.com (A.G.); efrain_salamanca@hotmail.com (E.S.)

**Keywords:** *Trypanosoma cruzi*, *Leishmania amazonensis*, *Leishmania braziliensis*, pulchrol, benzo[*c*]chromenes, Structure-Activity Relationships (SARs)

## Abstract

Neglected tropical diseases affect most of the underprivileged populations in tropical countries. Among these are chagas and leishmaniasis, present mainly in South and Central America, Africa and East Asia. Current treatments are long and have severe adverse effects, therefore there is a strong need to develop alternatives. In this study, we base our research on the plant metabolite pulchrol, a natural benzochromene which has been shown to possess antiparasitic activity against *Trypanosoma* and *Leishmania* species. In a recent study, we investigated how changes in the benzyl alcohol functionality affected the antiparasitic activity, but the importance of B- and C-ring substituents is not understood. Fifteen derivatives of pulchrol with different substituents in positions 1, 2, 3, and 6 while leaving the A-ring intact, were therefore prepared by total synthesis, assayed, and compared with pulchrol and positive controls. The generated series and parental molecule were tested in vitro for antiparasitic activity against *Trypanosoma cruzi*, *Leishmania braziliensis*, and *L. amazonensis*, and cytotoxicity using RAW cells. Substantial differences in the activity of the compounds synthesized were observed, of which some were more potent towards *Trypanosoma cruzi* than the positive control benznidazole. A general tendency is that alkyl substituents improve the potency, especially when positioned on C-2.

## 1. Introduction

Neglected Tropical Diseases (NTDs) native to tropical regions, affect around 1 billion people in 149 countries [1], most of them part of underprivileged populations [2,3,4]. Some NTDs are associated to parasites from the family Trypanosomatidae, among them *Trypanosoma cruzi* which causes the chagas disease, while several *Leishmania* species are responsible for leishmaniasis. Chagas disease (transmitted by a Triatominae bug) affects around 8 million people, mainly in South and Central America [5]. It may remain asymptomatic for decades until the heart tissue is eventually damaged sufficiently to cause death [6,7]. The antiparasitic drugs benznidazole and nifurtimox are used to treat chagas, and are efficient when given immediately after infection. However, they lose efficiency with time, and present serious adverse effects [8,9]. Leishmaniasis (transmitted by Phlebotomine sand flies) is found mainly in Africa, Latin America and East Asia. It exists in three forms: cutaneous, mucocutaneous, and visceral, and some 700,000 to 1 million new cases occur annually [10]. The treatment, mainly pentavalent antimonials, amphotericin B or miltefosine, can be associated to serious adverse effects, and in some cases require hospitalization [11].

An important source for the development of new drug candidates is Nature itself, and natural products may provide new bioactive and selective chemical leads [12]. However, the isolation of novel drug candidates from natural sources is often hampered by the minute amounts available, which prevent them from being assayed in pharmacological tests [12,13,14]. The development of synthetic routes to obtain these natural materials solve the problem, and also allows derivatives and analogues to be prepared in order to understand SARs [12,15].

**Figure 1:**
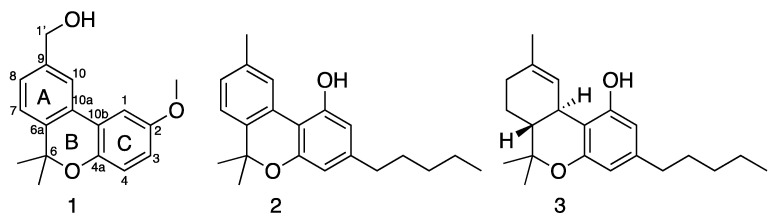
Structures of pulchrol (**1**), cannabinol (**2**) and tetrahydrocannabinol (THC) (**3**).

A scaffold for biologically active natural products is benzo[*c*]chromene, and benzo[*c*]chromenes have been shown to possess a wide range of biological activities, and this study is based on pulchrol (**1**). They have been isolated from lichens belonging to the *Graphis* genus, the fungus *Acremonium* (which also produces cephalosporin antibiotics), and many plants [16]. Benzo[*c*]chromenes may for example inhibit cholinesterase enzymes [17], and have an affinity for the cannabinoid receptors CB1 and CB2 [18]. Cannabinol (**2,** see Figure 1), isolated from the plant *Cannabis sativa*, has 10 times stronger affinity for the CB2 receptor compared to the main constituent of *Cannabis*, THC (**3**), which is responsible for its psychotropic activity [19]. Cannabinol (**2**) was also found to possess immunomodulatory [20], and antineoplastic activity in Lewis lung tumor cells [21].

The benzo[*c*]chromene pulchrol (**1**), found in the roots of *Bourreria pulchra*, [22] is known as “Bakalche” in Yucatan (Mexico) and used to treat cutaneous diseases, injuries, viral infections and fevers [23,24]. Pulchrol (**1**) was found to possess antiparasitic activity against *Leishmania braziliensis*, *L. amazonensis*, *L. mexicana* and *Trypanosoma cruzi* [22,25]. A synthetic route was developed in 2014 [26,27], and recently we reported the effects that transformations of its A-ring benzyl alcohol functionality have against *T. cruzi* epimastigotes, *L. amazonensis* promastigotes and *L. braziliensis* promastigotes, as well as their cytotoxicity towards mammalian cells (assayed in RAW cells) [28]. We are now interested to expand the previous study [28] with compounds having various substituents in the B- and C-rings. Our aim is to use the synthetic routes and some of the intermediates used for the synthesis of pulchrol (**1**), to obtain new derivatives. The only position available for exchange in the B-ring is C-6, and we were especially interested in examining the role of the alkyl substituents. Ring C has theoretically four positions open for substitution, but in practice only three. Here we focused on the presence and position of a methoxy group, as well as various alkyl substituents. As a result, we have prepared 15 new analogues (**8a**–**10g**, **10a**–**10h**) and we have tested them for antiparasitic activity towards *T. cruzi*, *L. amazonensis* and *L. braziliensis* together with **1** and the positive controls benznidazole and miltefosine. The cytotoxicity towards mammalian cells of all the compounds was determined with murine macrophage cells (RAW). This and additional studies of the antiparasitic activities of pulchrol analogues will eventually provide us with a model that can be used for the design of more potent antiparasitic structures with lower cytotoxicity.

## 2. Results and Discussion

### 2.1. Modifications in the B-Ring

**Scheme 1:**
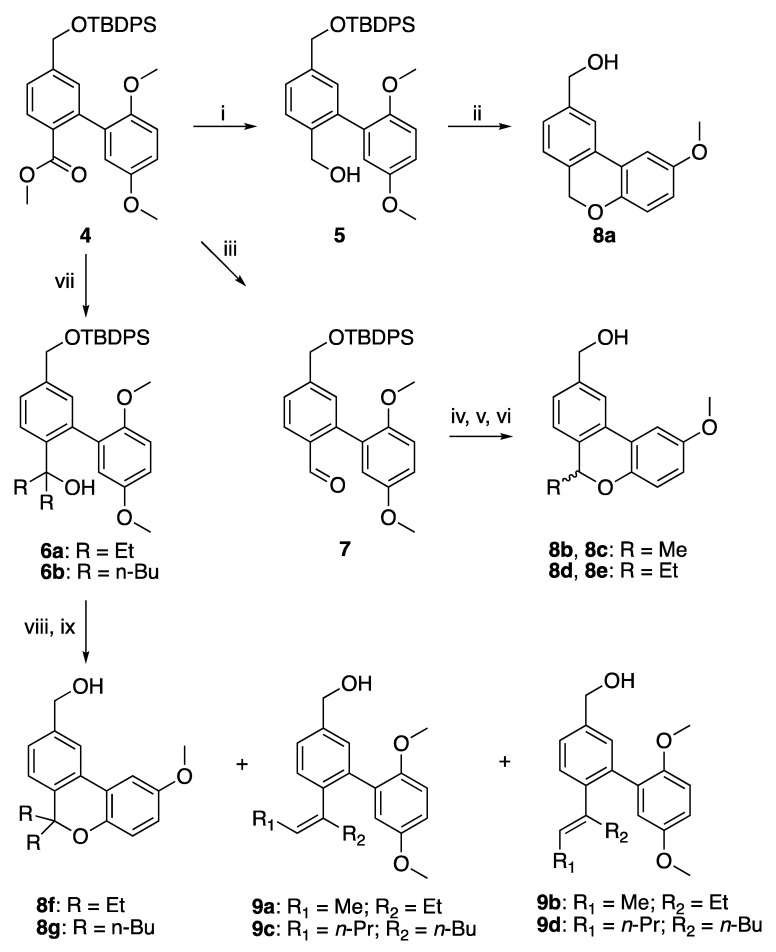
*Reagents and conditions*: (i) **4** (1 equiv), DIBALH (2.4 equiv), dry toluene, −78 °C; (ii) **5** (1 equiv), NaSEt (4 equiv), dry DMF, 110 °C; (iii) **4** (1 equiv), morpholine (2 equiv), DIBALH (1 equiv), dry THF, 0 °C; (iv) **7** (1 equiv), corresponding organolithium reagent (2 equiv), dry THF, 0 °C or −78 °C depending on the organolithium reagent; (v) PBr_3_ (0.34 equiv), LiI (3 equiv), dry CH_2_Cl_2_, rt; (vi) TBAF (2 equiv), THF, rt; (vii) **4** (1 equiv), organolithium reagent (4 equiv), dry THF, 0 °C or −78 °C depending on the organolithium reagent; (viii) **6** (1 equiv), HI (10 equiv), MeCN, rt; (ix) TBAF (1.1 equiv), THF, rt.

The synthetic routes used to prepare derivatives with variations in ring B were partly based on an already published synthetic route to yield pulchrol [26]. The common intermediate **4** was used as the starting material for the synthesis of all ring B derivatives (see Scheme 1).

Derivative **8a** was prepared by reducing the ester group in **4** to the alcohol **5**, which was treated with NaSEt in dry DMF at 110 °C to obtain an *ortho* demethylated phenol, which was not isolated as an intermediate as it spontaneously cyclized and was deprotected to the desired product **8a**, the 6-demethylated analogue of **1**, albeit in low yields (7%). The monosubstituted analogues **8b**–**8e** were prepared by reducing the ester functionality of **4** to the aldehyde **7**, which by alkyl addition was transformed to the corresponding secondary alcohol. Cyclization using PBr_3_ in the presence of LiI gave the desired compounds [29]. The products **8b**–**8e** were obtained as racemic pairs, and the enantiomers were separated by HPLC with a normal phase semipreparative chiral column. The pure enantiomers were obtained in low yields (less than 10%). The determination of the absolute configuration of **8b**–**8e**, which could have been done with the secondary alcohols by the Mosher’s method, was not attempted as the enantiomers were approximately equipotent (*vide infra*). The absolute configuration of C-6 does not appear to influence the potency.

The 6.6-diethyl and 6.6-dibutyl analogues **8f** and **8g** were prepared from **6**a and **6b**, based on the pulchrol synthetic route [26]. The new alkyl groups were introduced by a double addition step to the ester group in **4** using the corresponding organolithium reagents. For the cyclization of **6a** and **6b** to **8f** and **8g**, an excess in hydroiodic acid was used, to avoid the formation of cannabidiol type byproducts as a result of elimination [28]. Nevertheless, the byproducts **9a** and **9b** appeared together with **8f**, and **9c** and **9d** with **8g**. The desired products were obtained by HPLC purification, with moderate yields (**8f** 30% and **8g** 56%).

### 2.2. Modifications in the C-Ring

**Figure 2:**
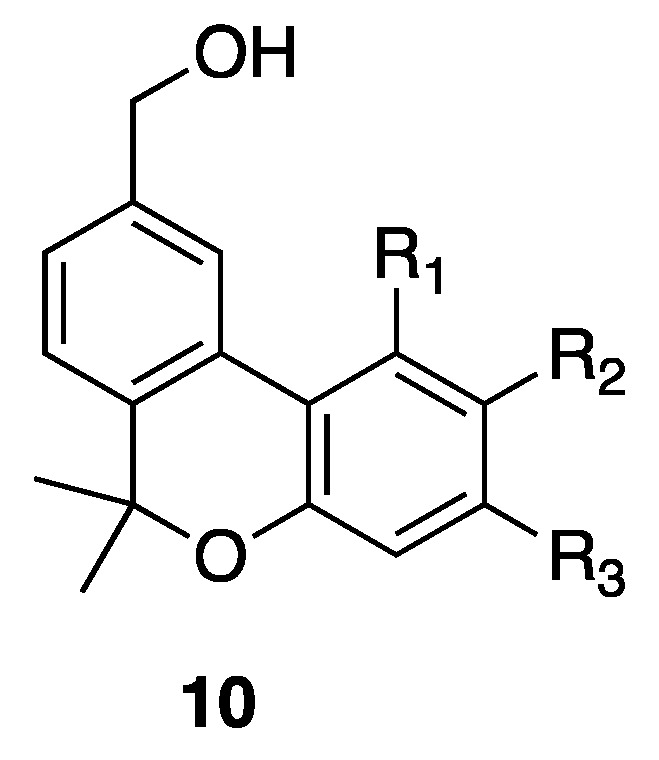
Modifications on ring C: **10a** R_1_ = H, R_2_ = H, R_3_ = OMe; **10b** R_1_ = OMe, R_2_ = H, R_3_ = H; **10c** R_1_ = H, R_2_ = Me, R_3_ = H; **10d** R_1_ = H, R_2_ = H, R_3_ = Me; **10e** R_1_ = H, R_2_ = H, R_3_ = H; **10f** R_1_ = H, R_2_ = *i*-Pr, R_3_ = H; **10g** R_1_ = H, R_2_ = H, R_3_ = *i*-Pr; **10h** R_1_ = H, R_2_ = *n*-Pen, R_3_ = H. See the Section 3 for synthetic details.

Analogues modified in the C-ring (see Figure 2 for the structures of compounds **10a** to **10h**) were prepared based on the procedure used to synthesize pulchrol (**1**) [26], but with different methoxylated phenyls forming the biaryl (corresponding to **4**) through a Suzuki coupling reaction. The major difference in the biaryl formation was that the reaction time in the microwave reactor had to be increased from 30 min to 60 min. The yields were generally good, varying from 75% to 92%. During the cyclization most of the alkyl substituted analogues were obtained in better yields (72% to 85%) than the derivatives **10a** and **10b** substituted with methoxy groups.

The biological activities of all the synthesized derivatives are given in Table 1, while the ^1^H- and ^13^C-NMR chemical shifts of the assayed compounds are given in Table 2 and Table 3.

### 2.3. Antiparasitic Activity of Pulchrol (***1***)

The natural product pulchrol has previously been investigated and shown to be toxic towards *Trypanosoma* and *Leishmania* parasites in vitro. The highest activity was reported towards *T. cruzi* epimastigotes (IC_50_ 18.5 μM) which is comparable to the potency shown by the drug benznidazole (19.2 μM) that currently is used to treat the chagas disease. Pulchrol also showed moderate leishmanicidal activity against *L. braziliensis* and *L. amazonesis* promastigotes, with IC_50_ values of 59.2 μM and 77.7 μM, respectively. The effects that modifications of the benzylic alcohol functionality of **1** have on the antiparasitic activity were studied previously [28], and especially esters of the alcohol increased the potency significantly. In this study, we evaluate how modifications on ring B and C affect the antiparasitic activity against *T. cruzi*, *L. braziliensis* and *L. amazonensis*. As a comparison, the cytotoxicity to mammalian murine macrophage cell lines (RAW) was determined, and the quotas IC_50_ RAW cells/ IC_50_ parasite is given as the selectivity index (SI) in Table 1.

#### 2.3.1. Antiparasitic Activity Against *Trypanosoma cruzi* Epimastigotes

Compared to pulchrol (**1**), the 6,6-didemethyl analogue **8a** is considerably less active (66.6 μM), indicating the importance of alkyl substituents on C-6 for the activity against *T. cruzi*. However, the toxicity to mammalian cells (SI) is also less. The 6-methyl enantiomers **8b** and **8c** were less potent than **1**, as are the 6-ethyl enantiomers **8d** and **8e**, suggesting the importance of a dialkylated C-6. In **8f** the two methyls of **1** have been exchanged for ethyls and compared to **1** as well as benznidazole the antiparasitic activity (10.4 μM) is higher. In addition, **8f** showed the highest selectivity (SI 4.2) among all molecules assayed towards *T. cruzi* in this study. Unlike **8f**, analogue **8g** with two *n*-butyl substituents was slightly less potent (22.8 μM) than **1**, and less selective than **8f**. A possibility is that the compounds for their effect on *T. cruzi* interact with a lipophilic pocket in a target protein around C-6, although its volume is limited.

Changing the position of the methoxy substituent in the C-ring to positions C-1 (compound **10a**) and C-3 (compound **10b**) was not beneficial, and the SI-value was lower. A methoxy group in the C-ring is only efficient in position 2, as in pulchrol (**1**), and the replacement of the methoxy groups in positions 2 and 3 with methyls (compounds **10c** and **10d**) resulted in equipotent compounds that were more active than **10a** and **10b** but less active than **1**. The analogue with no substituent in the C-ring, **10e**, was slightly less potent than **10c** and **10d**. It is possible that a methoxy group in position 2 enables a hydrogen bond at the target, while a methyl group in position 3 is better than a methoxy or no substituent at all. To further explore the effects of alkyl substituents in the positions 2 and 3, the isopropyl analogues **10f** and **10g** were prepared and assayed. Both were more potent than **1**, and comparable with the 6,6-diethyl analogue **8f**. Finally, **10h**, with a *n*-pentyl group in position 2, was found to possess the highest activity towards *T. cruzi* of all compounds assayed in this investigation (6.4 μM), being approximately three times as potent as pulchrol (**1**) and the positive control benznidazole. This contradicts the suggestion that the methoxy substituent at C-2 enables a hydrogen bond, and instead propose that the lipophilicity of the pulchrol analogues is correlated with the antiparasitic activity towards *T. cruzi*.

#### 2.3.2. Antiparasitic Activity Against *Leishmania braziliensis* Promastigotes

Similar to the results obtained for *T. cruzi*, the 6,6-didemethyl analogue **8a** was considerably less potent than **1**, and this is also true for the monomethyl enantiomers **8b** and **8c**. However, the monoethyl enantiomers **8d** and **8e** as well as the 6,6-diethyl analogue **8f** were more potent towards *L. braziliensis* and actually slightly more so compared to **1**. For the 6,6-dibutyl analogue **8g** with the IC_50_-value 29.3 μM this trend is even stronger. Towards *L. braziliensis* the positioning of the methoxy group in the C-ring at C-3 (**10a**) or C-1 (**10b**) instead of C-2 (**1**), as well as replacing the methoxy substituent at C-2 and C-3 for a methyl (analogues **10c** and **10d**) results in almost equipotent compounds that are slightly more potent than **1**. The analogue without substituents in the C-ring (**10e**) is less impressive, while the compounds with bigger alkyl substituents at C-2 and C-3 are the most potent towards *L. braziliensis*. The C-2 isopropyl analogue **10f**, as well as **10g** (C-3 isopropyl) and **10h** (C-2 *n*-pentyl) were all considerably more potent than **1** towards *L. braziliensis*, with IC_50_-values between 15 and 20 μM, close to that of the positive control miltefosine. However, their selectivity for the parasite over the mammalian cells was less impressing.

#### 2.3.3. Antiparasitic Activity Against *Leishmania amazonensis* Promastigotes

As can be seen in Table 1, the antiparasitic activity towards *L*. *amazonensis* is not improved compared to **1** by replacing the 6,6-dimethyl substituents in **1** for hydrogens (**8a**), one methyl and one hydrogen (**8b** and **8c**), or one ethyl and one hydrogen (**8d** and **8f**). More potent analogues are the 6,6-diethyl and 6,6-dibutyl analogues with IC_50_-values of 36.9 and 25.4 μM, respectively. This is similar to what was observed with *T. cruzi* and *L. braziliensis* (*vide supra*). For the C-ring analogues, there is a strong variation in the potency depending on the position of the methoxy group, and while the C-3 methoxy analogue **10a** is slightly more potent than **1**, the C-1 methoxy analogue (**10b**) is considerably less potent. Methyl groups in positions 2 and 3 (**10c** and **10d**) do not really change things, neither does the nonsubstituted **10e**. Again, the most potent analogues are those with larger alkyl groups in positions 2 and 3 (**10f**, **10g** and **10h**).

## 3. Materials and Methods

### 3.1. General

^1^H-NMR spectra (400 MHz) and ^13^C-NMR spectra (100 MHz) were recorded in CDCl_3_ with a Bruker Avance II instrument (Bruker Biospin AG, Fällanden, Switzerland). The individual 1D signals were assigned using 2D NMR experiments (COSY, HSQC, HMBC). The chemical shifts are given in ppm with the solvent signal as reference (7.27 ppm for ^1^H and 77.0 for ^13^C). Infrared spectra were recorded with a Bruker Alpha-P FT/IR instrument (Bruker Biospin AG) with a Diamond ATR sensor as films, and the intensities are given as vw (very weak), w (weak), m (medium), s (strong) and vs (very strong). High resolution mass spectra (HRMS) were recorded with a Waters XEVO-G2 QTOF instrument (Waters Corp, Milford, MA, USA) equipped with electrospray ionization (ESI). A weak solution (10 mg/mL) was leaked into the ionizing unit, and the mass spectrum was recorded. Synthetic reactions were monitored by TLC using alumina plates coated with silica gel and visualized using either UV light and/or spraying/heating with vanillin/H_2_SO_4_. Flash chromatography was performed with silica gel (35–70 μm, 60 Å). Chiral separations were performed by semipreparative HPLC (mod. 1260 Infinity system, column CHIRALPAK^®^ IB, 4 mL/min, 96:4 hexane/isopropyl, UV detector 254 nm, Agilent (Santa Clara, CA, USA). THF was distilled from sodium, acetonitrile was distilled from CaH_2_ and other reaction solvents were dried with Al_2_O_3_. Commercially available compounds were obtained from Aldrich (St. Louis, MO, USA). More detailed data are available in the supplementary materials.

### 3.2. Synthetic Procedures

*Methyl 4-(hydroxymethyl)-2-iodobenzoate* (intermediate in the synthesis of **4**), BH_3_-THF (1 M, 68 mL, 68.2 mmol) was slowly added to a stirred solution of 3-iodo-4-(methoxycarbonyl)benzoic acid (5.2 g, 17.1 mmol) in dry THF (250 mL) at 0 °C. After 30 h, saturated aqueous NaHCO_3_/H_2_O was added, and the aqueous phase was extracted with ethyl acetate (3 × 250 mL) before drying (Na_2_SO_4_) and removal of solvent under reduced pressure. Purification by column chromatography (SiO_2_, 4:6 heptane/ethyl acetate) gave (3.65 g, 73%) of the pure product as yellow crystals, identical to that previously reported [26].

*Methyl 4-(((tert-butyldiphenylsilyl)oxy)methyl)-2-iodobenzoate* (used in the synthesis of **4**), TBDPSCl (4.2 mL, 16.0 mmol) was added to a stirred solution of methyl 4-(hydroxymethyl)-2-iodobenzoate (prepared as described above, 3.90 g, 13.3 mmol) in pyridine (50 mL) at rt. After 24 h, saturated aqueous NH_4_Cl/H_2_O was added and the aqueous phase was extracted with diethyl ether (3 × 200 mL), the organic phase was washed with brine (2 × 500 mL) before drying (Na_2_SO_4_) and removal of solvent under reduced pressure. Purification by column chromatography (SiO_2_, 20:2 heptane/ethyl acetate) gave (4.3 g, 61%) of the pure product as white crystals, identical to that previously reported [26].

*General procedure for Suzuki coupling* (compound **4** and intermediates in the synthesis of **10a**–**10h**). The corresponding boronic acid (1.5 equiv), K_2_CO_3_ (5 equiv) and tetrakis(triphenylphosphine)-palladium(0) (0.17 equiv), were added to a stirred solution of methyl 4-(((*tert*-butyl-diphenylsilyl)oxy)methyl)-2-iodobenzoate (prepared as described above, 1 equiv) dissolved in 4:1 DME/water (15 mL), the mixture (contained in a microtube) was degasified under vaccum/N_2_ at −78 °C five times. The microwave reaction conditions were 100 °C, high pressure, and 10 s of pre-stirring. After 30 to 60 min in the microwave reactor, the mixture was filtered through a plug of celite and washed with ethyl acetate (250 mL) before drying (Na_2_SO_4_) and removal of solvent under reduced pressure. Purification by column chromatography (SiO_2_, 20:3 heptane/ethyl acetate) gave the pure products.

*Methyl 5-(((tert-butyldiphenylsilyl)oxy)methyl)-2′,5′-dimethoxy-[1,1′-biphenyl]-2-carboxylate* (**4**) The pure product was obtained as an orange wax (yield 91 %) identical to that previously reported [26].

*Methyl 5-(((tert-butyldiphenylsilyl)oxy)methyl)-2′,4′-dimethoxy-[1,1′-biphenyl]-2-carboxylate* (used in the synthesis of **10a**), the pure product was obtained as an orange wax (yield 75%). ^1^H-NMR δ 7.83 (d, *J* = 8.0 Hz, 1H), 7.72–7.67 (m, 4H), 7.46–7.41 (m, 2H), 7.41–7.37 (m, 4H), 7.36 (t, *J* = 1.5 Hz, 1H), 7.27 (d, *J* = 2.3 Hz, 1H), 7.15 (d, *J* = 8.4 Hz, 1H), 6.56 (dd, *J* = 8.3, 2.4 Hz, 1H), 6.48 (d, J = 2.4 Hz, 1H), 4.82 (s, 2H), 3.85 (s, 3H), 3.70 (s, 3H), 3.68 (s, 3H), 1.10 (s, 9H). ^13^C-NMR δ 168.94, 160.57, 157.19, 144.87, 138.54, 135.69, 133.41, 130.43, 130.30, 129.91, 129.66, 128.97, 127.90, 124.32, 123.56, 104.40, 98.34, 65.23, 55.48, 55.33, 51.80, 26.96, 19.45. HRMS-ESI+ (*m*/*z*): [M + Na]^+^ calcd for C_33_H_36_O_5_NaSi, 563.2230; found, 563.2225. IR (cm^−1^): 2952 (w, CH aliphatic), 2933 (w, CH aliphatic), 2857 (w, C-H aliphatic), 1727 (s, C=O), 1610 (m, C C aromatic), 1462 (m, C-C aromatic), 1286 (s, C-O), 1208 (m, C-O), 1110 (s, C-O), 824 (m), 704 (s), 505 (m).

*Methyl 5-(((tert-butyldiphenylsilyl)oxy)methyl)-2′,6′-dimethoxy-[1,1′-biphenyl]-2-carboxylate* (used in the synthesis of **10b**), the pure product was obtained as an orange wax (yield 90%). ^1^H-NMR δ 7.94 (d, *J* = 8.0 Hz, 1H), 7.72–7.68 (m, 4H), 7.45–7.41 (m, 2H), 7.40–7.37 (m, 4H), 7.36 (t, *J* = 1.6 Hz, 1H), 7.34 (d, *J* = 1.1 Hz, 1H), 7.29 (d, *J* = 8.4 Hz, 1H), 6.63 (d, *J* = 8.4 Hz, 2H), 4.83 (s, 2H), 3.70 (s, 6H), 3.64 (s, 3H), 1.09 (s, 9H). ^13^C-NMR δ 168.21, 157.19, 144.49, 135.70, 135.02, 133.48, 130.24, 130.06, 129.91, 129.86, 128.78, 127.87, 124.47, 119.17, 104.10, 65.25, 55.94, 51.69, 26.92, 19.45. HRMS-ESI+ (*m*/*z*): [M + Na]^+^ calcd for C_33_H_36_O_5_NaSi, 563.2229; found, 563.2230. IR (cm^−1^): 2932 (w, C-H aliphatic), 2856 (w, C-H aliphatic), 1729 (m, C=O), 1610 (w, C=C aromatic), 1470 (m, C-C aromatic), 1285 (m, C-O), 1244 (m, C-O), 1110 (s, C-O), 823 (w), 704 (m), 505 (w).

*Methyl 5-(((tert-butyldiphenylsilyl)oxy)methyl)-2′-methoxy-5′-methyl-[1,1′-biphenyl]-2-carboxylate* (used in the synthesis of **10c**), the pure product was obtained as a yellowish wax (yield 92%). ^1^H-NMR δ 7.86 (d, *J* = 8.0 Hz, 1H), 7.73–7.69 (m, 4H), 7.45 (td, *J* = 5.6, 2.2 Hz, 2H), 7.40 (dd, *J* = 7.8, 2.0 Hz, 4H), 7.37 (d, *J* = 1.7 Hz, 1H), 7.29 (d, *J* = 1.8 Hz, 1H), 7.12 (dd, *J* = 8.3, 2.4 Hz, 1H), 7.06 (d, *J* = 2.5 Hz, 1H), 6.80 (d, *J* = 8.4 Hz, 1H), 4.84 (s, 2H), 3.70 (s, 3H), 3.68 (s, 3H), 2.34 (s, 3H), 1.12 (s, 9H). ^13^C-NMR δ 168.77, 154.14, 144.91, 138.87, 135.69, 133.43, 130.79, 130.44, 130.31, 129.99, 129.91, 129.61, 129.17, 128.96, 127.90, 124.58, 110.16, 65.26, 55.45, 51.76, 26.96, 20.68, 19.47. HRMS-ESI+ (*m*/*z*): [M + H]^+^ calcd for C_33_H_37_O_4_Si, 525.2461; found, 525.2452. IR (cm^–1^): 2950 (m, C-H aliphatic), 1727 (s, C=O), 1503 (m, C-C aromatic), 1429 (m, C-C aromatic), 1289 (s, C-O), 1108 (vs, C-O), 807 (w), 703 (vs), 505 (m).

*Methyl 5-(((tert-butyldiphenylsilyl)oxy)methyl)-2′-methoxy-4′-methyl-[1,1′-biphenyl]-2-carboxylate* (used in the synthesis of **10d**), the pure product was obtained as a yellowish wax (yield 80%). ^1^H-NMR δ 7.83 (d, *J* = 7.9 Hz, 1H), 7.71–7.67 (m, 4H), 7.46–7.41 (m, 2H), 7.40–7.37 (m, 4H), 7.36 (t, *J* = 1.6 Hz, 1H), 7.28 (d, *J* = 2.2 Hz, 1H), 7.11 (d, *J* = 7.6 Hz, 1H), 6.84 (ddd, *J* = 7.6, 1.6, 0.7 Hz, 1H), 6.71 (d, *J* = 2.1 Hz, 1H), 4.81 (s, 2H), 3.71 (s, 3H), 3.68 (s, 3H), 2.40 (s, 3H), 1.09 (s, 9H). ^13^C-NMR (101 MHz, CDCl_3_) δ 168.81, 156.02, 144.88, 138.95, 138.89, 135.70, 133.43, 130.29, 129.91, 129.82, 129.62, 128.98, 127.91, 124.46, 121.55, 111.26, 65.27, 55.29, 51.78, 26.97, 21.85, 19.46. HRMS-ESI+ (*m*/*z*): [M + H]^+^ calcd for C_33_H_37_O_4_Si, 525.2461; found, 525.2472. IR (cm^−1^): 2931 (w, C-H aliphatic), 2857 (w, C-H aliphatic), 1721 (m, C=O), 1610 (w, C=C aromatic), 1462 (vw, C-C aromatic), 1428 (w, C-C aromatic), 1282 (s, C-O), 1092 (vs, C-O), 818 (m), 702 (vs), 505 (m).

*Methyl 5-(((tert-butyldiphenylsilyl)oxy)methyl)-2′-methoxy-[1,1′-biphenyl]-2-carboxylate* (intermediate in the synthesis of **10e**), the pure product was obtained as a yellowish wax (yield 91%). ^1^H-NMR δ 7.95 (d, *J* = 8.0 Hz, 1H), 7.82–7.74 (m, 4H), 7.51 (dq, *J* = 2.1, 1.3 Hz, 1H), 7.49–7.47 (m, 2H), 7.44 (tt, *J* = 7.8, 1.5 Hz, 4H), 7.41–7.36 (m, 2H), 7.30 (dd, *J* = 7.5, 1.8 Hz, 1H), 7.10 (td, *J* = 7.5, 1.1 Hz, 1H), 6.96 (dd, *J* = 8.3, 1.1 Hz, 1H), 4.91 (d, *J* = 0.9 Hz, 2H), 3.78 (s, 3H), 3.72 (s, 3H), 1.18 (s, 9H). ^13^C-NMR δ 168.61, 156.17, 144.89, 138.83, 135.63, 133.34, 130.74, 130.22, 129.98, 129.88, 129.65, 128.88 (d, *J* = 3.7 Hz), 127.87, 124.61, 120.83, 110.16, 65.21, 55.28, 51.68, 26.93, 19.40. HRMS-ESI+ (*m*/*z*): [M + H]^+^ calcd for C_32_H_35_O_4_Si, 511.2305; found, 511.2302. IR (cm^−1^): 2931 (w, C-H aliphatic), 1726 (m, C=O), 1429 (w, C-C aromatic), 1249 (m, C-O), 1106 (vs, C-O), 823 (w), 703 (vs), 505 (m).

*Methyl 5-(((tert-butyldiphenylsilyl)oxy)methyl)-5′-isopropyl-2′-methoxy-[1,1′-biphenyl]-2-carboxylate* (used in the synthesis of **10f**), the pure product was obtained as a colorless wax (yield 90%). ^1^H-NMR δ 7.84 (d, *J* = 7.9 Hz, 1H), 7.72–7.69 (m, 4H), 7.47–7.42 (m, 2H), 7.41–7.38 (m, 4H), 7.38–7.37 (m, 1H), 7.37 (d, *J* = 1.0 Hz, 1H), 7.18 (ddd, *J* = 8.4, 2.4, 0.7 Hz, 1H), 7.13 (d, *J* = 2.4 Hz, 1H), 6.83 (d, *J* = 8.4 Hz, 1H), 4.85 (s, 2H), 3.71 (s, 3H), 3.67 (s, 3H), 2.91 (hept, *J* = 6.9 Hz, 1H), 1.27 (d, *J* = 6.9 Hz, 6H), 1.11 (s, 9H). ^13^C-NMR δ 168.92, 154.25, 144.86, 141.15, 139.05, 135.68, 133.40, 130.39, 130.32, 129.92, 129.52, 128.94, 128.30, 127.91, 126.50, 124.43, 110.04, 65.16, 55.43, 51.78, 33.43, 26.95, 24.35, 19.46. HRMS-ESI+ (*m*/*z*): [M + H]^+^ calcd for C_35_H_41_O_4_Si, 553.2774; found, 553.2773. IR (cm^−1^): 2956 (m, C-H aliphatic), 1727 (s, C=O), 1609 (w, C=C aromatic), 1500 (m, C-C aromatic), 1429 (m, C-C aromatic), 1288 (s, C-O), 1106 (vs, C-O), 821 (m), 703 (vs), 505 (m).

*Methyl 5-(((tert-butyldiphenylsilyl)oxy)methyl)-2′-methoxy-4′-isopropyl-[1,1′-biphenyl]-2-carboxylate* (used in the synthesis of **10g**), the pure product was obtained as a yellowish wax (yield 38%). ^1^H-NMR δ 7.83 (d, *J* = 8.1 Hz, 1H), 7.71–7.66 (m, 4H), 7.45–7.41 (m, 2H), 7.40–7.37 (m, 4H), 7.35 (d, *J* = 1.7 Hz, 1H), 7.27 (d, *J* = 1.8 Hz, 1H), 7.14 (d, *J* = 7.7 Hz, 1H), 6.89 (dd, *J* = 7.7,1.7Hz,1H), 6.75 (d, *J* = 1.7Hz, 1H), 4.81 (s, 2H), 3.72 (s, 3H), 3.66 (s, 3H), 2.94 (hept, *J* = 7.0 Hz, 1H), 1.30 (d, *J* = 7.0 Hz, 6H), 1.09 (s, 9H). ^13^C-NMR δ 168.90, 156.16, 150.03, 144.81, 138.92, 135.72, 133.50, 130.40, 129.91, 129.65, 129.09, 128.17, 127.91, 124.49, 118.73, 108.77, 65.35, 55.31, 51.71, 34.39, 27.00, 24.13, 19.47. HRMS-ESI+ (*m*/*z*): [M + H]^+^ calcd for C_35_H_41_O_4_Si, 553.2724; found, 553.2770. IR (cm^−1^): 2957 (w, C-H aliphatic), 1722 (m, C=O), 1609 (w, C=C aromatic), 1461 (m, C-C aromatic), 1428 (m, C-C aromatic), 1254 (s, C-O), 1104 (vs, C-O), 822 (s), 700 (vs), 504 (vs).

*Methyl 5-(((tert-butyldiphenylsilyl)oxy)methyl)-2′-methoxy-5′-pentyl-[1,1′-biphenyl]-2-carboxylate* (used in the synthesis of **10h**), the pure product was obtained as a yellowish wax (yield 91%) ^1^H-NMR δ 7.83 (d, *J* = 8.0 Hz, 1H), 7.72–7.67 (m, 4H), 7.45–7.41 (m, 2H), 7.40–7.37 (m, 4H), 7.36 (t, *J* = 1.6 Hz, 1H), 7.31 (d, *J* = 1.7 Hz, 1H), 7.12 (dd, *J* = 8.3, 2.3 Hz, 1H), 7.05 (d, *J* = 2.3 Hz, 1H), 6.80 (d, *J* = 8.3 Hz, 1H), 4.83 (s, 2H), 3.69 (s, 3H), 3.66 (s, 3H), 2.62–2.55 (m, 2H), 1.67–1.58 (m, 2H), 1.33 (ddd, *J* = 7.1, 4.0, 2.9 Hz, 4H), 1.10 (s, 9H), 0.93–0.85 (m, 3H). ^13^C-NMR δ 168.85, 154.31, 144.86, 139.05, 135.72, 135.23, 133.48, 130.47, 130.41, 130.18, 129.91, 129.60, 128.99, 128.49, 127.91, 124.53, 110.16, 65.28, 55.48, 51.73, 35.26, 31.70, 31.50, 26.99, 22.69, 19.48, 14.20. HRMS-ESI+ (*m*/*z*): [M + H]^+^ calcd for C_37_H_45_O_4_Si, 581.3087; found, 581.3097. IR (cm^−1^): 2929 (m, C-H aliphatic), 2857 (w, C-H aliphatic), 1725 (s, C=O), 1609 (w, C=C aromatic), 1500 (m, C-C aromatic), 1429 (m, C-C aromatic), 1285 (s, C-O), 1107 (vs, C-O), 823 (m), 703 (s), 505 (m).

*General procedure for organolithium addition* (compound **6** and intermediates in the synthesis of **8f**, **8g**, and **10a**–**10h**), corresponding organolithium reagent (4 equiv) was added to a stirred solution of the Suzuki coupling product (1 equiv) in dry THF (70 mL), at 0 or −78 °C, depending on the organo-lithic reagent. After 12 h, saturated aqueous NH_4_Cl/H_2_O was added, and the aqueous phase was extracted with ethyl acetate (3 × 100 mL) before drying (Na_2_SO_4_) and removal of solvent under reduced pressure. Purification by column chromatography (SiO_2_, 20:4 heptane/ethyl acetate 20:4) gave the pure product.

*2-(5-(((tert-Butyldiphenylsilyl)oxy)methyl)-2′,4′-dimethoxy-[1,1′-biphenyl]-2-yl)propan-2-ol* (used in the synthesis of **10a**), the pure product was obtained as a light yellow wax (yield 45%). ^1^H-NMR δ 7.70–7.68 (m, 4H), 7.58 (d, *J* = 8.3 Hz, 1H), 7.43 (td, *J* = 3.0, 1.8 Hz, 1H), 7.40 (q, *J* = 1.6 Hz, 2H), 7.38 – 7.36 (m, 4H), 7.05–7.02 (m, 1H), 6.92 (d, *J* = 1.9 Hz, 1H), 6.54–6.51 (m, 2H), 4.75 (d, *J* = 4.3 Hz, 2H), 3.85 (s, 3H), 3.72 (s, 3H), 1.52 (s, 3H), 1.39 (s, 3H), 1.07 (s, 9H). ^13^C-NMR δ 160.45, 157.33, 145.68, 139.21, 135.76, 133.65, 131.60, 130.84, 129.78, 127.91, 127.81 (d, *J* = 2.2 Hz), 126.00, 125.27, 104.10, 98.76, 77.36, 65.25, 55.51 (d, *J* = 4.8 Hz), 32.10, 31.44, 26.98, 19.46. HRMS-ESI+ (*m*/*z*): [M + Na]^+^ calcd for C_34_H_40_O_4_NaSi, 563.2594; found, 563.2591. IR (cm^−1^): 3500 (vw, br, O-H), 2957 (m, C-H aliphatic), 2931 (m, C-H aliphatic), 2857 (m, C-H aliphatic), 1610 (m, C=C aromatic), 1463 (m, C-C aromatic), 1208 (s, C-O), 1111 (vs, C-O), 824 (m), 704 (s), 505 (m).

*2-(5-(((tert-Butyldiphenylsilyl)oxy)methyl)-2′,6′-dimethoxy-[1,1′-biphenyl]-2-yl)propan-2-ol* (used in the synthesis of **10b**), the pure product was obtained as a light yellow wax (yield 50%). ^1^H-NMR δ 7.70–7.67 (m, 4H), 7.61 (d, *J* = 8.1 Hz, 1H), 7.41 (d, *J* = 2.8 Hz, 2H), 7.39 (t, *J* = 1.5 Hz, 1H), 7.38–7.34 (m, 4H), 7.31 (t, *J* = 8.4 Hz, 1H), 6.90 (d, *J* = 2.0 Hz, 1H), 6.63 (d, *J* = 8.5 Hz, 2H), 4.76 (s, 2H), 3.71 (s, 6H), 1.44 (s, 6H), 1.07 (s, 9H). ^13^C-NMR δ 157.39, 145.52, 139.54, 135.78, 133.72, 132.07, 130.71, 129.74, 129.01, 127.77, 126.01, 125.24, 121.52, 104.11, 73.18, 65.32, 55.75, 30.87, 26.97, 19.46. HRMS-ESI+ (*m*/*z*): [M + Na]^+^ calcd for C_34_H_40_O_4_NaSi, 563.2594; found, 563.2592. IR (cm^−1^): 3500 (vw, br, O-H), 2958 (w, C-H aliphatic), 2931 (w, C-H aliphatic), 2857 (vw, C-H aliphatic), 1589 (w, C=C aromatic), 1470 (m, C-C aromatic), 1248 (m, C-O), 1110 (vs, C-O), 824 (w), 703 (m), 505 (w).

*2-(5-(((tert-Butyldiphenylsilyl)oxy)methyl)-2′-methoxy-5′-methyl-[1,1′-biphenyl]-2-yl)propan-2-ol* (used in the synthesis of **10c**), the pure product was obtained as a transparent wax (yield 49%). ^1^H-NMR δ 7.75–7.71 (m, 4H), 7.61 (d, *J* = 8.1 Hz, 1H), 7.48–7.44 (m, 2H), 7.43 (d, *J* = 1.5 Hz, 1H), 7.42–7.38 (m, 4H), 7.15 (dd, *J* = 8.4, 3.1 Hz, 1H), 6.97 (dd, *J* = 11.6, 2.3 Hz, 2H), 6.86 (d, *J* = 8.4 Hz, 1H), 4.80 (d, *J* = 5.1 Hz, 2H), 3.74 (s, 3H), 2.34 (s, 3H), 1.58 (s, 3H), 1.43 (s, 3H), 1.12 (s, 9H). ^13^C-NMR δ 154.15, 145.26, 139.14, 136.17, 135.73, 133.65, 132.75, 132.02, 130.36, 129.76 (d, *J* = 1.8 Hz), 129.09, 127.79 (d, *J* = 2.9 Hz), 126.02, 125.30, 110.81, 73.64, 65.30, 55.56, 32.07, 31.48, 26.98, 20.59, 19.44. HRMS-ESI+ (*m*/*z*): [M + NH_4_]^+^ calcd for C_34_H_44_NO_3_Si, 542.3090; found, 542.3094. IR (cm^−1^): 3500 (vw, br, O-H), 2930 (m, C-H aliphatic), 1502 (m, C-C aromatic), 1428 (m, C-C aromatic), 1236 (m, C-O), 1110 (vs, C-O), 824 (m), 703 (vs), 505 (m).

*2-(5-(((tert-Butyldiphenylsilyl)oxy)methyl)-2′-methoxy-4′-methyl-[1,1′-biphenyl]-2-yl)propan-2-ol* (used in the synthesis of **10d**), the pure product was obtained as a transparent wax (yield 71%). ^1^H-NMR δ 7.68 (ddd, *J* = 7.9, 5.3, 1.5 Hz, 4H), 7.57 (d, *J* = 8.3 Hz, 1H), 7.45–7.40 (m, 2H), 7.39 (d, *J* = 1.6 Hz, 1H), 7.39–7.35 (m, 4H), 7.01 (d, *J* = 7.6 Hz, 1H), 6.92 (d, *J* = 1.7 Hz, 1H), 6.80 (ddd, *J* = 7.6, 1.6, 0.8 Hz, 1H), 6.75 (s, 1H), 4.74 (d, *J* = 4.0 Hz, 2H), 3.73 (s, 3H), 2.40 (s, 3H), 1.53 (s, 3H), 1.37 (s, 3H), 1.07 (s, 9H).^13^C-NMR δ 156.12, 146.42, 138.87, 136.18, 135.76, 133.67, 131.09, 130.55, 129.78, 127.80, 125.99, 125.27, 121.08, 111.83, 73.65, 65.30, 55.42, 32.08, 31.48, 26.99, 21.79, 19.46. HRMS-ESI+ (*m*/*z*): [M + Na]^+^ calcd for C_34_H_40_O_3_NaSi, 547.2644; found, 547.2642. IR (cm^−1^): 3421 (vw, br, O-H), 2930 (w, C-H aliphatic), 2857 (w, C-H aliphatic), 1606 (vw, C=C aromatic), 1502 (w, C-C aromatic), 1427 (w, C-C aromatic), 1236 (w, C-O), 1110 (m, C-O), 823 (w), 703 (m), 505 (w).

*2-(5-(((tert-Butyldiphenylsilyl)oxy)methyl)-2′-methoxy-[1,1′-biphenyl]-2-yl)propan-2-ol* (used in the synthesis of **10e**), The pure product was obtained as a transparent wax (yield 93%). ^1^H-NMR δ 7.85 (ddd, *J* = 7.7, 5.5, 1.8 Hz, 4H), 7.77 (d, *J* = 8.3 Hz, 1H), 7.53 (d, *J* = 1.7 Hz, 1H), 7.51 (dd, *J* = 3.6, 1.8 Hz, 2H), 7.50–7.45 (m, 4H), 7.28 (dd, *J* = 7.4, 1.9 Hz, 1H), 7.11 (s, 2H), 7.05 (d, *J* = 8.4 Hz, 1H), 5.28 (s, 1H), 4.93 (d, *J* = 3.7 Hz, 2H), 3.83 (s, 3H), 1.67 (s, 3H), 1.52 (s, 3H), 1.25 (s, 9H). ^13^C-NMR δ 156.24, 145.34, 139.04, 136.01, 135.62, 133.52, 132.96, 131.23, 130.22, 129.71, 128.72, 127.74, 125.99, 125.26, 120.25, 110.73, 73.47, 65.22, 55.27, 32.04, 31.34, 26.91, 19.35. HRMS-ESI+ (*m*/*z*): [M + NH_4_]^+^ calcd for C_33_H_42_NO_4_Si, 528.2934; found, 528.2920. IR (cm^−1^): 3500 (vw, br, O-H), 2931 (m, C-H aliphatic), 1461 (m, C-C aromatic), 1428 (m, C-C aromatic), 1238 (m, C-O), 1111 (vs, C-O), 823 (m), 703 (vs), 505 (s).

*2-(5-(((tert-Butyldiphenylsilyl)oxy)methyl)-5′-isopropyl-2′-methoxy-[1,1′-biphenyl]-2-yl)propan-2-ol* (used in the synthesis of **10f**), the pure product was obtained as a transparent wax (yield 55%). ^1^H-NMR δ 7.71–7.66 (m, 4H), 7.59 (d, *J* = 8.3 Hz, 1H), 7.46–7.40 (m, 2H), 7.40 (d, *J* = 1.9 Hz, 1H), 7.39–7.35 (m, 4H), 7.17 (ddd, *J* = 8.4, 2.4, 0.6 Hz, 1H), 7.00 (d, *J* = 2.5 Hz, 1H), 6.95 (d, *J* = 2.0 Hz, 1H), 6.86 (d, *J* = 8.5 Hz, 1H), 4.76 (d, *J* = 2.9 Hz, 2H), 3.72 (s, 3H), 2.87 (hept, *J* = 6.8 Hz, 1H), 1.53 (s, 3H), 1.37 (s, 3H), 1.23 (dd, *J* = 6.9, 0.9 Hz, 6H), 1.08 (s, 9H). ^13^C-NMR δ 154.35, 145.25, 140.76, 139.24, 136.50, 135.76, 133.64, 132.55, 130.35, 129.78 (d), 129.55, 127.80 (d), 126.51, 125.99, 125.28, 110.68, 73.57, 65.27, 55.56, 33.36, 32.03, 31.37, 26.98, 24.39, 24.26, 19.46. HRMS-ESI+ (*m*/*z*): [M + Na]^+^ calcd for C_36_H_44_O_3_NaSi, 575.2957; found, 575.2957. IR (cm^−1^): 3500 (vw, br, O-H), 2960 (s, C-H aliphatic), 2932 (m, C-H aliphatic), 2858 (m, C-H aliphatic), 1501 (m, C-C aromatic), 1463 (m, C-C aromatic), 1428 (m, C-C aromatic), 1237 (m, C-O), 1111 (vs, C-O), 823 (m), 703 (vs), 505 (m).

*2-(5-(((tert-Butyldiphenylsilyl)oxy)methyl)-2′-methoxy-4′-isopropyl-[1,1′-biphenyl]-2-yl)propan-2-ol* (used in the synthesis of **10g**), the pure product was obtained as a transparent wax (yield 64%). ^1^H-NMR δ 7.68 (ddd, *J* = 8.1, 5.4, 1.5 Hz, 4H), 7.57 (d, *J* = 8.2 Hz, 1H), 7.45–7.40 (m, 2H), 7.39 (d, *J* = 1.6 Hz, 1H), 7.39–7.34 (m, 4H), 7.04 (d, *J* = 7.7 Hz, 1H), 6.92 (d, *J* = 2.1 Hz, 1H), 6.85 (dd, *J* = 7.7, 1.7 Hz, 1H), 6.78 (d, *J* = 1.8 Hz, 1H), 4.74 (d, *J* = 4.2 Hz, 2H), 3.74 (s, 3H), 2.94 (hept, *J* = 7.0 Hz, 1H), 1.53 (s, 3H), 1.38 (s, 3H), 1.30 (d, *J* = 6.8 Hz, 6H), 1.08 (s, 9H). ^13^C-NMR δ 156.17, 150.05, 145.45, 139.16, 136.20, 135.77, 133.74, 131.14, 130.65, 130.24, 129.77, 127.82, 126.03, 125.31, 118.32, 109.37, 73.72, 65.37, 55.44, 34.38, 32.08, 31.61, 27.02, 24.17, 19.47. HRMS-ESI+ (*m*/*z*): [M + Na]^+^ calcd for C_36_H_44_O_3_NaSi, 575.2957; found, 575.2955. IR (cm^−1^): 3500 (vw, br, O-H), 2959 (m, C-H aliphatic), 2931 (m, C-H aliphatic), 2857 (m, C-H aliphatic), 1461 (m, C-C aromatic), 1427 (m, C-C aromatic), 1106 (s, C-O), 822 (s), 701 (vs), 504 (s).

*2-(5-(((tert-Butyldiphenylsilyl)oxy)methyl)-2′-methoxy-5′-pentyl-[1,1′-biphenyl]-2-yl)propan-2-ol* (used in the synthesis of **10h**) The product was obtained as a mixture and was used directly in the next step (yield 33%).

*3-(5-(((tert-Butyldiphenylsilyl)oxy)methyl)-2′,5′-dimethoxy-[1,1′-biphenyl]-2-yl)pentan-3-ol* (used in the synthesis of **8f**), the pure product was obtained as a light yellow wax (yield 64%). ^1^H-NMR δ 7.70–7.66 (m, 4H), 7.42–7.39 (m, 2H), 7.38 (d, *J* = 0.9 Hz, 2H), 7.37–7.33 (m, 4H), 6.90 (dd, *J* = 1.6, 0.8 Hz, 1H), 6.83–6.82 (m, 2H), 6.67 (dd, *J* = 2.3, 1.2 Hz, 1H), 4.76 (d, *J* = 6.1 Hz, 2H), 3.76 (s, 3H), 3.67 (s, 3H), 1.93 (dd, *J* = 14.0, 7.5 Hz, 1H), 1.73–1.66 (m, 2H), 1.61 (dd, *J* = 13.9, 7.4 Hz, 1H), 1.07 (s, 9H), 0.82 (t, *J* = 7.4 Hz, 3H), 0.71 (t, *J* = 7.4 Hz, 3H). ^13^C-NMR δ 153.07, 150.72, 142.14, 138.68, 136.49, 135.78, 134.42, 133.69, 130.25, 129.77, 127.78 (d), 127.46, 125.07, 116.96, 112.86, 111.45, 65.33, 55.87 (d), 35.15, 34.63, 27.00, 19.46, 8.47, 8.16. HRMS-ESI+ (*m*/*z*): [M + Na]^+^ calcd for C_36_H_44_O_4_NaSi, 591.2907; found, 591.2903. IR (cm^−1^): 3500 (vw, br, O-H), 2960 (s, C-H aliphatic), 2932 (s, C-H aliphatic), 2832 (s, C-H aliphatic), 1503 (m, C-C aromatic), 1463 (m, C-C aromatic), 1427 (m, C-C aromatic), 1218 (s, C-O), 1111 (vs, C-O), 823 (m), 703 (vs), 505 (m).

*5-(5-(((tert-Butyldiphenylsilyl)oxy)methyl)-2′,5′-dimethoxy-[1,1′-biphenyl]-2-yl)nonan-5-ol* (used in the synthesis of **8g**), the pure product was obtained as a light yellow wax (yield 39%). ^1^H-NMR δ 7.70–7.66 (m, 4H), 7.41–7.39 (m, 2H), 7.38 (d, *J* = 3.1 Hz, 2H), 7.37–7.34 (m, 4H), 6.89 (d, *J* = 2.4 Hz, 1H), 6.83–6.81 (m, 2H), 6.65 (dd, *J* = 2.6, 0.9 Hz, 1H), 4.76 (d, *J* = 6.4 Hz, 2H), 3.75 (s, 3H), 3.67 (s, 3H), 1.72–1.56 (m, 4H), 1.37–1.23 (m, 4H), 1.23–1.14 (m, 4H), 1.07 (s, 9H), 0.87 (t, *J* = 7.1 Hz, 3H), 0.82 (t, *J* = 7.1 Hz, 3H). ^13^C-NMR δ 153.06, 150.63, 142.94, 138.59, 136.21, 135.79, 134.27, 133.69, 130.25, 129.77, 127.78 (d), 127.27, 125.08, 116.79, 113.16, 111.39, 78.66, 65.33, 55.84, 55.80, 42.89, 42.57, 27.01, 26.22, 26.00, 23.39, 23.32, 19.46, 14.36, 14.28. HRMS-ESI+ (*m*/*z*): [M + Na − H_2_O]^+^ calcd for C_40_H_50_O_3_NaSi, 629.3427; found, 629.3421. IR (cm^−1^): 3500 (vw, br, O-H), 2955 (vs, C-H aliphatic), 2931 (vs, C-H aliphatic), 2833 (s, C-H aliphatic), 1504 (s, C-C aromatic), 1463 (s, C-C aromatic), 1428 (m, C-C aromatic), 1252 (s, C-O), 1111 (vs, C-O), 824 (m), 702 (vs), 505 (m).

*(5-(((tert-Butyldiphenylsilyl)oxy)methyl)-2′,5′-dimethoxy-[1,1′-biphenyl]-2-yl)methanol* (**5**), DIBALH (1 M, 1.3 mL, 1.3 mmol) was slowly added to a stirred solution of **4** (291 mg, 0.5 mmol) in dry toluene (30 mL) at −78 °C. After 35 min, aqueous HCL (1 N, 10 mL) was added, followed by water (10 mL), and the aqueous phase was extracted with ethyl acetate (3 × 50 mL) before drying (Na_2_SO_4_) and removal of solvent under reduced pressure. Purification by column chromatography (SiO_2_, 2:1 heptane/ethyl acetate) gave the pure product as light yellow wax (230.5 mg, 84%). ^1^H-NMR δ 7.73–7.66 (m, 4H), 7.52 (d, *J* = 7.9 Hz, 1H), 7.47–7.43 (m, 1H), 7.43–7.40 (m, 2H), 7.40–7.33 (m, 4H), 7.17 (d, *J* = 1.5 Hz, 1H), 6.93 (d, *J* = 8.4 Hz, 1H), 6.88 (dd, *J* = 8.9, 2.9 Hz, 1H), 6.73 (d, *J* = 2.3 Hz, 1H), 4.80 (s, 2H), 4.41 (d, *J* = 12.5 Hz, 2H), 3.78 (s, 3H), 3.69 (s, 3H), 1.09 (s, 9H). ^13^C-NMR δ 154.09, 150.71, 140.80, 138.12, 137.42, 135.75, 133.63, 131.32, 129.85, 129.10, 128.01, 127.86, 125.99, 117.05, 113.89, 113.02, 65.46, 63.83, 56.87, 55.87, 27.00, 19.48. HRMS-ESI+ (*m*/*z*): [M + H]^+^ calcd for C_32_H_37_O_4_Si, 495.2355; found, 495.2362. IR (cm^−1^): 3411 (vw, br, O-H), 2931 (w, C-H aliphatic), 2856 (w, C-H aliphatic), 1492 (m, C-C aromatic), 1462 (m, C-C aromatic), 1426 (m, C-C aromatic), 1215 (s, C-O), 1109 (s, C-O), 822 (s), 700 (vs), 503 (s).

*5-(((tert-Butyldiphenylsilyl)oxy)methyl)-2′,5′-dimethoxy-[1,1′-biphenyl]-2-carbaldehyde* (**7**), Morpholine (0.2 mL, 2.2 mmol) was added to a solution of DIBALH (1 M, 1.1 mL, 1.1 mmol) in dry THF (30 mL) at 0 °C. After 3 h, **4** (600 mg, 1.1 mmol) in dry THF (20 mL) was added, 10 min later, DIBALH (1 M, 1.1 mL, 1.1 mmol) was added again at 0 °C. After 4 h, aqueous HCL (1 N, 20 mL) was added, and the aqueous phase was extracted with diethyl ether (3 × 50 mL) before drying (Na_2_SO_4_) and removal of solvent under reduced pressure. Purification by column chromatography (SiO_2_, 20:4 heptane/ethyl acetate) gave the pure product as a light yellow wax (89.1 mg, 16%). ^1^H-NMR δ 9.77 (d, *J* = 0.9 Hz, 1H), 7.98 (d, *J* = 7.9 Hz, 1H), 7.71–7.67 (m, 4H), 7.48 (ddd, *J* = 8.1, 1.7, 0.9 Hz, 1H), 7.46–7.41 (m, 2H), 7.40–7.36 (m, 4H), 7.33 (d, *J* = 1.1 Hz, 1H), 6.94 (dd, *J* = 8.9, 3.1 Hz, 1H), 6.89 (d, *J* = 8.7 Hz, 1H), 6.84 (d, *J* = 2.6 Hz, 1H), 4.85 (s, 2H), 3.80 (s, 3H), 3.68 (s, 3H), 1.11 (s, 9H). ^13^C-NMR δ 192.51, 153.92, 150.90, 147.38, 141.74, 135.71, 133.29, 133.01, 129.99, 128.45, 127.95, 126.95, 125.40, 117.23, 114.74, 111.91, 65.31, 56.06, 55.95, 26.98, 19.48. HRMS-ESI+ (*m*/*z*): [M + H]^+^ calcd for C_32_H_35_O_4_Si, 511.2305; found, 511.2304. IR (cm^−1^): 2931 (m, C-H aliphatic), 2856 (m, C-H aliphatic), 1694 (s, C=O), 1500 (m, C-C aromatic), 1462 (m, C-C aromatic), 1427 (m, C-C aromatic), 1218 (s, C-O), 1111 (s, C-O), 824 (m), 703 (s), 505 (m).

*(2-Methoxy-6H-benzo[c]chromen-9-yl)methanol* (**8a**), NaSEt (100.9 mg, 1.2 mmol) was added to a stirred solution of **5** (150 mg, 0.3 mmol) in dry DMF (2 mL), the mixture was heated to 110 °C. After 6 h, the mixture was cooled to rt, saturated aqueous NH_4_Cl (2 mL) was added, the aqueous phase was extracted with ethyl acetate (3 × 10 mL), and the organic layer was washed with brine (3 × 30 mL), before drying (Na_2_SO_4_) and removal of solvent under reduced pressure. Purification by column chromatography (SiO_2_, 1:1 heptane/ethyl acetate, Sephadex LH20 1:1 chloroform/methanol) gave the pure product as a light yellow wax (4.8 mg, 7%). ^1^H- and ^13^C-NMR data are shown in Table 2 and Table 3. HRMS-ESI+ (*m*/*z*): [M − H]^−^ calcd for C_15_H_13_O_3_, 241.0865; found, 241.0875. IR (cm^−1^): 3435 (vw, br, O-H), 2956 (m, C-H aliphatic), 2919 (m, C-H aliphatic), 2850 (w, C-H aliphatic), 1505 (m, C-C aromatic), 1463 (w, C-C aromatic), 1427 (m, C-C aromatic), 1221 (m, C-O), 1195 (m, C-O), 1040 (m, C-O), 821 (w), 705 (vw), 507 (vw).

*General procedure to prepare compounds***8b**–**8e**. The corresponding organolithium reagent (2 equiv) was added to **7** (1 equiv) in dry THF (5 mL) at 0 °C or −78 °C depending on the organolithium reagent. After 6 h, saturated aqueous NH_4_Cl/H_2_O was added, and the aqueous phase was extracted with ethyl acetate (3 × 20 mL) before drying (Na_2_SO_4_) and removal of solvent under reduced pressure. PBr_3_ (0.34 equiv) was added to the crude product (1 equiv) in dichloromethane (10 mL) at rt. After 2 h, LiI (3 equiv) was added at rt. After 12 h, saturated aqueous Na_2_S_2_O_3_/H_2_O was added, and the aqueous phase was extracted with ethyl acetate (3 × 20 mL) before drying (Na_2_SO_4_) and removal of solvent under reduced pressure. TBAF (2 equiv) was added to the crude product in THF (25 mL) at rt, after 5 h, saturated aqueous NaHCO_3_/H_2_O was added, and the aqueous phase was extracted with ethyl acetate (3 × 25 mL) before drying (Na_2_SO_4_) and removal of solvent under reduced pressure. Purification by column chromatography (SiO_2_, 1:1 heptane/ethyl acetate) and the enantiomers were separated using a semipreparative HPLC (Chiralpack B column, 96:4 hexane/isopropanol).

*(2-Methoxy-6-methyl-6H-benzo[c]chromen-9-yl)methanol* (**8b**), the pure product was obtained as a light yellow wax (yield 3%), [α]^D^_20_ = −20.8°. ^1^H- and ^13^C-NMR data are shown in Table 2 and Table 3. HRMS-ESI+ (*m*/*z*): [M − H]^−^ calcd for C_16_H_15_O_3_, 255.1021; found, 255.1018. IR (cm^−1^): 3401 (w, br, O-H), 2960 (m, C-H aliphatic), 2930 (m, C-H aliphatic), 2867 (w, C-H aliphatic), 1503 (s, C-C aromatic), 1463 (w, C-C aromatic), 1426 (s, C-C aromatic), 1216 (vs, C-O), 1194 (s, C-O), 1039 (s, C-O), 821 (m), 705 (w).

*(2-Methoxy-6-methyl-6H-benzo[c]chromen-9-yl)methanol* (**8c**), the pure product was obtained as a light yellow wax (yield 3%), [α]^D^_20_ = +21.8°. ^1^H- and ^13^C-NMR data are shown in Table 2 and Table 3. HRMS-ESI+ (*m*/*z*): [M − H]^−^ calcd for C_16_H_15_O_3_, 255.1021; found, 255.1028. IR (cm^−1^): 3434 (w, br, O-H), 2931 (m, C-H aliphatic), 2859 (w, C-H aliphatic), 1504 (s, C-C aromatic), 1464 (m, C-C aromatic), 1425 (s, C-C aromatic), 1216 (vs, C-O), 1195 (s, C-O), 1038 (s, C-O), 822 (m), 705 (w).

*(6-Ethyl-2-methoxy-6H-benzo[c]chromen-9-yl)methanol* (**8d**), the pure product was obtained as a light yellow wax (yield 6%), [α]^D^_20_ = −63.2°. ^1^H- and ^13^C-NMR data are shown in Table 2 and Table 3. HRMS-ESI+ (*m*/*z*): [M − H]^−^ calcd for C_17_H_17_O_3_, 269.1178; found, 269.1187. IR (cm^−1^): 3432 (vw, br, O-H), 2930 (w, C-H aliphatic), 2856 (vw, C-H aliphatic), 1502 (m, C-C aromatic), 1463 (m, C-C aromatic), 1426 (m, C-C aromatic), 1217 (m, C-O), 1040 (m, C-O), 822 (w), 704 (w).

*(6-Ethyl-2-methoxy-6H-benzo[c]chromen-9-yl)methanol* (**8e**), the pure product was obtained as a light yellow wax (yield 7%), [α]^D^_20_ = +62.3°. ^1^H- and ^13^C-NMR data are shown in Table 2 and Table 3. HRMS-ESI+ (*m*/*z*): [M − H]^−^ calcd for C_17_H_17_O_3_, 269.1178; found, 269.1178. IR (cm^−1^): 3427 (vw, br, O-H), 2929 (w, C-H aliphatic), 2856 (vw, C-H aliphatic), 1503 (m, C-C aromatic), 1462 (m, C-C aromatic), 1426 (m, C-C aromatic), 1217 (m, C-O), 1041 (m, C-O), 823 (w), 704 (w).

*General procedure to prepare compounds***8f**, **8g**, *and***10a**–**10h**. HI (55%, 10 equiv) was added to a stirred solution of the appropriate starting material in acetonitrile (25 mL), at rt. After 30 min, saturated aqueous Na_2_S_2_O_3_ (25 mL) was added, and the aqueous layer was extracted with ethyl acetate (3 × 50 mL), before drying (Na_2_SO_4_) and removal of solvent under reduced pressure. TBAF (1 M, 1.1 equiv) was added to the crude product in THF (150 mL). After 3 h, aqueous saturated NaHCO_3_ (50 mL) was added, and the aqueous layer was extracted with ethyl acetate (3 × 50 mL), before drying (Na_2_SO_4_) and removal of solvent under reduced pressure. Purification by column chromatography (SiO_2_, 1:1 heptane/ethyl acetate) gave the pure product.

*(6,6-Diethyl-2-methoxy-6H-benzo[c]chromen-9-yl)methanol* (**8f**), the pure product was obtained as a light yellow wax (yield 56%). ^1^H- and ^13^C-NMR data are shown in Table 2 and Table 3. HRMS-ESI+ (*m*/*z*): [M + H]^+^ calcd for C_19_H_23_O_3_, 299.1647; found, 299.1646. IR (cm^−1^): 3400 (vw, br, O-H), 2962 (s, C-H aliphatic), 2928 (vs, C-H aliphatic), 2868 (m, C-H aliphatic), 1595 (m, C=C aromatic), 1505 (m, C-C aromatic), 1463 (s, C-C aromatic), 1256 (s, C-O).

*(6,6-Dibutyl-2-methoxy-6H-benzo[c]chromen-9-yl)methanol* (**8g**), the pure product was obtained as a light yellow wax (yield 13%). ^1^H- and ^13^C-NMR data are shown in Table 2 and Table 3. HRMS-ESI+ (*m*/*z*): [M + H]^+^ calcd for C_23_H_31_O_3_, 355.2273; found, 355.2272. IR (cm^−1^): 3400 (vw, br, O-H), 2957 (vs, C-H aliphatic), 2928 (vs, C-H aliphatic), 2869 (m, C-H aliphatic), 1504 (s, C-C aromatic), 1214 (s, C-O) 1027 (m, C-O).

*(3-Methoxy-6,6-dimethyl-6H-benzo[c]chromen-9-yl)methanol* (**10a**), the pure product was obtained as a light yellow wax (yield 38%). ^1^H- and ^13^C-NMR data are shown in Table 2 and Table 3. HRMS-ESI+ (*m*/*z*): [M + H]^+^ calcd for C_17_H_19_O_3_, 271.1334; found, 271.1338. IR (cm^−1^): 3379 (m, br, O-H), 2966 (m, C-H aliphatic), 2932 (m, C-H aliphatic), 2857 (w, C-H aliphatic), 1615 (vs, C=C aromatic), 1589 (m, C=C aromatic), 1510 (m, C-C aromatic), 1496 (m, C-C aromatic), 1417 (m, C-C aromatic), 1290 (s, C-O), 1273 (s, C-O), 1201 (s, C-O), 1055 (vs, C-O), 1110 (s, C-O), 981(m).

*(1-Methoxy-6,6-dimethyl-6H-benzo[c]chromen-9-yl)methanol* (**10b**), the pure product was obtained as a light yellow wax (yield 48%). ^1^H- and ^13^C-NMR data are shown in Table 2 and Table 3. HRMS-ESI+ (*m*/*z*): [M + H]^+^ calcd for C_17_H_19_O_3_, 271.1334; found, 271.1338. IR (cm^−1^): 3395 (w, br, O-H), 2970 (w, C-H aliphatic), 2930 (w, C-H aliphatic), 2863 (vw, C-H aliphatic), 1600 (m, C=C aromatic), 1586 (m, C=C aromatic), 1503 (m, C-C aromatic), 1462 (s, C-C aromatic), 1436 (m, C-C aromatic), 1413 (m, C-C aromatic), 1234 (vs, C-O), 1088 (vs, C-O), 1080 (vs, C-O).

*(2,6,6-Trimethyl-6H-benzo[c]chromen-9-yl)methanol* (**10c**), the pure product was obtained as a transparent wax (yield 73%). ^1^H- and ^13^C-NMR data are shown in Table 2 and Table 3. HRMS-ESI+ (*m*/*z*): [M + H]^+^ calcd for C_17_H_19_O_2_, 255.1385; found, 255.1383. IR (cm^−1^): 3400 (w, br, O-H), 2978 (m, C-H aliphatic), 1505 (s, C-C aromatic), 1425 (s, C-C aromatic), 1256 (vs, C-O), 1114 (m), 818 (s).

*(3,6,6-Trimethyl-6H-benzo[c]chromen-9-yl)methanol* (**10d**), the pure product was obtained as a transparent wax (yield 85%). ^1^H- and ^13^C-NMR data are shown in Table 2 and Table 3. HRMS-ESI+ (*m*/*z*): [M − OH]^−^ calcd for C_17_H_17_O, 237.1285; found, 237.1281. IR (cm^−1^): 3405 (w, br, O-H), 2928 (w, C-H aliphatic), 1589 (w, C=C aromatic), 1502 (m, C-C aromatic), 1270 (w, C-O), 1115 (w).

*(6,6-Dimethyl-6H-benzo[c]chromen-9-yl)methanol* (**10e**) the pure product was obtained as a transparent wax (yield 68%). ^1^H- and ^13^C-NMR data are shown in Table 2 and Table 3. HRMS-ESI+ (*m*/*z*): [M + H]^+^ calcd for C_16_H_17_O_2_, 241.1229; found, 241.1227. IR (cm^−1^): 3300 (w, br, O-H), 2979 (w, C-H aliphatic), 1589 (w, C=C aromatic), 1492 (m, C-C aromatic), 1419 (m, C-C aromatic), 1253 (vs, C-O), 1105 (m), 752 (s).

*(2-Isopropyl-6,6-dimethyl-6H-benzo[c]chromen-9-yl)methanol* (**10f**), the pure product was obtained as a transparent wax (yield 31%). ^1^H- and ^13^C-NMR data are shown in Table 2 and Table 3. HRMS-ESI+ (*m*/*z*): [M + H]^+^ calcd for C_19_H_23_O_2_, 283.1698; found, 283.1701. IR (cm^−1^): 3361 (m, br, O-H), 2961 (w, C-H aliphatic), 2927 (m, C-H aliphatic), 2870 (m, C-H aliphatic), 1613 (w, C=C aromatic), 1588 (w, C=C aromatic), 1504 (s, C-C aromatic), 1462 (s, C-C aromatic), 1416 (m, C-C aromatic), 1255 (vs, C-O), 1156 (m), 1114 (m), 818 (m).

*(3-Isopropyl-6,6-dimethyl-6H-benzo[c]chromen-9-yl)methanol* (**10g**), the pure product was obtained as a transparent wax (yield 85%). ^1^H- and ^13^C-NMR data are shown in Table 2 and Table 3. HRMS-ESI+ (*m*/*z*): [M − OH]^−^ calcd for C_19_H_21_O_2_, 265.1592; found, 265.1597. IR (cm^−1^): 3439 (vw, br, O-H), 2960 (m, C-H aliphatic), 2931 (m, C-H aliphatic), 2858 (w, C-H aliphatic), 1610 (vw, C=C aromatic), 1567 (vw, C=C aromatic), 1462 (m, C-C aromatic), 1414 (m, C-C aromatic), 1110 (s), 822 (m), 703 (s).

*(6,6-Dimethyl-2-pentyl-6H-benzo[c]chromen-9-yl)methanol* (**10h**), the pure product was obtained as a transparent wax (yield 72%). ^1^H- and ^13^C-NMR data are shown in Table 2 and Table 3. HRMS-ESI+ (*m*/*z*): [M − OH]^−^ calcd for C_21_H_25_O_2_, 293.1905; found, 293.1902. IR (cm^−1^): 3397 (w, br, O-H), 1588 (w, C=C), 1116 (w).

### 3.3. Biological Assays

#### 3.3.1. Evaluations Against *Leishmania parasites*

Promastigotes of Leishmania-Leishmania: *L. amazonensis*, Clone 1, NHOM-BR-76-LTB-012 (Lma, donated by the Paul Sabatier Université, Toulouse, France) and Leishmania-Viannia: *L. braziliensis* M2904 C192 RJA (M2904, donated by Dr. Jorge Arévalo from Universidad Peruana Cayetano Heredia, San Martin des Porres, Peru), [30]. All strains were cultured in Schneider’s insect medium, (pH 6.2) supplemented with 10% FBS and incubated at 26 °C. Medium changes were made every 72 h to maintain a viable parasitic population. Leishmanicidal activity was determined according to Williams with some modifications [31]. Samples were dissolved in DMSO (maximum final concentration 1%) at 10 mg/mL. Promastigotes in logarithmic phase of growth, at the concentration 3 × 106 parasites/mL, were distributed (100 μL/well) in 96-well flat bottom microtiter plates. Samples with different concentrations (3.1–100 μg/mL) were added (100 μL). Miltefosine (3.1–100 μg/mL), was used as control drug [32]. Assays were performed in triplicates. The microwell plates were incubated for 72 h at 26 °C. After incubation, a solution of XTT (1 mg/mL) in PBS (pH 7.0 at 37 °C) with PMS (0.06 mg/mL) was added (50 μL/well), and incubated for 3 h at 26 °C. The optical density of each well was measured and the IC_50_ values calculated. A negative control experiments with only 1% DMSO was carried out, showing that the solvent by itself has no antiparasitic activity.

#### 3.3.2. Evaluations Against *Trypanosoma cruzi*

Cultures of *Trypanosoma cruzi* (epimastigotes, donated by the Parasitology Department of INLASA, Tc-INLASA, city, country), were maintained in medium LIT (pH 7.2), supplemented with 10% FBS and incubated at 26 °C. Medium changes were made every 72 h to maintain a viable parasitic population. Trypanocidal activity was determined according to Muelas-Serrano with some modifications [33]. Samples were dissolved in DMSO (maximum final concentration 1%) at 10 mg/mL. Epimastigotes in logarithmic phase of growth, at a concentration of 3 × 106 parasites/mL, were distributed (100 μL/well) in 96-well flat bottom microtiter plates. Samples at different concentrations (3.1–100 μg/mL) were added (100 μL). Benznidazole (3.1–100 μg/mL) was used as the control drug. Assays were performed in triplicates. The microwell plates were incubated for 72 h at 26 °C. After incubation, a solution of XTT (1 mg/mL) in PBS (pH 7.0 at 37 °C) with PMS (0.06 mg/mL) was added (50 μL/well) and incubated for 4 h at 26 °C. The optical density of each well was measured and the IC_50_ values were calculated. A negative control experiments with only 1% DMSO was carried out, showing that the solvent by itself has no antiparasitic activity.

#### 3.3.3. Evaluations Against RAW Cells

The Raw 264.7 murine macrophage cell line was purchased from the American Type Culture Collection (ATCC-TIB71, ARCC (Manassas, VA, USA). The cells were maintained in DMEM-HG medium supplemented with 10% fetal bovine serum, 100 U/mL of penicillin and 100 μg/mL of streptomycin, and sodium bicarbonate (2.2 g/L) in humidified atmosphere at 37 °C with 5% CO_2_. Samples were dissolved in DMSO and diluted (maximum final concentration of DMSO: 1%) at different concentrations (6.2–200 μg/mL). Medium blank, control drugs and cell growth controls were included to evaluate cell viability. The plates were incubated for 72 h at 37 °C with 5% CO_2_ and 3 × 10^4^ cells/well. After incubation for the indicated time, the cells were washed, after which 10 μL of resazurin reagent (2.0 mM) was added. They were further incubated at 37 °C for 3 h in a humidified incubator. The IC_50_ values were assessed using a fluorometric reader (BioTek (Winooski, VT, USA), 540 nm excitation, 590 nm emission) and the Gen5 software (v. 2017, BioTek). All assays were performed in triplicate.

## 4. Conclusions

Fifteen derivatives of pulchrol with modifications on ring B and ring C were prepared and tested towards *T. cruzi*, *L. braziliensis* and *L. amazonensis*, together with **1**. The importance of the presence of methyl substituents on ring B was investigated, and the unsubstituted derivative **8a** was shown to be less active compared to **1** towards all the three parasites. The effect on bioactivity that just one substituent has on C-6 was different between the parasites. The 6-methyl monosubstituted enantiomers were not more active than **1**, suggesting that two methyl substituents instead of one may improve orientation and lipophilic interactions in the binding site. 6-Ethyl monosubstituted derivatives are slightly more potent towards the two *Leishmania* species, but with *T. cruzi* they are less potent. The longer the alkyl substituents on C-6 are, the more interesting is the activity. A preference for disubstituted rather than monosubstituted analogues appears to be at hand, but with *T. cruzi* the 6,6-diethyl analogue is better that the 6,6-dibutyl analogue, and **8f** was found to be more potent and selective than the positive control benznidazole. This suggests that additional derivatives with larger and branched alkyl groups at C-6 should be prepared and assayed.

The methoxy group in the C-ring was also shown to play a role for pulchrol’s bioactivity, as a derivative without substituents (**10e**) was considerably less active compared to **1**. A methoxy substituent in either C-3 or C-2 appears beneficial compared to C-1, although the differences are not massive. A methyl at C-2 or C-3 instead of a methoxy group has a small impact, although for *T. cruzi* **1** is still the most potent. Longer and more bulky alkyl substituents in positions C-2 and C-3 (**10f**, **10g** and **10h**) are clearly more potent, with all three parasites. The C-2 *n*-pentyl analogue **10h** showed the best activities towards *T. cruzi*, while **10h** together with the C-2 isopropyl analogues **10f** and **10g** showed the best results with *L. braziliensis* and *L. amazonensis*.

Most of the differences in the antiparasitic activity observed in this study can be tentatively suggested to be linked to the lipophilicity of the compounds. However, nothing is known about the molecular targets in these parasites, and to increase our understanding it is necessary to expand our studies in a systematic way. Compared to the QSARs suggested in the previous study of the benzyl alcohol function, we have now a new wish list of compounds to prepare and assay.

The 1D ^1^H and ^13^C-NMR shifts of the assayed compounds are given in Table 2 and Table 3, and as the shifts reflects the electronic conditions in the vicinity of each nucleus they may indicate SARs. However, with the data available in this study, no SARs are obvious from the NMR shifts.

## Figures and Tables

**Table 1:** Antileishmanial, antitrypanozomal and cytotoxic activity of the synthesised derivatives, compared to the positive controls Benznidazole and Miltefosine. The IC_50_ values are given in mM as average data, with the standard deviations from at least three independent experiments. See Experimental for details about the assays.

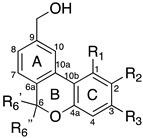												
**Mol.**	**R_6′_**	**R_6′_^’^**	**R_1_**	**R_2_**	**R_3_**	***T. cruzi***		***L. brasiliensis***		***L. amazoniensis***		**Cytotoxicity ^a^**
						**IC_50_ (μM)**	**SI ^b^**	**IC_50_ (μM)**	**SI ^b^**	**IC_50_ (μM)**	**SI ^b^**	**IC_50_ (μM)**
**1**	Me	Me	H	OMe	H	18.5 ± 9.6	1.7	59.2 ± 11.8	0.5	77.7 ± 5.5	0.4	30.7 ± 1.1
**8a**	H	H	H	OMe	H	66.0 ± 18.2	1.3	248.1 ± 54.1	0.3	132.1 ± 29.3	0.6	82.6 ± 9.5
**8b**	Me	H	H	OMe	H	35.9 ± 11.7	1.0	156.1 ± 23.4	0.2	156.1 ± 58.5	0.2	37.1 ± 3.9
**8c**	H	Me	H	OMe	H	67.1 ± 31.2	0.8	128.8 ± 24.6	0.4	71.8 ± 12.5	0.7	52.7 ± 9.0
**8d**	Et	H	H	OMe	H	51.8 ± 9.2	0.6	45.9 ± 20.3	0.7	71.4 ± 1.1	0.4	30.0 ± 3.7
**8e**	H	Et	H	OMe	H	37.0 ± 0.7	1.2	45.1 ± 19.2	1.0	70.3 ± 3.7	0.6	44.4 ± 11.5
**8f**	Et	Et	H	OMe	H	10.4 ± 0.3	4.2	46.9 ± 5.0	0.9	36.9 ± 3.4	1.2	43.6 ± 15.1
**8g**	Bu	Bu	H	OMe	H	22.8 ± 8.5	1.5	29.3 ± 1.4	1.2	25.4 ± 1.1	1.4	35.3 ± 17.5
**10a**	Me	Me	H	H	OMe	88.4 ± 12.2	0.7	37.4 ± 1.1	1.6	66.6 ± 7.4	0.9	59.2 ± 25.9
**10b**	Me	Me	OMe	H	H	92.5 ± 14.8	0.5	48.1 ± 18.5	1.0	179.8 ± 21.9	0.3	48.1 ± 18.5
**10c**	Me	Me	H	Me	H	31.5 ± 7.9	1.3	39.3 ± 2.4	1.0	64.9 ± 12.6	0.6	39.3 ± 1.6
**10d**	Me	Me	H	H	Me	33.0 ± 3.9	1.1	40.1 ± 8.3	0.9	51.9 ± 13.8	0.7	35.4 ± 7.9
**10e**	Me	Me	H	H	H	50.8 ± 6.2	1.2	74.9 ± 16.6	0.8	91.6 ± 5.4	0.7	62.4 ± 6.2
**10f**	Me	Me	H	*i*-Pr	H	12.4 ± 3.5	0.7	18.1 ± 0.7	0.5	15.6 ± 2.8	0.6	8.9 ± 3.5
**10g**	Me	Me	H	H	*i*-Pr	14.2 ± 4.2	1.6	19.1 ± 1.1	1.1	21.2 ± 7.1	1.0	22.0 ± 7.1
**10h**	Me	Me	H	*n*-Pen	H	6.4 ± 0.3	2.0	16.4 ± 0.3	0.8	16.8 ± 1.9	0.8	12.9 ± 3.2
Benznidazole						19.2 ± 7.7	3.9	-	-	-	-	74.7 ± 9.1
Miltefosine						-	-	13.0 ± 1.2	5.9	10.8 ± 1.5	7.1	76.6 ± 3.2

^a^ Toxicity was measured on RAW cells, see Experimental for details, ^b^ SI, selectivity index (cytotoxicity/anti-protozoal activity).

**Table 2:** Proton chemical shifts (in ppm) for the compounds prepared in this study, measured in CDCl_3_ at 400 MHz. The assignments were made with 2D NMR spectroscopy, COSY, HMQC and HMBC experiments.

Compd.	1-H	2-H	3-H	4-H	7-H	8-H	10-H	1′-H/H_2_	2-OCH_3_	6-H/H_2_	6,6-CH_3_
**1**	7.26	-	6.81	6.89	7.23	7.30	7.68	4.74	3.85	-	1.61
**8a**	7.27	-	6.82	6.93	7.15	7.29	7.68	4.76	3.85	5.06	-
**8b**	7.26	-	6.81	6.92	7.15	7.29	7.67	4.75	3.84	5.20	1.60
**8c**	7.26	-	6.82	6.92	7.16	7.29	7.68	4.75	3.85	5.20	1.60
**8d ^a^**	7.25	-	6.81	6.92	7.12	7.28	7.68	4.75	3.85	4.98	-
**8e ^b^**	7.26	-	6.81	6.92	7.12	7.28	7.68	4.75	3.85	4.99	-
**8f ^c^**	7.25	-	6.80	6.87	7.11	7.28	7.71	4.75	3.84	-	-
**8g ^d^**	7.24	-	6.79	6.85	7.11	7.28	7.70	4.75	3.84	-	-
**10a ^e^**	7.65	6.60	-	6.51	7.21	7.24	7.65	4.73	-	-	1.63
**10b ^f^**	-	6.63	7.17	6.64	7.25	7.30	8.43	4.72	-	-	1.61
**10c ^g^**	7.56	-	7.04	6.85	7.23	7.28	7.73	4.74	-	-	1.62
**10d ^h^**	7.62	6.84	-	6.78	7.21	7.24	7.68	4.71	-	-	1.62
**10e**	7.74	7.03	7.24	6.96	7.22	7.27	7.72	4.72	-	-	1.64
**10f ^i^**	7.59	-	7.10	6.87	7.23	7.28	7.75	4.76	-	-	1.62
**10g ^j^**	7.66	6.89	-	6.83	7.22	7.26	7.70	4.73	-	-	1.63
**10h ^k^**	7.55	-	7.05	6.86	7.22	7.27	7.74	4.74	-	-	1.62

^a^ Ethyl signals at 1.89, 1.69 and 1.03 ppm. ^b^ Ethyl signals at 1.89, 1.69 and 1.03 ppm. ^c^ Ethyl signals at 1.91 and 0.88 ppm. ^d^ *n*-butyl signals at 1.86, 1.28, 1.24, 0.83 ppm. ^e^ Methoxy signal at 3.82 ppm. ^f^ Methoxy signal at 3.95 ppm. ^g^ Methyl signal at 2.36 ppm. ^h^ Methyl signal at 2.34 ppm. ^i^ Isopropyl signals at 2.93 and 1.29 ppm. ^j^ Isopropyl signals at 2.88 and 1.26 ppm. ^k^ *n*-Pentyl signals at 2.61, 1.66, 1.36, 0.93 ppm.

**Table 3:** ^13^C-NMR chemical shifts (in ppm) for the assayed compounds **1**, **3a**–**h**, **4a**–**l**, **5a**–**e** and **6** determined at 100 MHz in CDCl_3_. The assignments were made with 2D NMR spectroscopy, COSY, HMQC and HMBC experiments.

Compd	C-1	C-2	C-3	C-4	C-4a	C-6	C-6a	C-7	C-8	C-9	C-10	C-10a	C-10b	C-1′	2-OCH_3_	6,6-CH_3_/6-CH_3_
**1**	108.0	154.6	115.5	118.8	146.9	77.4	139.5	123.7	126.8	140.4	121.0	129.1	123.0	65.3	56.0	27.5
**8a**	108.3	155.0	115.6	118.2	149.0	68.6	131.4	125.1	126.5	141.2	120.8	130.7	123.5	65.4	56.0	-
**8b**	108.1	154.8	115.6	118.6	147.7	73.6	135.8	124.5	126.7	140.8	121.0	129.9	123.2	65.4	56.0	20.1
**8c**	108.1	154.8	115.6	118.6	147.7	73.6	135.8	124.5	126.7	140.8	121.0	129.9	123.2	65.4	56.0	20.1
**8d** ^a^	108.1	154.7	115.5	118.7	146.9	78.9	134.9	125.3	126.5	140.8	121.0	129.6	123.1	65.4	56.0	-
**8e** ^b^	108.1	154.7	115.5	118.7	146.9	78.9	134.9	125.4	126.5	140.8	121.0	129.6	123.1	65.5	56.0	-
**8f** ^c^	107.8	154.2	115.4	118.4	147.1	82.4	136.3	125.2	126.2	139.9	120.8	130.0	122.2	65.3	55.9	-
**8g** ^d^	107.9	154.3	115.6	118.5	147.2	82.2	137.1	125.3	126.4	140.0	120.9	129.9	122.2	65.4	56.0	-
**10a** ^e^	124.0	108.5	161.2	102.9	154.2	78.1	137.9	123.6	125.7	140.4	120.2	129.2	115.4	65.4	-	27.7
**10b** ^f^	157.7	104.7	129.2	111.4	154.7	77.3	139.5	122.9	126.2	139.8	125.8	128.0	112.3	65.8	-	27.1
**10c** ^g^	123.5	130.8	130.4	117.9	150.7	77.5	139.2	123.6	126.5	140.3	120.9	129.2	122.0	65.4	-	27.6
**10d** ^h^	122.8	122.6	140.0	118.6	152.8	77.5	138.6	123.6	126.2	140.3	120.6	129.1	119.6	65.3	-	27.7
**10e**	123.0	121.7	129.7	118.2	152.9	77.6	139.0	123.6	126.6	140.4	120.9	129.0	122.4	65.2	-	27.7
**10f** ^i^	120.8	142.1	127.7	117.9	150.9	77.4	139.3	123.7	126.5	140.3	120.9	129.4	121.9	65.5	-	27.7
**10g** ^j^	122.8	120.1	151.3	115.8	152.9	77.6	138.7	123.6	126.2	140.3	120.7	129.2	119.8	65.4	-	27.8
**10h** ^k^	122.8	136.1	129.7	117.9	150.8	77.5	139.2	123.6	126.5	140.3	120.9	129.3	121.9	65.4	-	27.7

^a^ Ethyl signals at 27.6 and 10.2 ppm. ^b^ Ethyl signals at 27.6 and 10.2 ppm. ^c^ Ethyl signals at 30.6 and 8.2 ppm. ^d^ *n-*Butyl signals at 38.4, 26.1, 23.2 and 14.2 ppm. ^e^ Methoxy signal at 55.5 ppm. ^f^ Methoxy signal at 55.8 ppm. ^g^ Methyl signal at 21.1 ppm. ^h^ Methyl signal at 21.5 ppm. ^I^ Isopropyl signals at 33.9 and 24.4 ppm. ^j^ Isopropyl signals at 34.1 and 23.9 ppm. ^k^ *n*-Pentyl signals at 35.7, 31.7, 31.6, 22.7 and 14.2 pp.

## Supplementary Materials

The following are available online: ^1^H and ^13^C NMR spectra of all intermediates and all pulchrol analogues prepared and assayed in this investigation.
