# Supplementary material for: SARs for the Antiparasitic Plant Metabolite Pulchrol. Part 2: B- and C-Ring Substituents
Source: Molecules. 2020 Oct 1;25(19):4510. doi: 10.3390/molecules25194510 (PMC7582507; doi:10.3390/molecules25194510)
Supplement: Supplementary file 1 [file molecules-25-04510-s001.pdf]

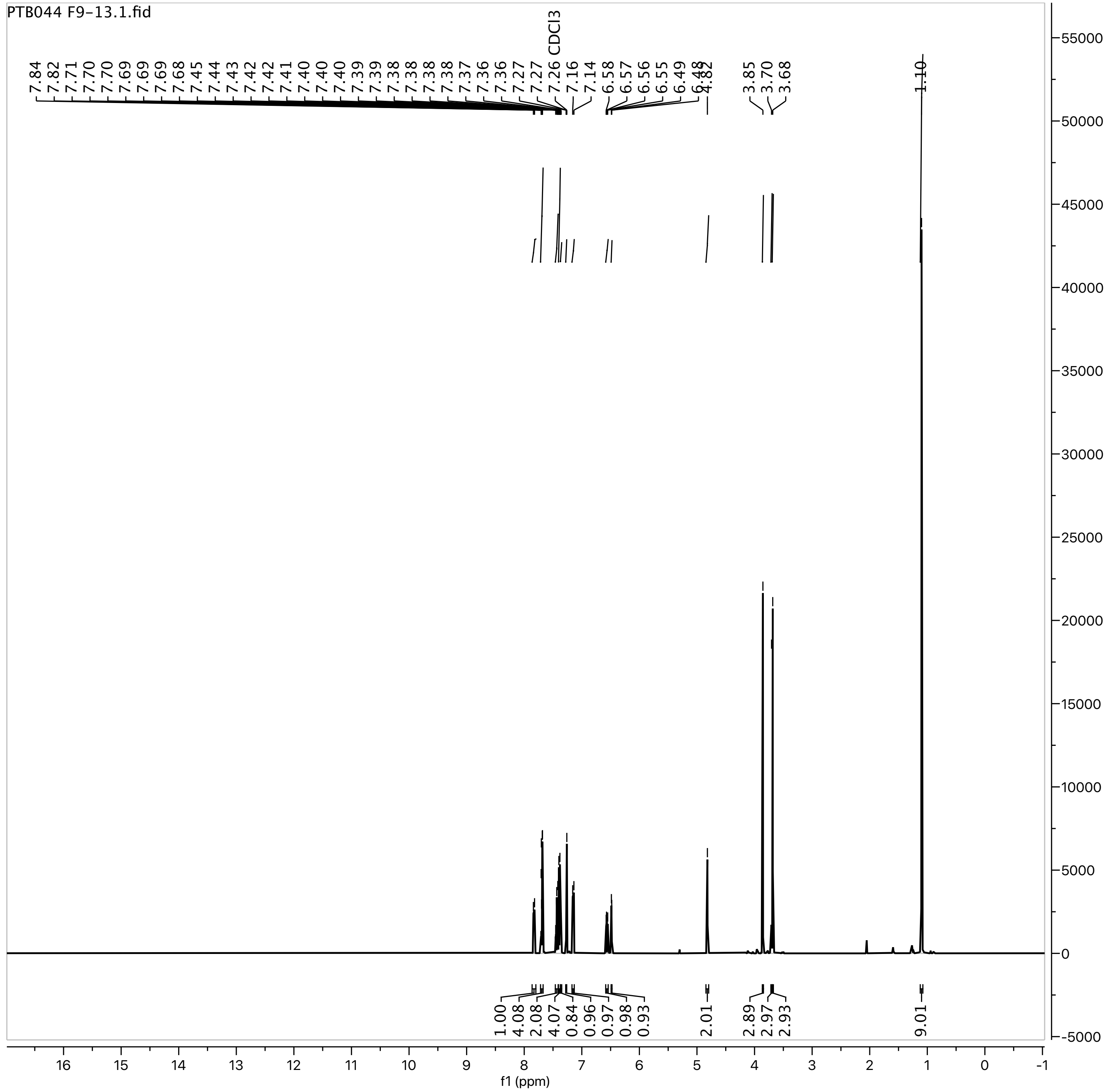

	Parameter	Value
1	Solvent	CDCl3
2	Experiment	1D
3	Spectrometer Frequency	400.13
4	Nucleus	1H

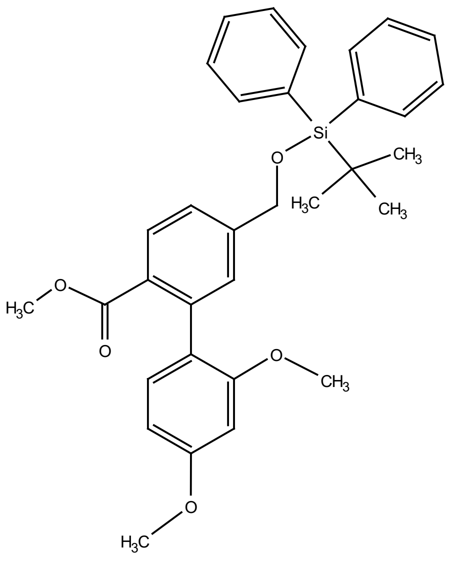

^{1}H NMR (400 MHz, $CDCl_3$) δ 7.83 (d, *J* = 8.0 Hz, 1H), 7.72 – 7.67 (m, 4H), 7.46 – 7.41 (m, 2H), 7.41 – 7.37 (m, 4H), 7.36 (t, *J* = 1.5 Hz, 1H), 7.27 (d, *J* = 2.3 Hz, 1H), 7.15 (d, *J* = 8.4 Hz, 1H), 6.56 (dd, *J* = 8.3, 2.4 Hz, 1H), 6.48 (d, *J* = 2.4 Hz, 1H), 4.82 (s, 2H), 3.85 (s, 3H), 3.70 (s, 3H), 3.68 (s, 3H), 1.10 (s, 9H).

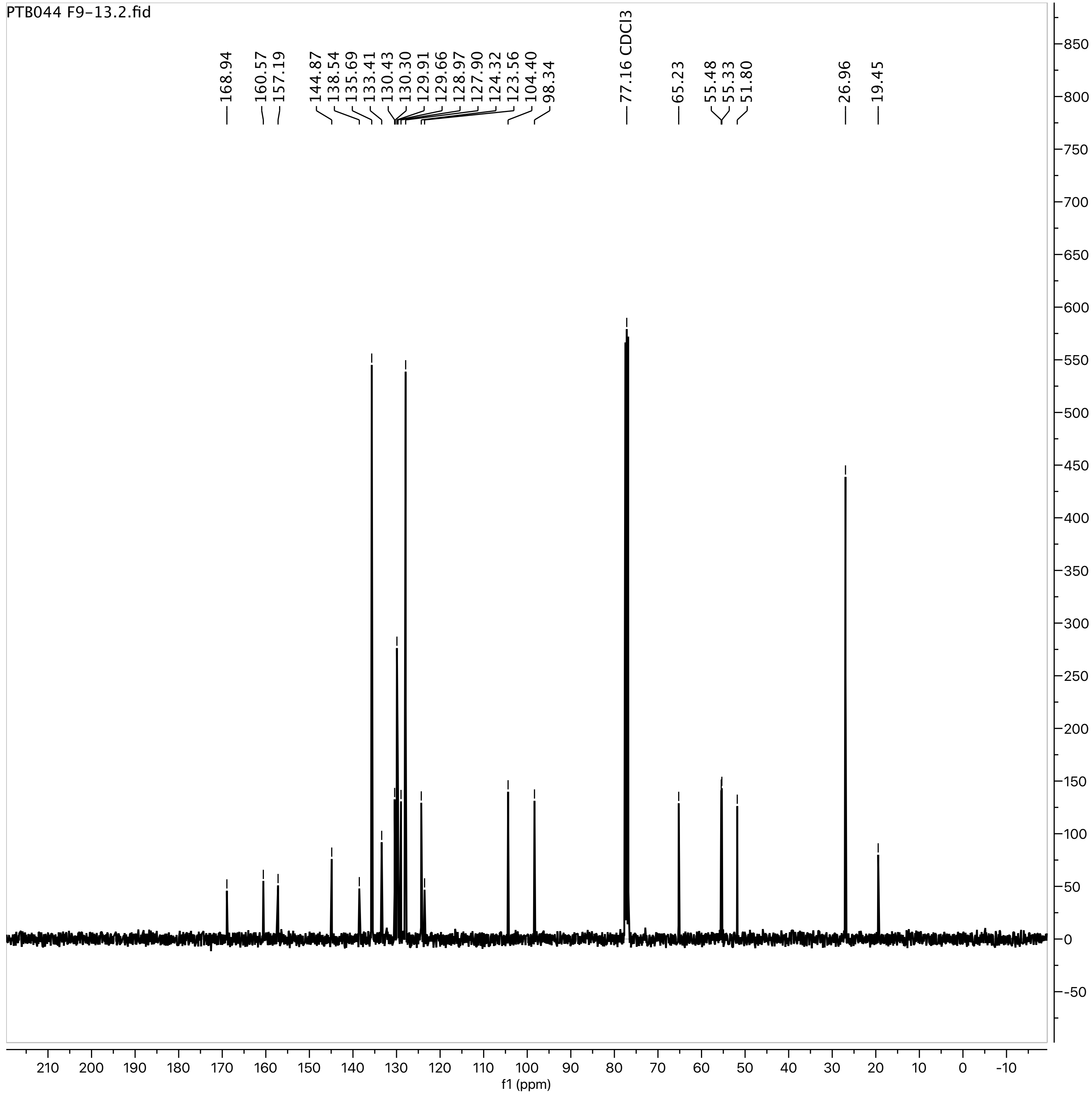

	Parameter	Value
1	Solvent	CDCl3
2	Experiment	1D
3	Spectrometer Frequency	100.62
4	Nucleus	13C

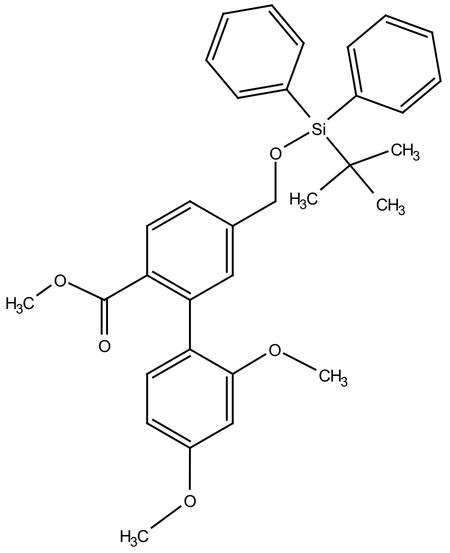

^{13}C NMR (101 MHz, $CDCl_3$) δ 168.94, 160.57, 157.19, 144.87, 138.54, 135.69, 133.41, 130.43, 130.30, 129.91, 129.66, 128.97, 127.90, 124.32, 123.56, 104.40, 98.34, 65.23, 55.48, 55.33, 51.80, 26.96, 19.45.

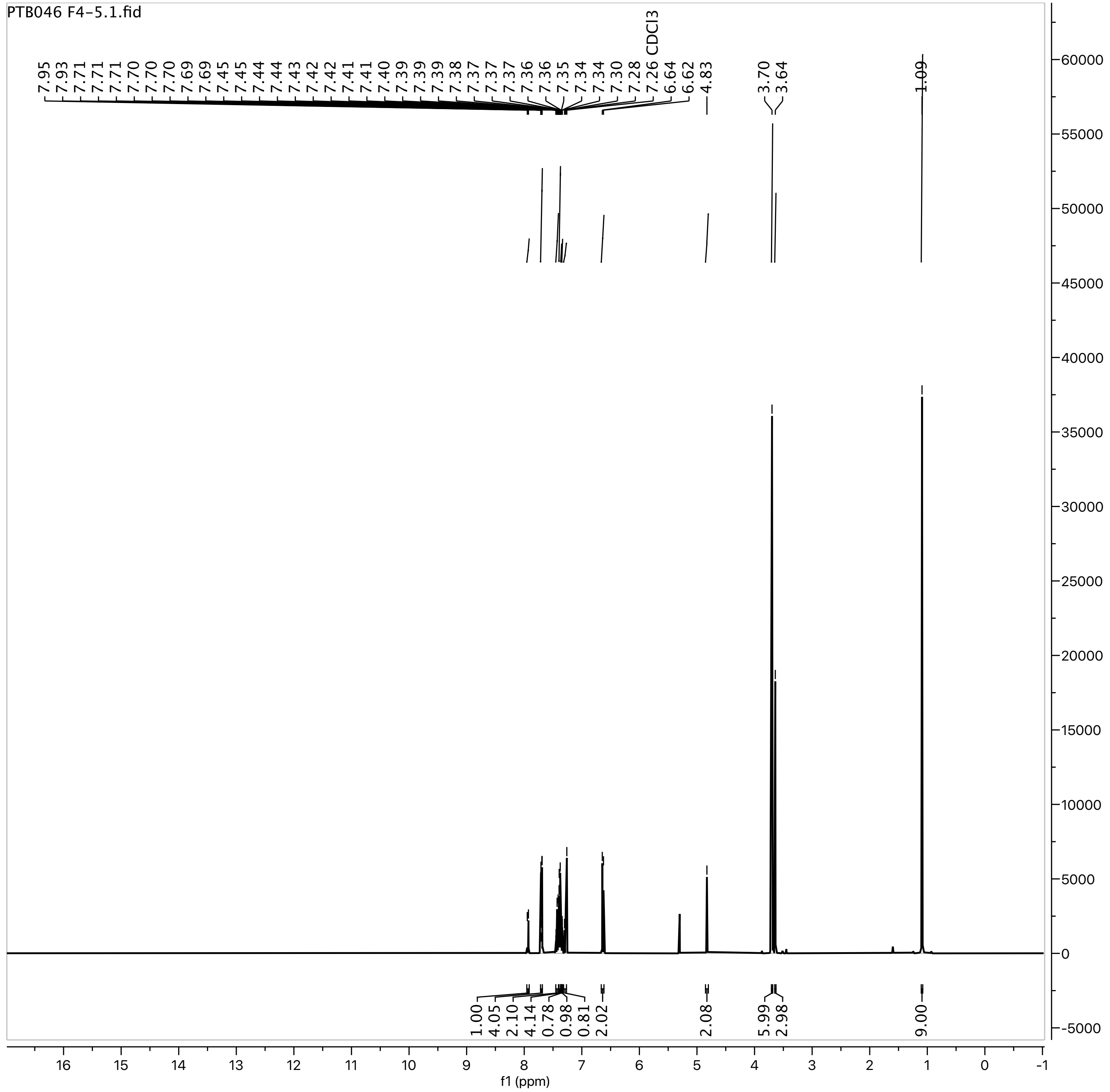

	Parameter	Value
1	Solvent	CDCl3
2	Experiment	1D
3	Spectrometer Frequency	400.13
4	Nucleus	1H

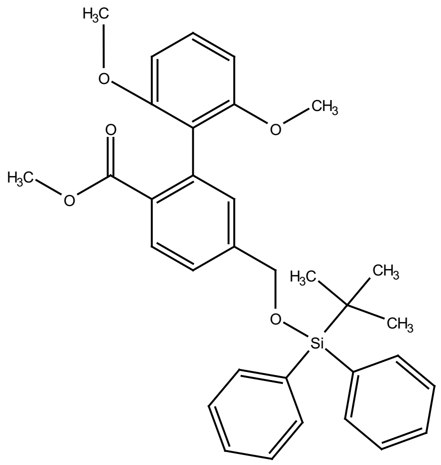

^{1}H NMR (400 MHz, $CDCl_3$) δ 7.94 (d, J = 8.0 Hz, 1H), 7.72 – 7.68 (m, 4H), 7.45 – 7.41 (m, 2H), 7.40 – 7.37 (m, 4H), 7.36 (t, J = 1.6 Hz, 1H), 7.34 (d, J = 1.1 Hz, 1H), 7.29 (d, J = 8.4 Hz, 1H), 6.63 (d, J = 8.4 Hz, 2H), 4.83 (s, 2H), 3.70 (s, 6H), 3.64 (s, 3H), 1.09 (s, 9H).

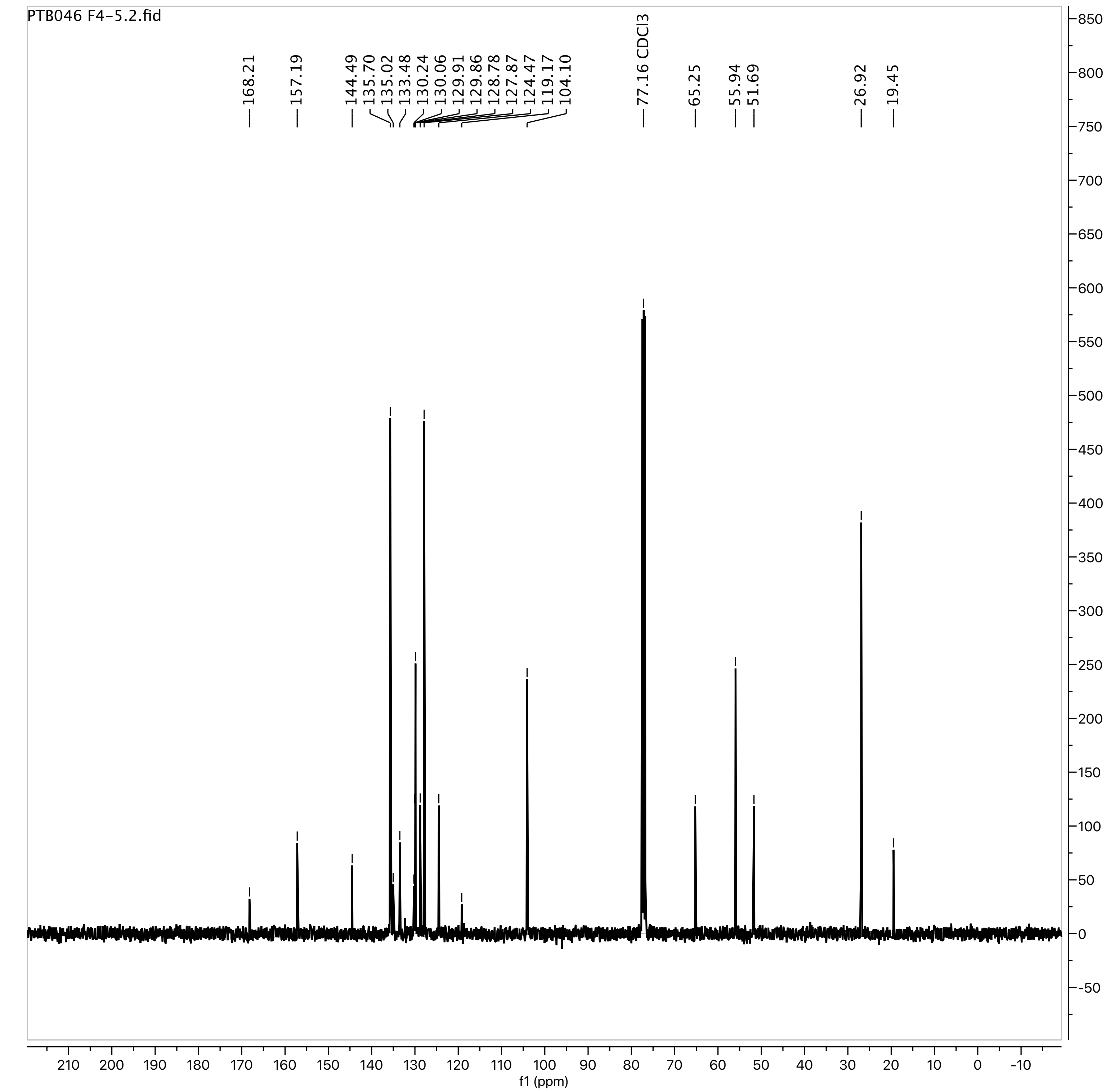

	Parameter	Value
1	Solvent	CDCl3
2	Experiment	1D
3	Spectrometer Frequency	100.62
4	Nucleus	13C

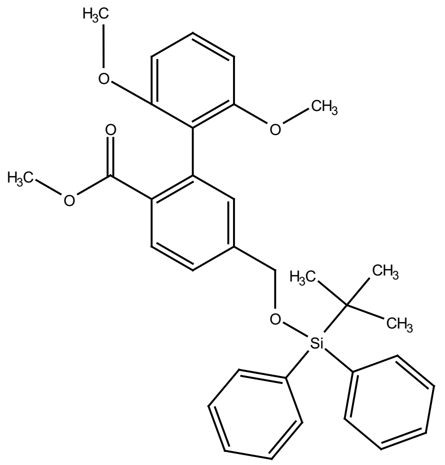

^{13}C NMR (101 MHz, $CDCl_3$) δ 168.21, 157.19, 144.49, 135.70, 135.02, 133.48, 130.24, 130.06, 129.91, 129.86, 128.78, 127.87, 124.47, 119.17, 104.10, 65.25, 55.94, 51.69, 26.92, 19.45.

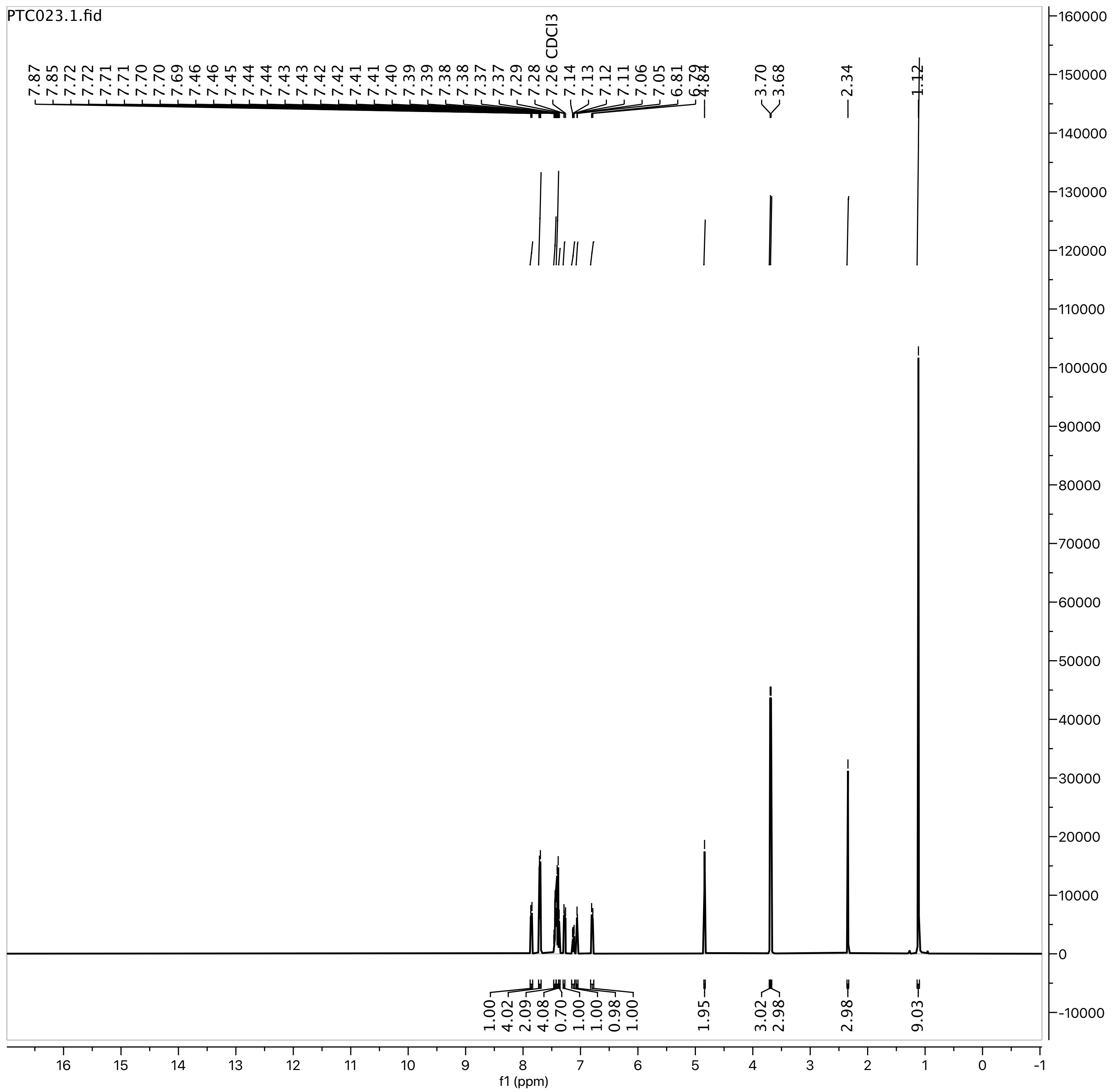

	Parameter	Value
1	Solvent	CDCl3
2	Experiment	1D
3	Spectrometer Frequency	400.13
4	Nucleus	1H

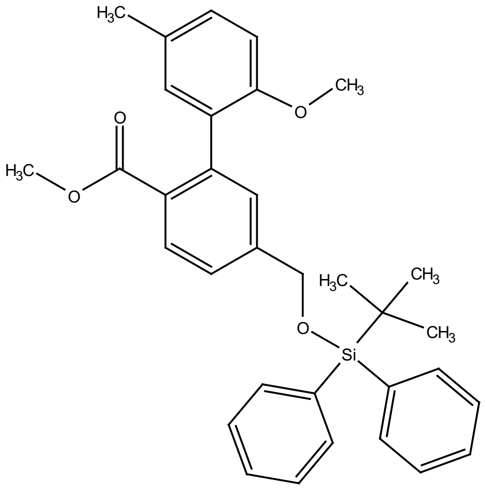

^{1}H NMR (400 MHz, $CDCl_3$) δ 7.86 (d, J = 8.0 Hz, 1H), 7.73 – 7.69 (m, 4H), 7.45 (td, J = 5.6, 2.2 Hz, 2H), 7.40 (dd, J = 7.8, 2.0 Hz, 4H), 7.37 (d, J = 1.7 Hz, 1H), 7.29 (d, J = 1.8 Hz, 1H), 7.12 (dd, J = 8.3, 2.4 Hz, 1H), 7.06 (d, J = 2.5 Hz, 1H), 6.80 (d, J = 8.4 Hz, 1H), 4.84 (s, 2H), 3.70 (s, 3H), 3.68 (s, 3H), 2.34 (s, 3H), 1.12 (s, 9H).

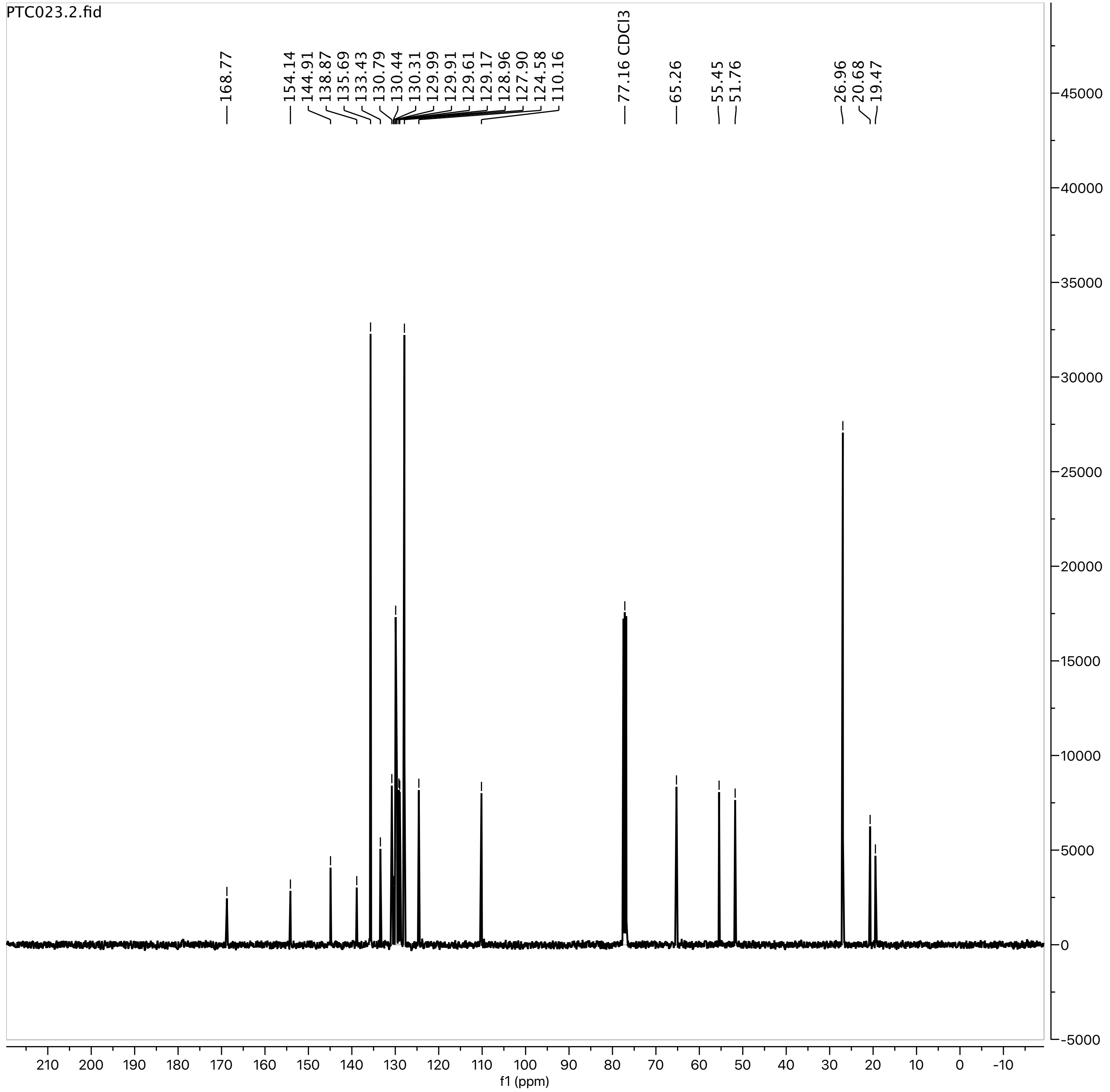

	Parameter	Value
1	Solvent	CDCl3
2	Experiment	1D
3	Spectrometer Frequency	100.62
4	Nucleus	13C

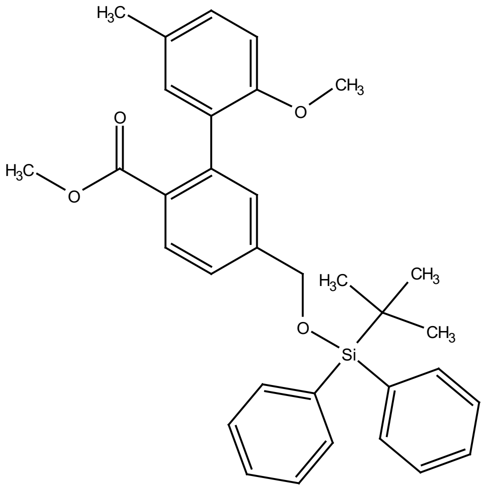

^{13}C NMR (101 MHz, $CDCl_3$) δ 168.77, 154.14, 144.91, 138.87, 135.69, 133.43, 130.79, 130.44, 130.31, 129.99, 129.91, 129.61, 129.17, 128.96, 127.90, 124.58, 110.16, 65.26, 55.45, 51.76, 26.96, 20.68, 19.47.

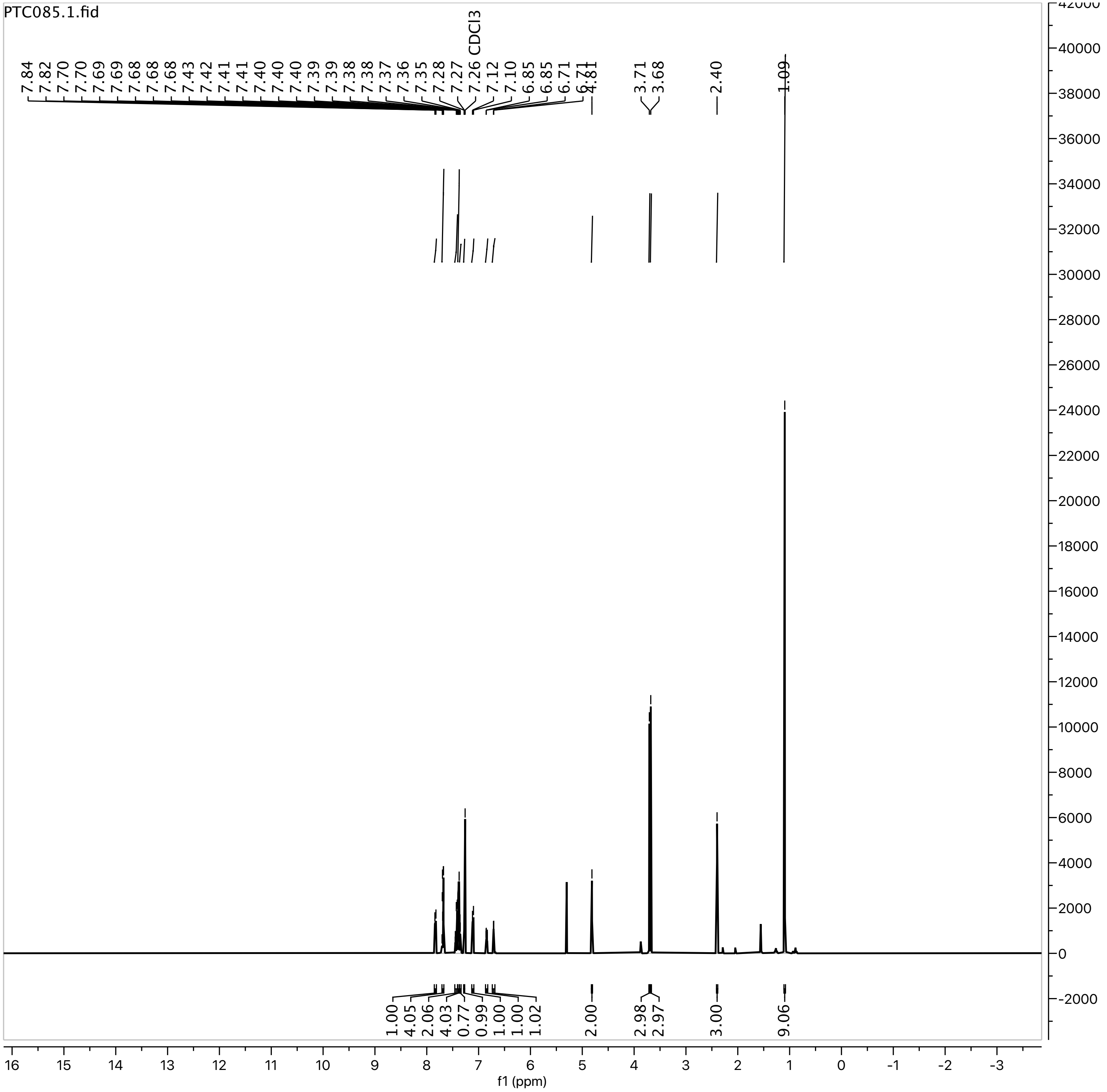

	Parameter	Value
1	Solvent	CDCl3
2	Experiment	1D
3	Spectrometer Frequency	400.13
4	Nucleus	1H

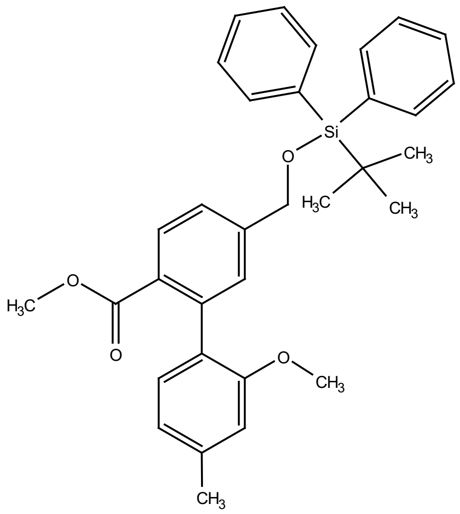

^{1}H NMR (400 MHz, $CDCl_3$) δ 7.83 (d, J = 7.9 Hz, 1H), 7.71 – 7.67 (m, 4H), 7.46 – 7.41 (m, 2H), 7.40 – 7.37 (m, 4H), 7.36 (t, J = 1.6 Hz, 1H), 7.28 (d, J = 2.2 Hz, 1H), 7.11 (d, J = 7.6 Hz, 1H), 6.84 (ddd, J = 7.6, 1.6, 0.7 Hz, 1H), 6.71 (d, J = 2.1 Hz, 1H), 4.81 (s, 2H), 3.71 (s, 3H), 3.68 (s, 3H), 2.40 (s, 3H), 1.09 (s, 9H).

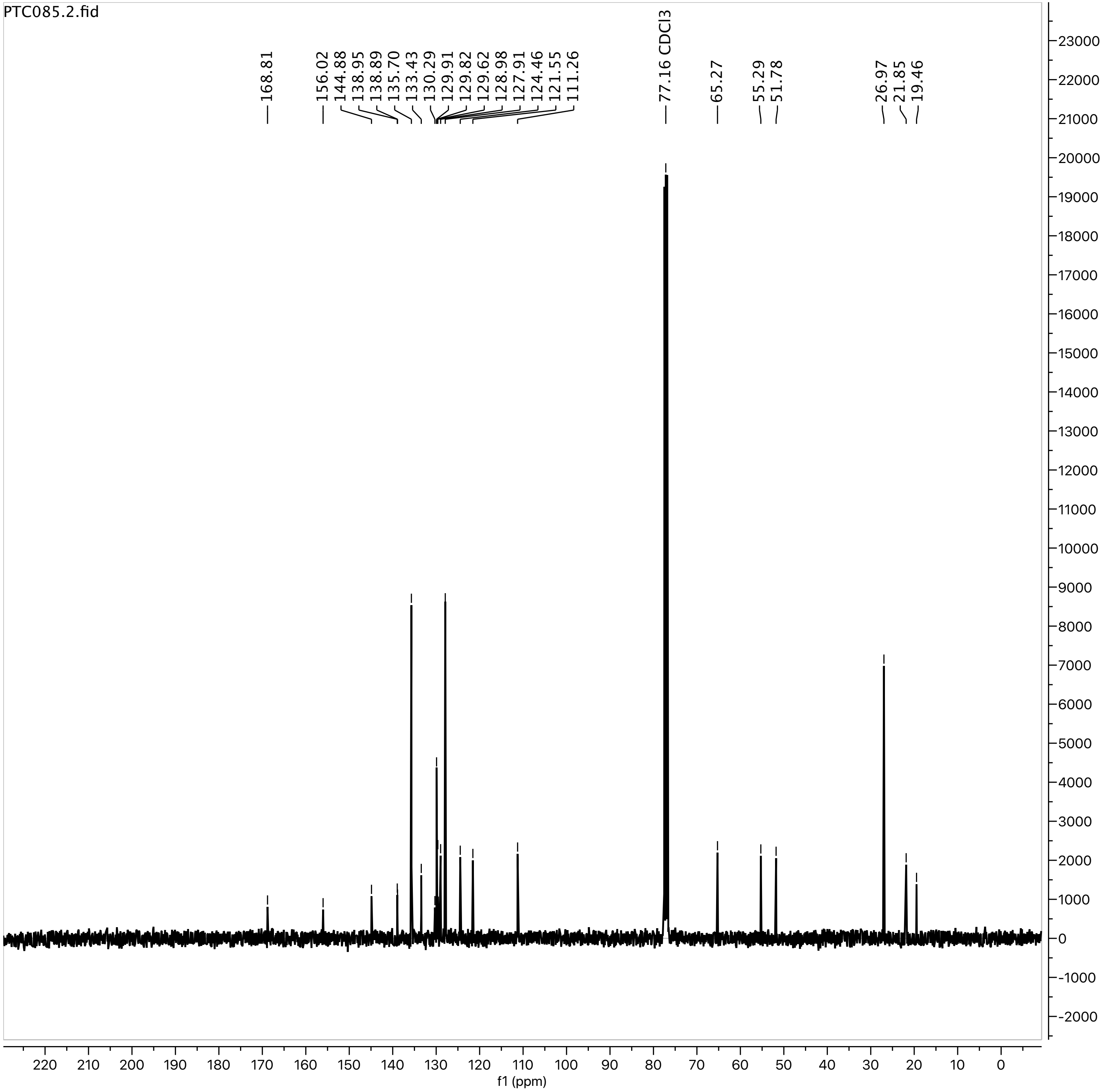

	Parameter	Value
1	Solvent	CDCl3
2	Experiment	1D
3	Spectrometer Frequency	100.62
4	Nucleus	13C

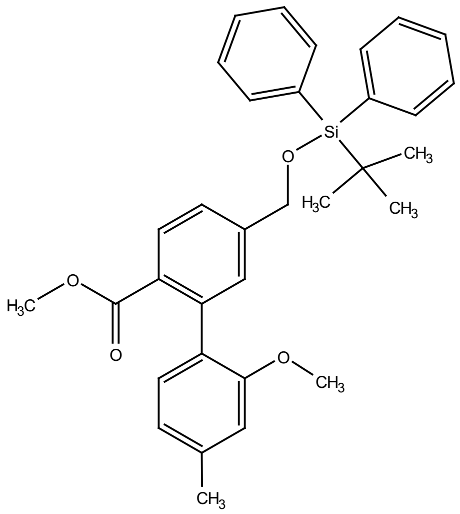

^{13}C NMR (101 MHz, $CDCl_3$) δ 168.81, 156.02, 144.88, 138.95, 138.89, 135.70, 133.43, 130.29, 129.91, 129.82, 129.62, 128.98, 127.91, 124.46, 121.55, 111.26, 65.27, 55.29, 51.78, 26.97, 21.85, 19.46.

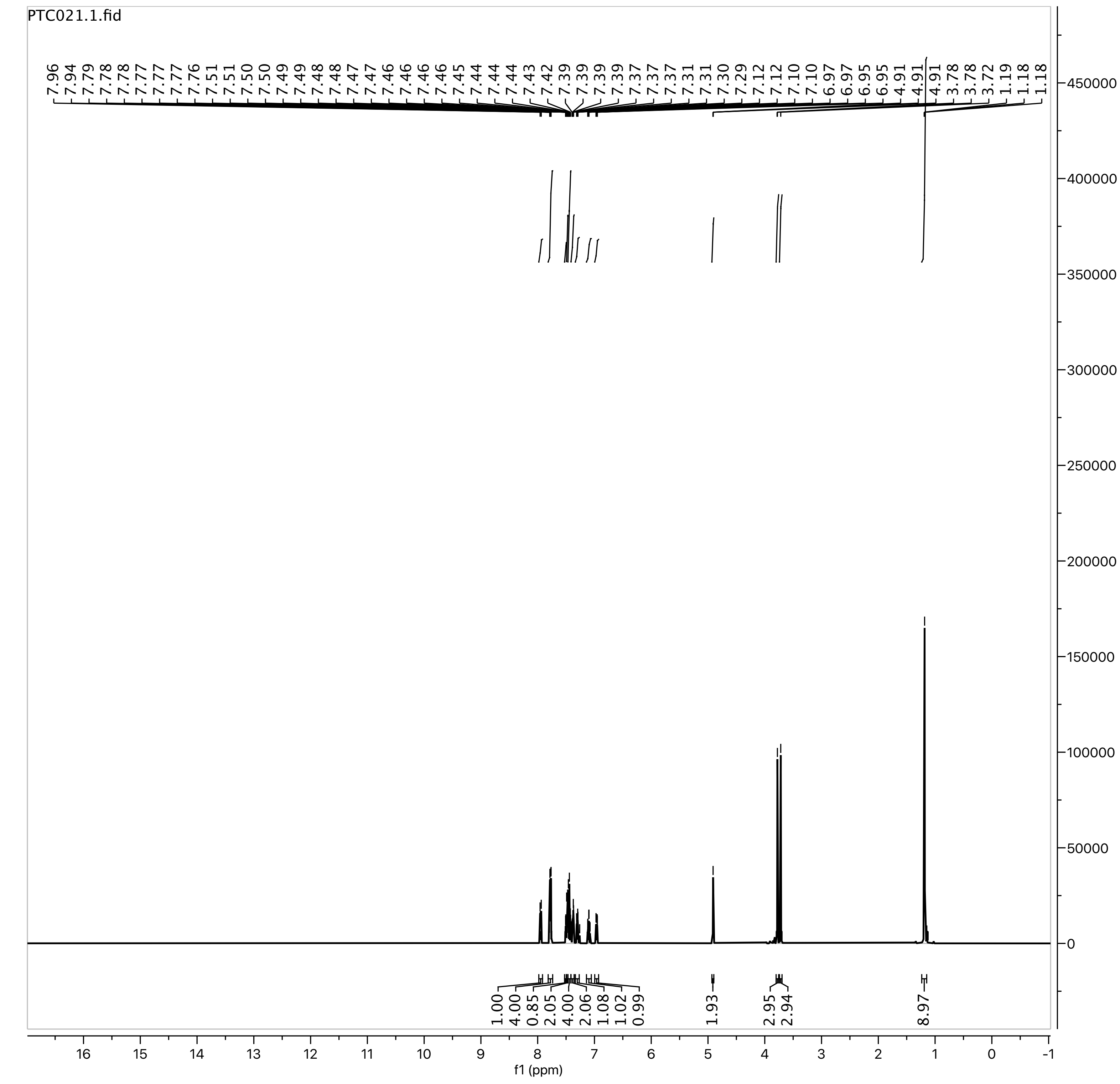

	Parameter	Value
1	Solvent	CDCl3
2	Experiment	1D
3	Spectrometer Frequency	400.13
4	Nucleus	1H

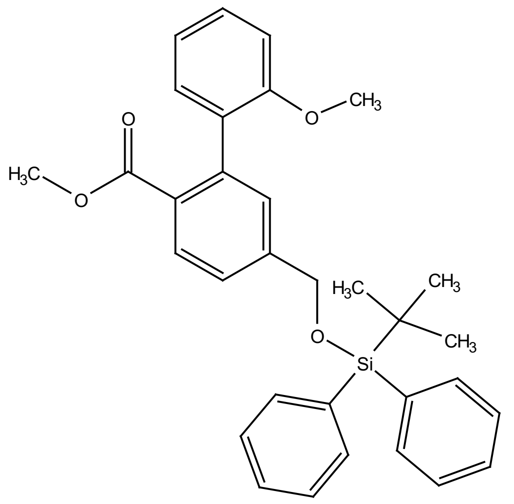

^{1}H NMR (400 MHz, $CDCl_3$) δ 7.95 (d, J = 8.0 Hz, 1H), 7.82 – 7.74 (m, 4H), 7.51 (dq, J = 2.1, 1.3 Hz, 1H), 7.49 – 7.47 (m, 2H), 7.44 (tt, J = 7.8, 1.5 Hz, 4H), 7.41 – 7.36 (m, 2H), 7.30 (dd, J = 7.5, 1.8 Hz, 1H), 7.10 (td, J = 7.5, 1.1 Hz, 1H), 6.96 (dd, J = 8.3, 1.1 Hz, 1H), 4.91 (d, J = 0.9 Hz, 2H), 3.78 (s, 3H), 3.72 (s, 3H), 1.18 (s, 9H).

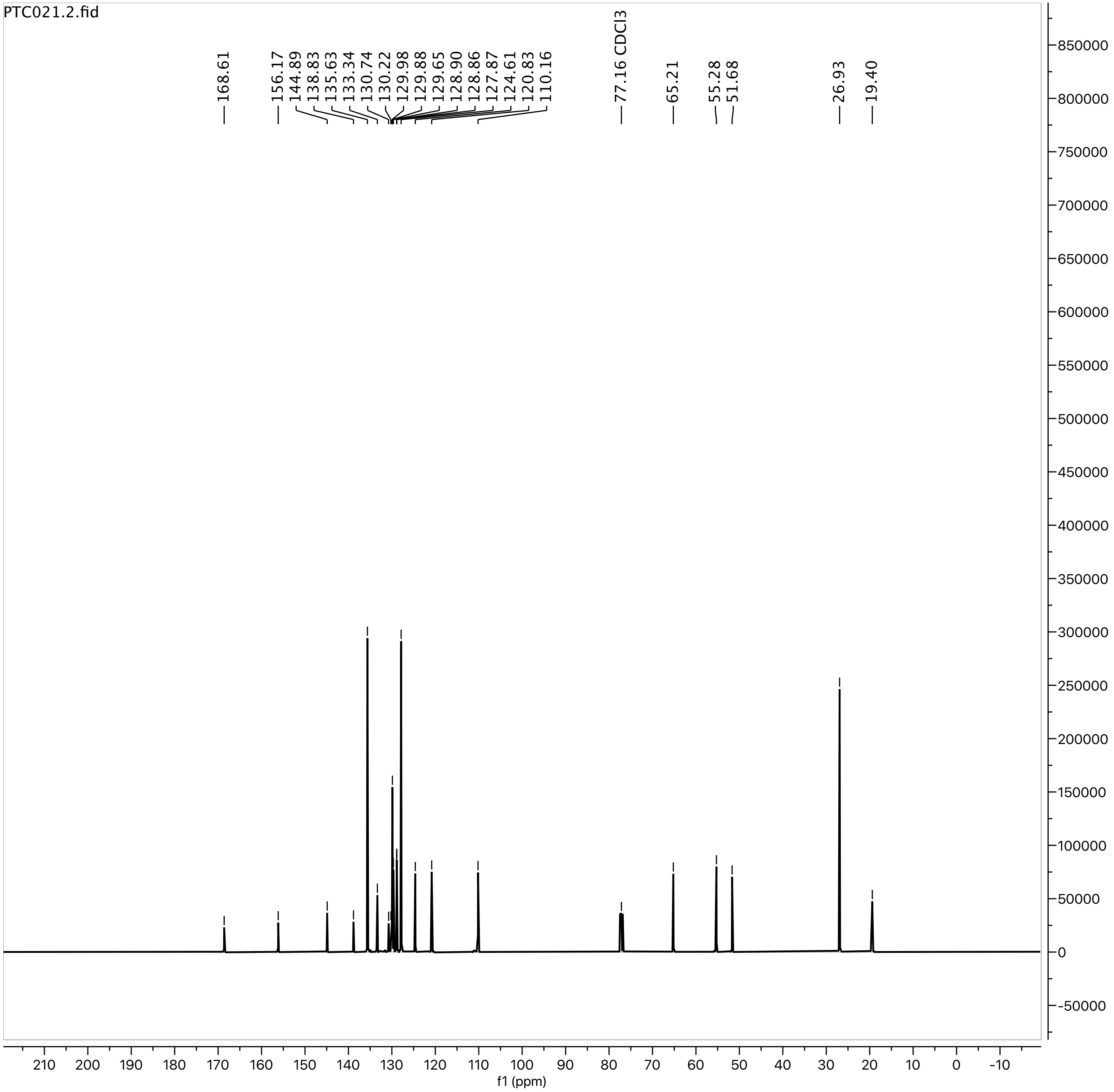

	Parameter	Value
1	Solvent	CDCl3
2	Experiment	1D
3	Spectrometer Frequency	100.62
4	Nucleus	13C

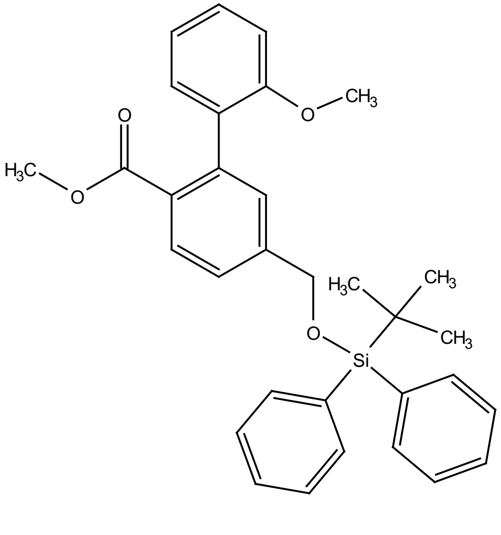

^{13}C NMR (101 MHz, $CDCl_3$) δ 168.61, 156.17, 144.89, 138.83, 135.63, 133.34, 130.74, 130.22, 129.98, 129.88, 129.65, 128.88 (d, J = 3.7 Hz), 127.87, 124.61, 120.83, 110.16, 65.21, 55.28, 51.68, 26.93, 19.40.

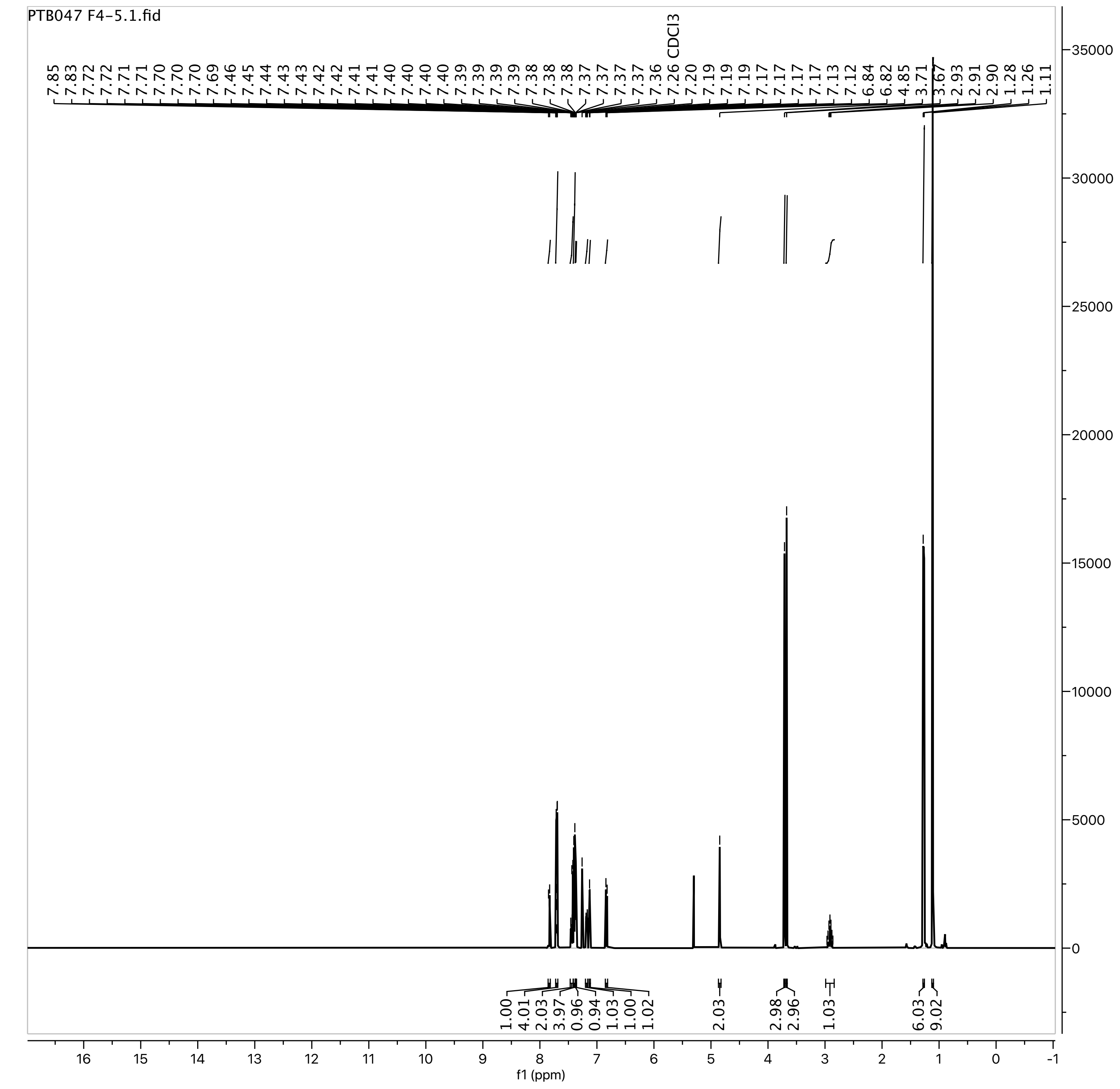

	Parameter	Value
1	Solvent	CDCl3
2	Experiment	1D
3	Spectrometer Frequency	400.13
4	Nucleus	1H

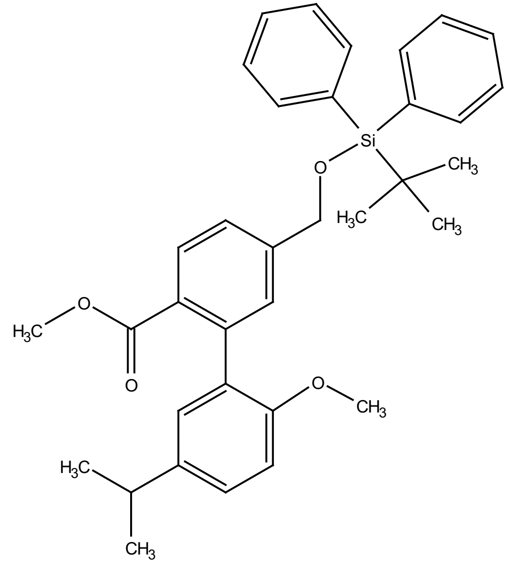

^{1}H NMR (400 MHz, $CDCl_3$) δ 7.84 (d, *J* = 7.9 Hz, 1H), 7.72 – 7.69 (m, 4H), 7.47 – 7.42 (m, 2H), 7.41 – 7.38 (m, 4H), 7.38 – 7.37 (m, 1H), 7.37 (d, *J* = 1.0 Hz, 1H), 7.18 (ddd, *J* = 8.4, 2.4, 0.7 Hz, 1H), 7.13 (d, *J* = 2.4 Hz, 1H), 6.83 (d, *J* = 8.4 Hz, 1H), 4.85 (s, 2H), 3.71 (s, 3H), 3.67 (s, 3H), 2.91 (hept, *J* = 6.9 Hz, 1H), 1.27 (d, *J* = 6.9 Hz, 6H), 1.11 (s, 9H).

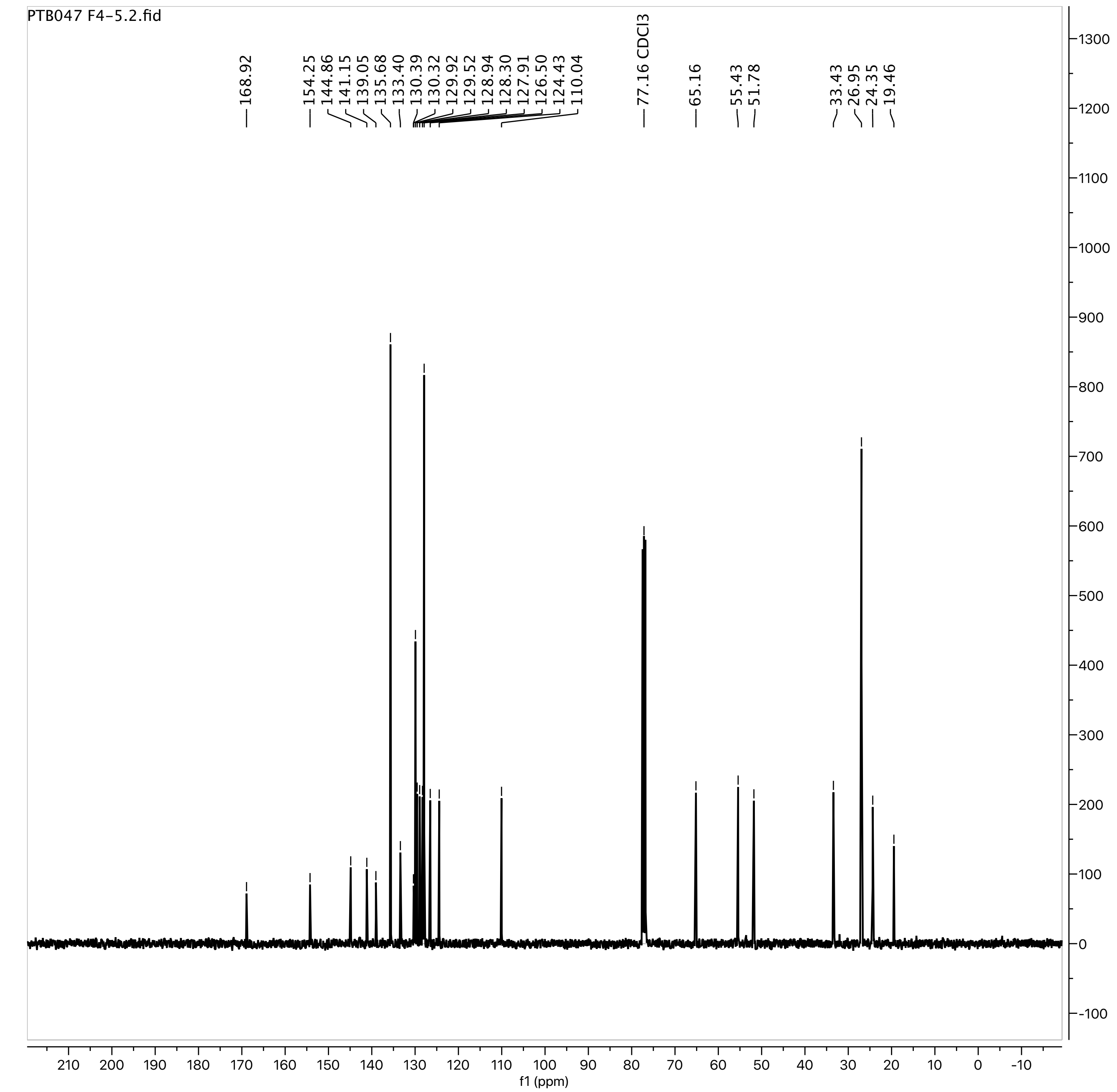

	Parameter	Value
1	Solvent	CDCl3
2	Experiment	1D
3	Spectrometer Frequency	100.62
4	Nucleus	13C

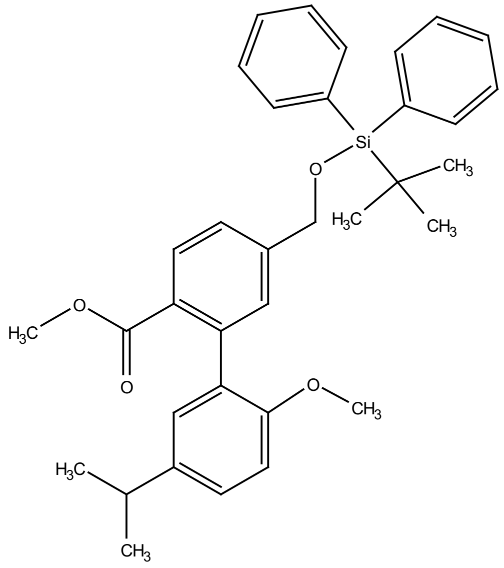

^{13}C NMR (101 MHz, $CDCl_3$) δ 168.92, 154.25, 144.86, 141.15, 139.05, 135.68, 133.40, 130.39, 130.32, 129.92, 129.52, 128.94, 128.30, 127.91, 126.50, 124.43, 110.04, 65.16, 55.43, 51.78, 33.43, 26.95, 24.35, 19.46.

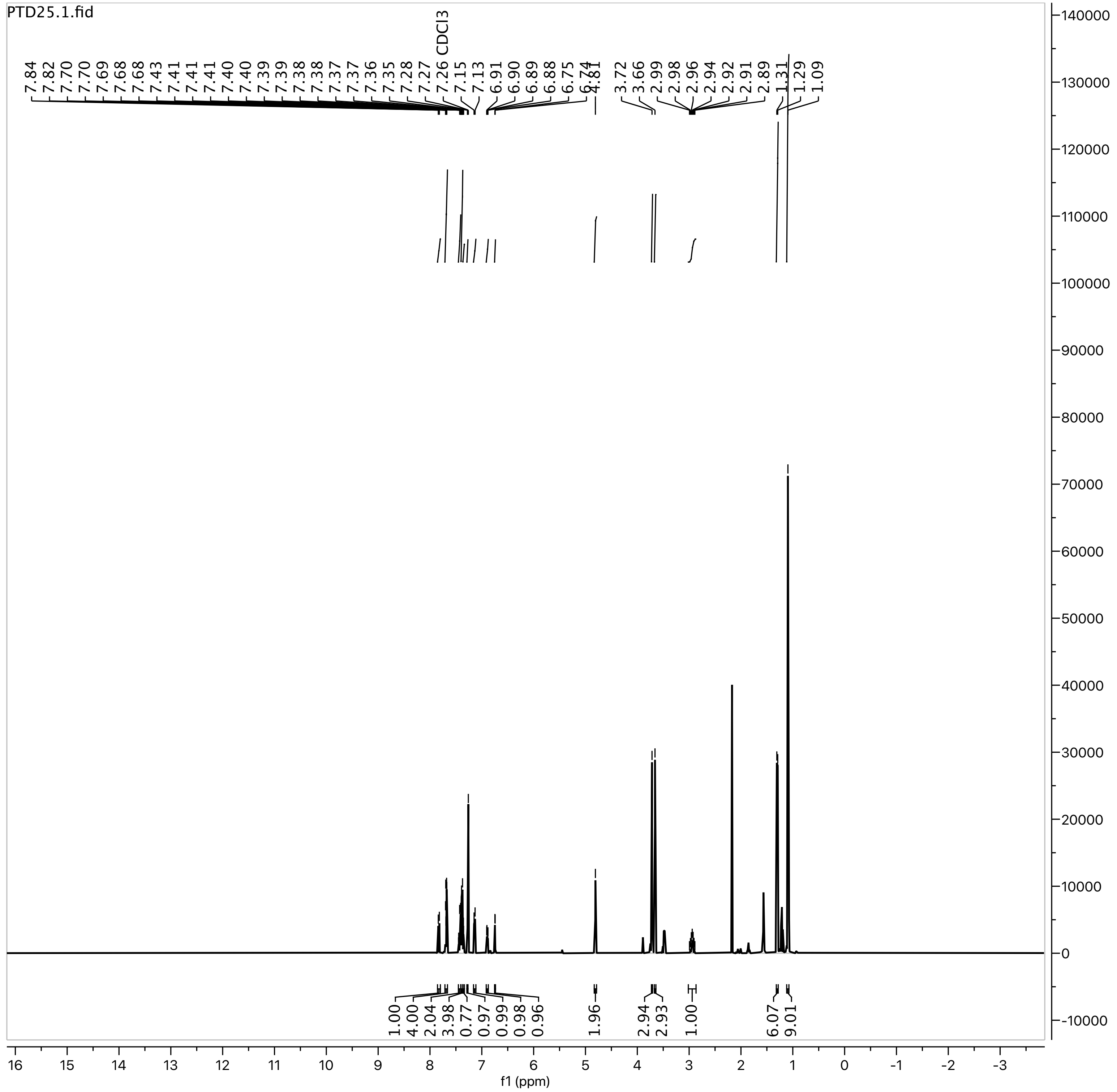

	Parameter	Value
1	Solvent	CDCl3
2	Experiment	1D
3	Spectrometer Frequency	400.13
4	Nucleus	1H

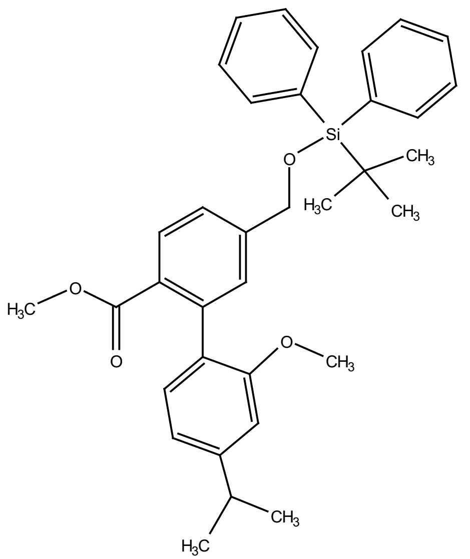

^{1}H NMR (400 MHz, $CDCl_3$) δ 7.83 (d, J = 8.1 Hz, 1H), 7.71 – 7.66 (m, 4H), 7.45 – 7.41 (m, 2H), 7.40 – 7.37 (m, 4H), 7.35 (d, J = 1.7 Hz, 1H), 7.27 (d, J = 1.8 Hz, 1H), 7.14 (d, J = 7.7 Hz, 1H), 6.89 (dd, J = 7.7, 1.7 Hz, 1H), 6.75 (d, J = 1.7 Hz, 1H), 4.81 (s, 2H), 3.72 (s, 3H), 3.66 (s, 3H), 2.94 (hept, J = 7.0 Hz, 1H), 1.30 (d, J = 7.0 Hz, 6H), 1.09 (s, 9H).

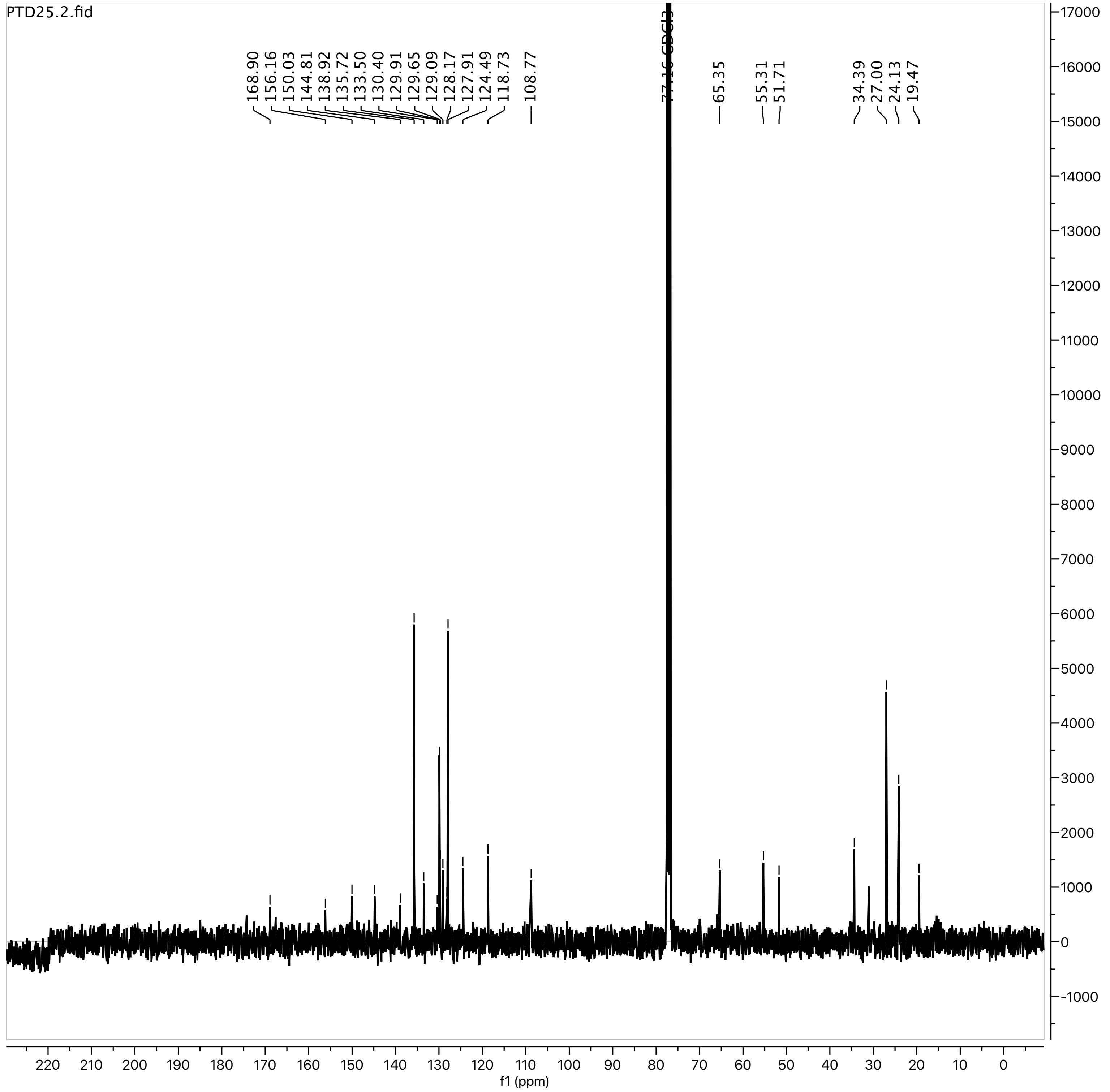

	Parameter	Value
1	Solvent	CDCl3
2	Experiment	1D
3	Spectrometer Frequency	100.62
4	Nucleus	13C

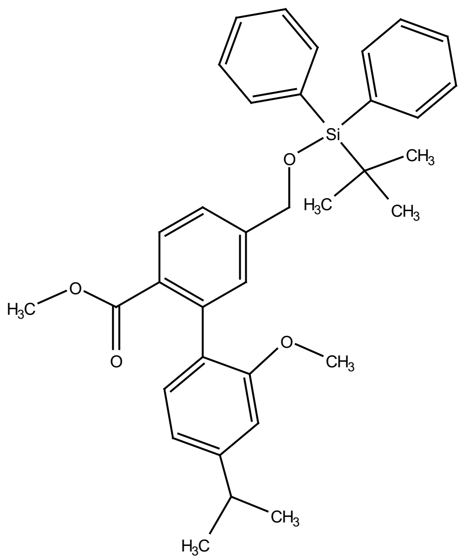

^{13}C NMR (101 MHz, $CDCl_3$) δ 168.90, 156.16, 150.03, 144.81, 138.92, 135.72, 133.50, 130.40, 129.91, 129.65, 129.09, 128.17, 127.91, 124.49, 118.73, 108.77, 65.35, 55.31, 51.71, 34.39, 27.00, 24.13, 19.47.

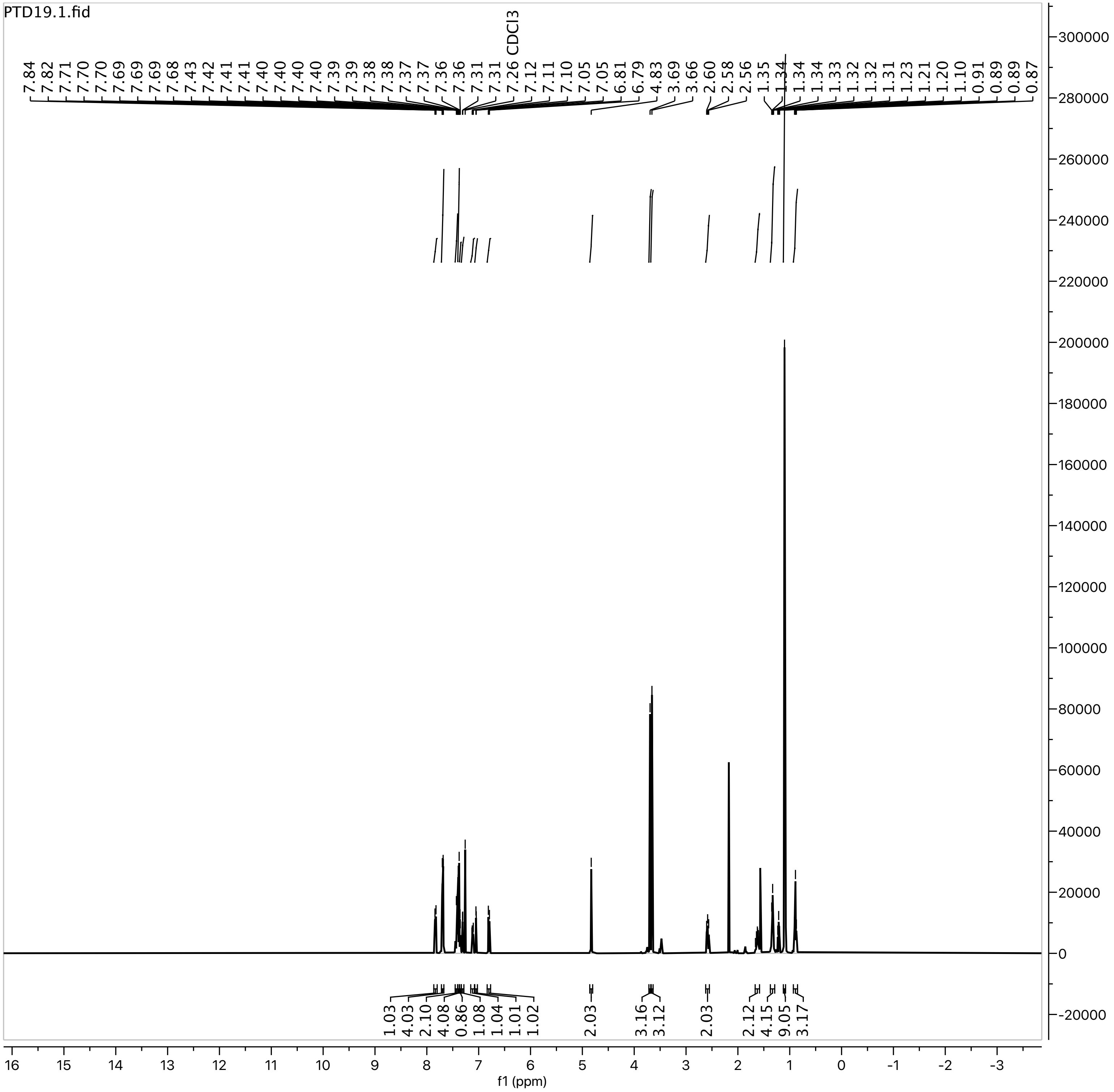

	Parameter	Value
1	Solvent	CDCl3
2	Experiment	1D
3	Spectrometer Frequency	400.13
4	Nucleus	1H

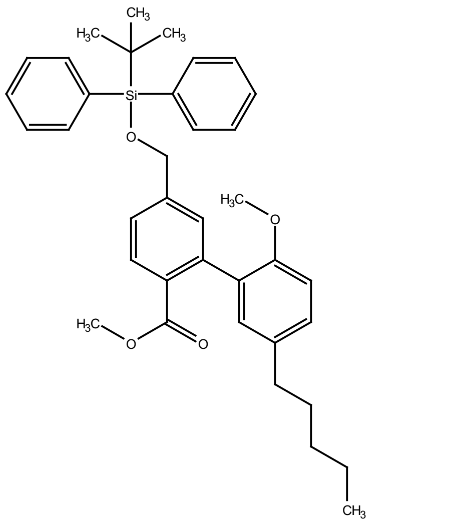

^{1}H NMR (400 MHz, $CDCl_3$) δ 7.83 (d, J = 8.0 Hz, 1H), 7.72 – 7.67 (m, 4H), 7.45 – 7.41 (m, 2H), 7.40 – 7.37 (m, 4H), 7.36 (t, J = 1.6 Hz, 1H), 7.31 (d, J = 1.7 Hz, 1H), 7.12 (dd, J = 8.3, 2.3 Hz, 1H), 7.05 (d, J = 2.3 Hz, 1H), 6.80 (d, J = 8.3 Hz, 1H), 4.83 (s, 2H), 3.69 (s, 3H), 3.66 (s, 3H), 2.62 – 2.55 (m, 2H), 1.67 – 1.58 (m, 2H), 1.33 (ddd, J = 7.1, 4.0, 2.9 Hz, 4H), 1.10 (s, 9H), 0.93 – 0.85 (m, 3H).

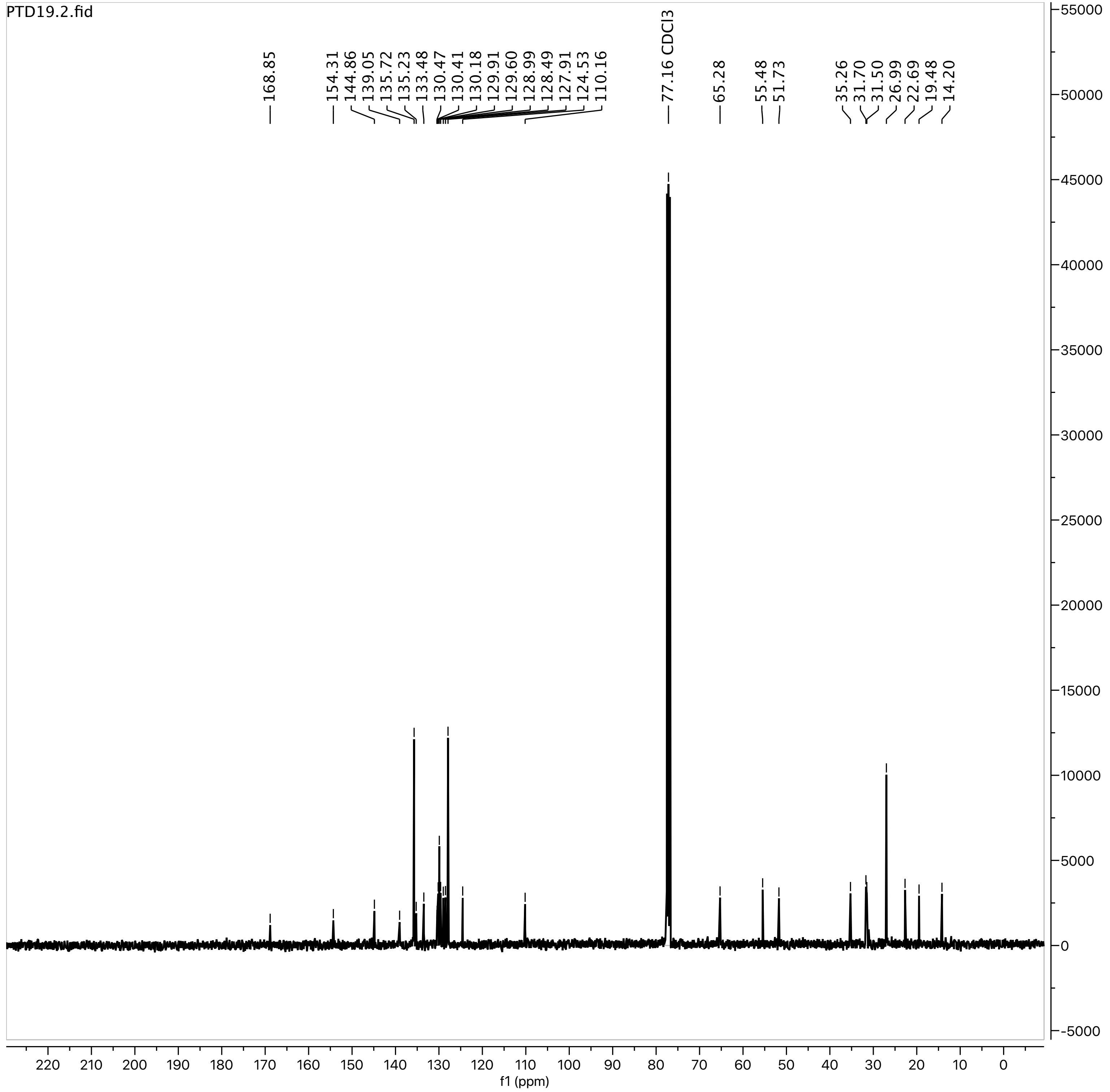

	Parameter	Value
1	Solvent	CDCl3
2	Experiment	1D
3	Spectrometer Frequency	100.62
4	Nucleus	13C

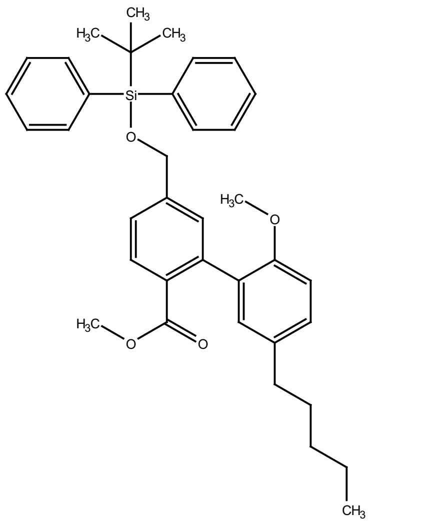

^{13}C NMR (101 MHz, $CDCl_3$) δ 168.85, 154.31, 144.86, 139.05, 135.72, 135.23, 133.48, 130.47, 130.41, 130.18, 129.91, 129.60, 128.99, 128.49, 127.91, 124.53, 110.16, 65.28, 55.48, 51.73, 35.26, 31.70, 31.50, 26.99, 22.69, 19.48, 14.20.

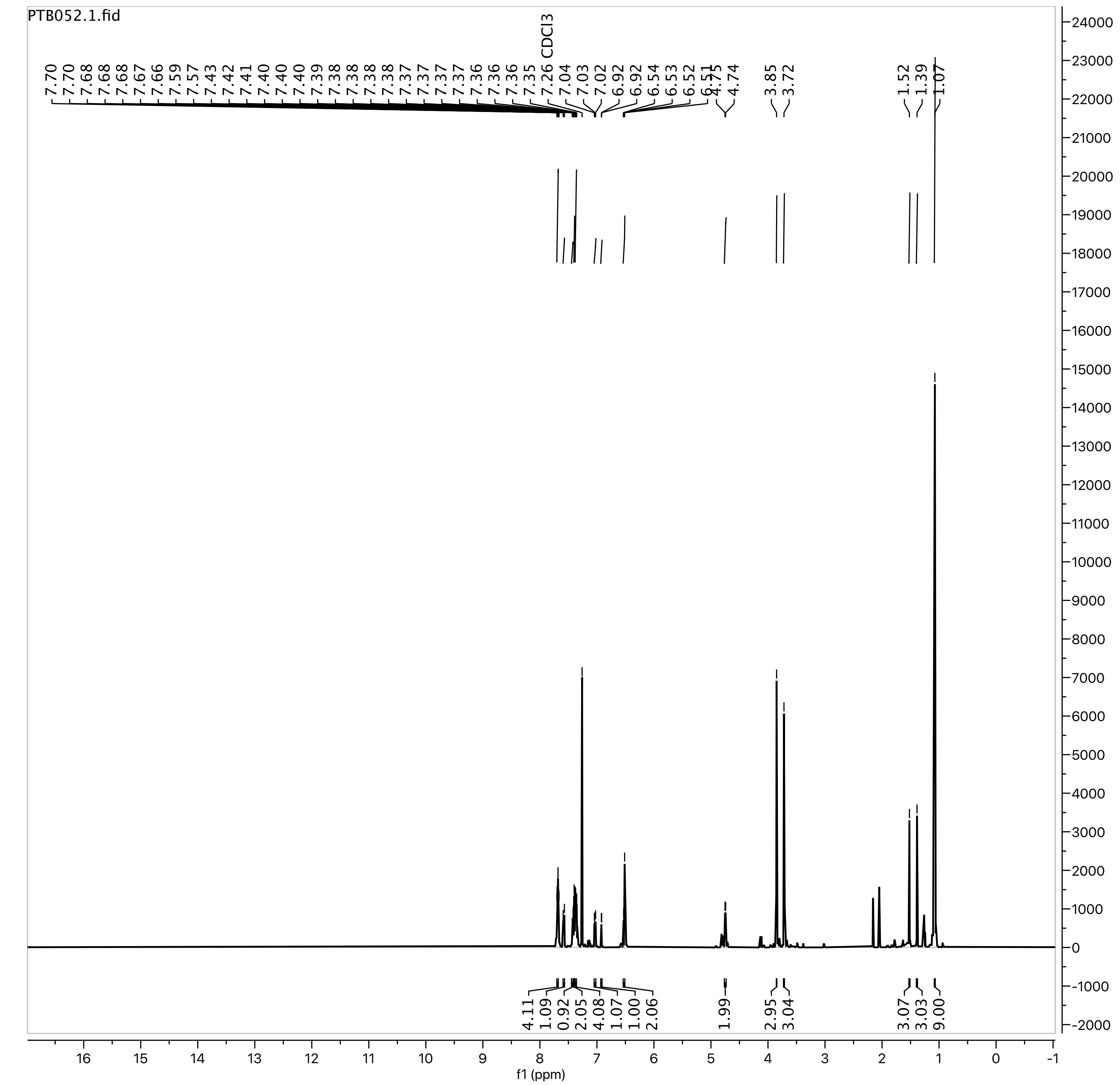

	Parameter	Value
1	Solvent	CDCl3
2	Experiment	1D
3	Spectrometer Frequency	400.13
4	Nucleus	1H

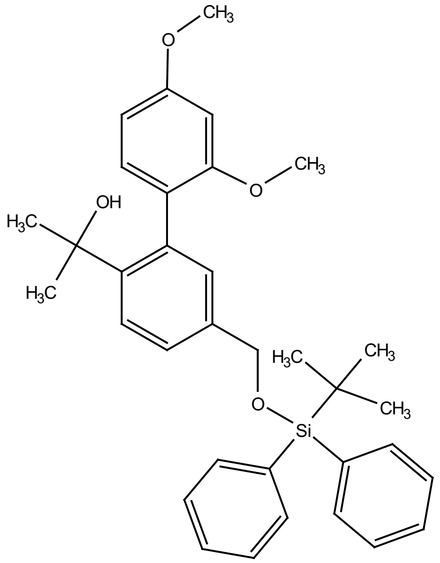

^{1}H NMR (400 MHz, $CDCl_3$) δ 7.70 – 7.68 (m, 4H), 7.58 (d, J = 8.3 Hz, 1H), 7.43 (td, J = 3.0, 1.8 Hz, 1H), 7.40 (q, J = 1.6 Hz, 2H), 7.38 – 7.36 (m, 4H), 7.05 – 7.02 (m, 1H), 6.92 (d, J = 1.9 Hz, 1H), 6.54 – 6.51 (m, 2H), 4.75 (d, J = 4.3 Hz, 2H), 3.85 (s, 3H), 3.72 (s, 3H), 1.52 (s, 3H), 1.39 (s, 3H), 1.07 (s, 9H).

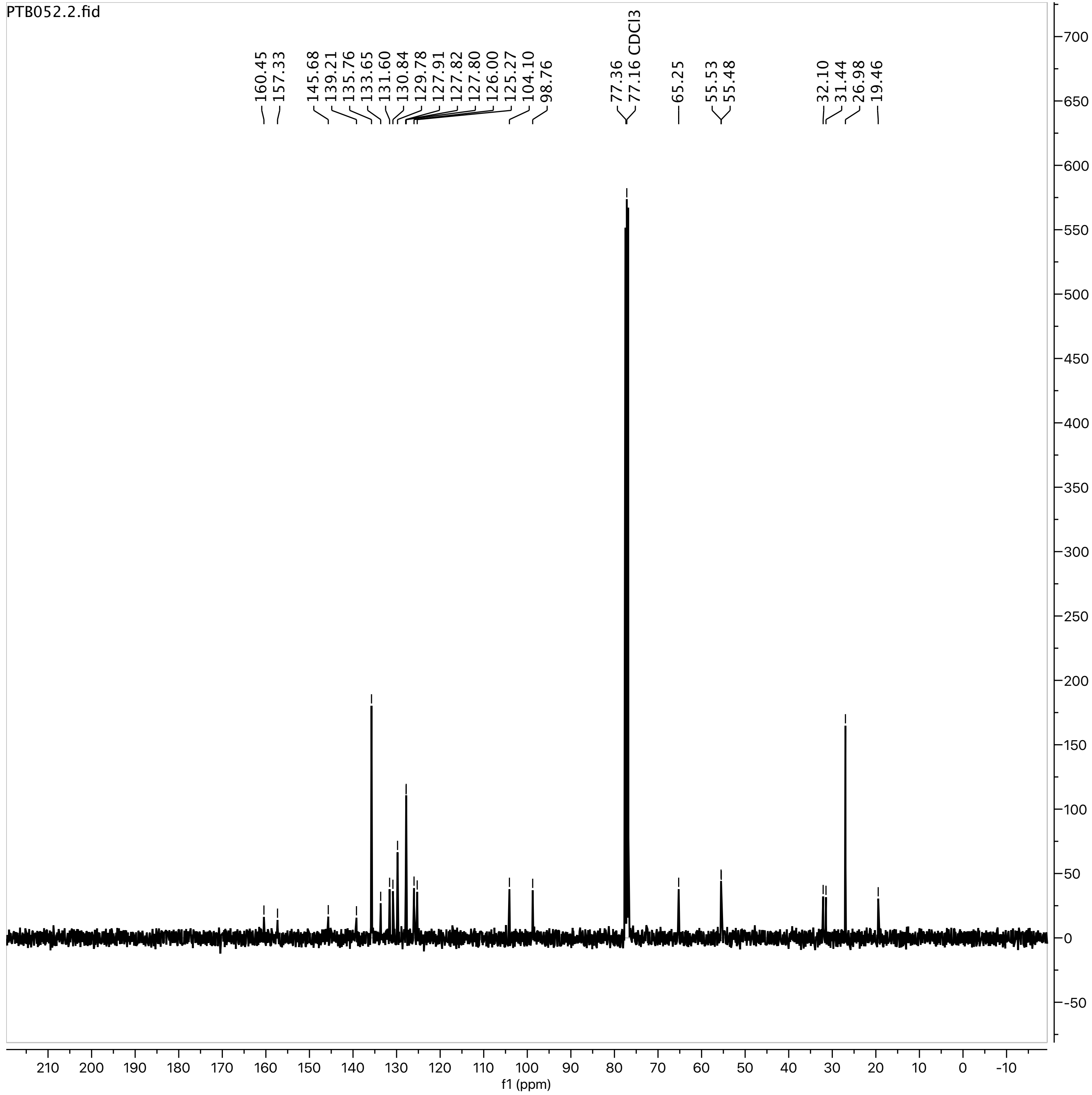

	Parameter	Value
1	Solvent	CDCl3
2	Experiment	1D
3	Spectrometer Frequency	100.62
4	Nucleus	13C

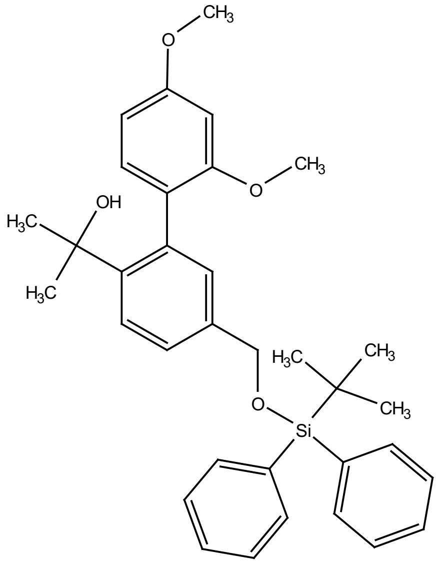

^{13}C NMR (101 MHz, $CDCl_3$) δ 160.45, 157.33, 145.68, 139.21, 135.76, 133.65, 131.60, 130.84, 129.78, 127.91, 127.81 (d, J = 2.2 Hz), 126.00, 125.27, 104.10, 98.76, 77.36, 65.25, 55.51 (d, J = 4.8 Hz), 32.10, 31.44, 26.98, 19.46.

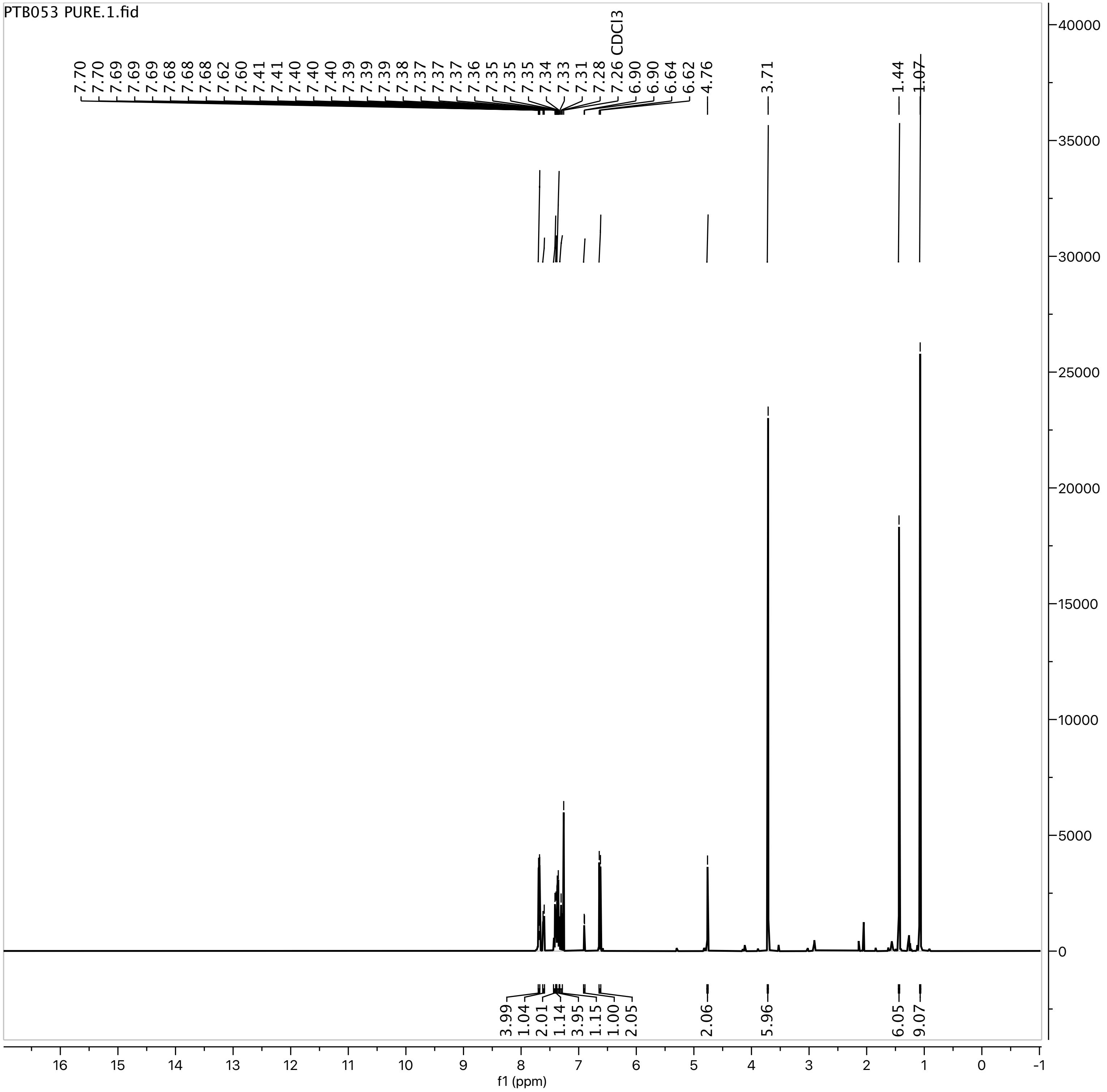

	Parameter	Value
1	Solvent	CDCl3
2	Experiment	1D
3	Spectrometer Frequency	400.13
4	Nucleus	1H

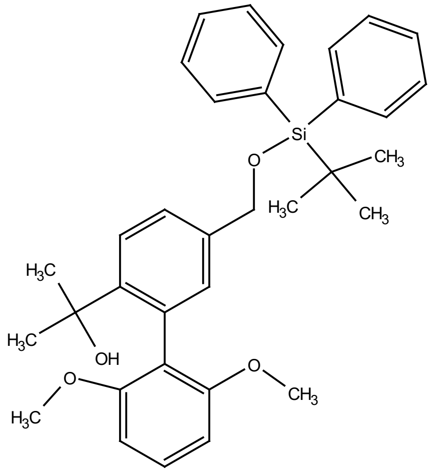

^{1}H NMR (400 MHz, $CDCl_3$) δ 7.70 – 7.67 (m, 4H), 7.61 (d, J = 8.1 Hz, 1H), 7.41 (d, J = 2.8 Hz, 2H), 7.39 (t, J = 1.5 Hz, 1H), 7.38 – 7.34 (m, 4H), 7.31 (t, J = 8.4 Hz, 1H), 6.90 (d, J = 2.0 Hz, 1H), 6.63 (d, J = 8.5 Hz, 2H), 4.76 (s, 2H), 3.71 (s, 6H), 1.44 (s, 6H), 1.07 (s, 9H).

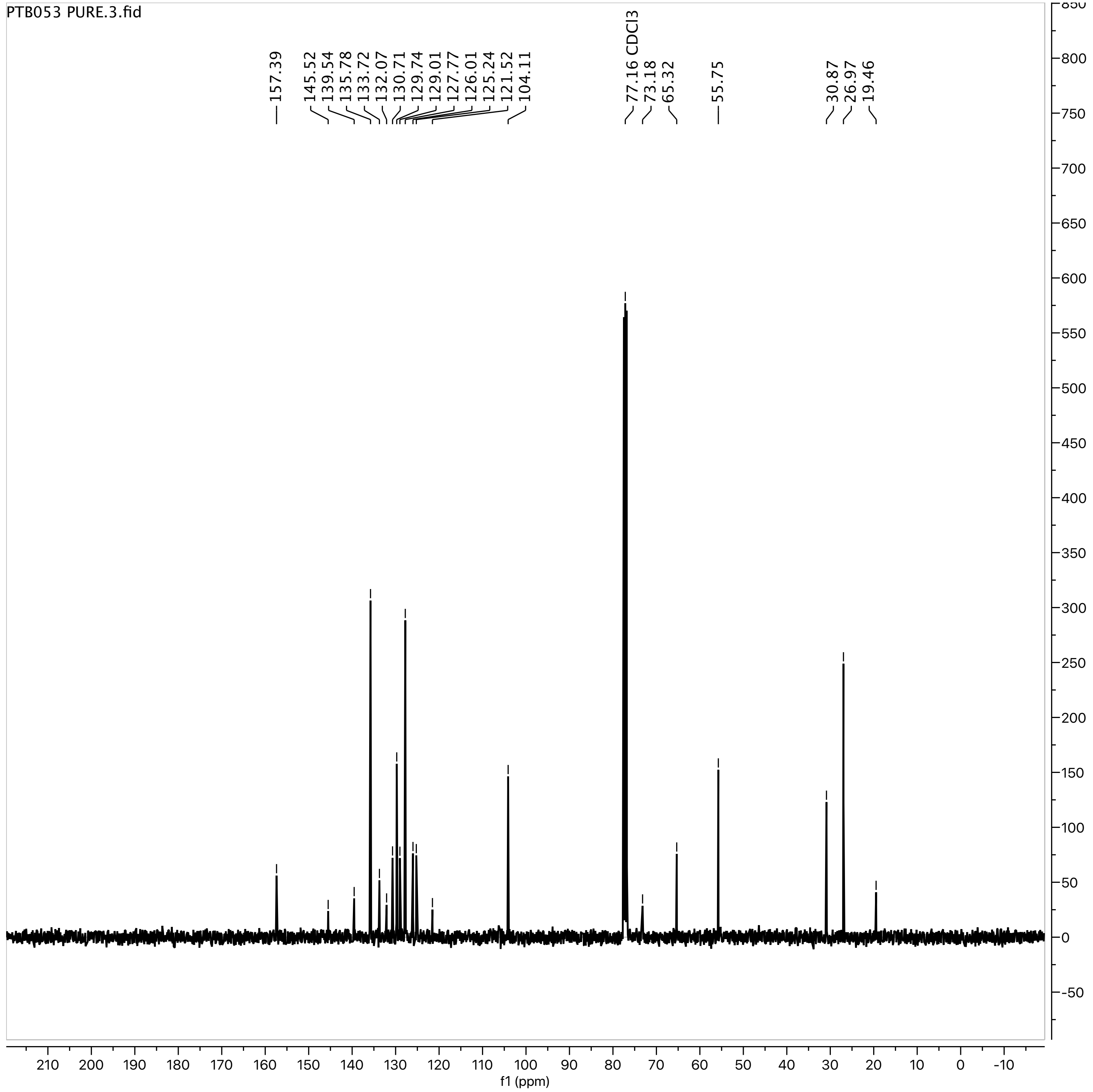

	Parameter	Value
1	Solvent	CDCl3
2	Experiment	1D
3	Spectrometer Frequency	100.62
4	Nucleus	13C

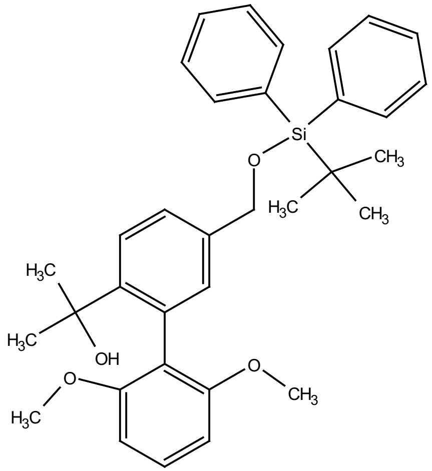

^{13}C NMR (101 MHz, $CDCl_3$) δ 157.39, 145.52, 139.54, 135.78, 133.72, 132.07, 130.71, 129.74, 129.01, 127.77, 126.01, 125.24, 121.52, 104.11, 73.18, 65.32, 55.75, 30.87, 26.97, 19.46.

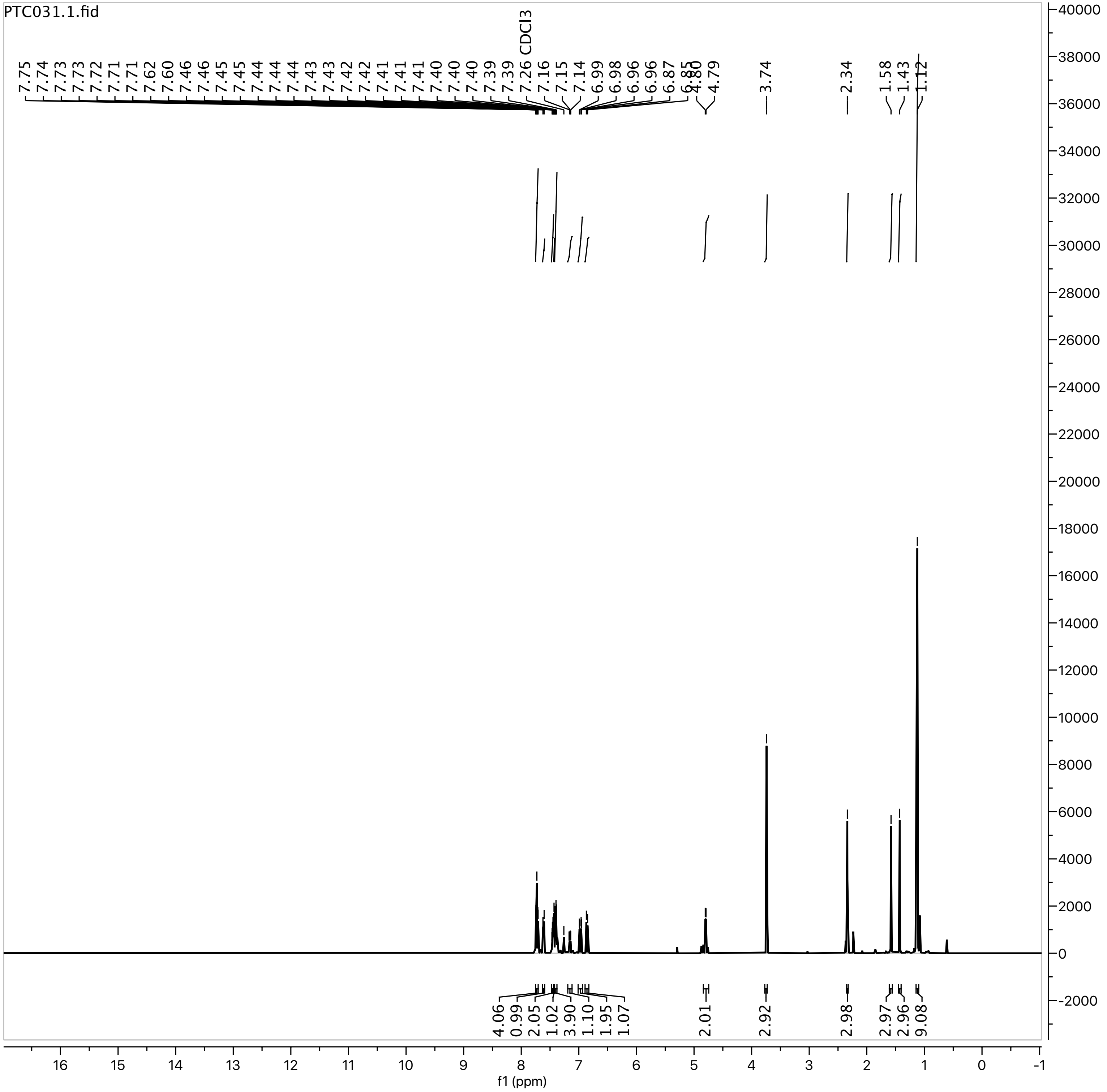

	Parameter	Value
1	Solvent	CDCl3
2	Experiment	1D
3	Spectrometer Frequency	400.13
4	Nucleus	1H

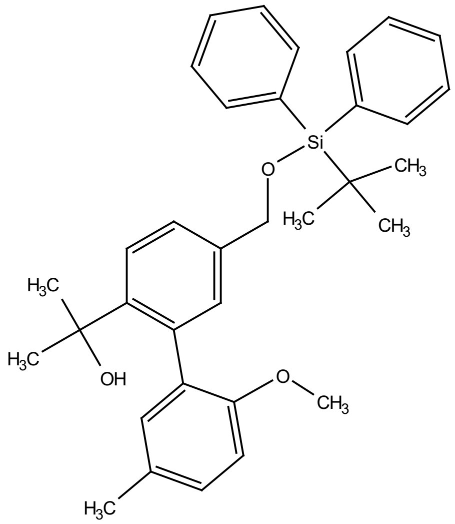

^{1}H NMR (400 MHz, $CDCl_3$) δ 7.75 – 7.71 (m, 4H), 7.61 (d, J = 8.1 Hz, 1H), 7.48 – 7.44 (m, 2H), 7.43 (d, J = 1.5 Hz, 1H), 7.42 – 7.38 (m, 4H), 7.15 (dd, J = 8.4, 3.1 Hz, 1H), 6.97 (dd, J = 11.6, 2.3 Hz, 2H), 6.86 (d, J = 8.4 Hz, 1H), 4.80 (d, J = 5.1 Hz, 2H), 3.74 (s, 3H), 2.34 (s, 3H), 1.58 (s, 3H), 1.43 (s, 3H), 1.12 (s, 9H), .

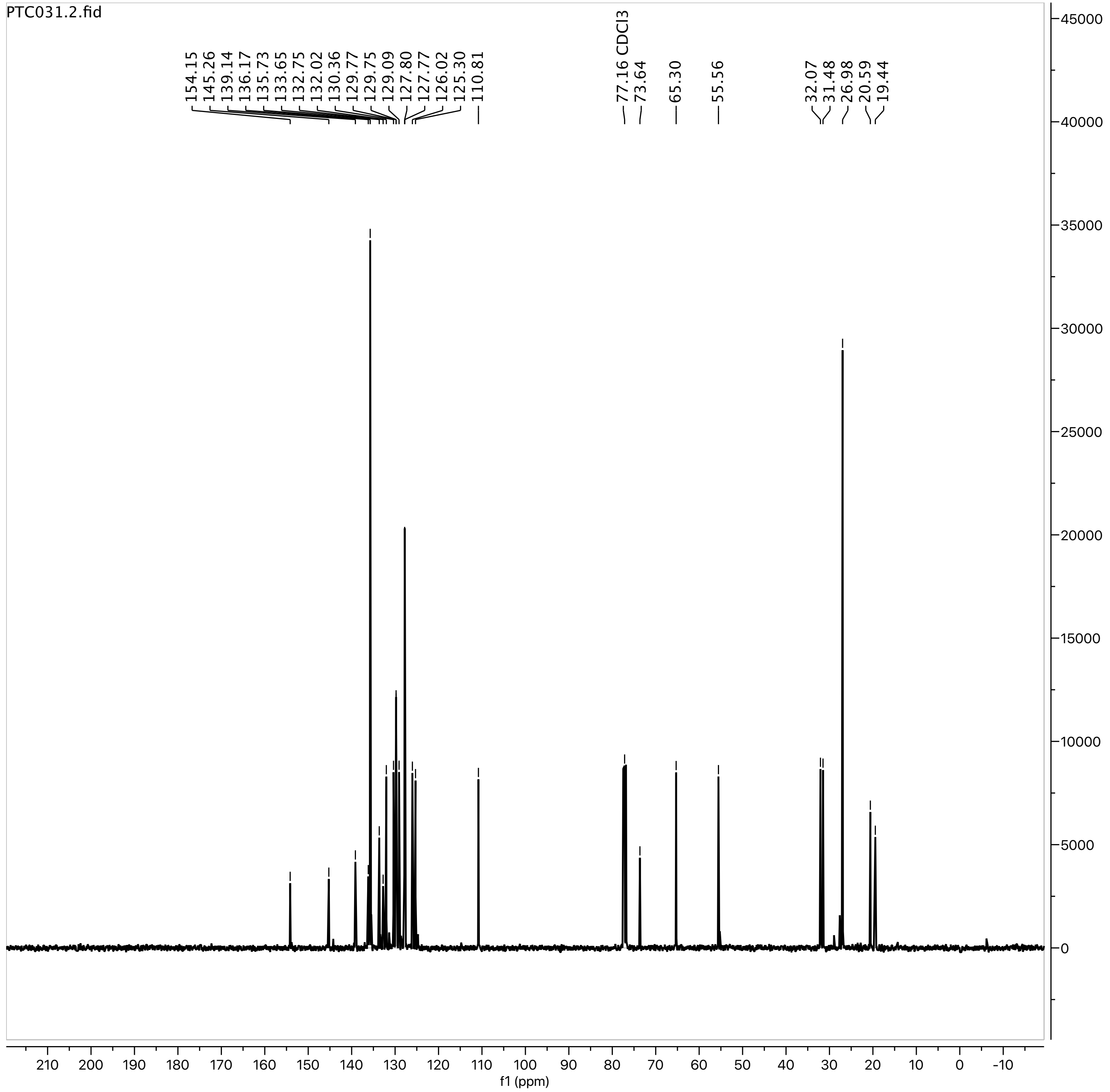

	Parameter	Value
1	Solvent	CDCl3
2	Experiment	1D
3	Spectrometer Frequency	100.62
4	Nucleus	13C

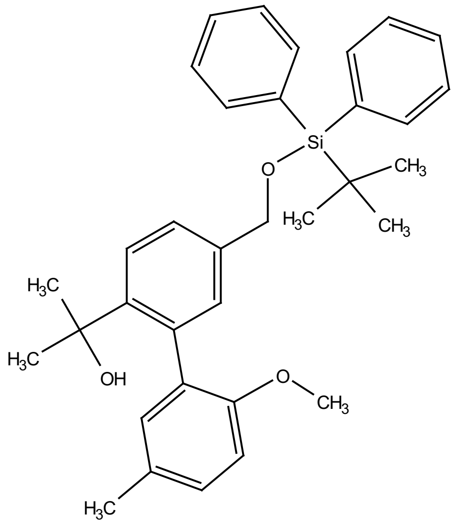

^{13}C NMR (101 MHz, $CDCl_3$) δ 154.15, 145.26, 139.14, 136.17, 135.73, 133.65, 132.75, 132.02, 130.36, 129.76 (d, J = 1.8 Hz), 129.09, 127.79 (d, J = 2.9 Hz), 126.02, 125.30, 110.81, 73.64, 65.30, 55.56, 32.07, 31.48, 26.98, 20.59, 19.44.

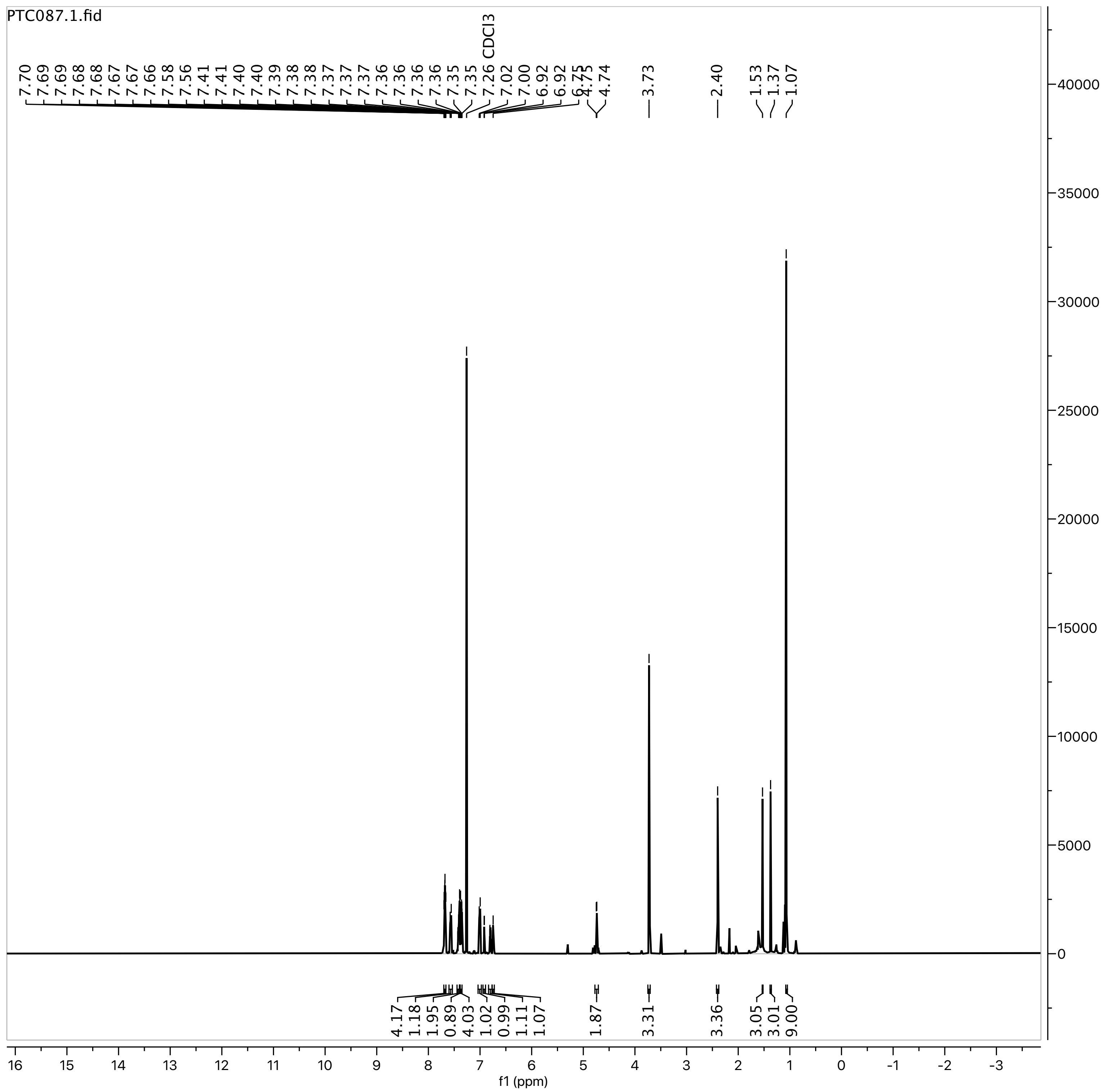

	Parameter	Value
1	Solvent	CDCl3
2	Experiment	1D
3	Spectrometer Frequency	400.13
4	Nucleus	1H

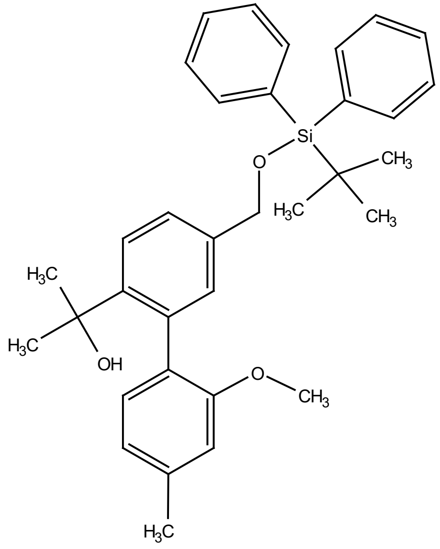

^{1}H NMR (400 MHz, $CDCl_3$) δ 7.68 (ddd, J = 7.9, 5.3, 1.5 Hz, 4H), 7.57 (d, J = 8.3 Hz, 1H), 7.45 – 7.40 (m, 2H), 7.39 (d, J = 1.6 Hz, 1H), 7.39 – 7.35 (m, 4H), 7.01 (d, J = 7.6 Hz, 1H), 6.92 (d, J = 1.7 Hz, 1H), 6.80 (ddd, J = 7.6, 1.6, 0.8 Hz, 1H), 6.75 (s, 1H), 4.74 (d, J = 4.0 Hz, 2H), 3.73 (s, 3H), 2.40 (s, 3H), 1.53 (s, 3H), 1.37 (s, 3H), 1.07 (s, 9H).

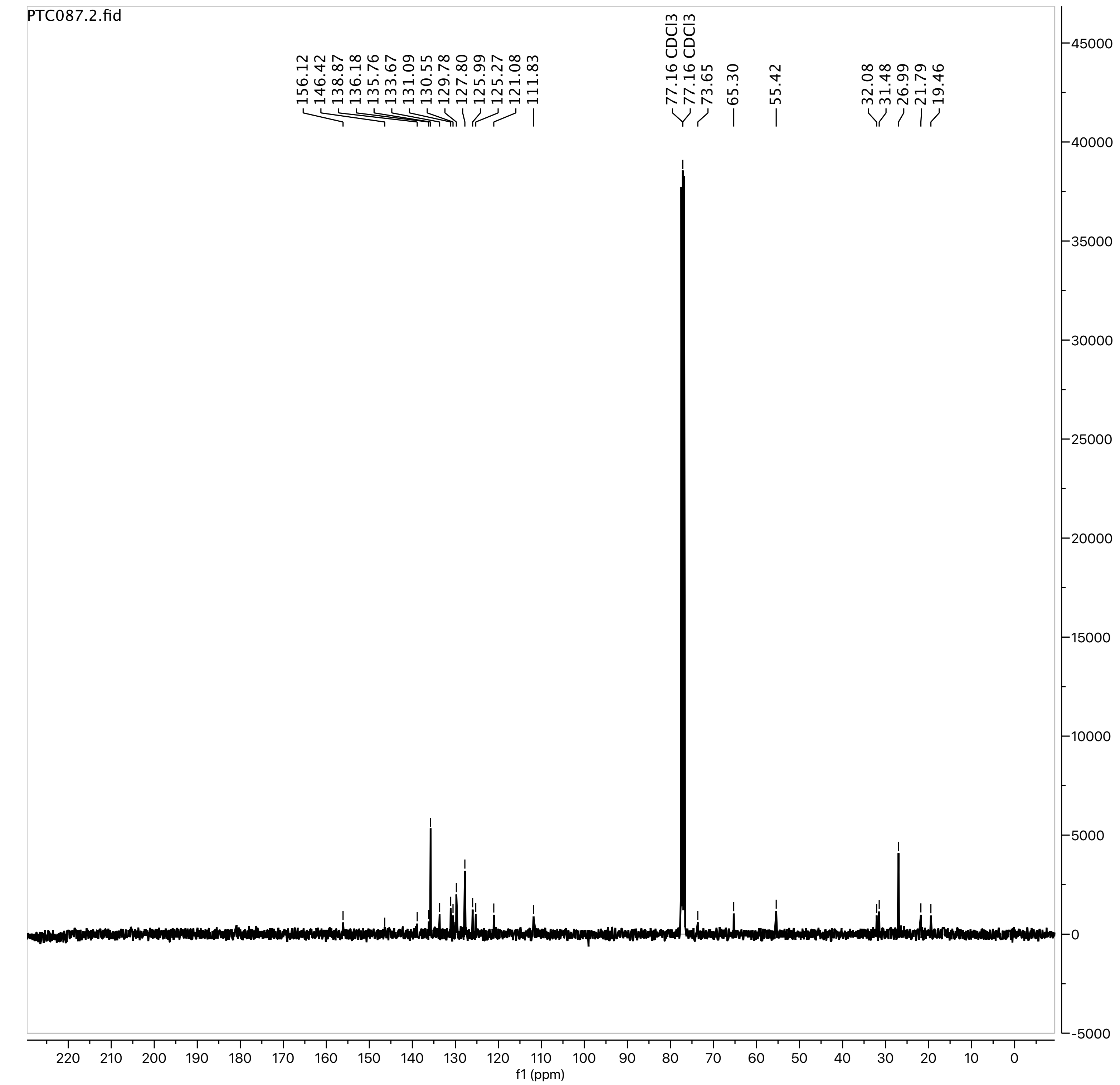

	Parameter	Value
1	Solvent	CDCl3
2	Experiment	1D
3	Spectrometer Frequency	100.62
4	Nucleus	13C

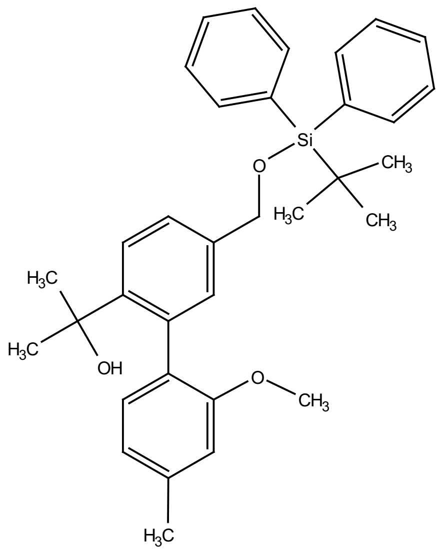

^{13}C NMR (101 MHz, $CDCl_3$) δ 156.12, 146.42, 138.87, 136.18, 135.76, 133.67, 131.09, 130.55, 129.78, 127.80, 125.99, 125.27, 121.08, 111.83, 73.65, 65.30, 55.42, 32.08, 31.48, 26.99, 21.79, 19.46.

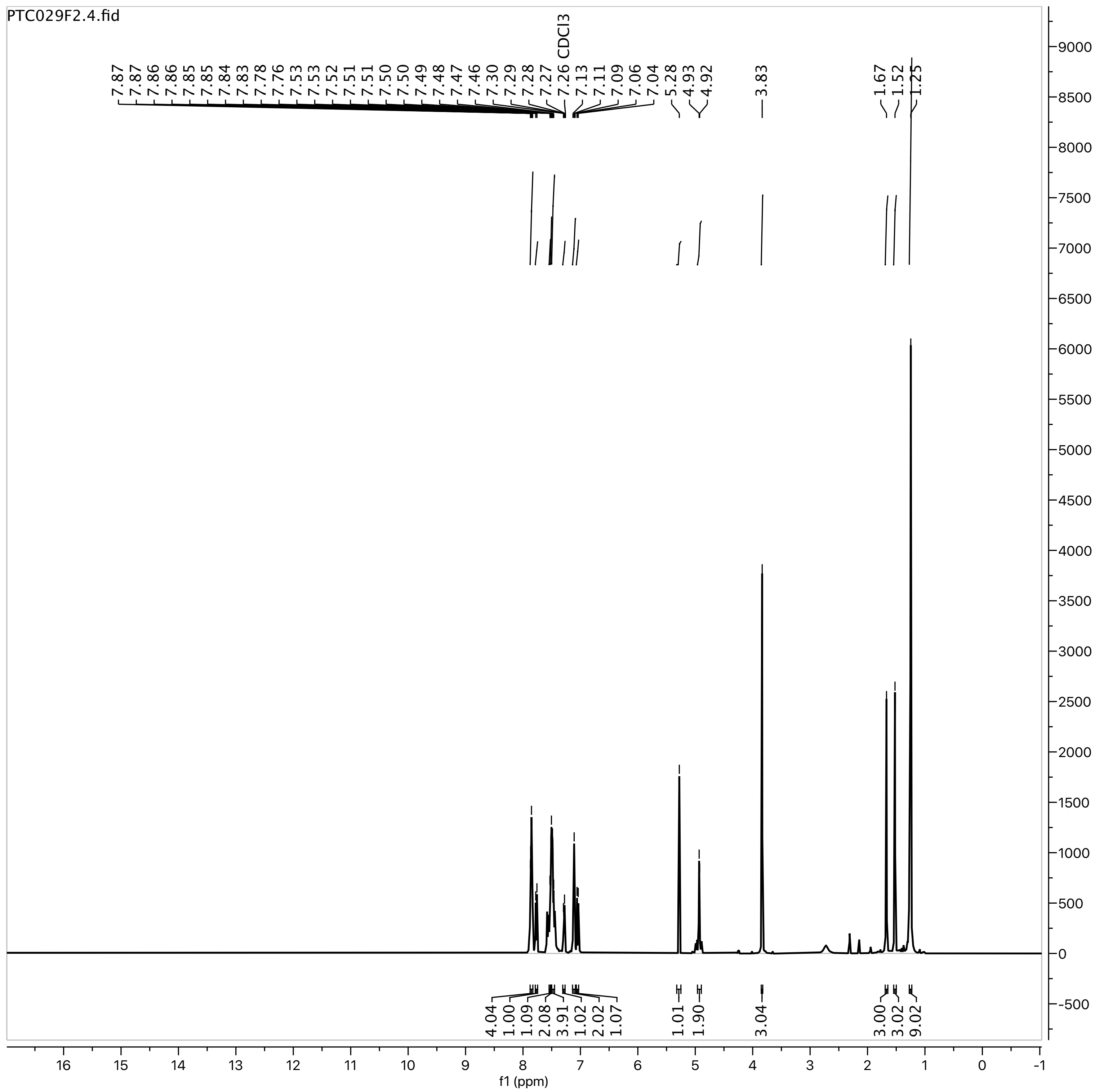

	Parameter	Value
1	Solvent	CDCl3
2	Experiment	1D
3	Spectrometer Frequency	400.13
4	Nucleus	1H

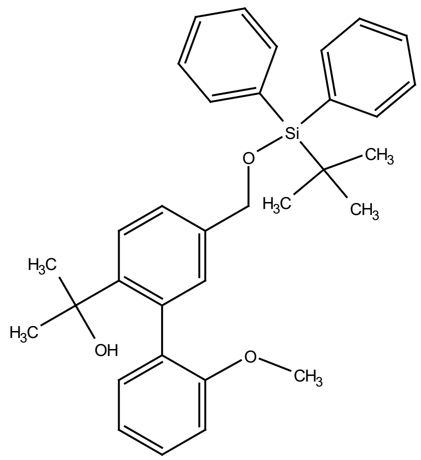

^{1}H NMR (400 MHz, $CDCl_3$) δ 7.85 (ddd, J = 7.7, 5.5, 1.8 Hz, 4H), 7.77 (d, J = 8.3 Hz, 1H), 7.53 (d, J = 1.7 Hz, 1H), 7.51 (dd, J = 3.6, 1.8 Hz, 2H), 7.50 – 7.45 (m, 4H), 7.28 (dd, J = 7.4, 1.9 Hz, 1H), 7.11 (s, 2H), 7.05 (d, J = 8.4 Hz, 1H), 5.28 (s, 1H), 4.93 (d, J = 3.7 Hz, 2H), 3.83 (s, 3H), 1.67 (s, 3H), 1.52 (s, 3H), 1.25 (s, 9H).

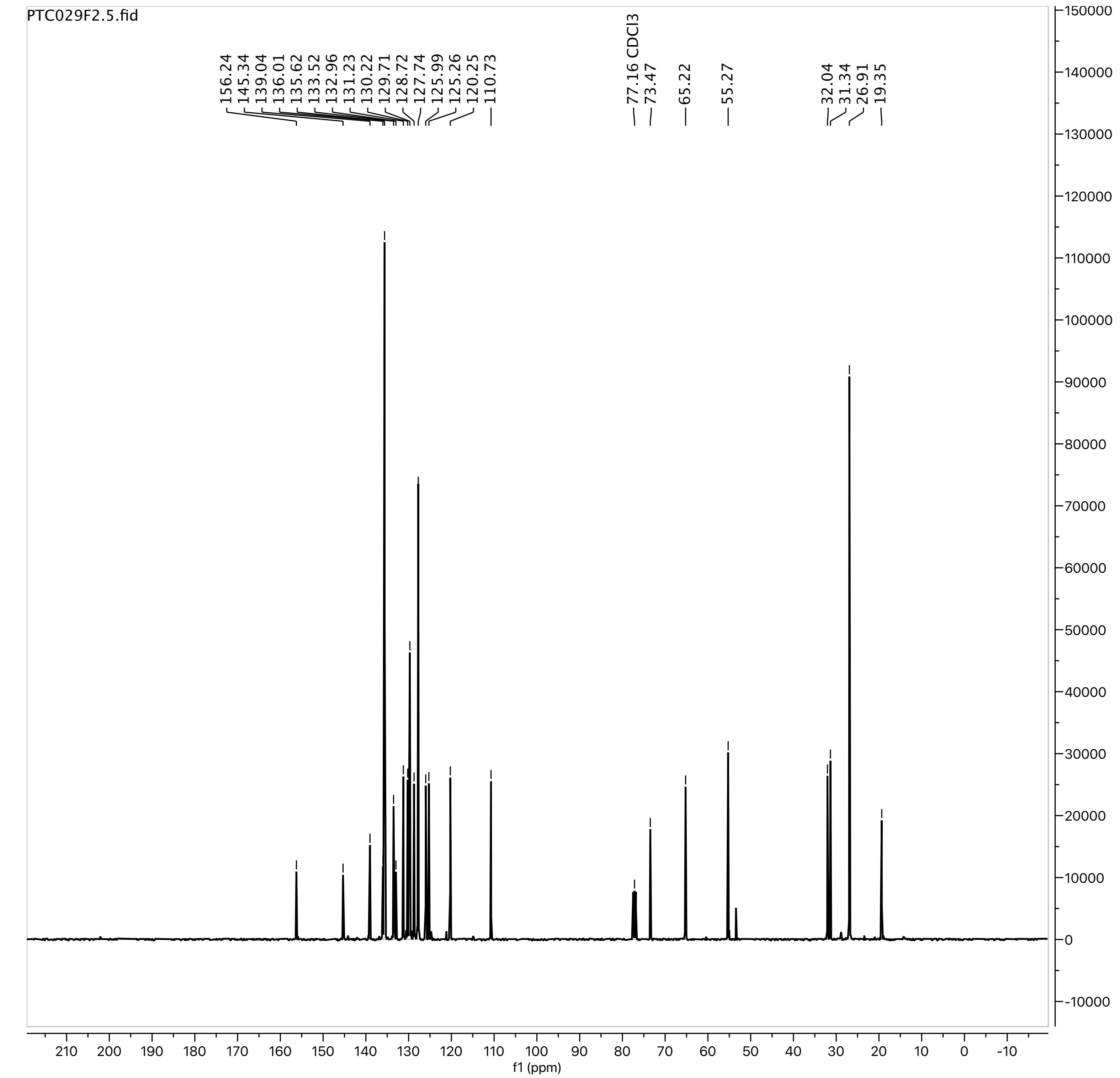

	Parameter	Value
1	Solvent	CDCl3
2	Experiment	1D
3	Spectrometer Frequency	100.62
4	Nucleus	13C

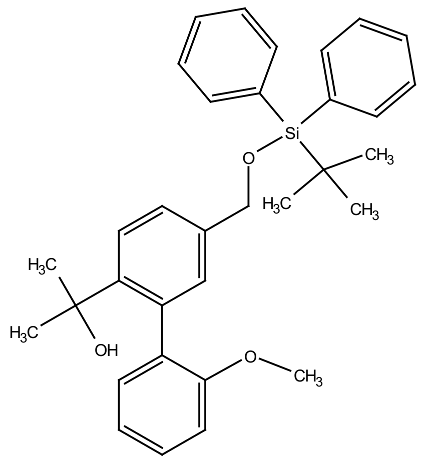

^{13}C NMR (101 MHz, $CDCl_3$) δ 156.24, 145.34, 139.04, 136.01, 135.62, 133.52, 132.96, 131.23, 130.22, 129.71, 128.72, 127.74, 125.99, 125.26, 120.25, 110.73, 73.47, 65.22, 55.27, 32.04, 31.34, 26.91, 19.35.

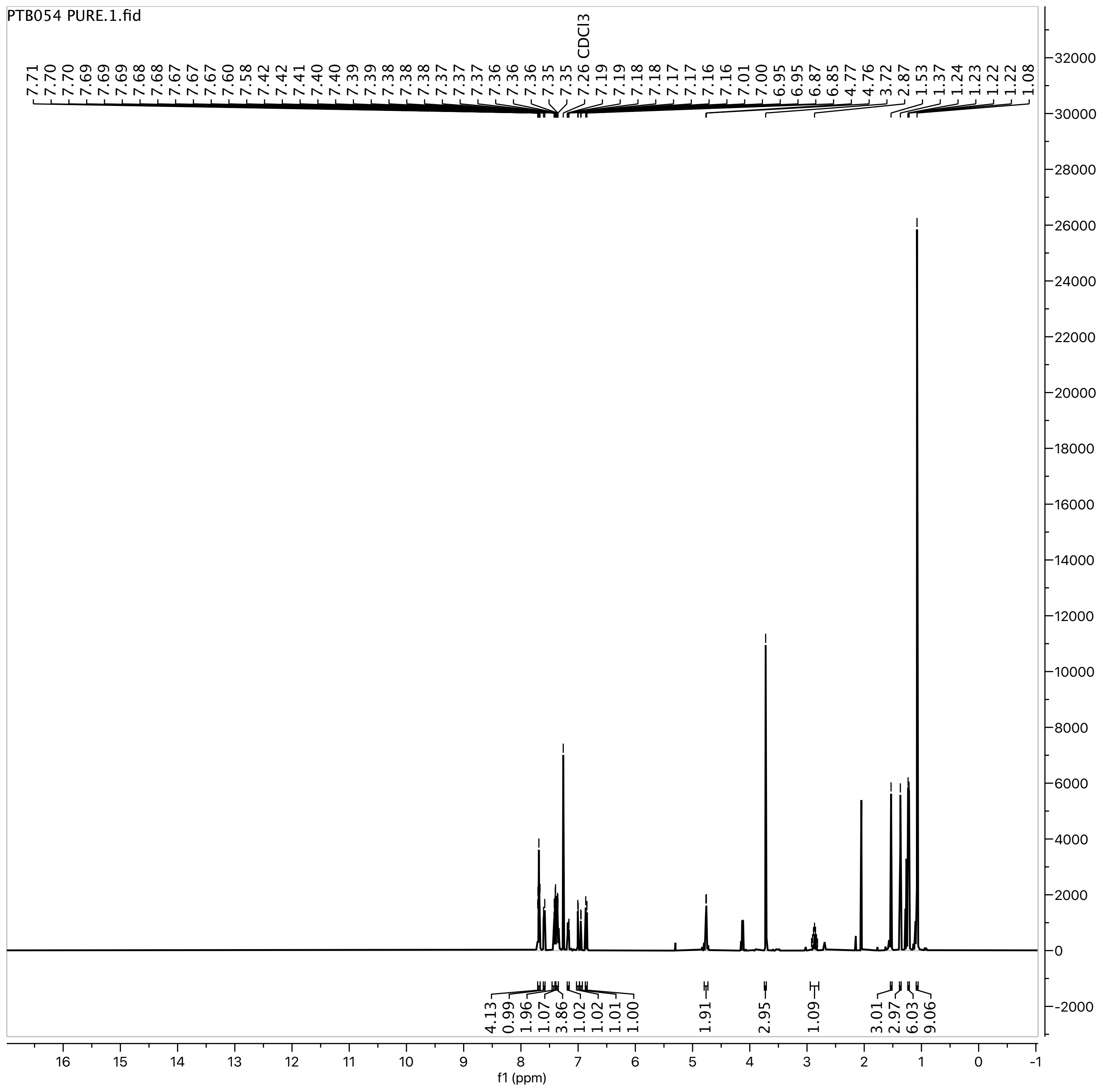

	Parameter	Value
1	Solvent	CDCl3
2	Experiment	1D
3	Spectrometer Frequency	400.13
4	Nucleus	1H

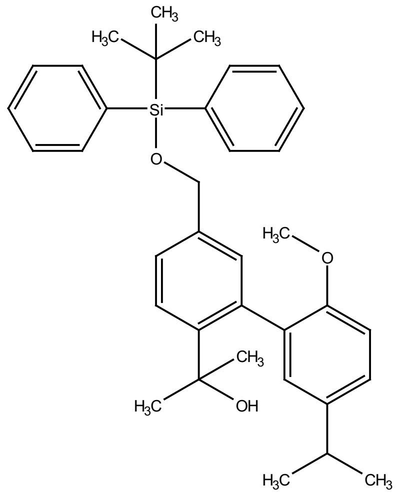

^{1}H NMR (400 MHz, $CDCl_3$) δ 7.71 – 7.66 (m, 4H), 7.59 (d, J = 8.3 Hz, 1H), 7.46 – 7.40 (m, 2H), 7.40 (d, J = 1.9 Hz, 1H), 7.39 – 7.35 (m, 4H), 7.17 (ddd, J = 8.4, 2.4, 0.6 Hz, 1H), 7.00 (d, J = 2.5 Hz, 1H), 6.95 (d, J = 2.0 Hz, 1H), 6.86 (d, J = 8.5 Hz, 1H), 4.76 (d, J = 2.9 Hz, 2H), 3.72 (s, 3H), 2.87 (hept, J = 6.8 Hz, 1H), 1.53 (s, 3H), 1.37 (s, 3H), 1.23 (dd, J = 6.9, 0.9 Hz, 6H), 1.08 (s, 9H).

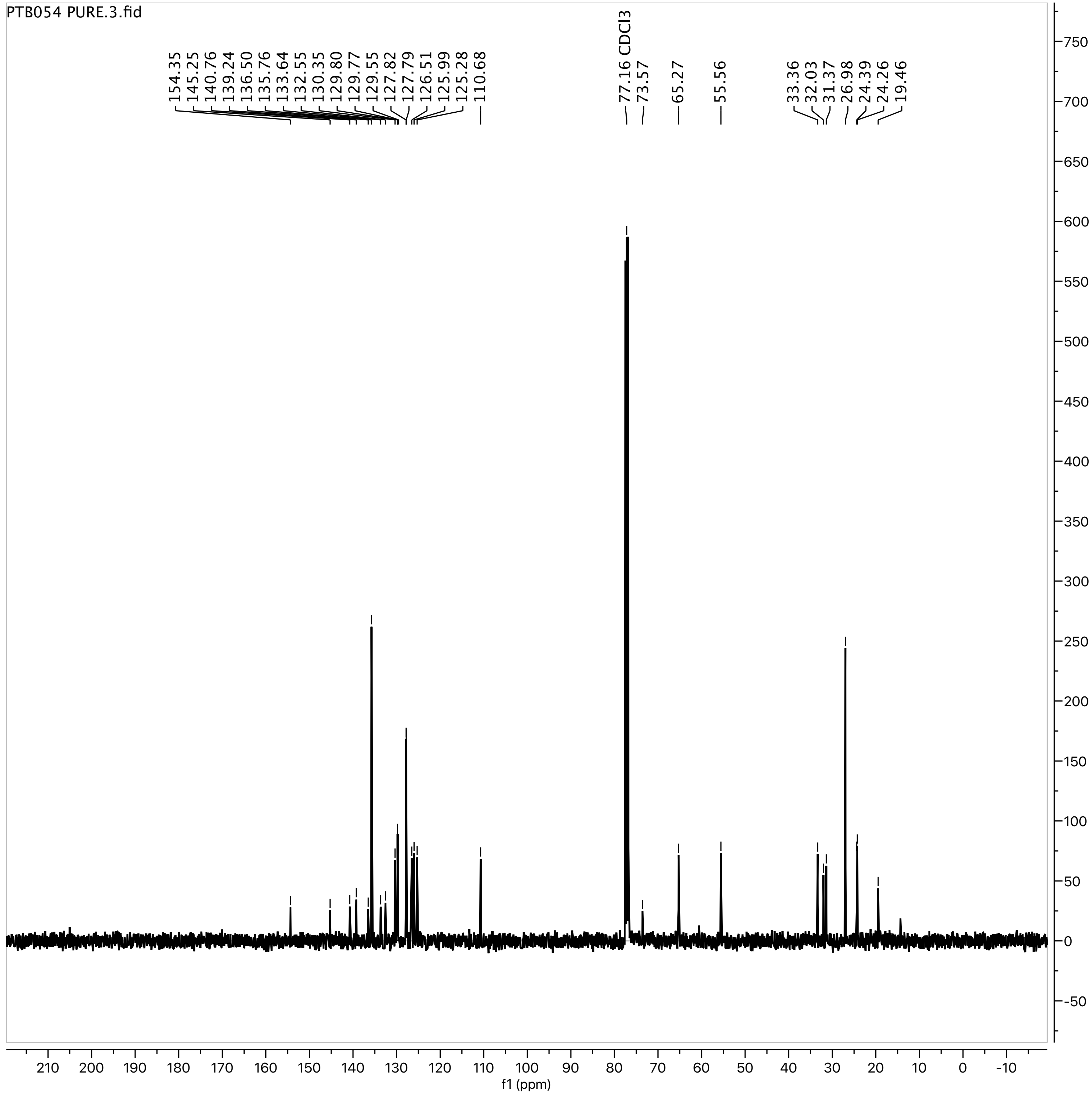

	Parameter	Value
1	Solvent	CDCl3
2	Experiment	1D
3	Spectrometer Frequency	100.62
4	Nucleus	13C

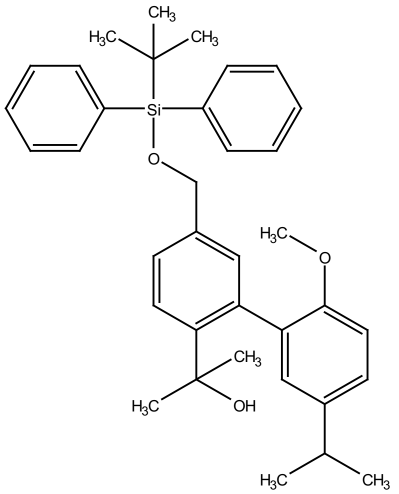

^{13}C NMR (101 MHz, $CDCl_3$) δ 154.35, 145.25, 140.76, 139.24, 136.50, 135.76, 133.64, 132.55, 130.35, 129.78 (d, J = 2.9 Hz), 129.55, 127.80 (d, J = 2.9 Hz), 126.51, 125.99, 125.28, 110.68, 73.57, 65.27, 55.56, 33.36, 32.03, 31.37, 26.98, 24.39, 24.26, 19.46.

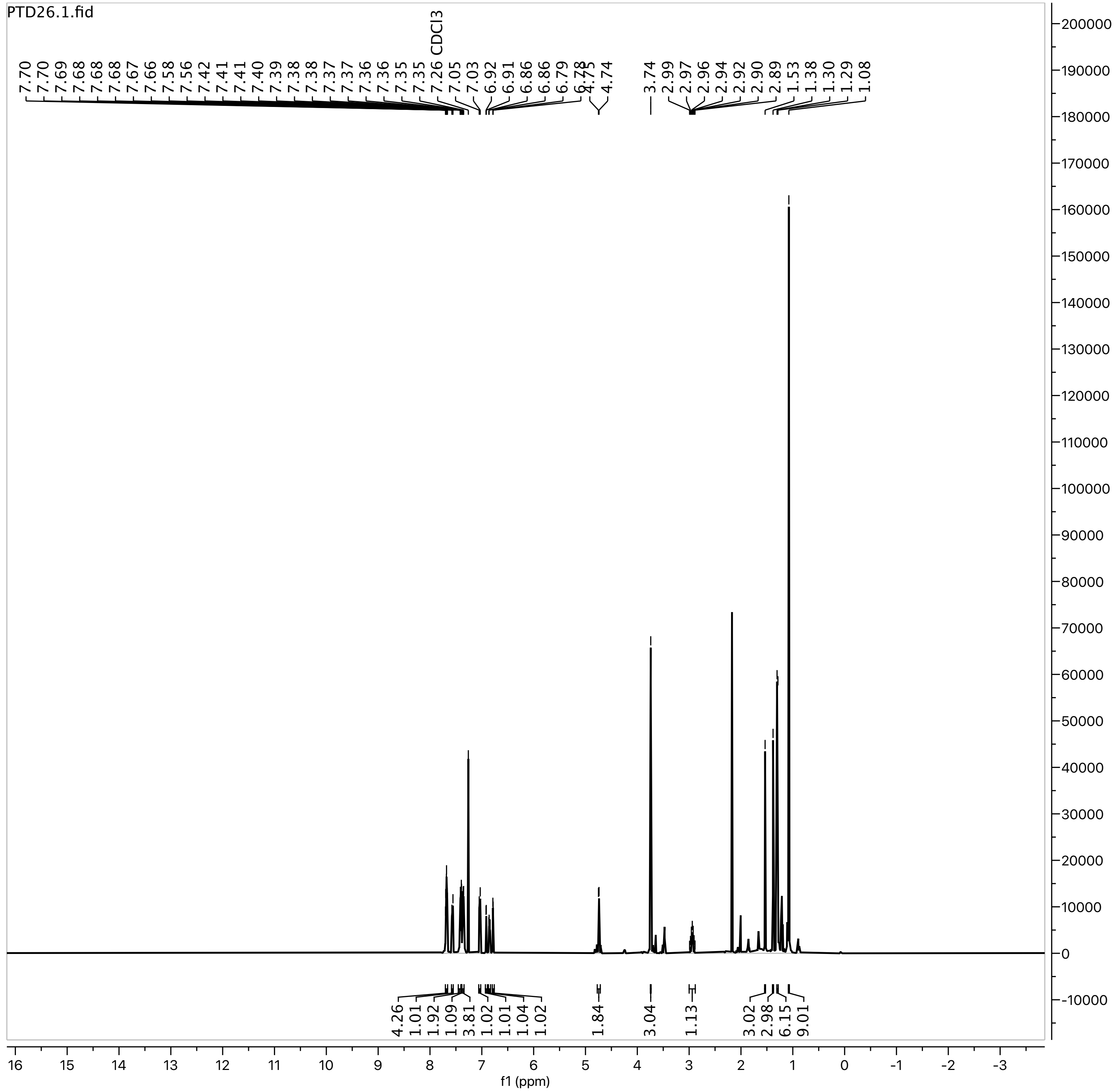

	Parameter	Value
1	Solvent	CDCl3
2	Experiment	1D
3	Spectrometer Frequency	400.13
4	Nucleus	1H

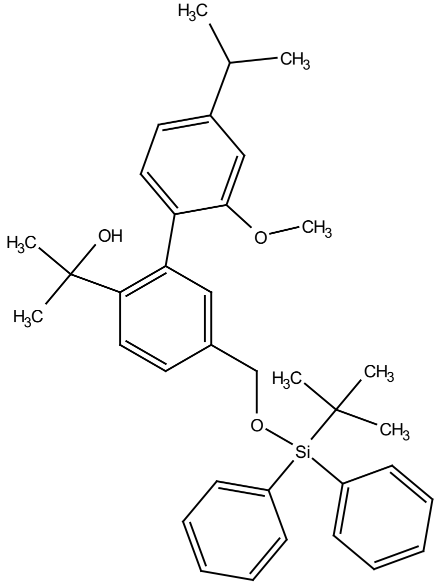

^{1}H NMR (400 MHz, $CDCl_3$) δ 7.68 (ddd, *J* = 8.1, 5.4, 1.5 Hz, 4H), 7.57 (d, *J* = 8.2 Hz, 1H), 7.45 – 7.40 (m, 2H), 7.39 (d, *J* = 1.6 Hz, 1H), 7.39 – 7.34 (m, 4H), 7.04 (d, *J* = 7.7 Hz, 1H), 6.92 (d, *J* = 2.1 Hz, 1H), 6.85 (dd, *J* = 7.7, 1.7 Hz, 1H), 6.78 (d, *J* = 1.8 Hz, 1H), 4.74 (d, *J* = 4.2 Hz, 2H), 3.74 (s, 3H), 2.94 (hept, *J* = 7.0 Hz, 1H), 1.30 (s, 3H), 1.29 (s, 3H), 1.08 (s, 9H).

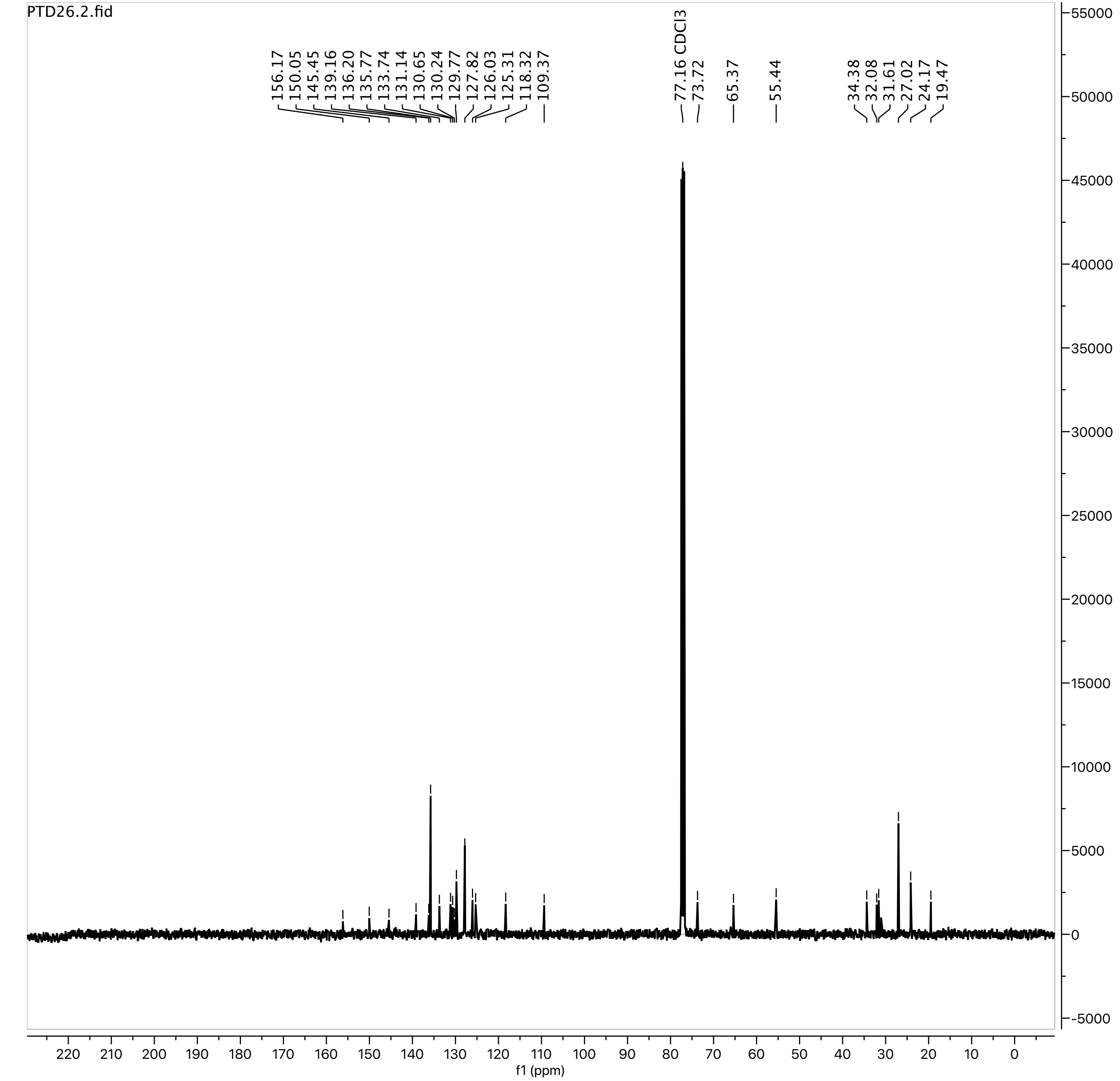

	Parameter	Value
1	Solvent	CDCl3
2	Experiment	1D
3	Spectrometer Frequency	100.62
4	Nucleus	13C

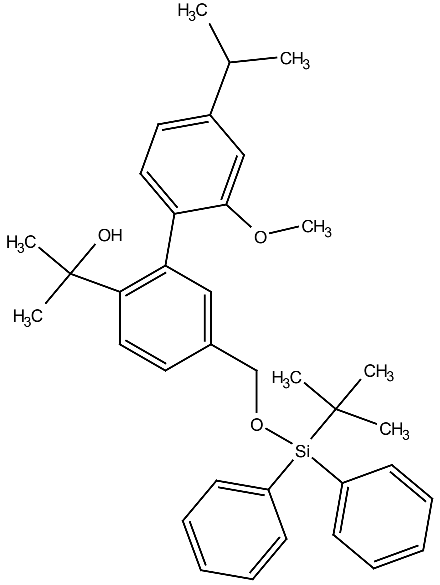

^{13}C NMR (101 MHz, $CDCl_3$) δ 156.17, 150.05, 145.45, 139.16, 136.20, 135.77, 133.74, 131.14, 130.65, 130.24, 129.77, 127.82, 126.03, 125.31, 118.32, 109.37, 73.72, 65.37, 55.44, 34.38, 32.08, 31.61, 27.02, 24.17, 19.47.

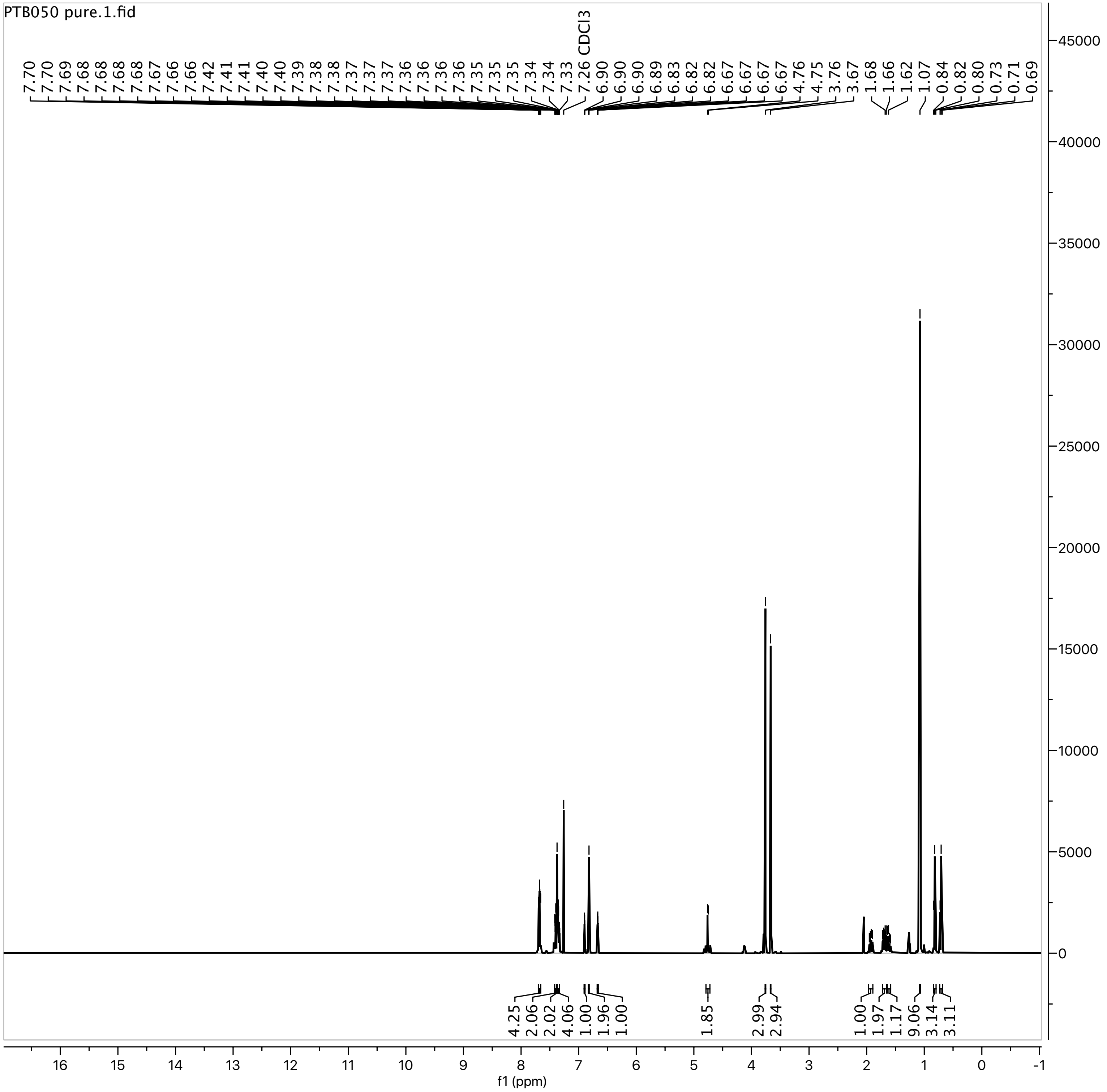

	Parameter	Value
1	Solvent	CDCl3
2	Experiment	1D
3	Spectrometer Frequency	400.13
4	Nucleus	1H

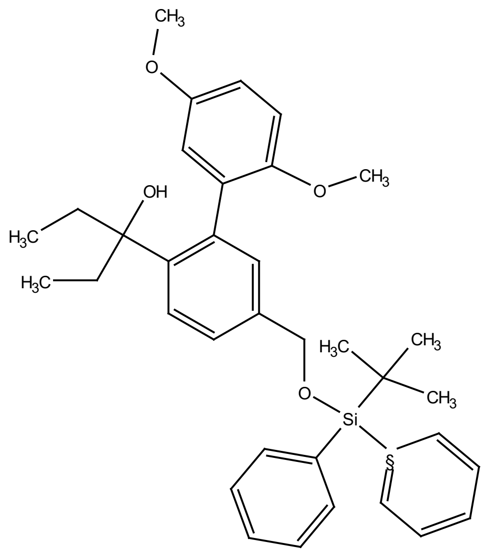

^{1}H NMR (400 MHz, $CDCl_3$) δ 7.70 – 7.66 (m, 4H), 7.42 – 7.39 (m, 2H), 7.38 (d, 2H), 7.37 – 7.33 (m, 4H), 6.90 (dd, 1H), 6.83 – 6.82 (m, 2H), 6.67 (dd, 1H), 4.76 (d, 2H), 3.76 (s, 3H), 3.67 (s, 3H), 1.93 (dd, 1H), 1.73 – 1.66 (m, 2H), 1.61 (dd, 1H), 1.07 (s, 9H), 0.82 (t, 3H), 0.71 (t, 3H).

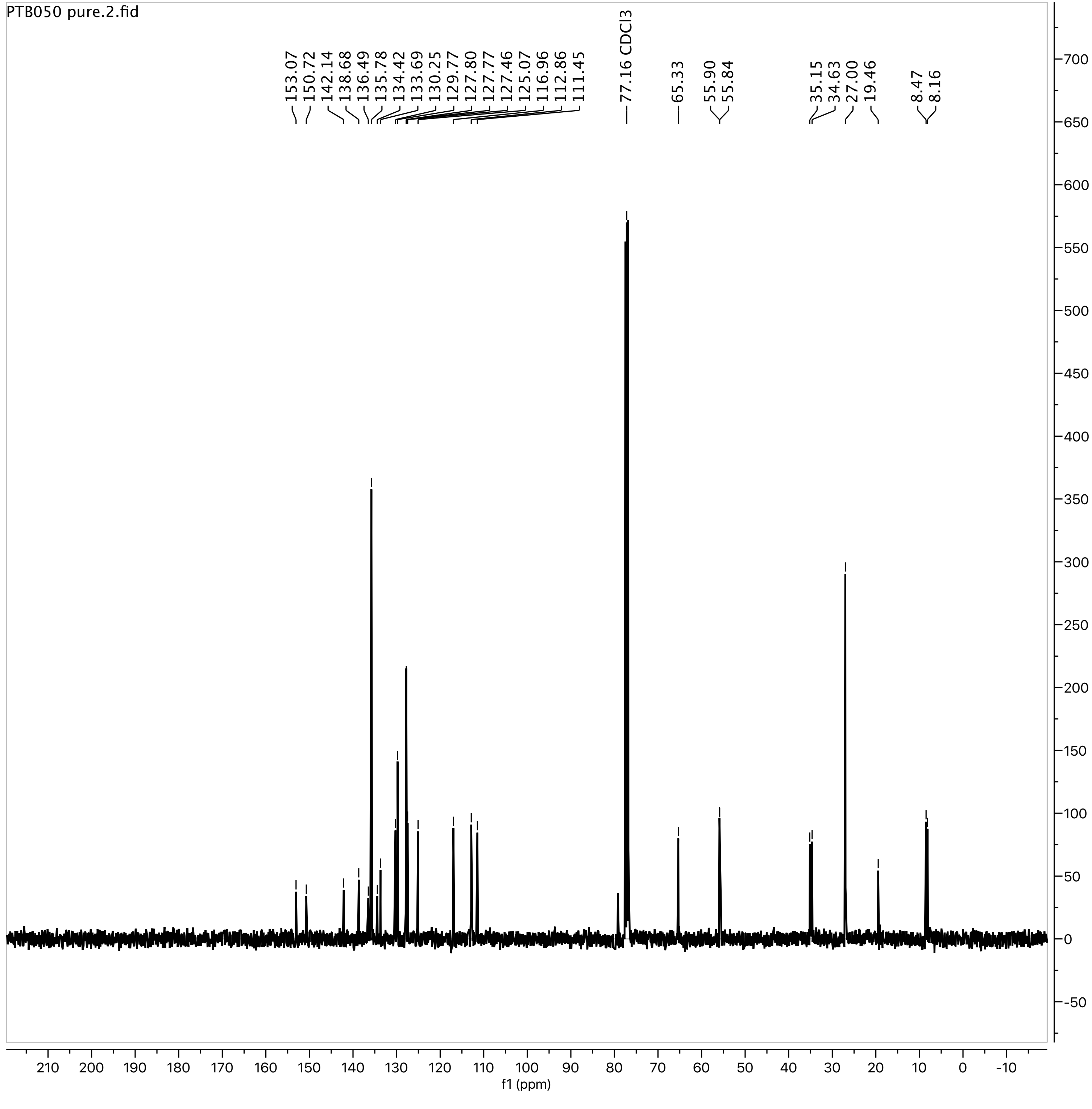

	Parameter	Value
1	Solvent	CDCl3
2	Experiment	1D
3	Spectrometer Frequency	100.62
4	Nucleus	13C

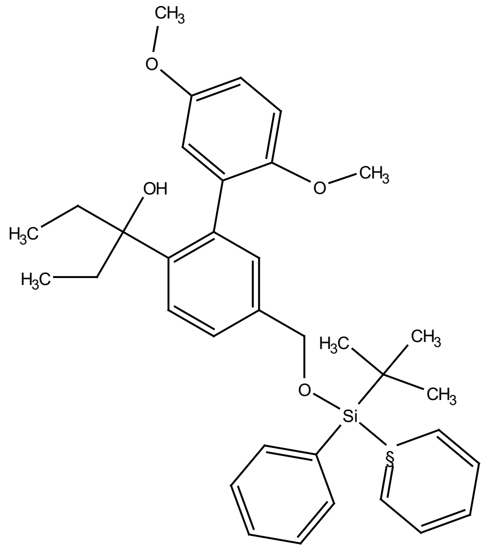

^{13}C NMR (101 MHz, $CDCl_3$) δ 153.07, 150.72, 142.14, 138.68, 136.49, 135.78, 134.42, 133.69, 130.25, 129.77, 127.78 (d, J = 2.9 Hz), 127.46, 125.07, 116.96, 112.86, 111.45, 65.33, 55.87 (d, J = 5.9 Hz), 35.15, 34.63, 27.00, 19.46, 8.47, 8.16.

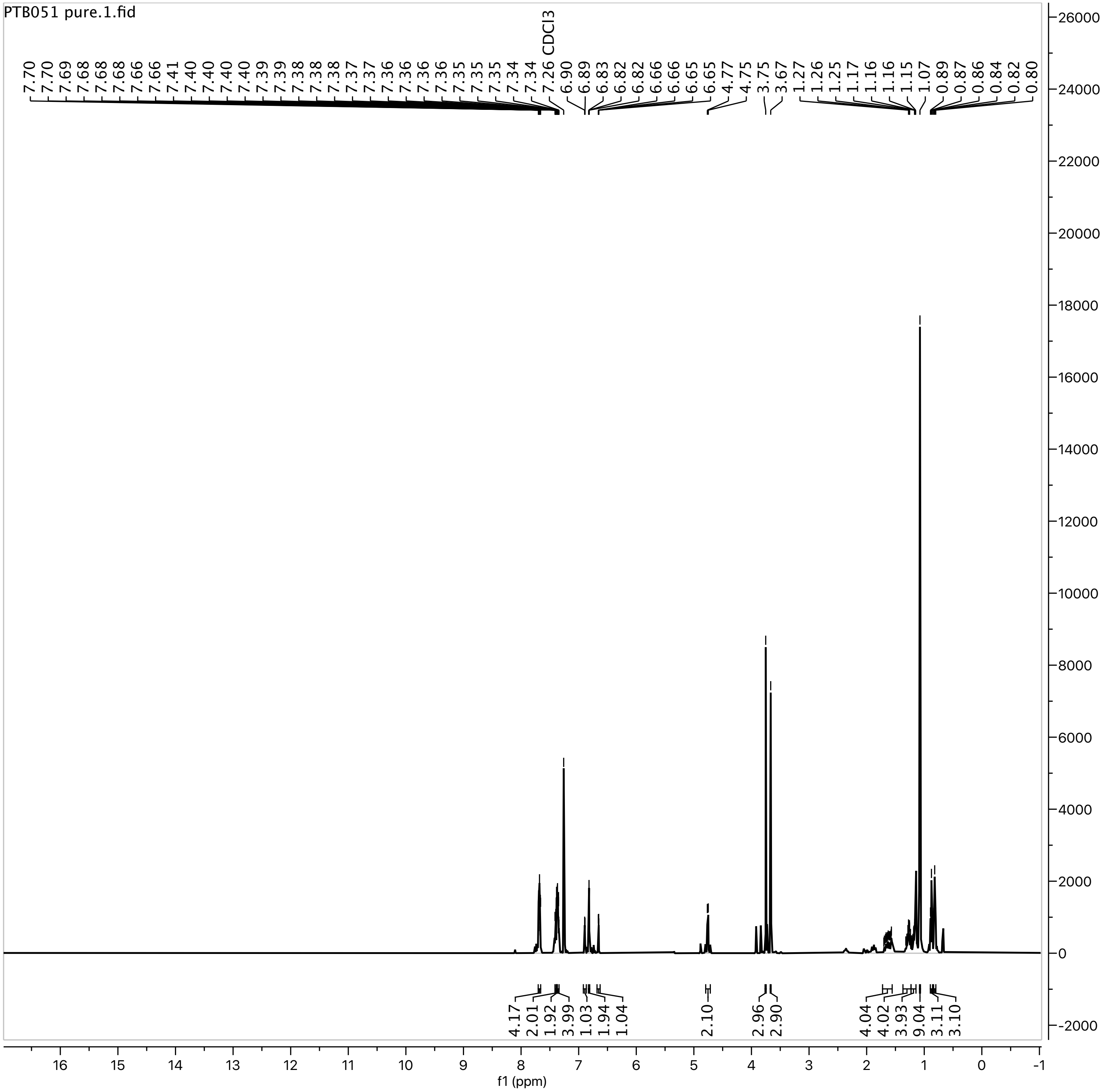

	Parameter	Value
1	Solvent	CDCl3
2	Experiment	1D
3	Spectrometer Frequency	400.13
4	Nucleus	1H

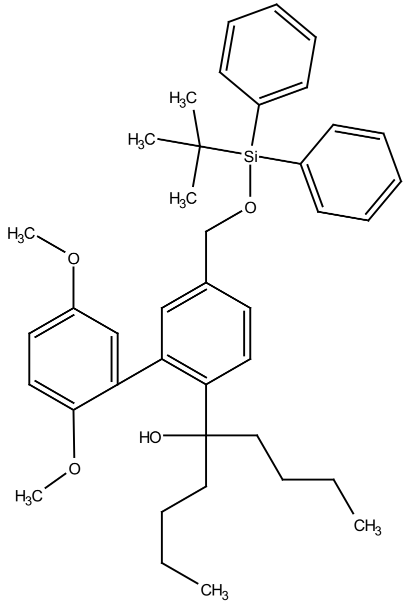

^{1}H NMR (400 MHz, $CDCl_3$) δ 7.70 – 7.66 (m, 4H), 7.41 – 7.39 (m, 2H), 7.38 (d, 2H), 7.37 – 7.34 (m, 4H), 6.89 (d, 1H), 6.83 – 6.81 (m, 2H), 6.65 (dd, 1H), 4.76 (d, 2H), 3.75 (s, 3H), 3.67 (s, 3H), 1.72 – 1.56 (m, 4H), 1.37 – 1.23 (m, 4H), 1.23 – 1.14 (m, 4H), 1.07 (s, 9H), 0.87 (t, 3H), 0.82 (t, 3H).

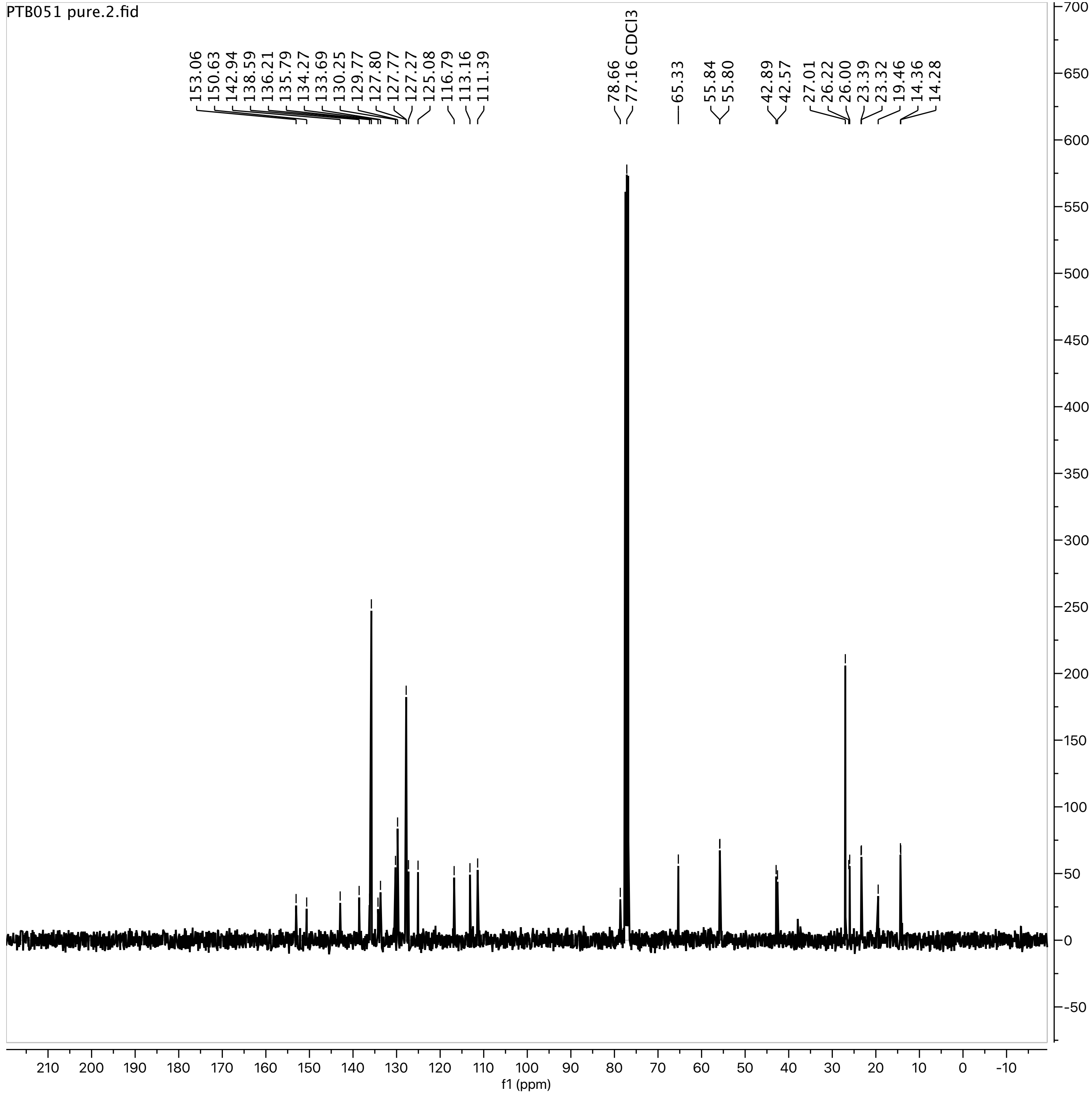

	Parameter	Value
1	Solvent	CDCl3
2	Experiment	1D
3	Spectrometer Frequency	100.62
4	Nucleus	13C

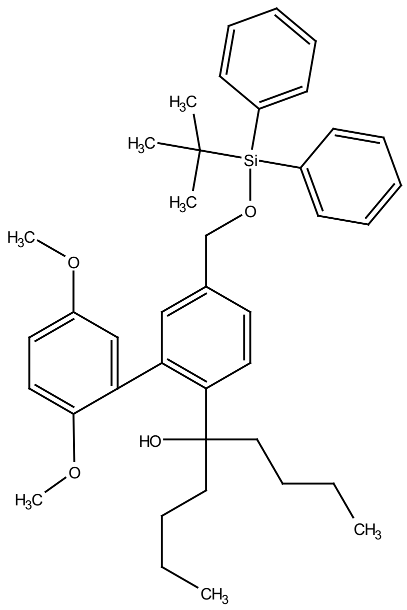

^{13}C NMR (101 MHz, $CDCl_3$) δ 153.06, 150.63, 142.94, 138.59, 136.21, 135.79, 134.27, 133.69, 130.25, 129.77, 127.78 (d, J = 2.9 Hz), 127.27, 125.08, 116.79, 113.16, 111.39, 78.66, 65.33, 55.84, 55.80, 42.89, 42.57, 27.01, 26.22, 26.00, 23.39, 23.32, 19.46, 14.36, 14.28.

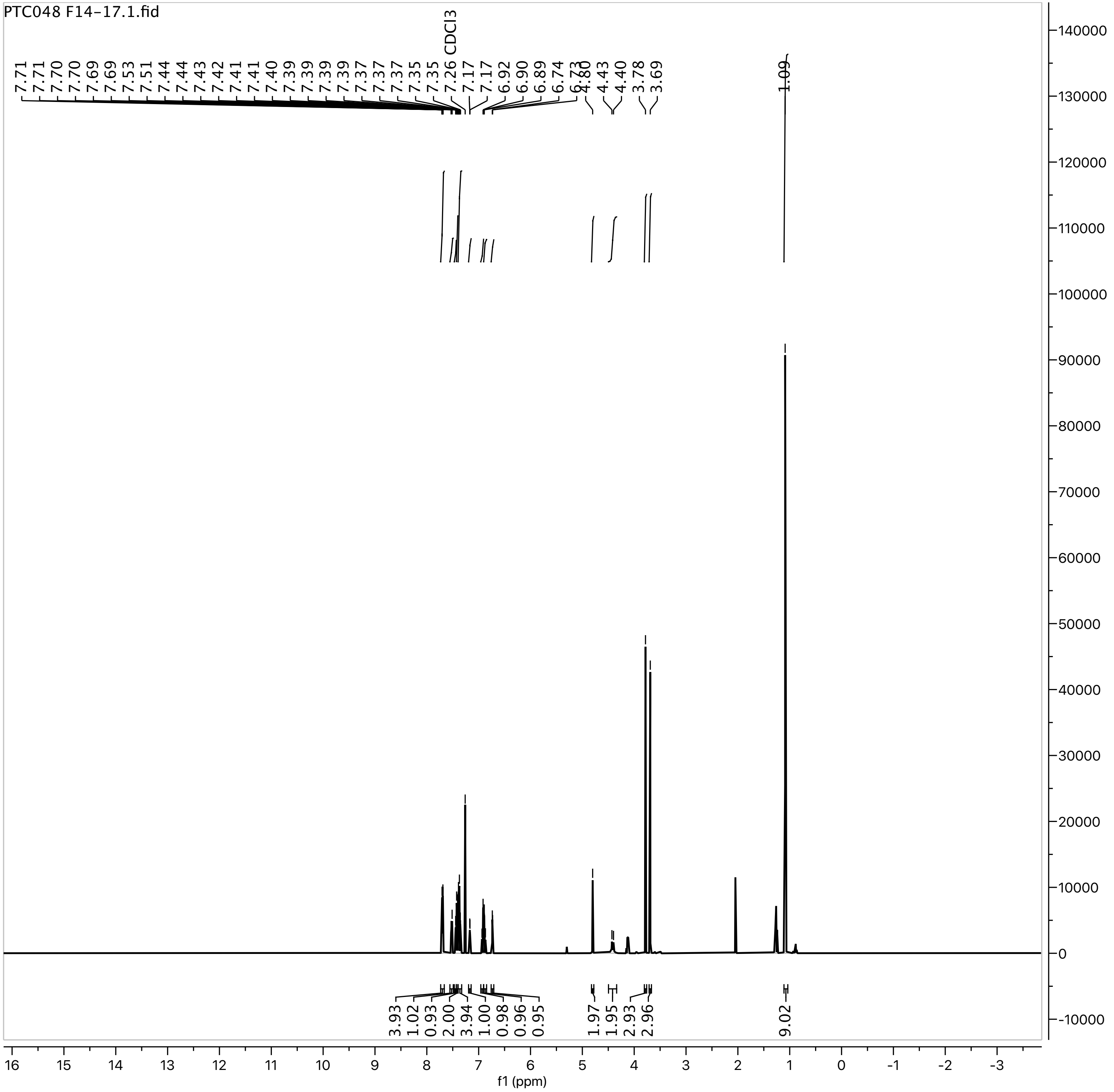

	Parameter	Value
1	Solvent	CDCl3
2	Experiment	1D
3	Spectrometer Frequency	400.13
4	Nucleus	1H

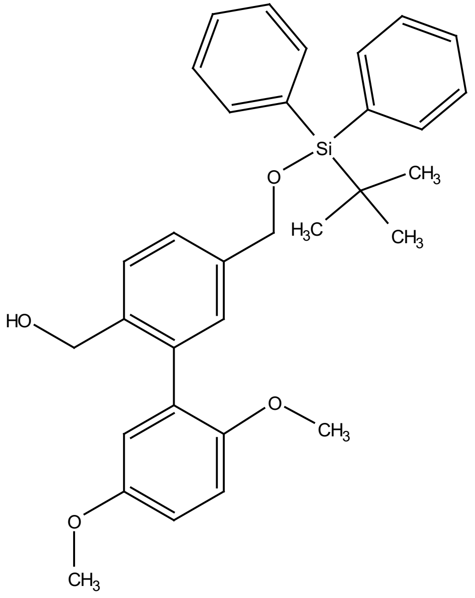

^{1}H NMR (400 MHz, $CDCl_3$) δ 7.73 – 7.66 (m, 4H), 7.52 (d, *J* = 7.9 Hz, 1H), 7.47 – 7.43 (m, 1H), 7.43 – 7.40 (m, 2H), 7.40 – 7.33 (m, 4H), 7.17 (d, *J* = 1.5 Hz, 1H), 6.93 (d, *J* = 8.4 Hz, 1H), 6.88 (dd, *J* = 8.9, 2.9 Hz, 1H), 6.73 (d, *J* = 2.3 Hz, 1H), 4.80 (s, 2H), 4.41 (d, *J* = 12.5 Hz, 2H), 3.78 (s, 3H), 3.69 (s, 3H), 1.09 (s, 9H).

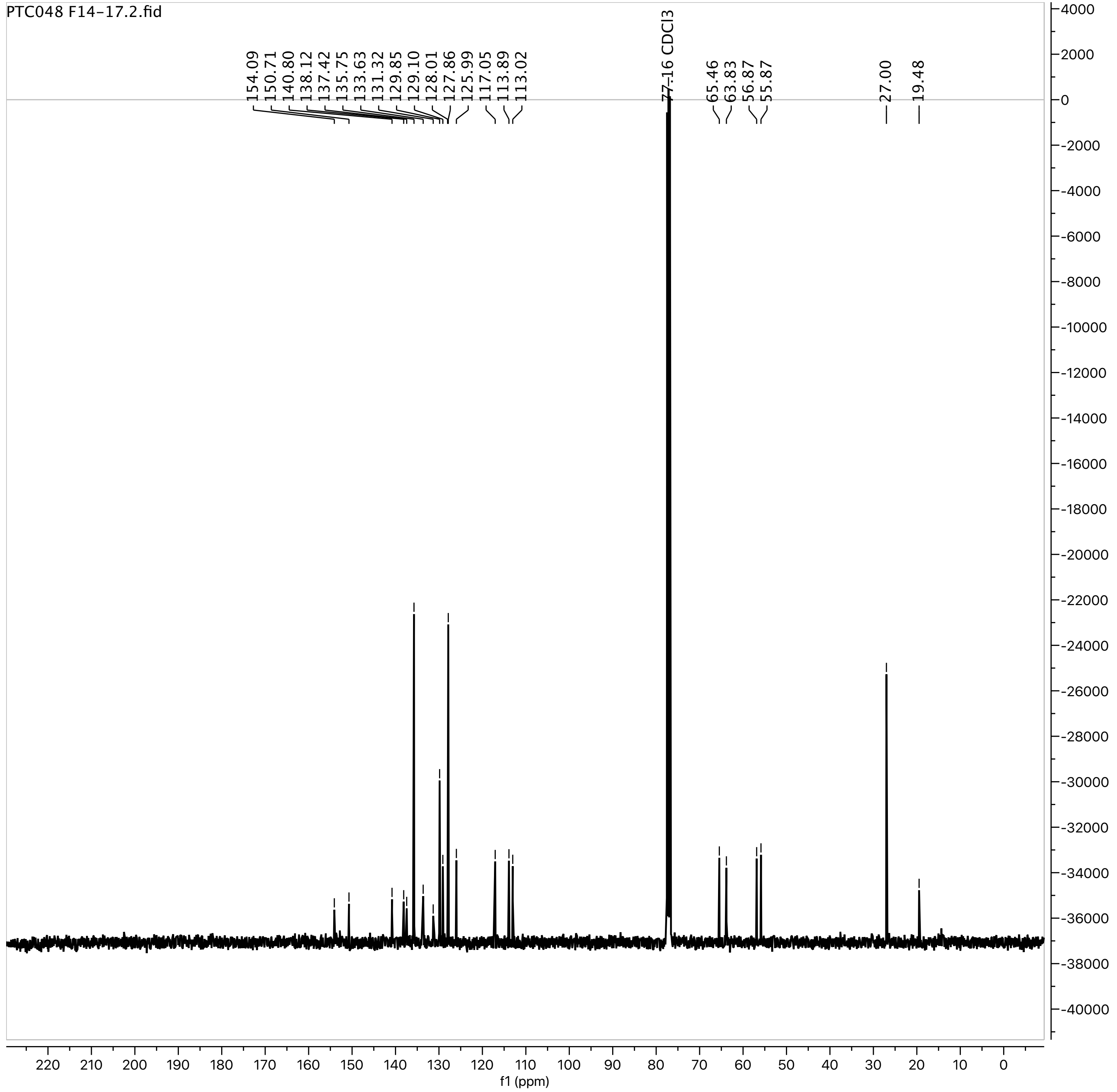

	Parameter	Value
1	Solvent	CDCl3
2	Experiment	1D
3	Spectrometer Frequency	100.62
4	Nucleus	13C

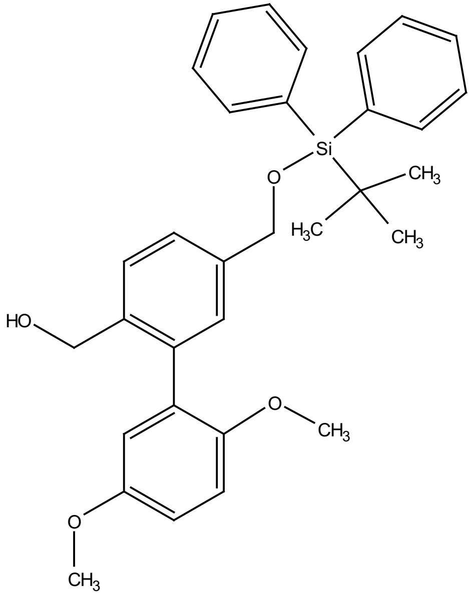

^{13}C NMR (101 MHz, $CDCl_3$) δ 154.09, 150.71, 140.80, 138.12, 137.42, 135.75, 133.63, 131.32, 129.85, 129.10, 128.01, 127.86, 125.99, 117.05, 113.89, 113.02, 65.46, 63.83, 56.87, 55.87, 27.00, 19.48.

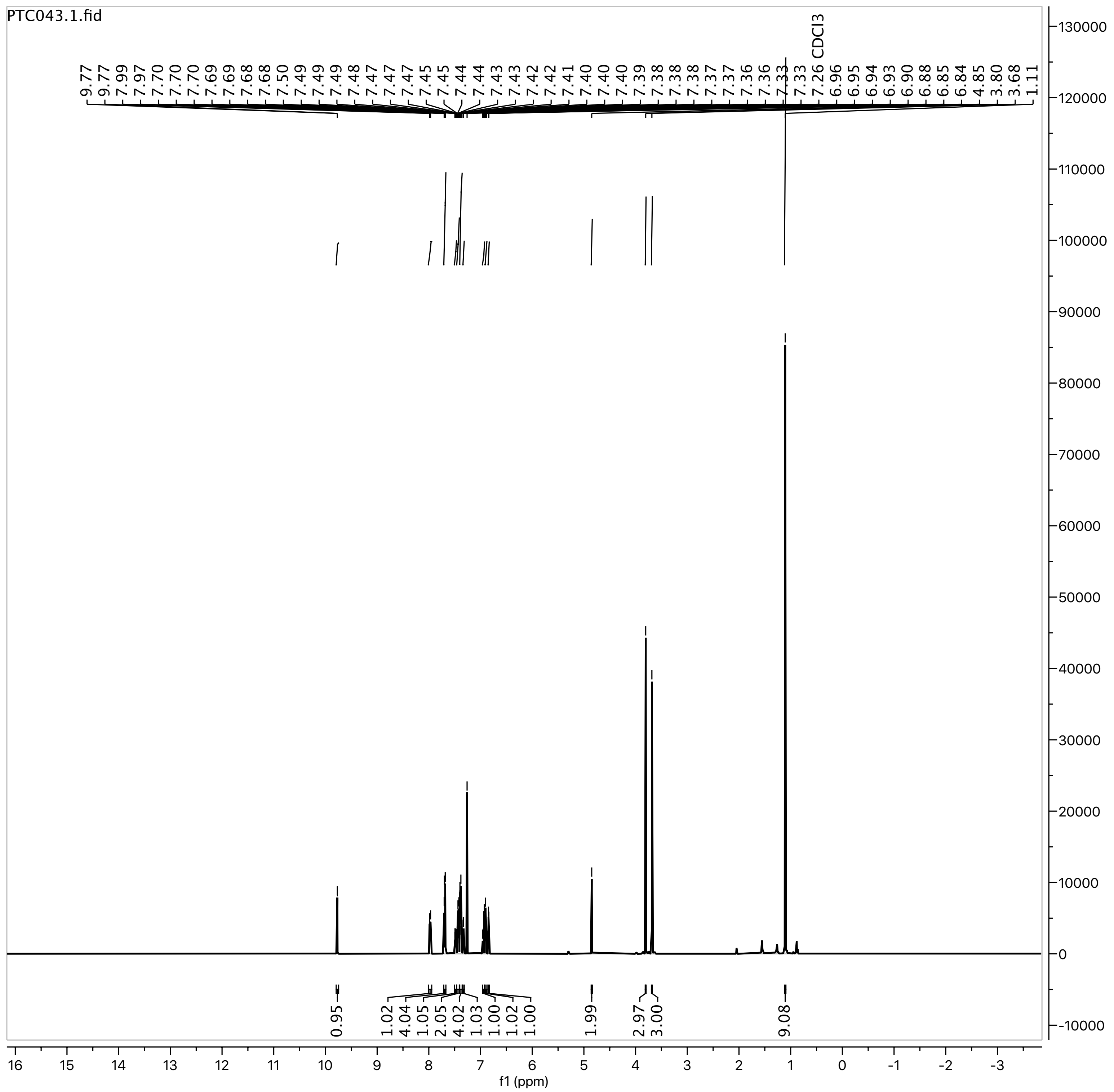

	Parameter	Value
1	Solvent	CDCl3
2	Experiment	1D
3	Spectrometer Frequency	400.13
4	Nucleus	1H

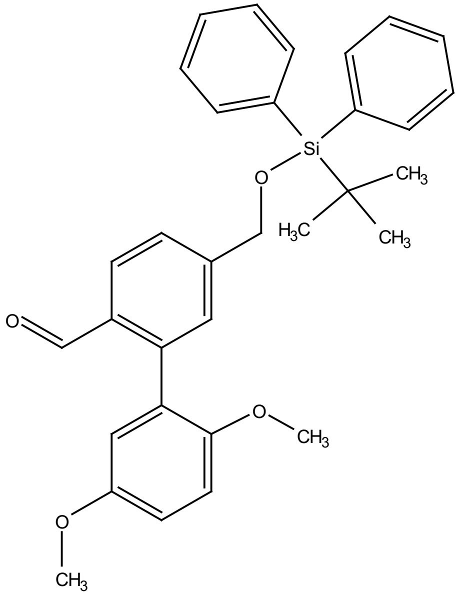

^{1}H NMR (400 MHz, $CDCl_3$) δ 9.77 (d, J = 0.9 Hz, 1H), 7.98 (d, J = 7.9 Hz, 1H), 7.71 – 7.67 (m, 4H), 7.48 (ddd, J = 8.1, 1.7, 0.9 Hz, 1H), 7.46 – 7.41 (m, 2H), 7.40 – 7.36 (m, 4H), 7.33 (d, J = 1.1 Hz, 1H), 6.94 (dd, J = 8.9, 3.1 Hz, 1H), 6.89 (d, J = 8.7 Hz, 1H), 6.84 (d, J = 2.6 Hz, 1H), 4.85 (s, 2H), 3.80 (s, 3H), 3.68 (s, 3H), 1.11 (s, 9H).

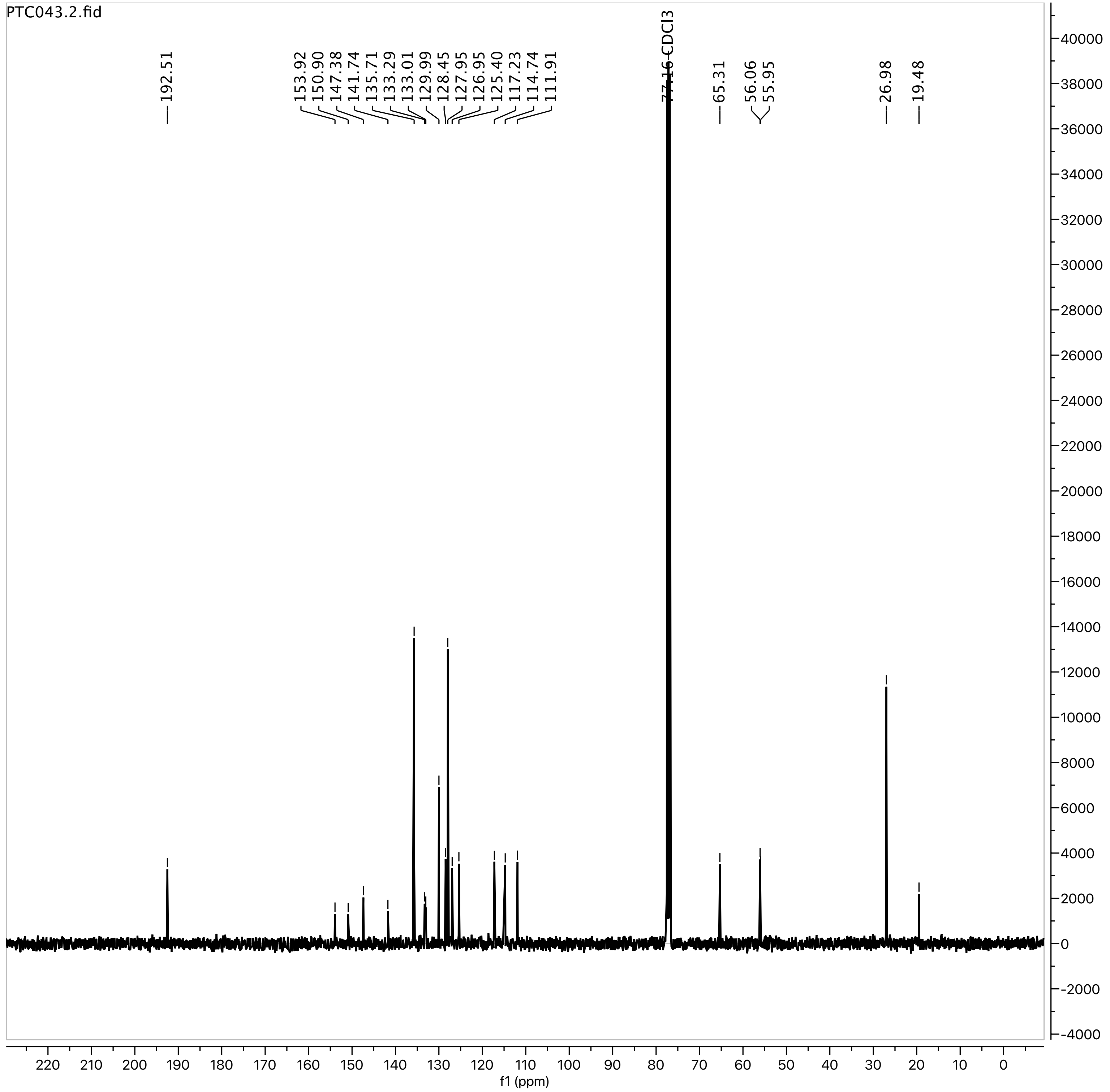

	Parameter	Value
1	Solvent	CDCl3
2	Experiment	1D
3	Spectrometer Frequency	100.62
4	Nucleus	13C

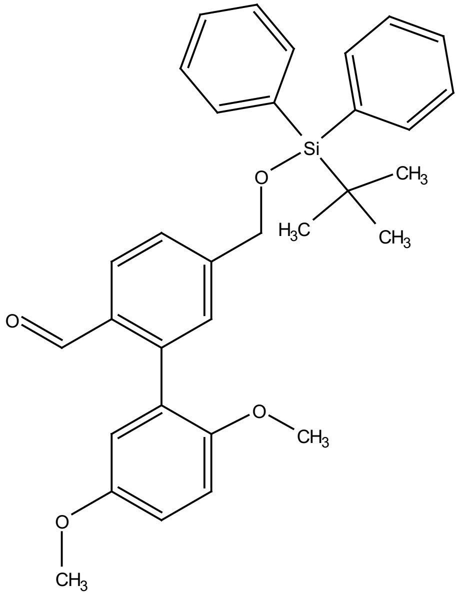

^{13}C NMR (101 MHz, $CDCl_3$) δ 192.51, 153.92, 150.90, 147.38, 141.74, 135.71, 133.29, 133.01, 129.99, 128.45, 127.95, 126.95, 125.40, 117.23, 114.74, 111.91, 65.31, 56.06, 55.95, 26.98, 19.48.

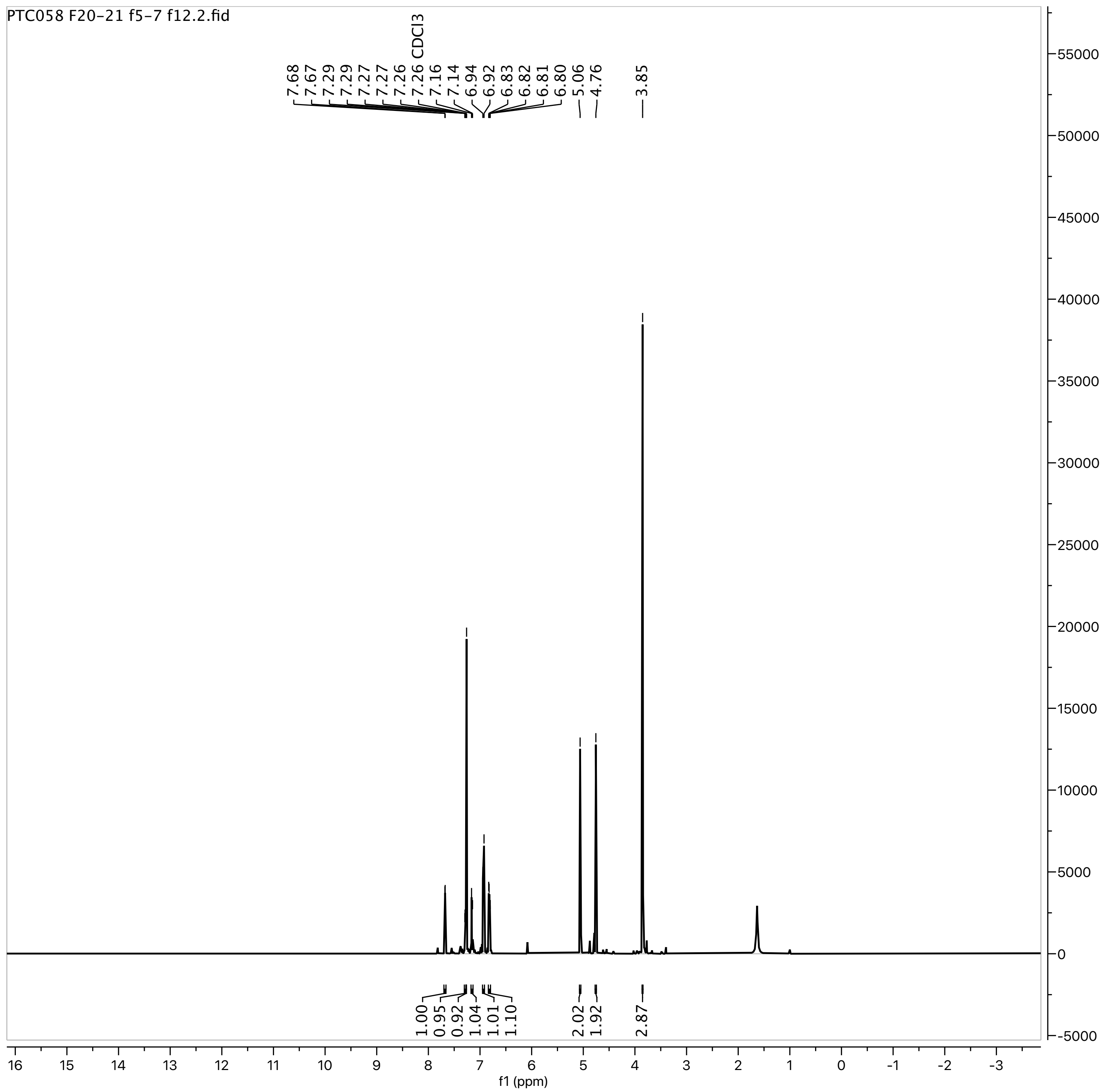

	Parameter	Value
1	Solvent	CDCl3
2	Experiment	1D
3	Spectrometer Frequency	400.13
4	Nucleus	1H

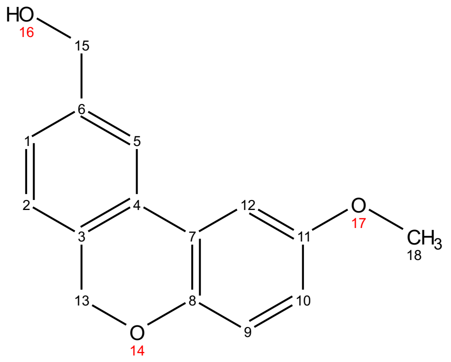

^{1}H NMR (400 MHz, $CDCl_3$) δ 7.68 (d, *J* = 1.8 Hz, 1H), 7.30 – 7.27 (m, 1H), 7.27 (d, *J* = 2.8 Hz, 1H), 7.15 (d, *J* = 7.7 Hz, 1H), 6.93 (d, *J* = 8.8 Hz, 1H), 6.82 (dd, *J* = 8.8, 2.9 Hz, 1H), 5.06 (s, 2H), 4.76 (s, 2H), 3.85 (s, 3H).

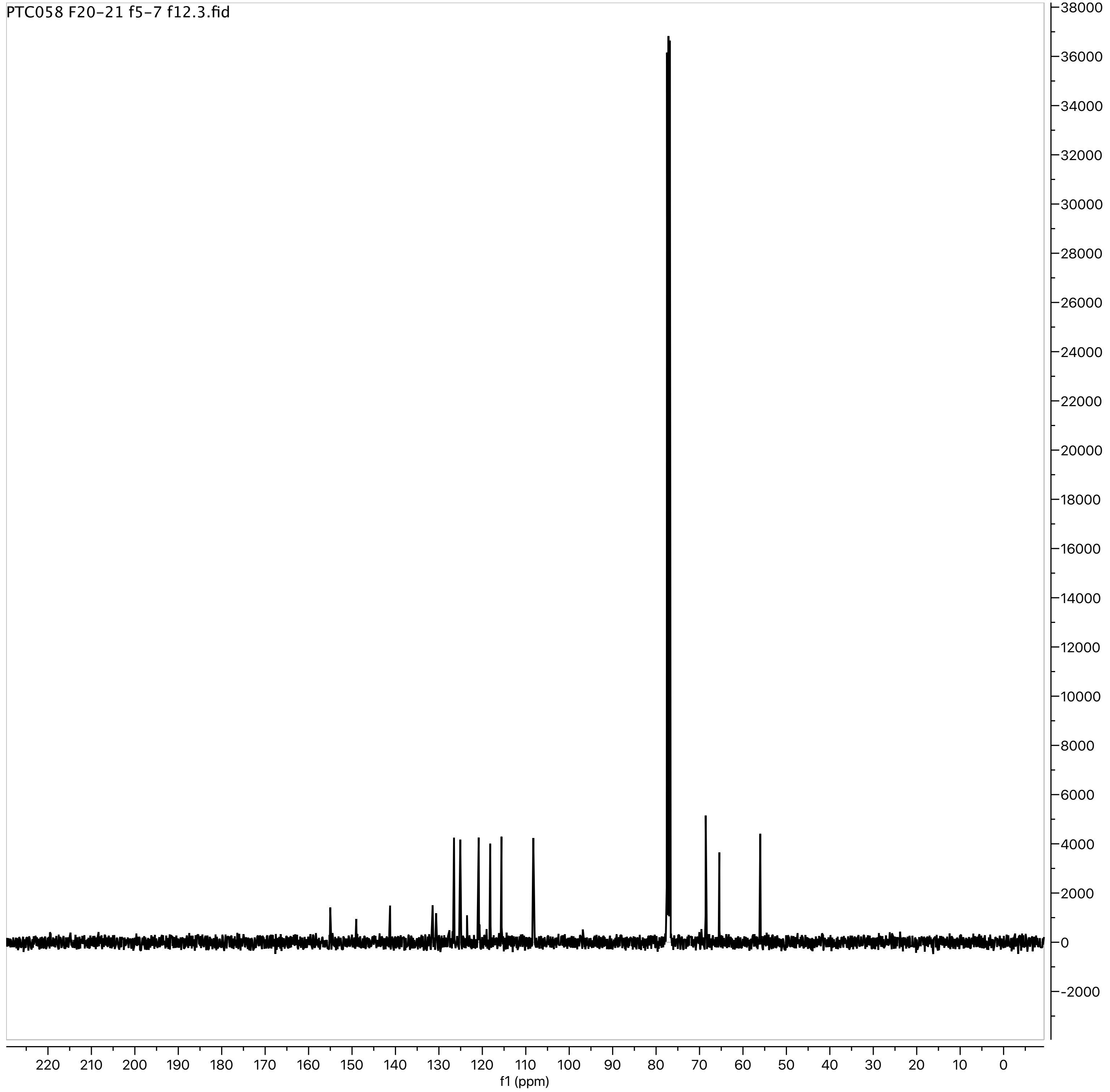

	Parameter	Value
1	Solvent	CDCl3
2	Experiment	1D
3	Spectrometer Frequency	100.62
4	Nucleus	13C

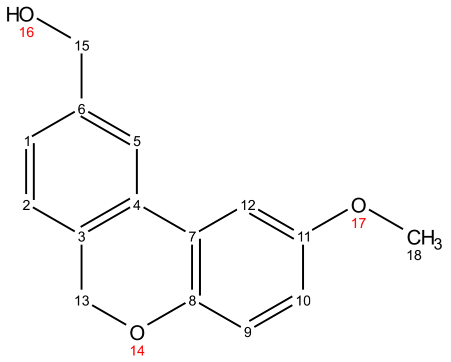

^{13}C NMR (101 MHz, $CDCl_3$) δ 155.01, 149.03, 141.24, 131.44, 130.65, 126.52, 125.08, 123.53, 120.84, 118.19, 115.59, 108.27, 68.58, 65.44, 56.02.

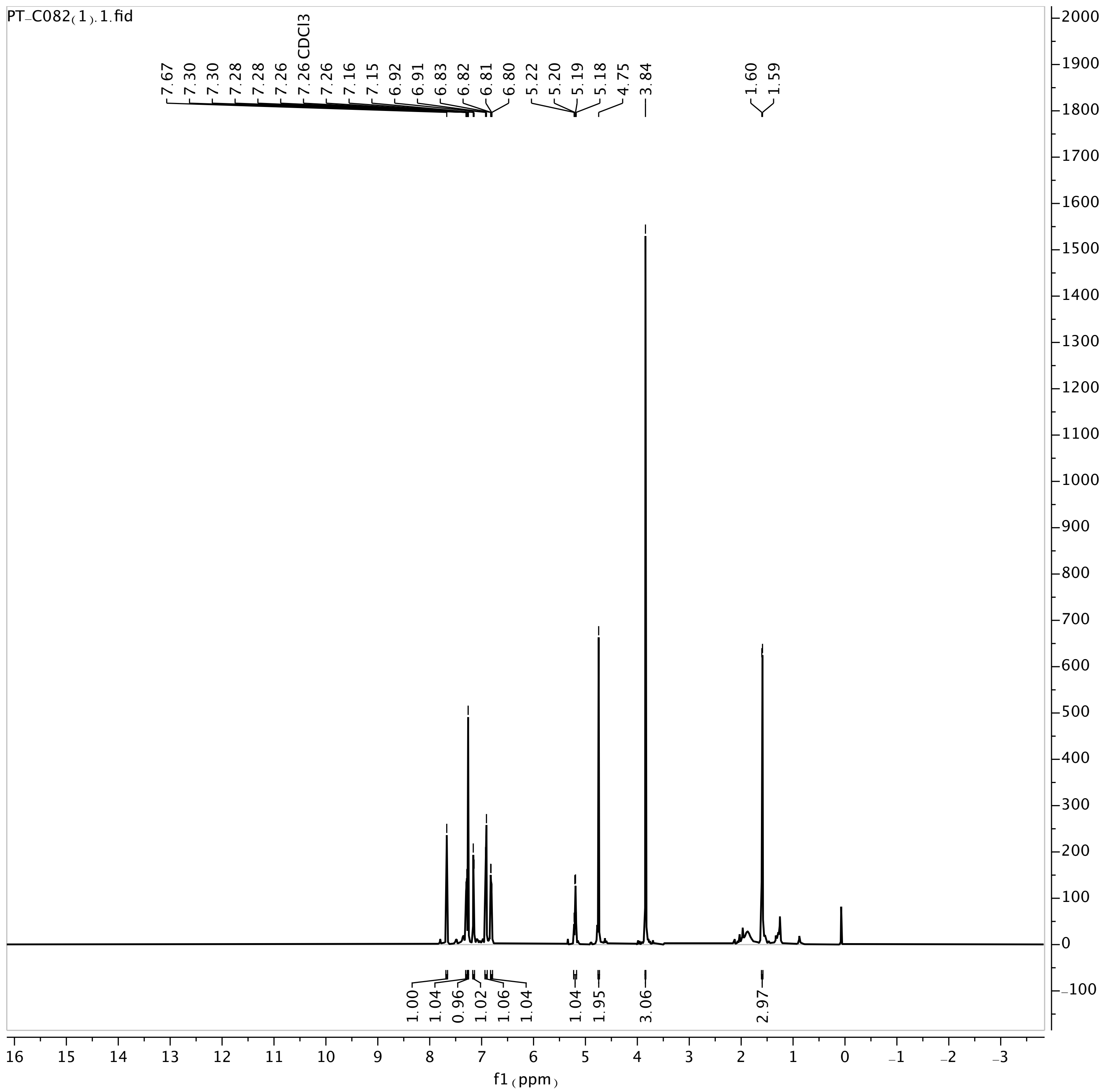

	Parameter	Value
1	Solvent	CDCl3
2	Experiment	1D
3	Spectrometer Frequency	500.17
4	Nucleus	1H

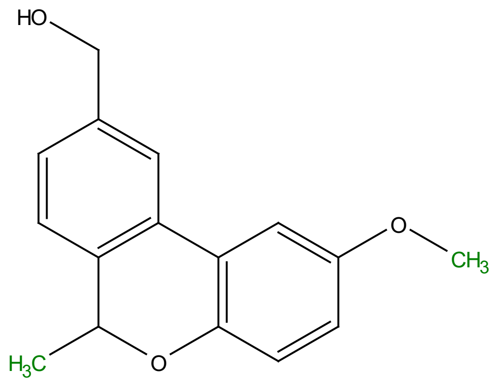

^{1}H NMR (500 MHz, $CDCl_3$) δ 7.67 (s, 1H), 7.29 (dd, *J* = 7.7, 1.7 Hz, 1H), 7.26 (d, *J* = 2.1 Hz, 1H), 7.15 (d, *J* = 7.7 Hz, 1H), 6.92 (d, *J* = 8.7 Hz, 1H), 6.81 (dd, *J* = 8.8, 2.9 Hz, 1H), 5.20 (q, *J* = 6.6 Hz, 1H), 4.75 (s, 2H), 3.84 (s, 3H), 1.60 (d, *J* = 6.6 Hz, 3H).

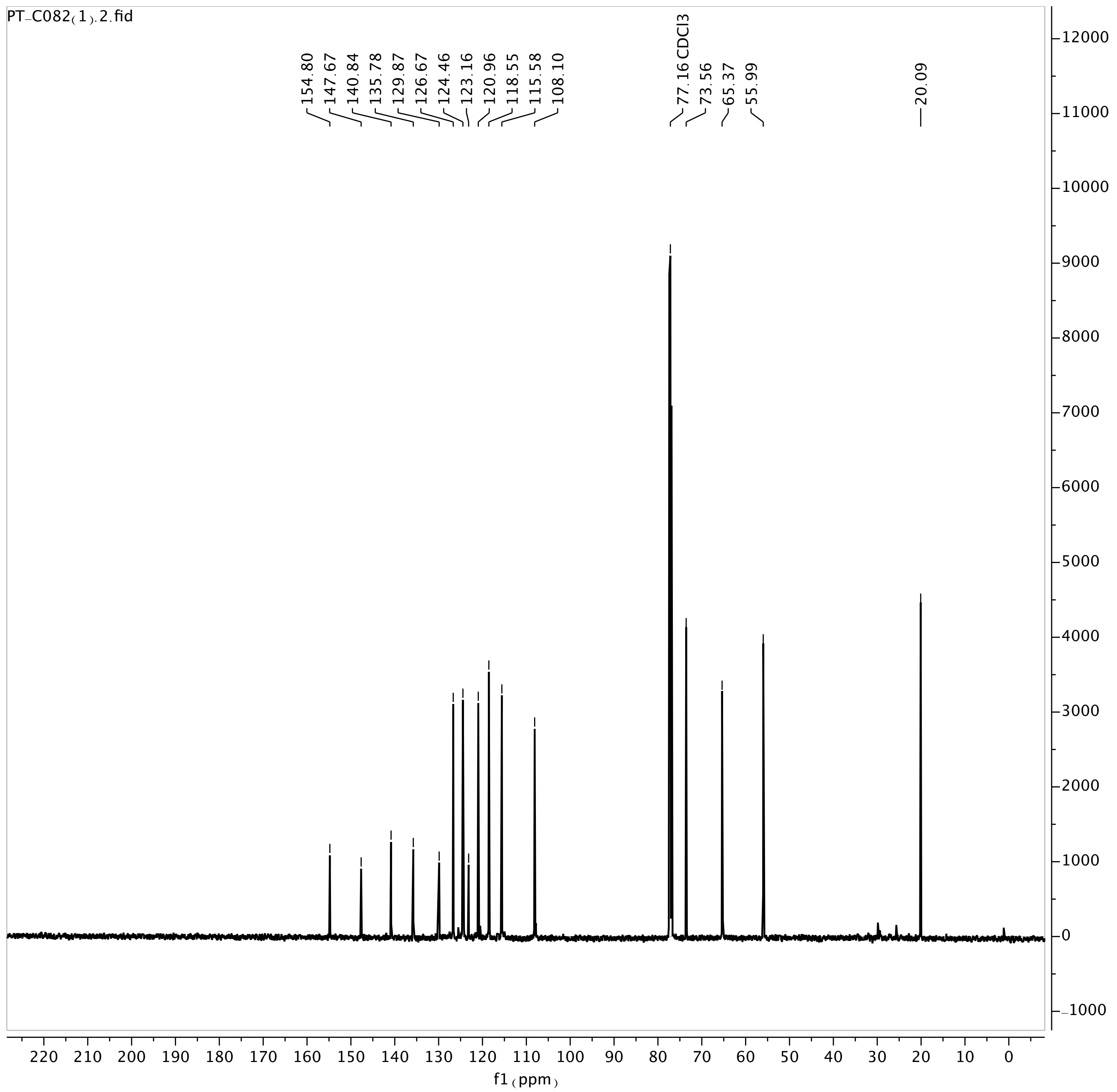

Parameter		Value
1	Solvent	CDCl3
2	Experiment	1D
3	Spectrometer Frequency	125.78
4	Nucleus	13C

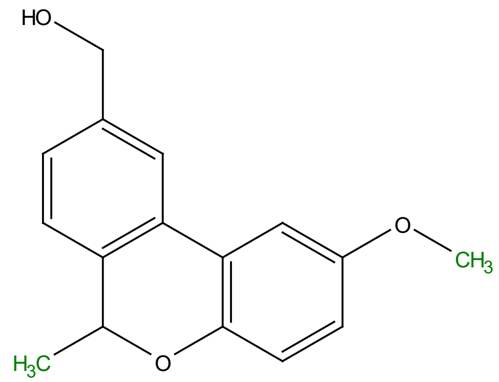

^{13}C NMR (126 MHz, $CDCl_3$) δ 154.80, 147.67, 140.84, 135.78, 129.87, 126.67, 124.46, 123.16, 120.96, 118.55, 115.58, 108.10, 73.56, 65.37, 55.99, 20.09.

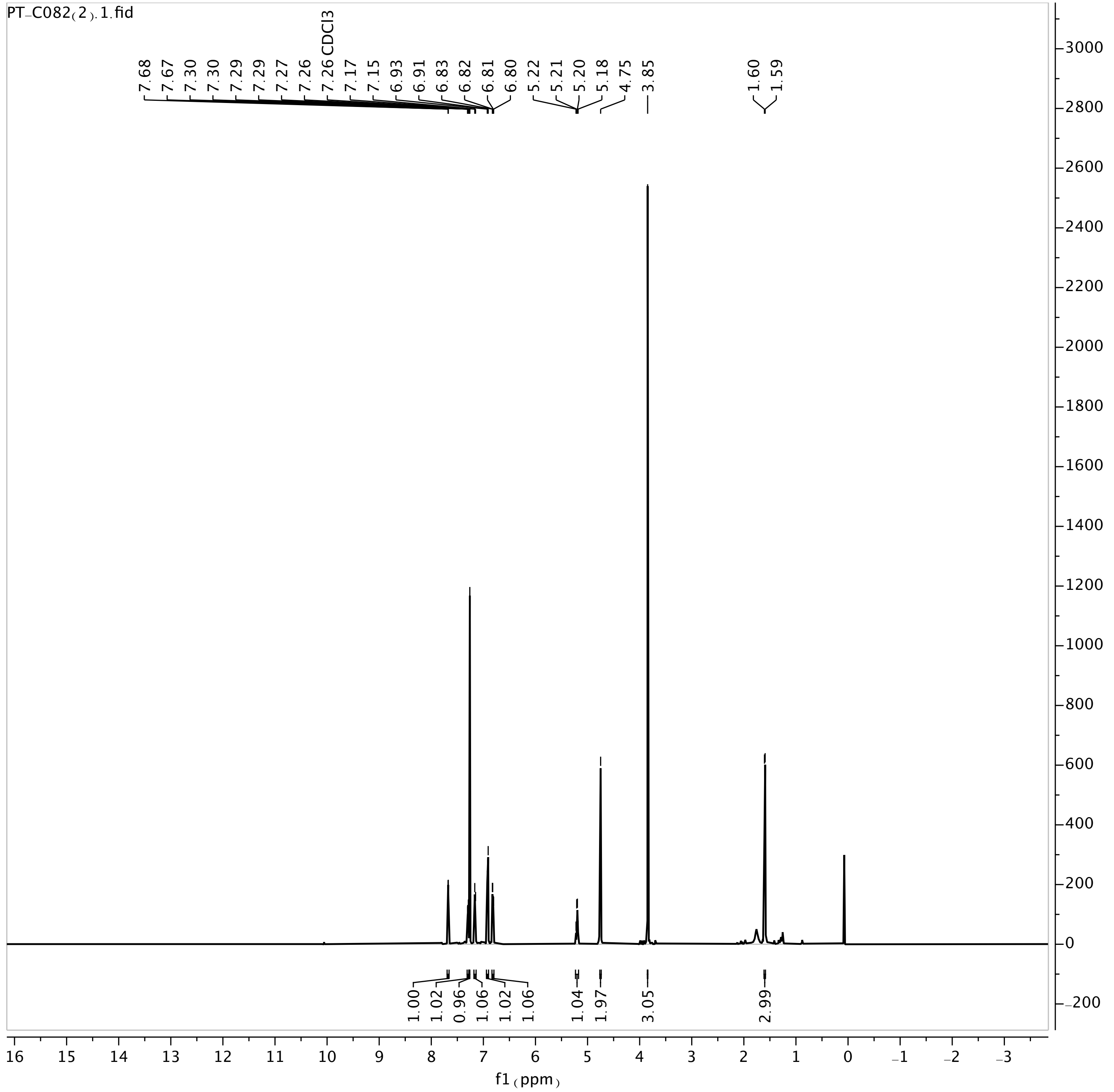

	Parameter	Value
1	Solvent	CDCl3
2	Experiment	1D
3	Spectrometer Frequency	500.17
4	Nucleus	1H

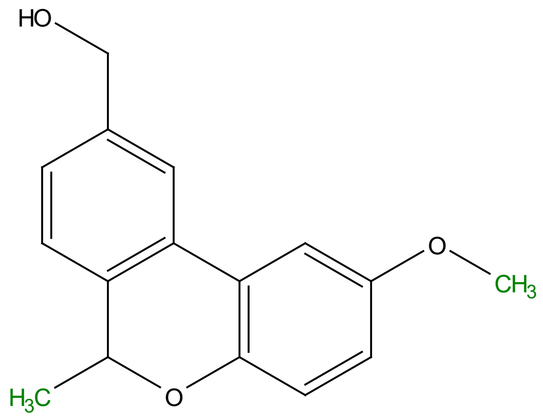

^{1}H NMR (500 MHz, $CDCl_3$) δ 7.68 (d, *J* = 2.0 Hz, 1H), 7.29 (dd, *J* = 7.8, 1.7 Hz, 1H), 7.26 (d, *J* = 2.8 Hz, 1H), 7.16 (d, *J* = 7.7 Hz, 1H), 6.92 (d, *J* = 8.8 Hz, 1H), 6.82 (dd, *J* = 8.8, 3.0 Hz, 1H), 5.20 (q, *J* = 6.6 Hz, 1H), 4.75 (s, 2H), 3.85 (s, 3H), 1.60 (d, *J* = 6.5 Hz, 3H).

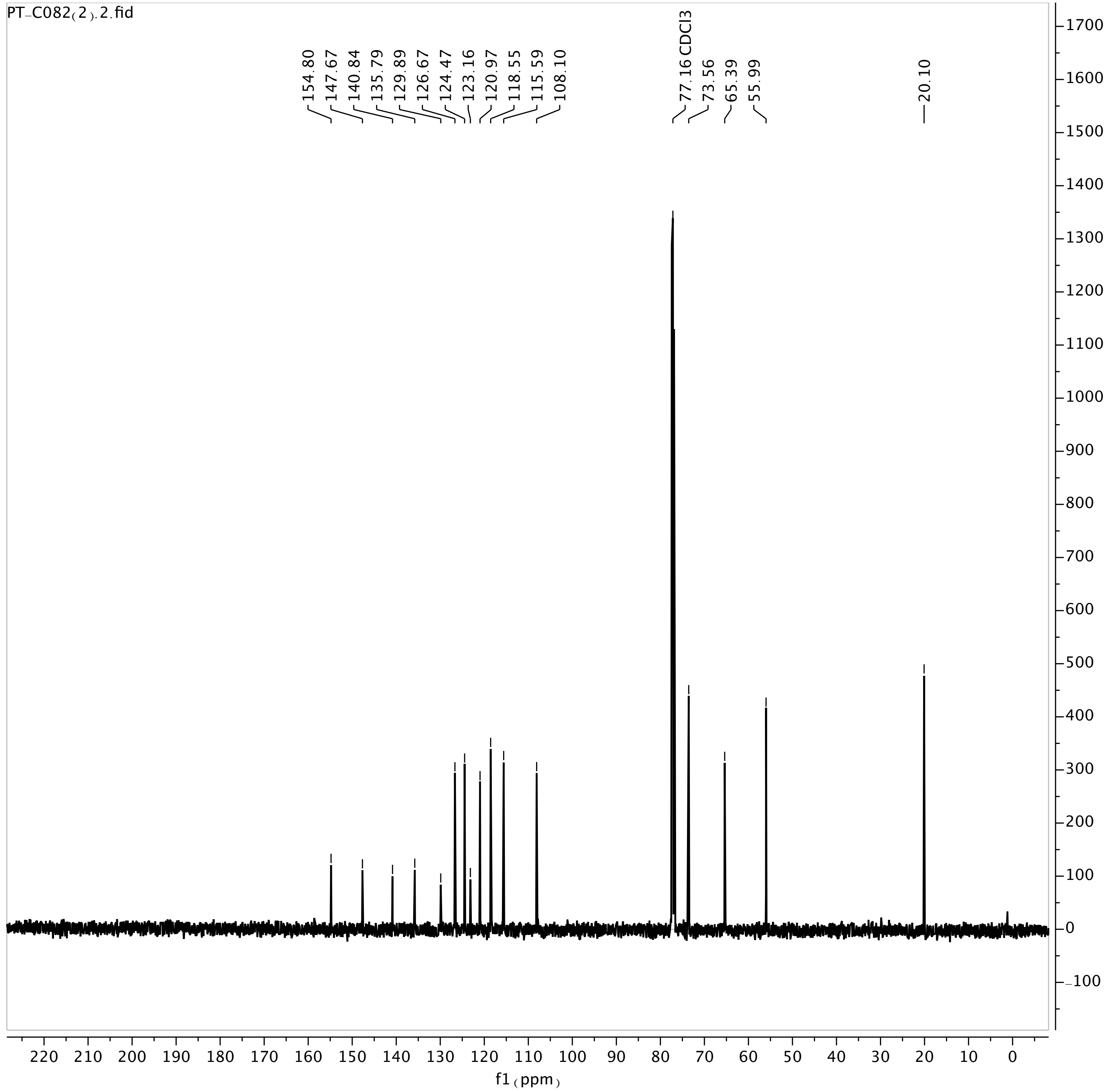

	Parameter	Value
1	Solvent	CDCl3
2	Experiment	1D
3	Spectrometer Frequency	125.78
4	Nucleus	13C

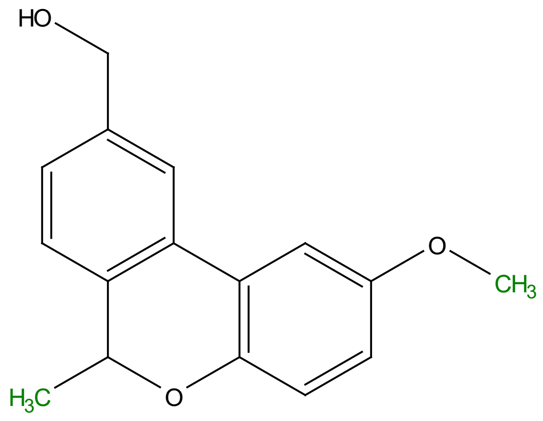

^{13}C NMR (126 MHz, $CDCl_3$) δ 154.80, 147.67, 140.84, 135.79, 129.89, 126.67, 124.47, 123.16, 120.97, 118.55, 115.59, 108.10, 73.56, 65.39, 55.99, 20.10.

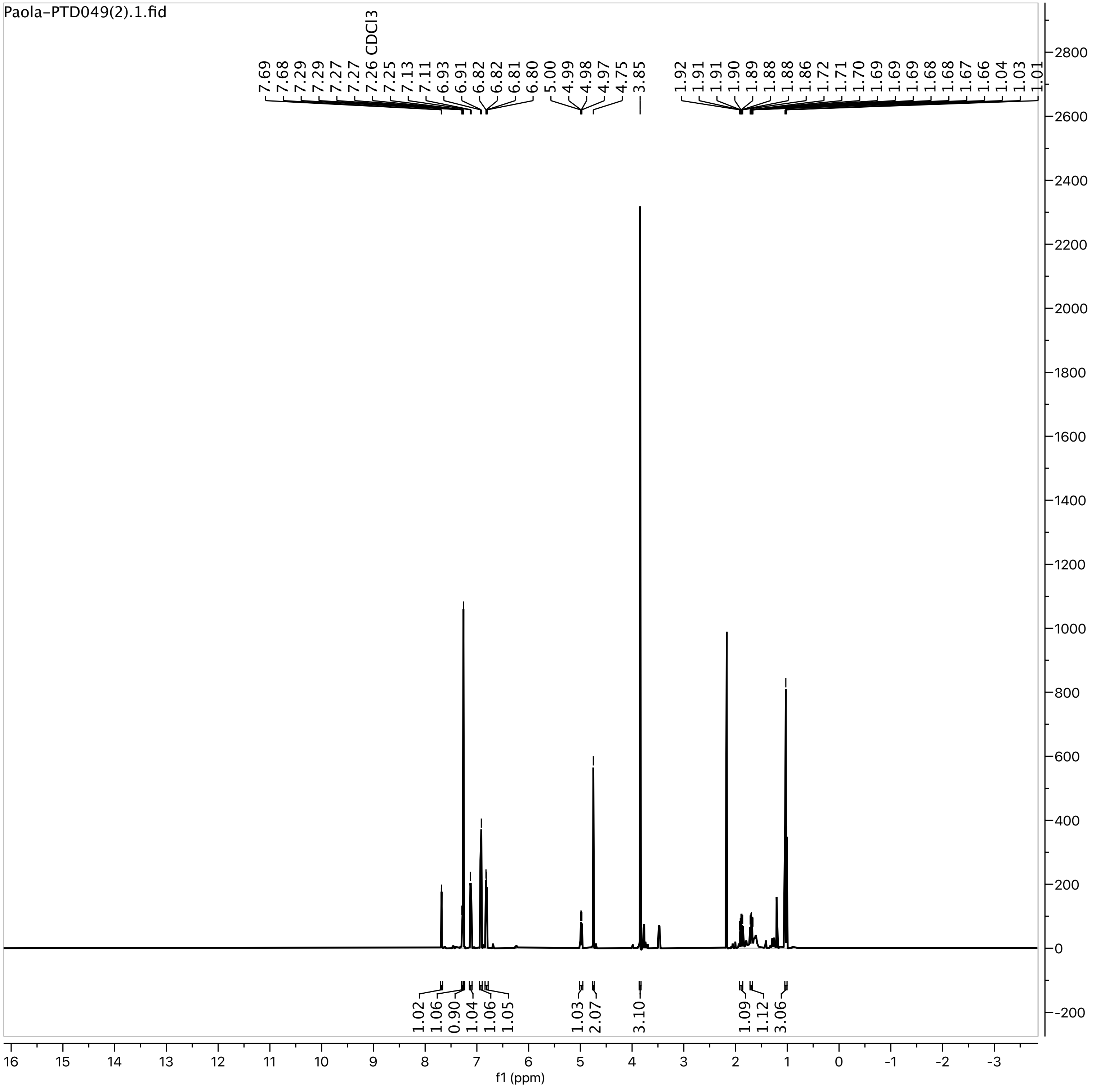

	Parameter	Value
1	Solvent	CDCl3
2	Experiment	1D
3	Spectrometer Frequency	500.17
4	Nucleus	1H

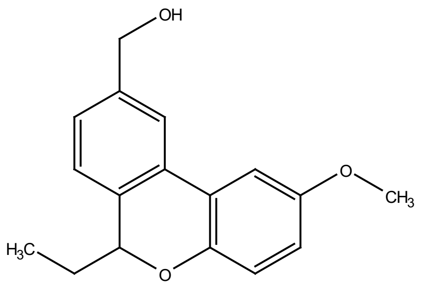

^{1}H NMR (500 MHz, $CDCl_3$) δ 7.68 (d, J = 2.1 Hz, 1H), 7.28 (dd, J = 7.7, 1.7 Hz, 1H), 7.25 (s, 1H), 7.12 (d, J = 7.7 Hz, 1H), 6.92 (d, J = 8.8 Hz, 1H), 6.81 (dd, J = 8.8, 3.0 Hz, 1H), 4.98 (dd, J = 8.8, 4.9 Hz, 1H), 4.75 (s, 2H), 3.85 (s, 3H), 1.89 (dtd, J = 14.2, 7.3, 1.6 Hz, 1H), 1.72 – 1.68 (m, 1H), 1.03 (s, 3H).

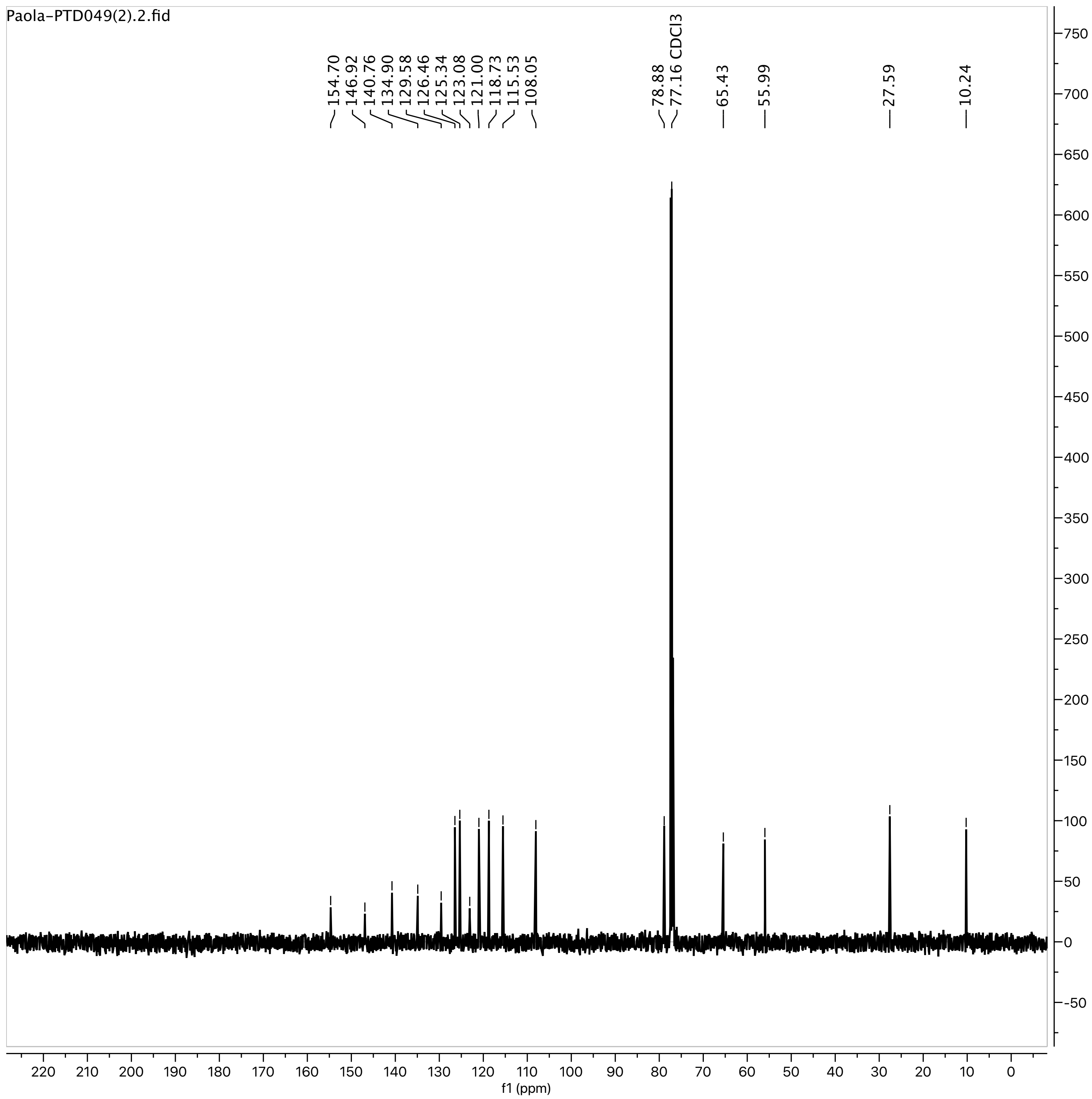

	Parameter	Value
1	Solvent	CDCl3
2	Experiment	1D
3	Spectrometer Frequency	125.78
4	Nucleus	13C

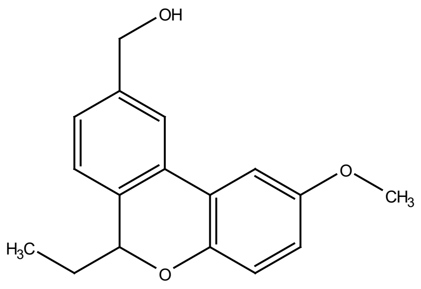

^{13}C NMR (126 MHz, $CDCl_3$) δ 154.70, 146.92, 140.76, 134.90, 129.58, 126.46, 125.34, 123.08, 121.00, 118.73, 115.53, 108.05, 78.88, 65.43, 55.99, 27.59, 10.24.

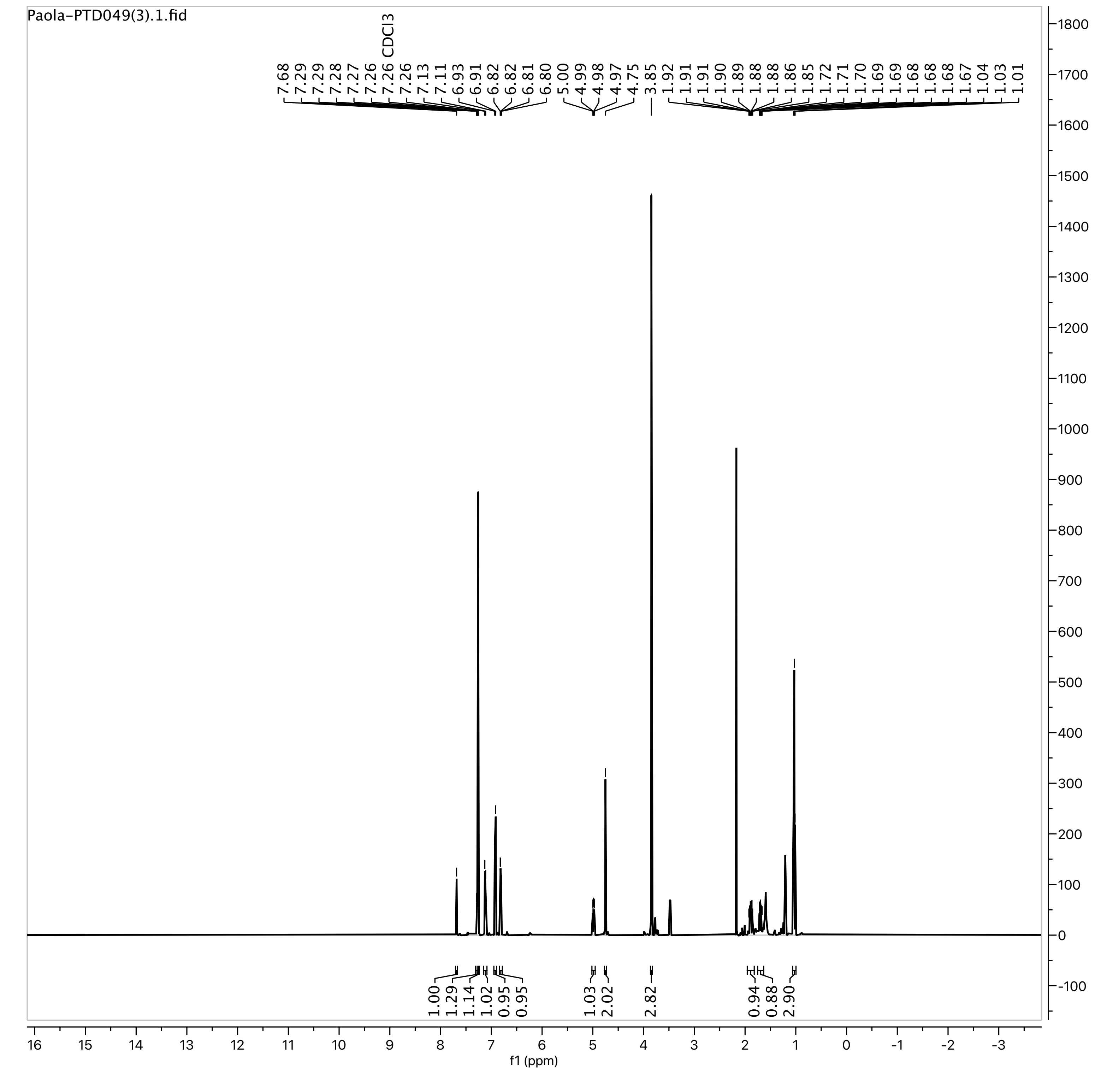

	Parameter	Value
1	Solvent	CDCl3
2	Experiment	1D
3	Spectrometer Frequency	500.17
4	Nucleus	1H

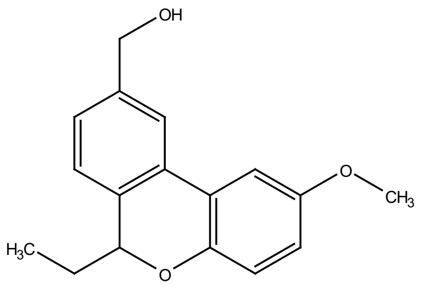

^{1}H NMR (500 MHz, $CDCl_3$) δ 7.68 (s, 1H), 7.28 (dd, J = 7.7, 1.7 Hz, 1H), 7.26 (d, J = 2.8 Hz, 1H), 7.12 (d, J = 7.7 Hz, 1H), 6.92 (d, J = 8.7 Hz, 1H), 6.81 (dd, J = 8.8, 2.9 Hz, 1H), 4.99 (dd, J = 8.9, 4.9 Hz, 1H), 4.75 (s, 2H), 3.85 (s, 3H), 1.96 – 1.82 (m, 1H), 1.69 (ddd, J = 14.2, 7.4, 5.0 Hz, 1H), 1.03 (t, J = 7.4 Hz, 3H).

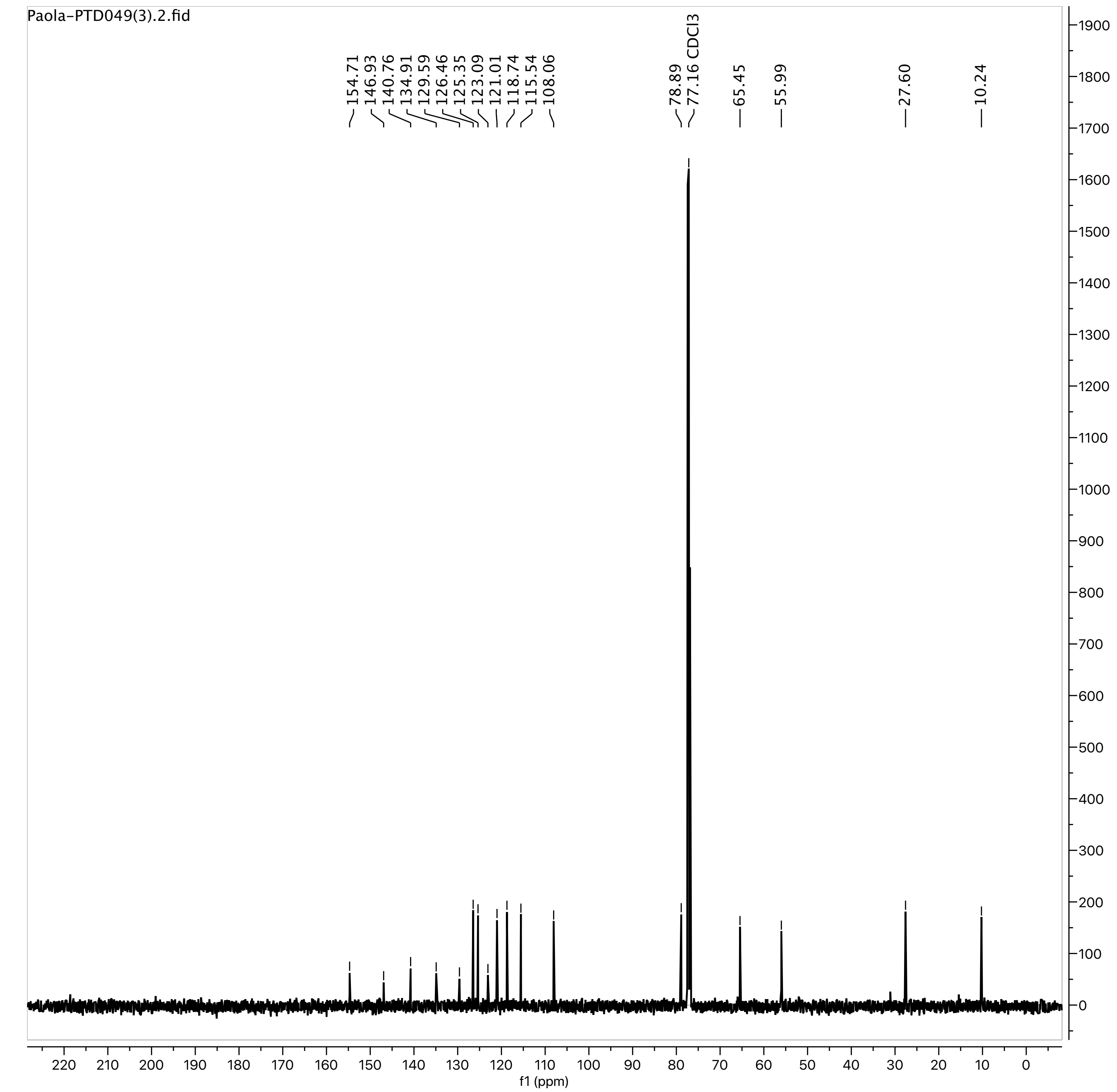

	Parameter	Value
1	Solvent	CDCl3
2	Experiment	1D
3	Spectrometer Frequency	125.78
4	Nucleus	13C

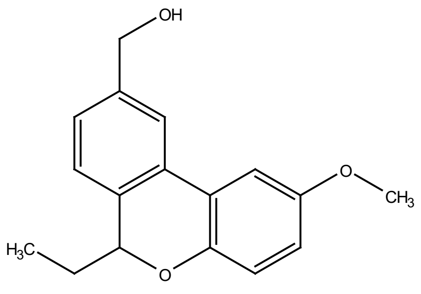

^{13}C NMR (126 MHz, $CDCl_3$) δ 154.71, 146.93, 140.76, 134.91, 129.59, 126.46, 125.35, 123.09, 121.01, 118.74, 115.54, 108.06, 78.89, 65.45, 55.99, 27.60, 10.24.

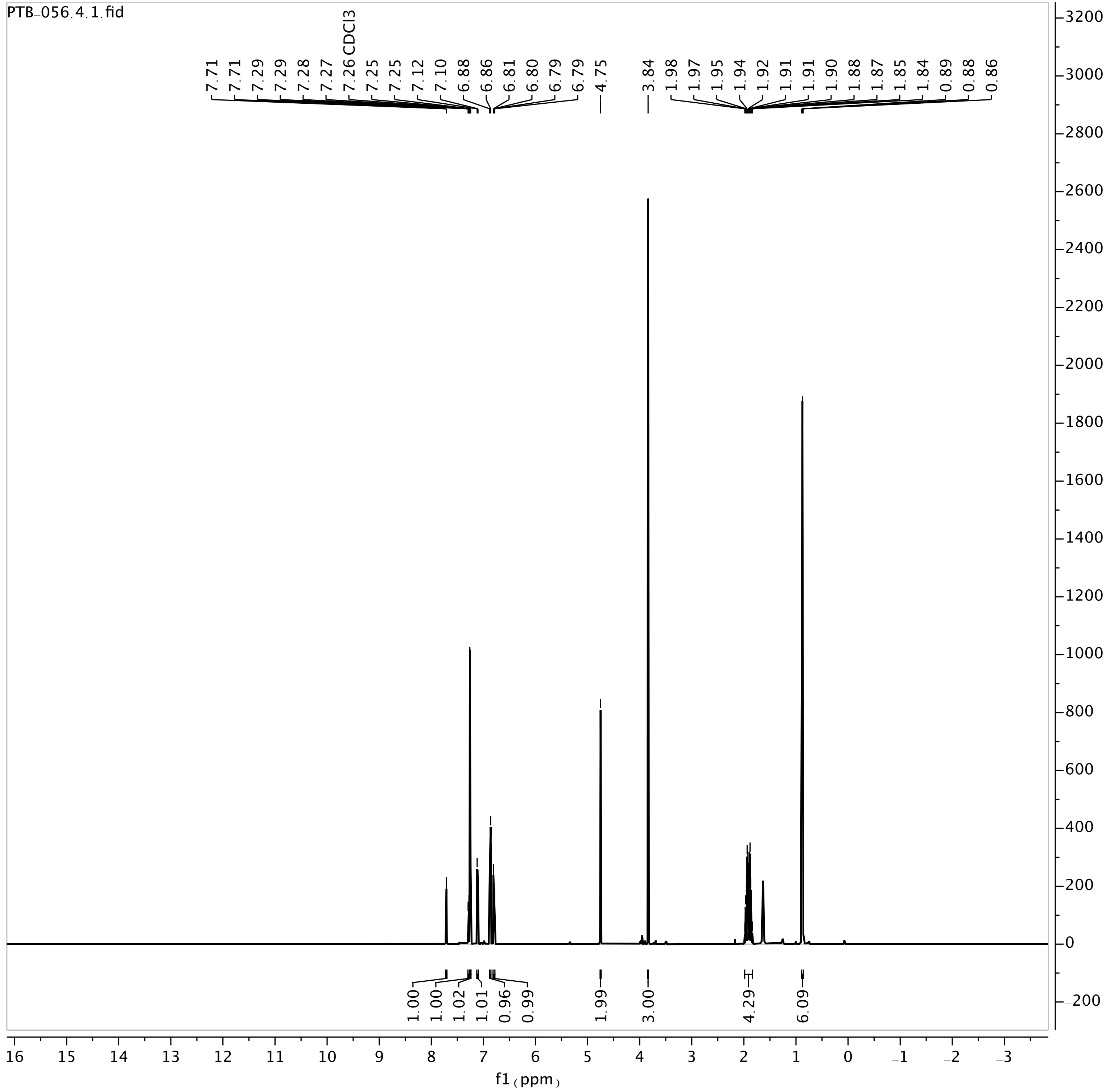

	Parameter	Value
1	Solvent	CDCl3
2	Experiment	1D
3	Spectrometer Frequency	500.17
4	Nucleus	1H

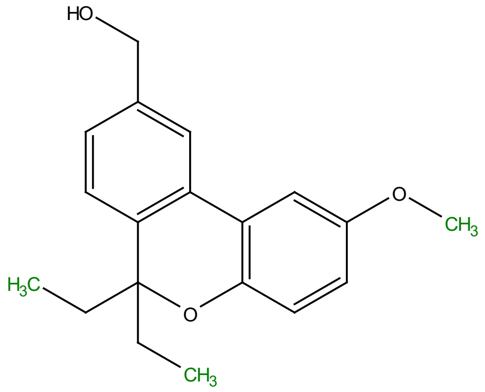

^{1}H NMR (500 MHz, $CDCl_3$) δ 7.71 (d, J = 1.8 Hz, 1H), 7.28 (dd, J = 7.9, 1.8 Hz, 1H), 7.25 (d, J = 2.9 Hz, 1H), 7.11 (d, J = 7.9 Hz, 1H), 6.87 (d, J = 8.7 Hz, 1H), 6.80 (dd, J = 8.7, 2.9 Hz, 1H), 4.75 (s, 2H), 3.84 (s, 3H), 1.98 – 1.84 (m, 4H), 0.88 (t, J = 7.4 Hz, 6H).

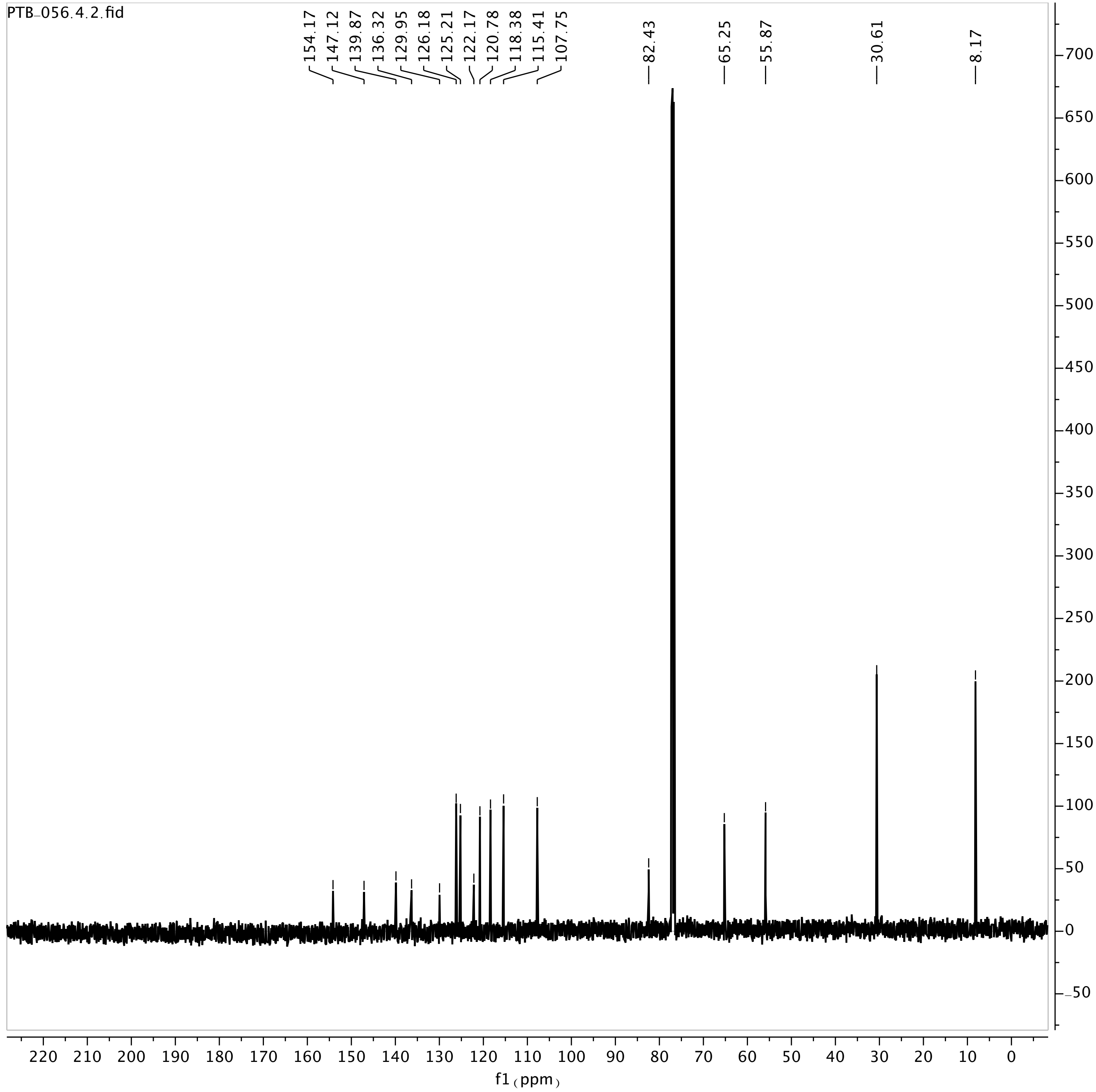

	Parameter	Value
1	Solvent	CDCl3
2	Experiment	1D
3	Spectrometer Frequency	125.78
4	Nucleus	13C

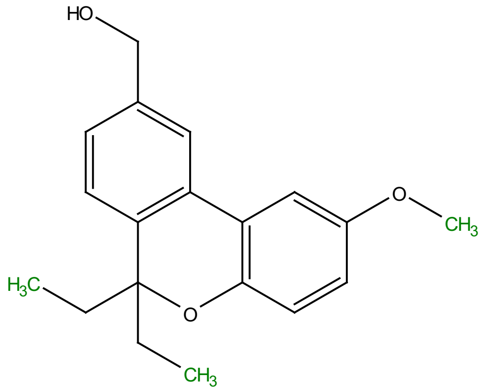

^{13}C NMR (126 MHz, $CDCl_3$) δ 154.17, 147.12, 139.87, 136.32, 129.95, 126.18, 125.21, 122.17, 120.78, 118.38, 115.41, 107.75, 82.43, 65.25, 55.87, 30.61, 8.17.

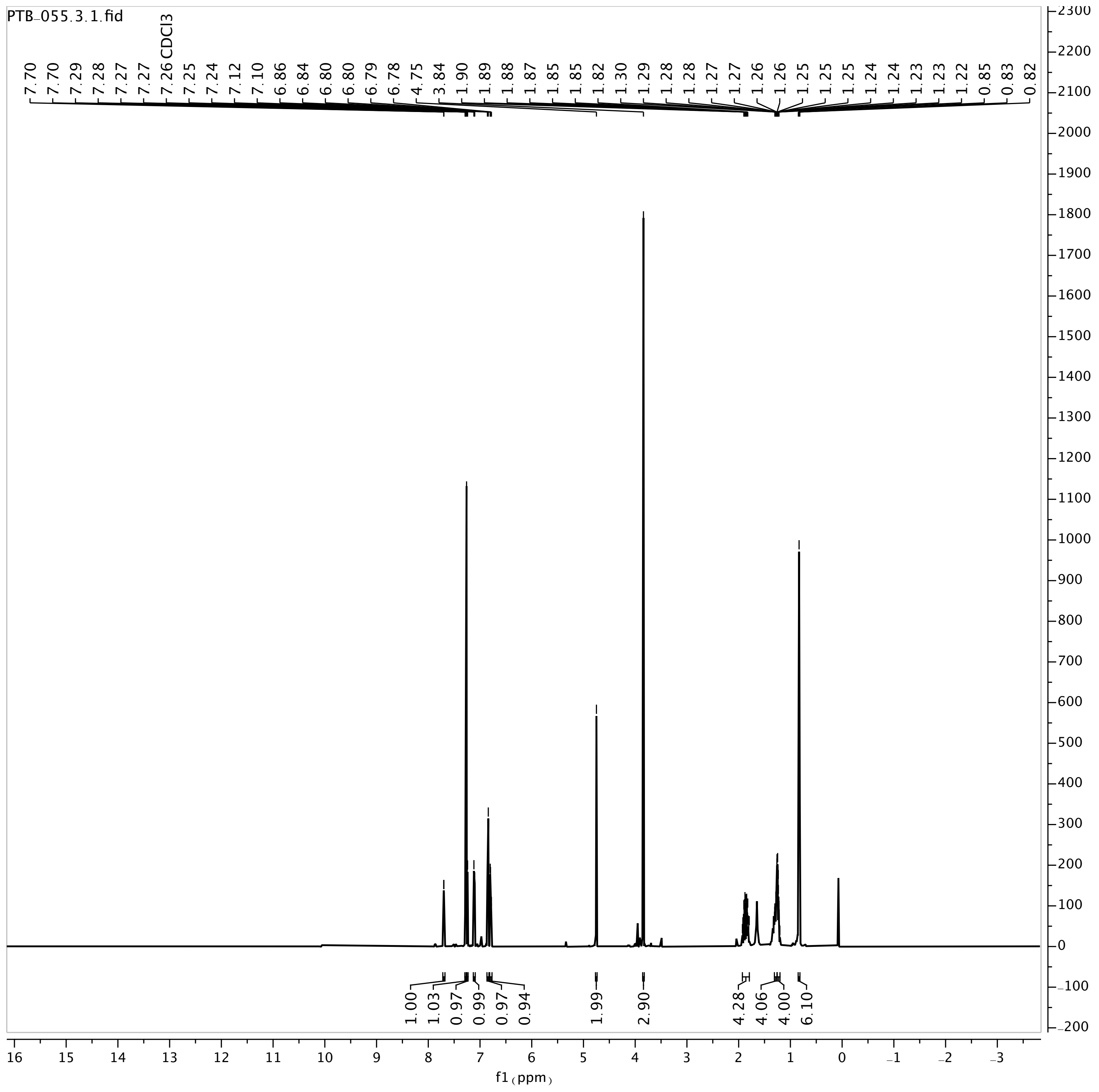

	Parameter	Value
1	Solvent	CDCl3
2	Experiment	1D
3	Spectrometer Frequency	500.17
4	Nucleus	1H

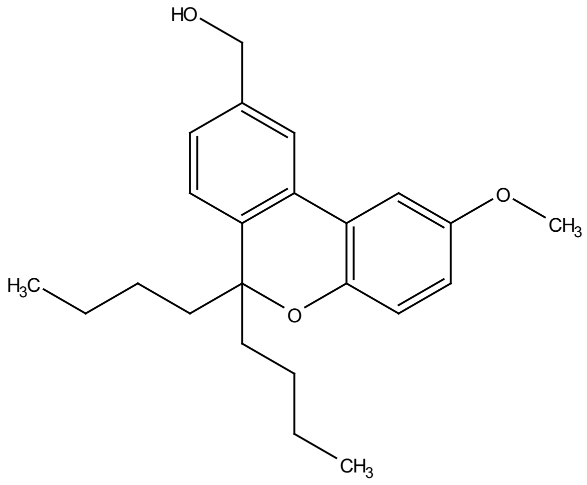

^{1}H NMR (500 MHz, $CDCl_3$) δ 7.70 (d, *J* = 1.7 Hz, 1H), 7.28 (dd, *J* = 7.9, 1.8 Hz, 1H), 7.24 (d, *J* = 2.9 Hz, 1H), 7.11 (d, *J* = 7.9 Hz, 1H), 6.85 (d, *J* = 8.8 Hz, 1H), 6.79 (dd, *J* = 8.8, 2.9 Hz, 1H), 4.75 (s, 2H), 3.84 (s, 3H), 1.93 – 1.79 (m, 4H), 1.31 – 1.26 (m, 4H), 1.26 – 1.20 (m, 4H), 0.83 (t, *J* = 7.1 Hz, 6H).

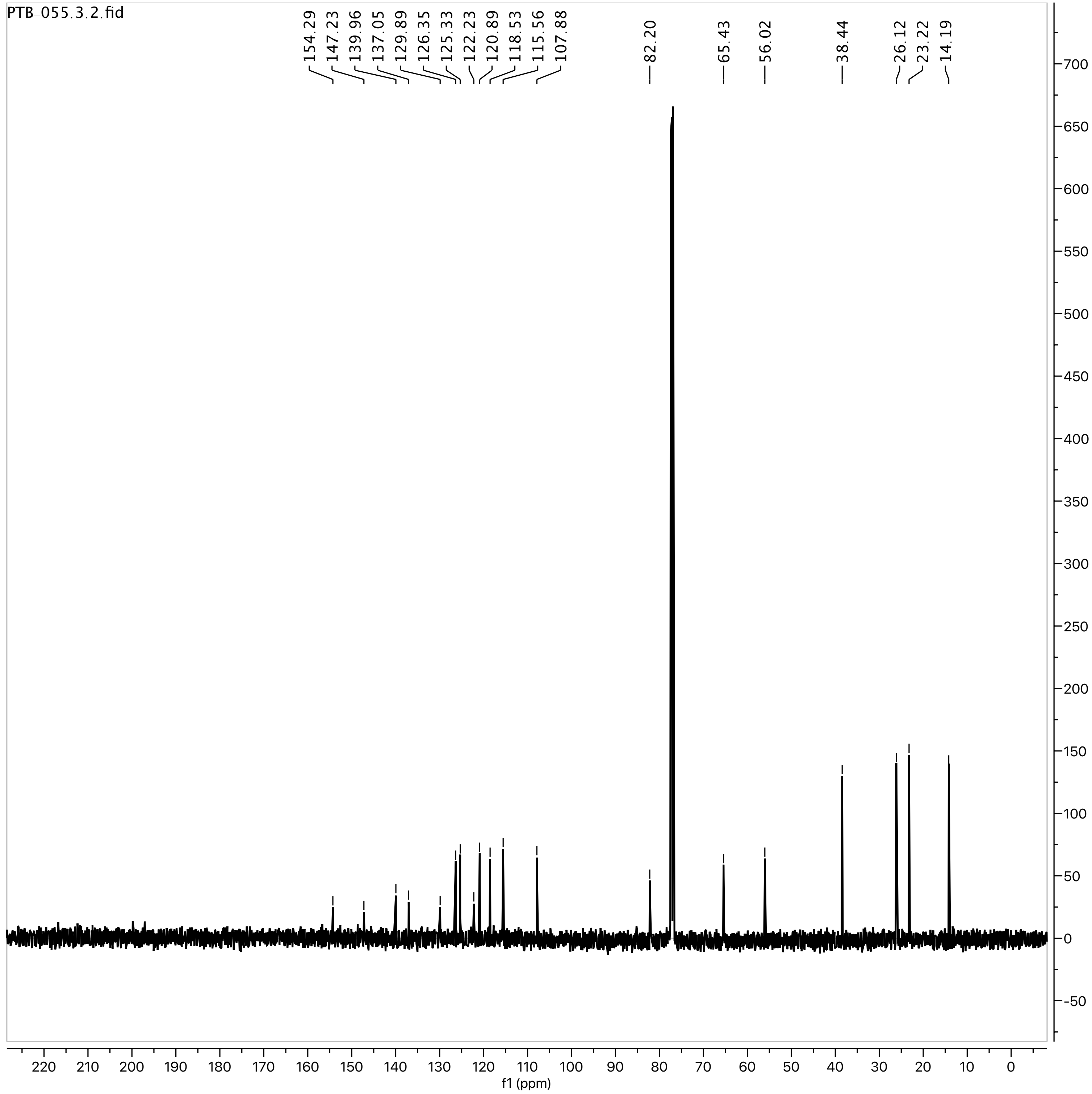

	Parameter	Value
1	Solvent	CDCl3
2	Experiment	1D
3	Spectrometer Frequency	125.78
4	Nucleus	13C

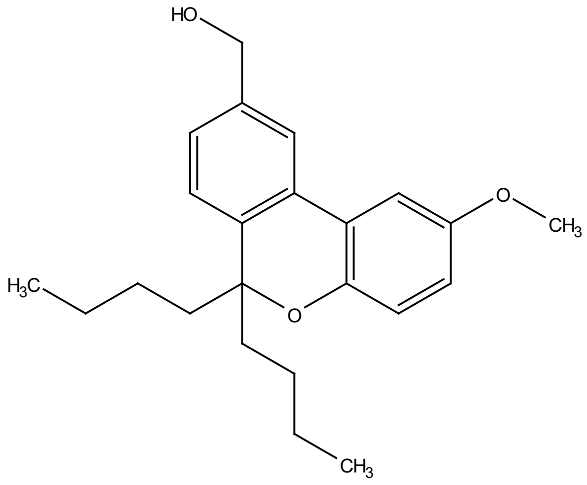

^{13}C NMR (126 MHz, $CDCl_3$) δ 154.29, 147.23, 139.96, 137.05, 129.89, 126.35, 125.33, 122.23, 120.89, 118.53, 115.56, 107.88, 82.20, 65.43, 56.02, 38.44, 26.12, 23.22, 14.19.

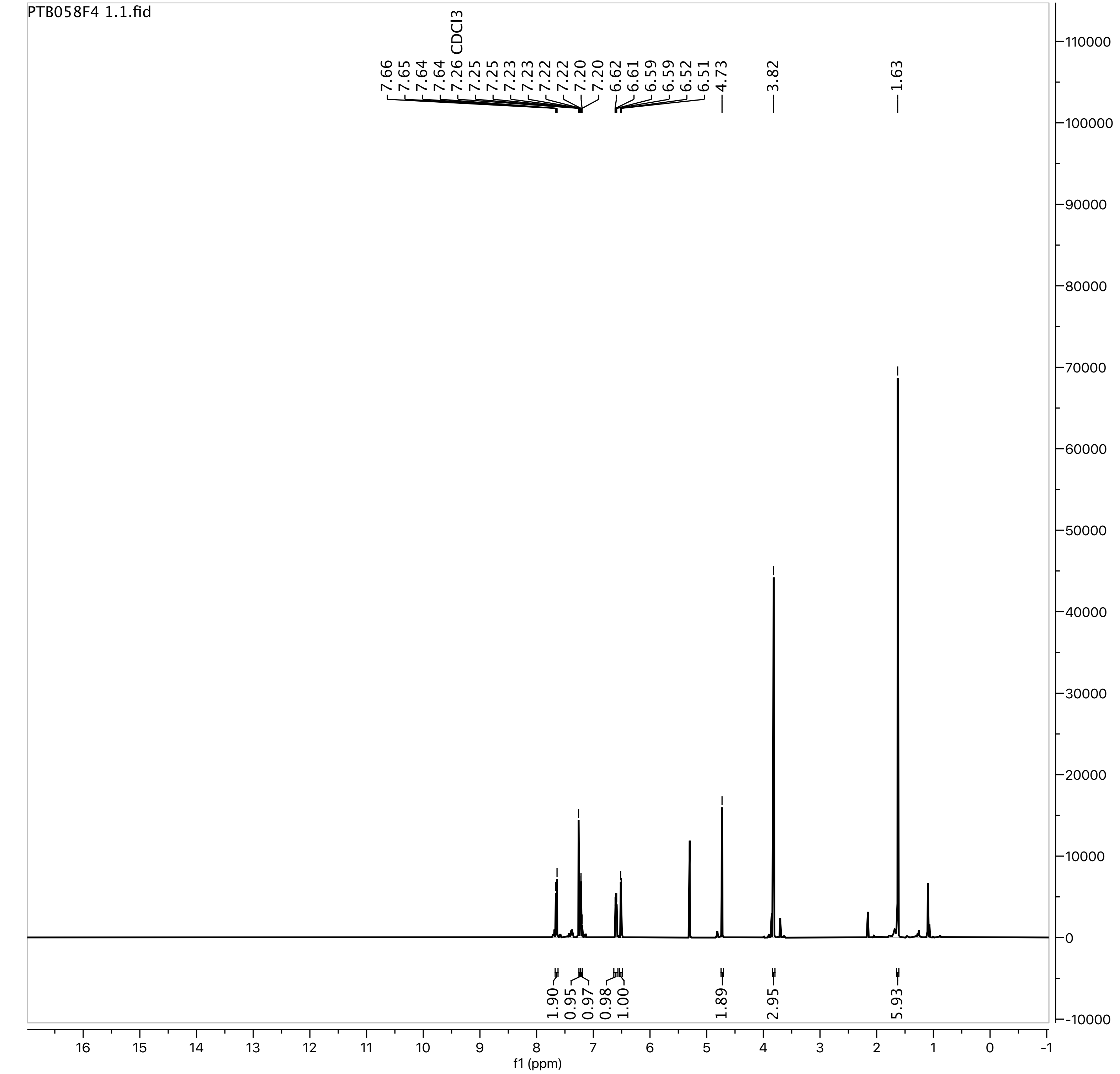

	Parameter	Value
1	Solvent	CDCl3
2	Experiment	1D
3	Spectrometer Frequency	400.13
4	Nucleus	1H

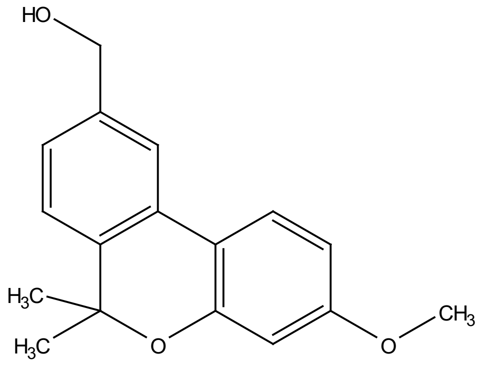

^{1}H NMR (400 MHz, $CDCl_3$) δ 7.67 – 7.62 (m, 2H), 7.24 (dd, J = 7.9, 1.6 Hz, 1H), 7.21 (dd, J = 7.9, 0.7 Hz, 1H), 6.60 (dd, J = 8.6, 2.6 Hz, 1H), 6.51 (d, J = 2.6 Hz, 1H), 4.73 (s, 2H), 3.82 (s, 3H), 1.63 (s, 6H).

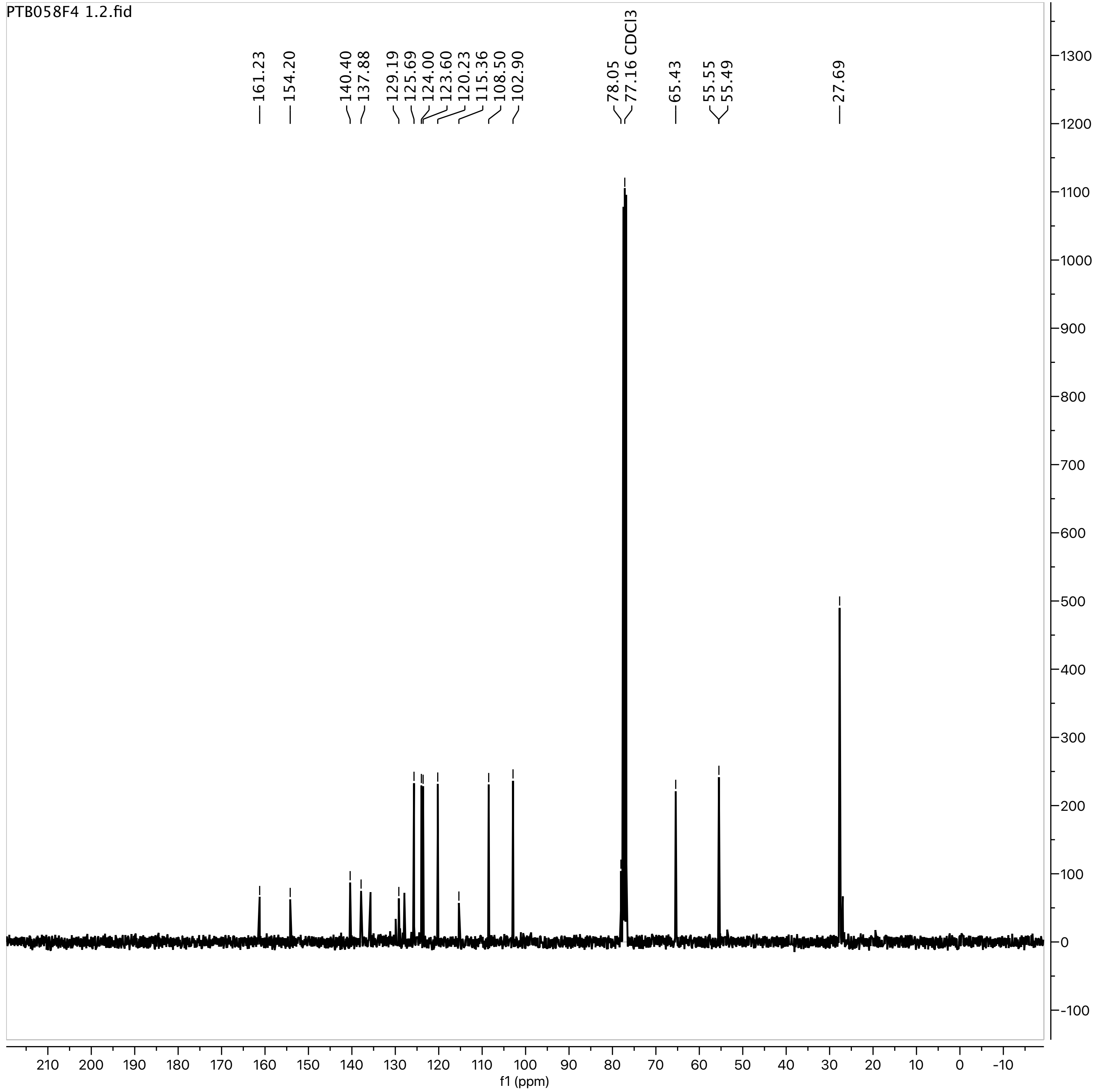

	Parameter	Value
1	Solvent	CDCl3
2	Experiment	1D
3	Spectrometer Frequency	100.62
4	Nucleus	13C

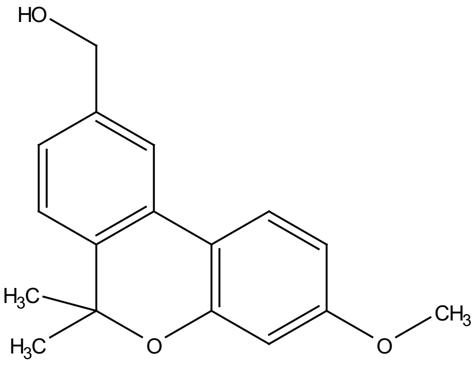

^{13}C NMR (101 MHz, $CDCl_3$) δ 161.23, 154.20, 140.40, 137.88, 129.19, 125.69, 124.00, 123.60, 120.23, 115.36, 108.50, 102.90, 78.05, 65.43, 55.49, 27.69.

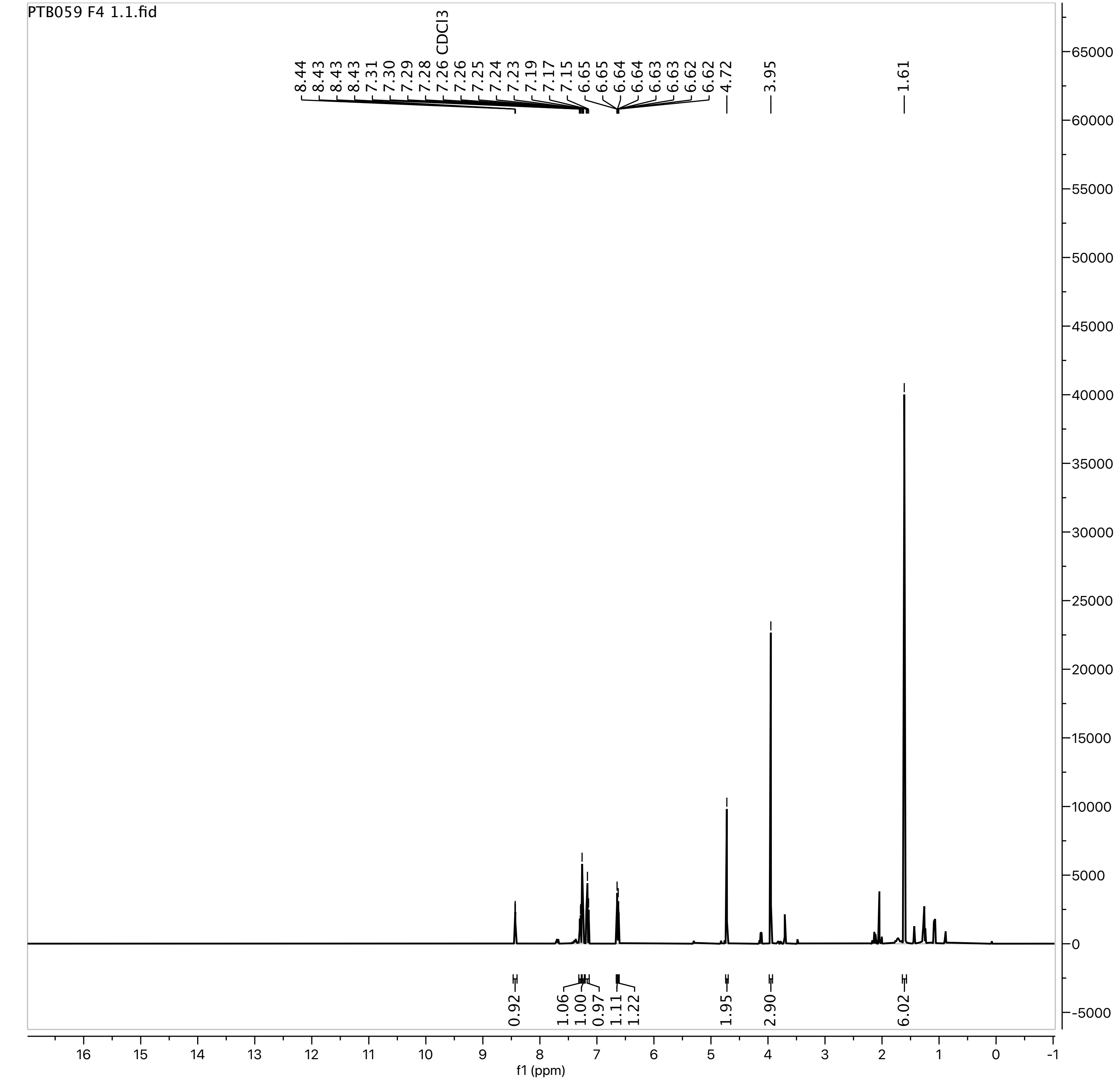

	Parameter	Value
1	Solvent	CDCl3
2	Experiment	1D
3	Spectrometer Frequency	400.13
4	Nucleus	1H

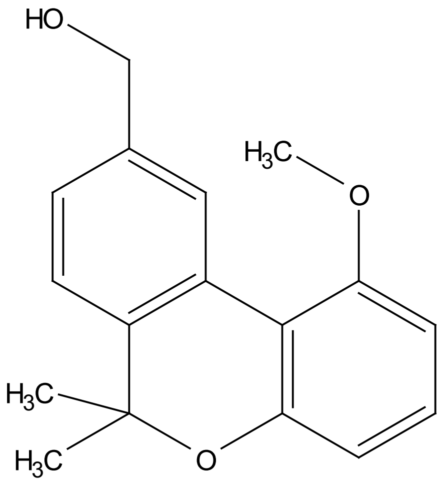

^{1}H NMR (400 MHz, $CDCl_3$) δ 8.43 (dd, J = 1.8, 0.7 Hz, 1H), 7.30 (dd, J = 7.9, 1.8 Hz, 1H), 7.25 (dd, J = 7.9, 0.4 Hz, 1H), 7.17 (t, J = 8.2 Hz, 1H), 6.64 (dd, J = 8.1, 1.1 Hz, 1H), 6.63 (dd, J = 8.3, 1.2 Hz, 1H), 4.72 (s, 2H), 3.95 (s, 3H), 1.61 (s, 6H).

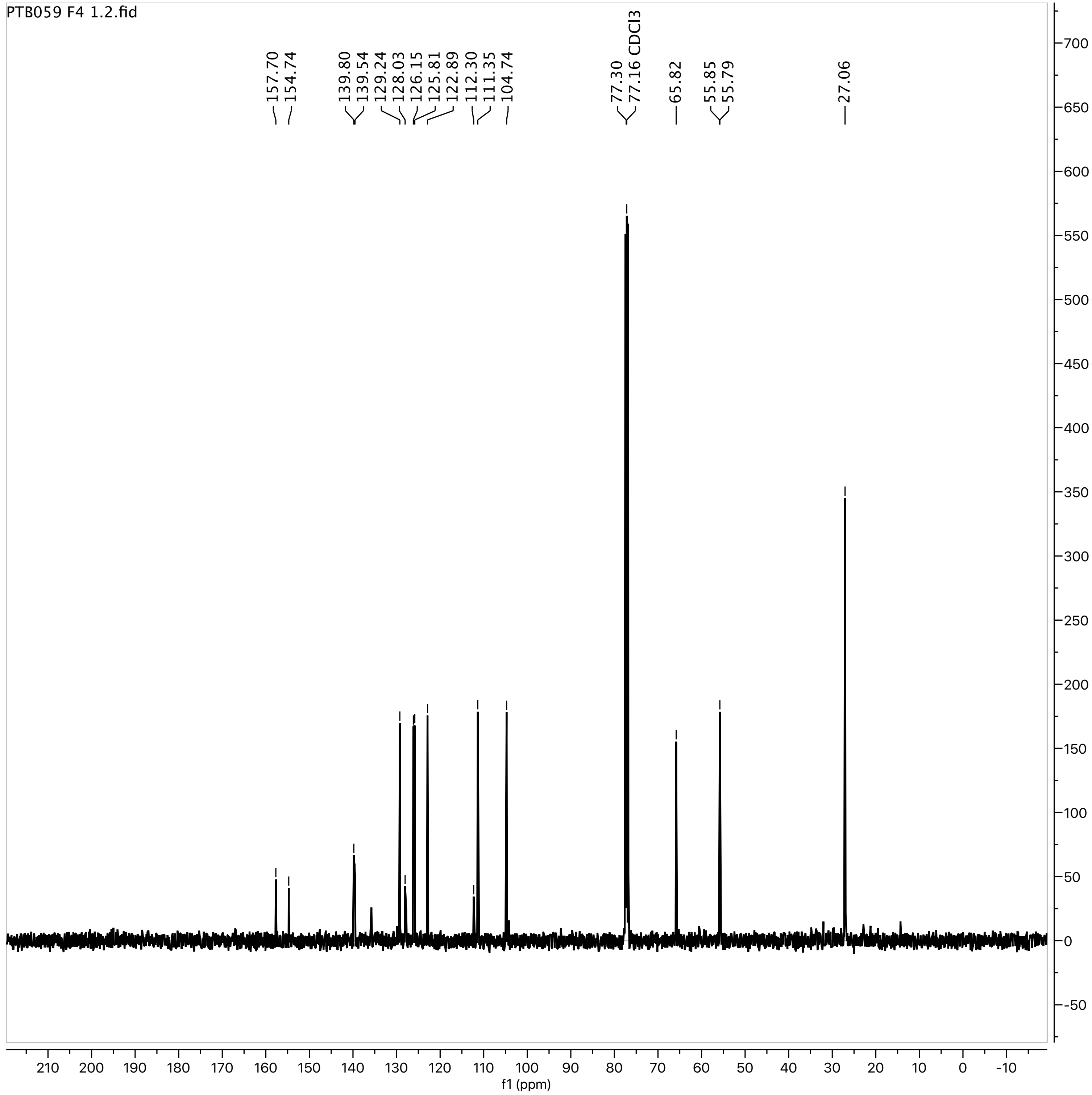

	Parameter	Value
1	Solvent	CDCl3
2	Experiment	1D
3	Spectrometer Frequency	100.62
4	Nucleus	13C

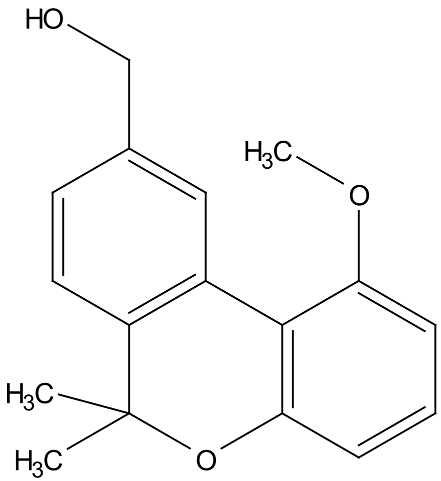

^{13}C NMR (101 MHz, $CDCl_3$) δ 157.70, 154.74, 139.80, 139.54, 129.24, 128.03, 126.15, 125.81, 122.89, 112.30, 111.35, 104.74, 77.30, 65.82, 55.79, 27.06.

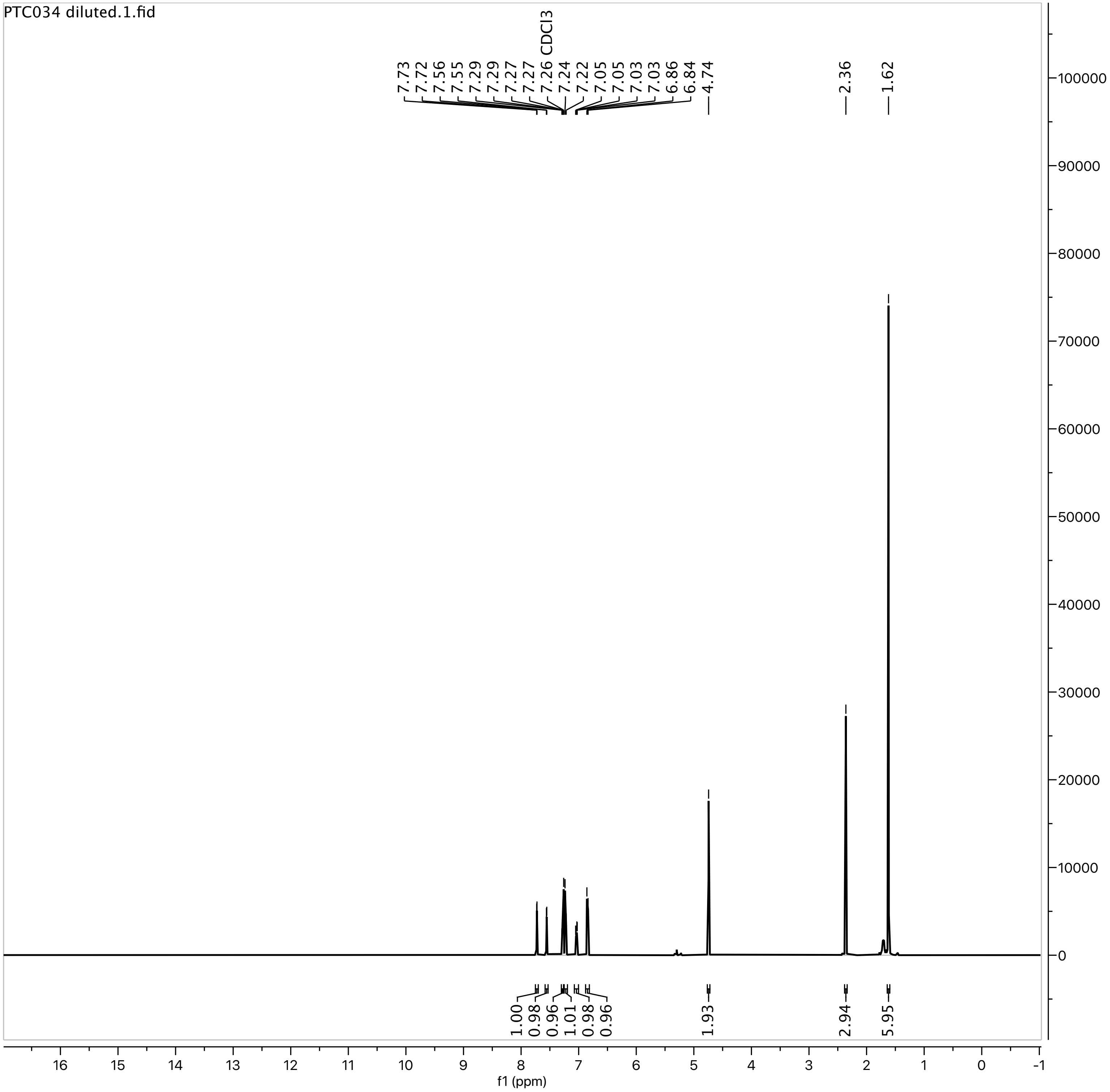

	Parameter	Value
1	Solvent	CDCl3
2	Experiment	1D
3	Spectrometer Frequency	400.13
4	Nucleus	1H

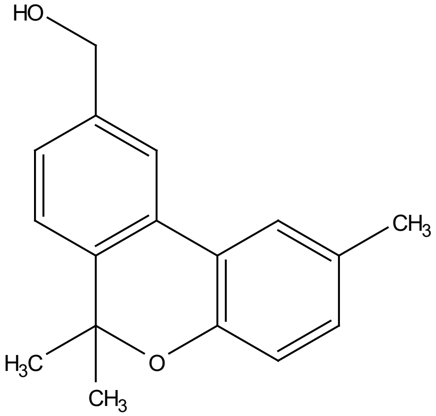

^{1}H NMR (400 MHz, $CDCl_3$) δ 7.73 (d, J = 2.0 Hz, 1H), 7.56 (d, J = 2.4 Hz, 1H), 7.28 (dd, J = 7.9, 1.9 Hz, 1H), 7.23 (d, J = 7.9 Hz, 1H), 7.04 (dd, J = 8.2, 2.4 Hz, 1H), 6.85 (d, J = 8.1 Hz, 1H), 4.74 (s, 2H), 2.36 (s, 3H), 1.62 (s, 6H).

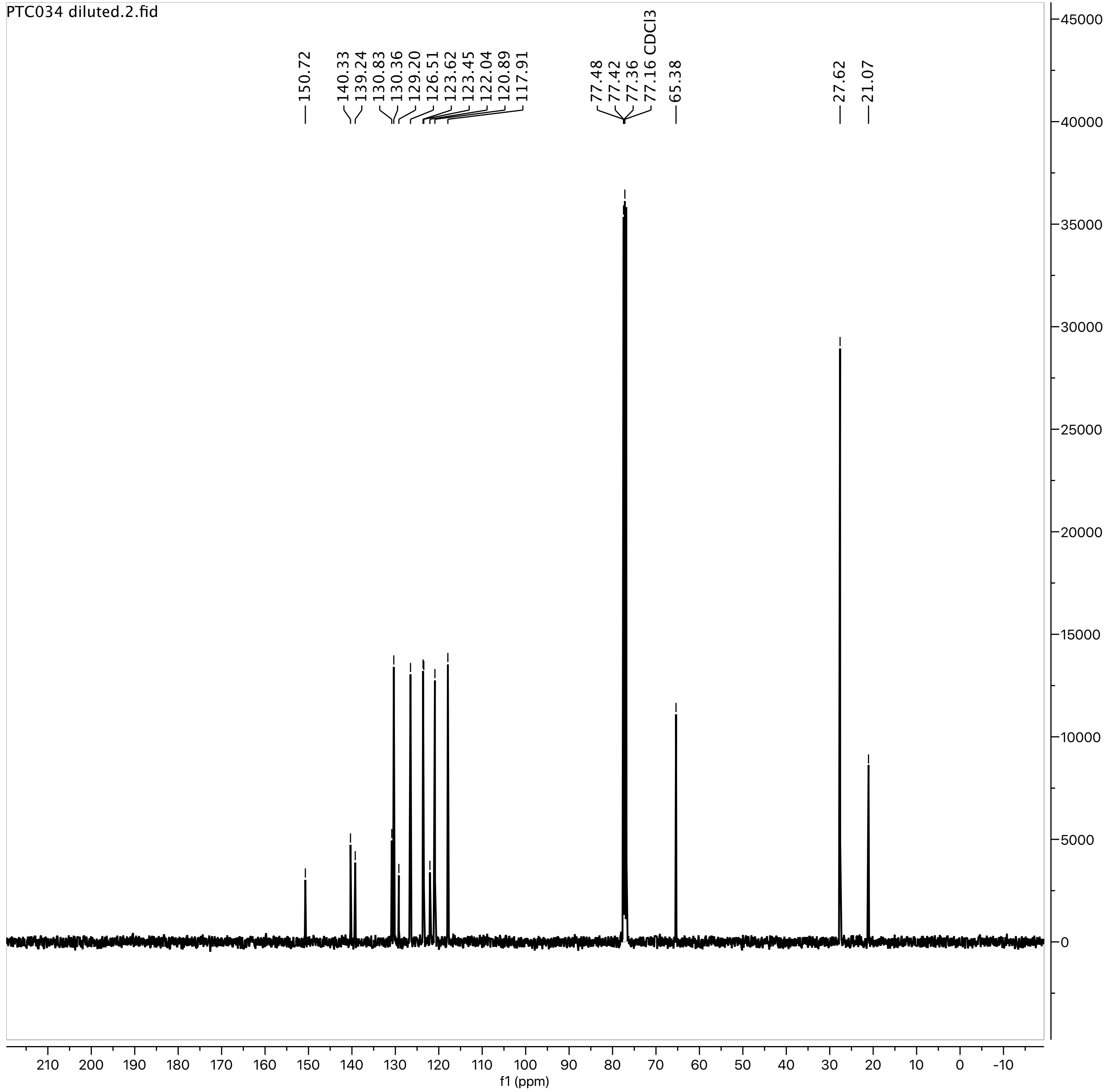

	Parameter	Value
1	Solvent	CDCl3
2	Experiment	1D
3	Spectrometer Frequency	100.62
4	Nucleus	13C

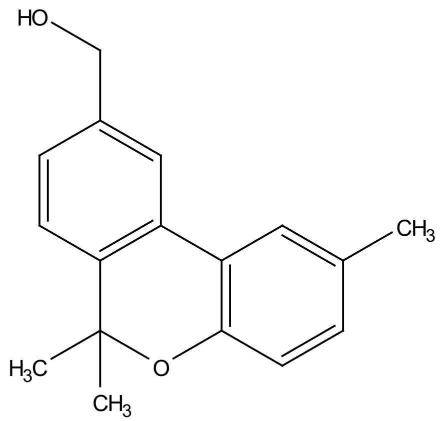

^{13}C NMR (101 MHz, $CDCl_3$) δ 150.72, 140.33, 139.24, 130.83, 130.36, 129.20, 126.51, 123.62, 123.45, 122.04, 120.89, 117.91, 77.45 (d, J = 5.5 Hz), 65.38, 27.62, 21.07.

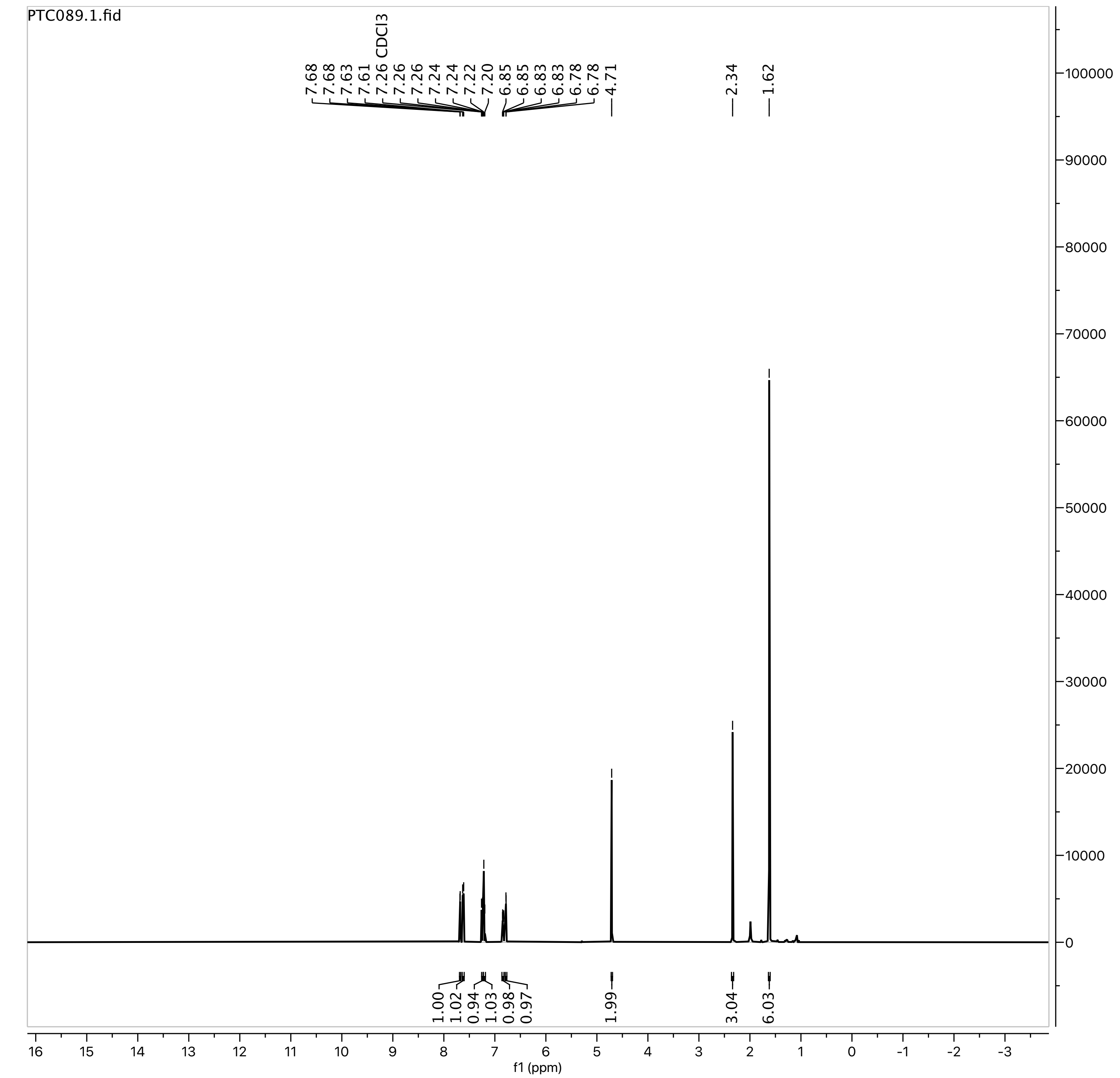

	Parameter	Value
1	Solvent	CDCl3
2	Experiment	1D
3	Spectrometer Frequency	400.13
4	Nucleus	1H

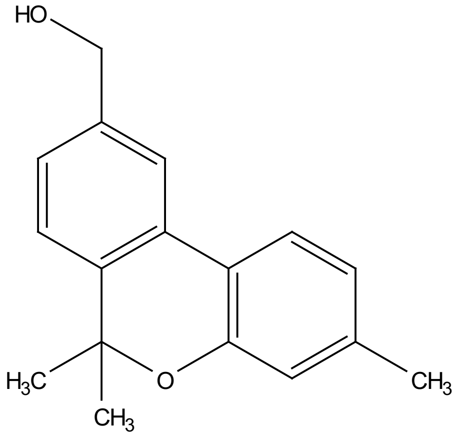

^{1}H NMR (400 MHz, $CDCl_3$) δ 7.68 (d, J = 1.8 Hz, 1H), 7.62 (d, J = 7.9 Hz, 1H), 7.26 – 7.23 (m, 1H), 7.21 (d, J = 7.8 Hz, 1H), 6.84 (dd, J = 7.8, 1.0 Hz, 1H), 6.78 (d, J = 1.0 Hz, 1H), 4.71 (s, 2H), 2.34 (s, 3H), 1.62 (s, 6H).

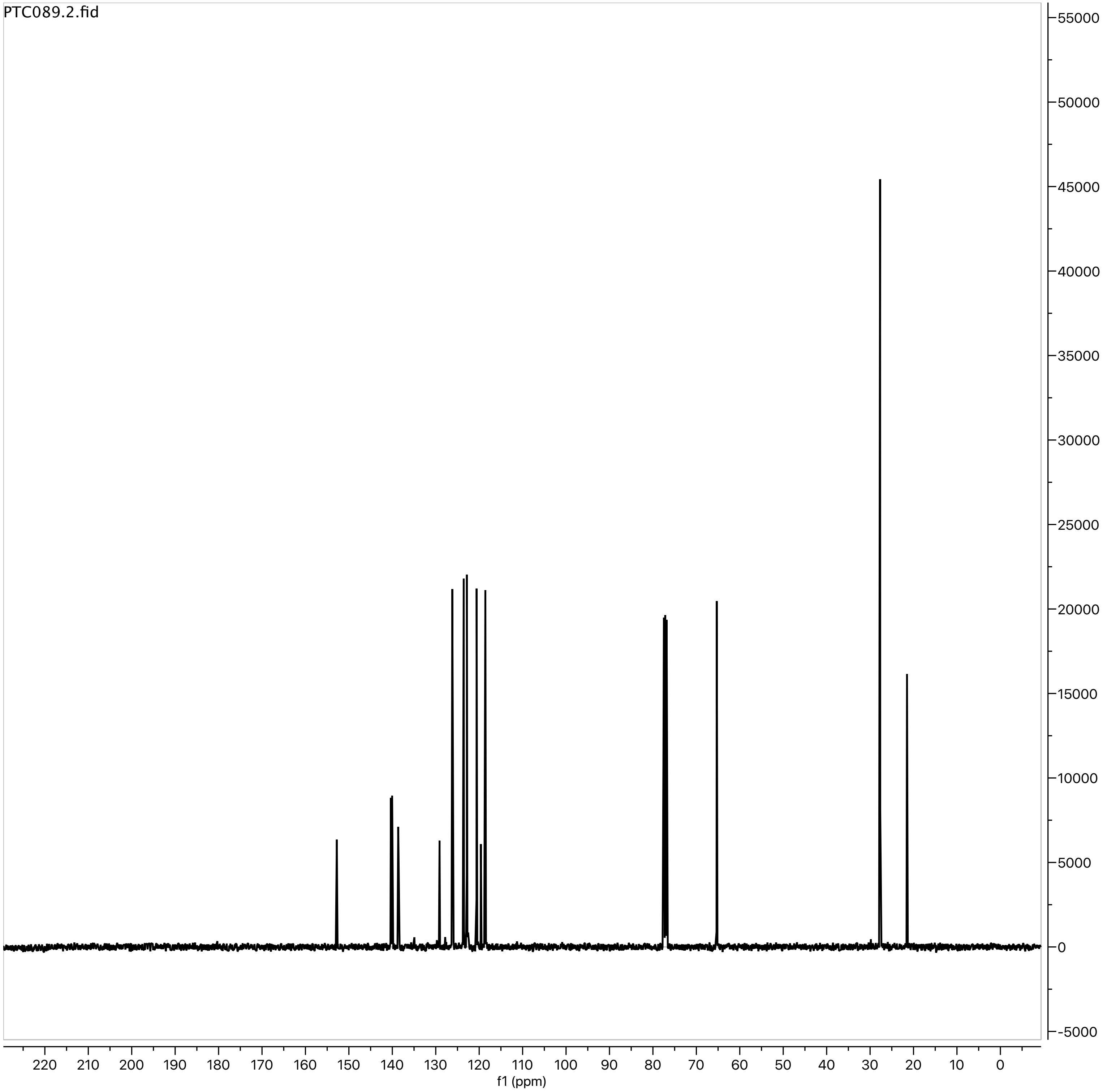

	Parameter	Value
1	Solvent	CDCl3
2	Experiment	1D
3	Spectrometer Frequency	100.62
4	Nucleus	13C

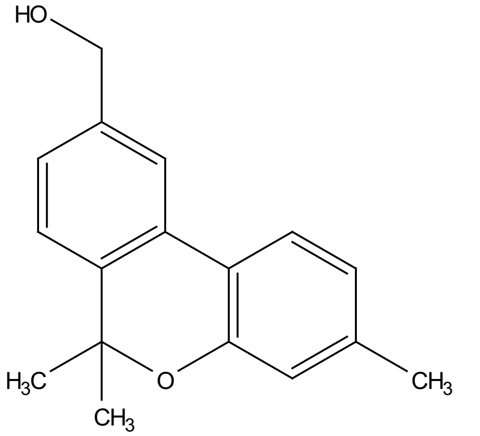

^{13}C NMR (101 MHz, $CDCl_3$) δ 152.78, 140.33, 140.04, 138.64, 129.13, 126.19, 123.56, 122.83, 122.60, 120.59, 119.61, 118.58, 77.53 (d, J = 11.4 Hz), 65.30, 27.69, 21.50.

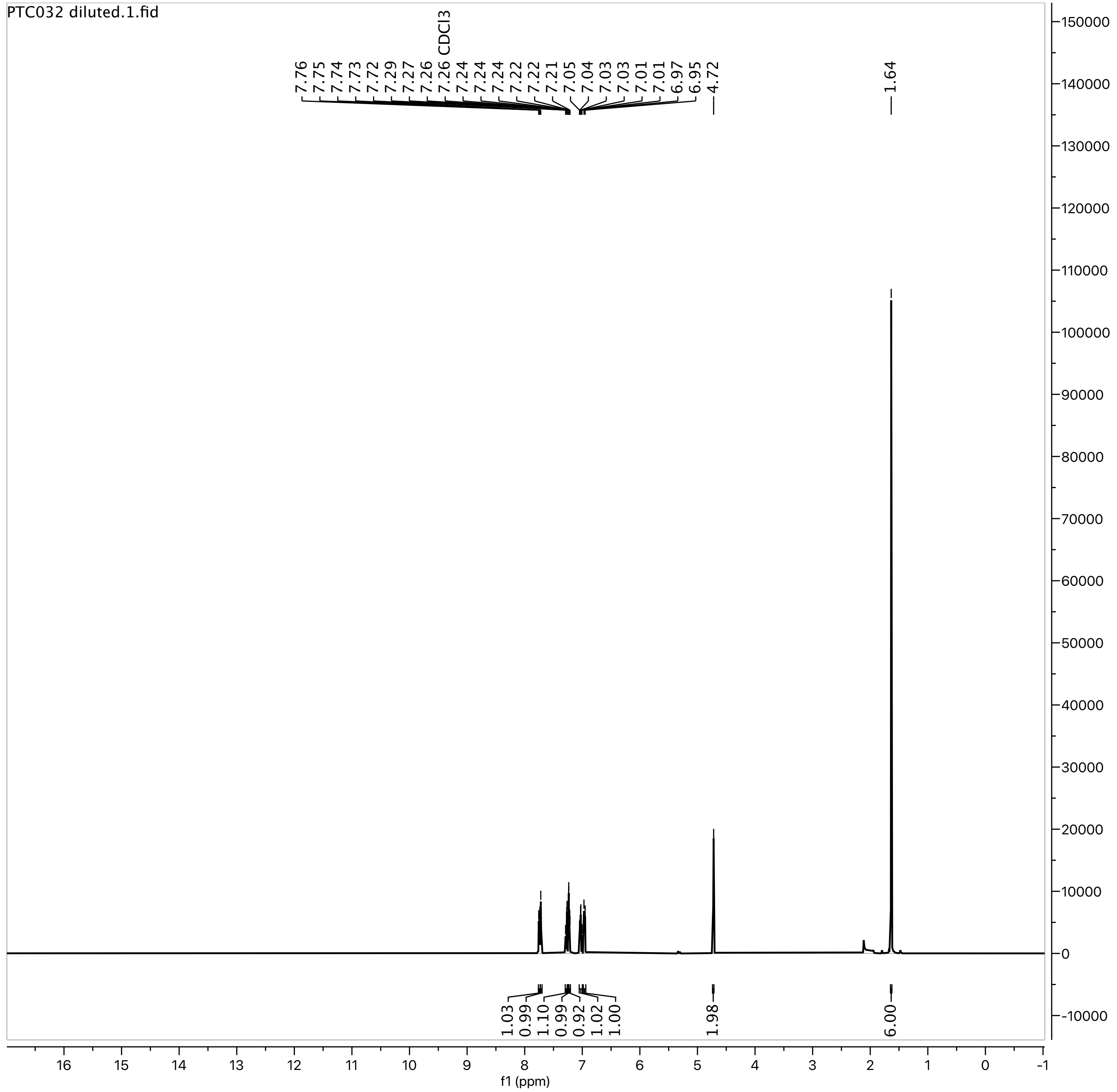

	Parameter	Value
1	Solvent	CDCl3
2	Experiment	1D
3	Spectrometer Frequency	400.13
4	Nucleus	1H

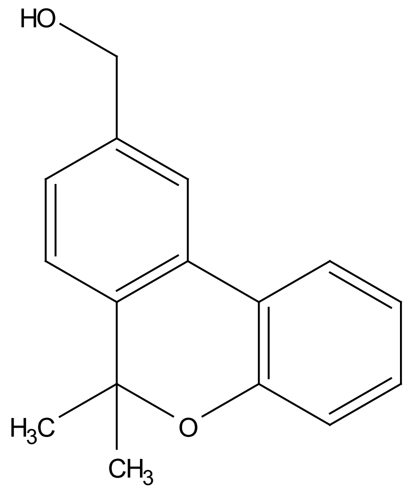

^1H NMR (400 MHz, $CDCl_3$) δ 7.74 (dd, J = 7.8, 1.7 Hz, 1H), 7.72 (s, 1H), 7.30 – 7.26 (m, 1H), 7.25 – 7.23 (m, 1H), 7.23 – 7.21 (m, 1H), 7.03 (td, J = 7.6, 1.5 Hz, 1H), 6.96 (d, J = 8.0 Hz, 1H), 4.72 (s, 2H), 1.64 (s, 6H).

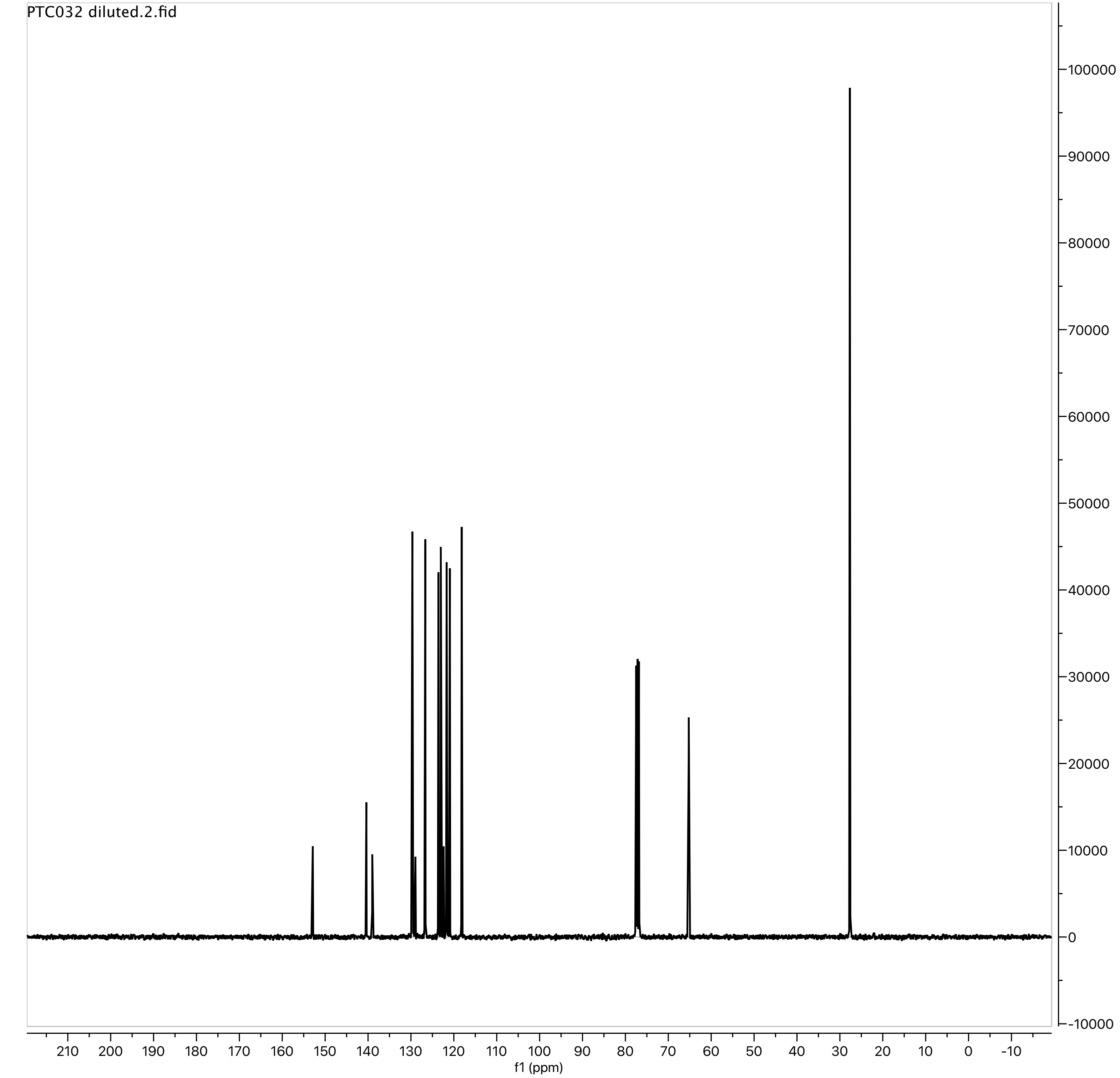

	Parameter	Value
1	Solvent	CDCl3
2	Experiment	1D
3	Spectrometer Frequency	100.62
4	Nucleus	13C

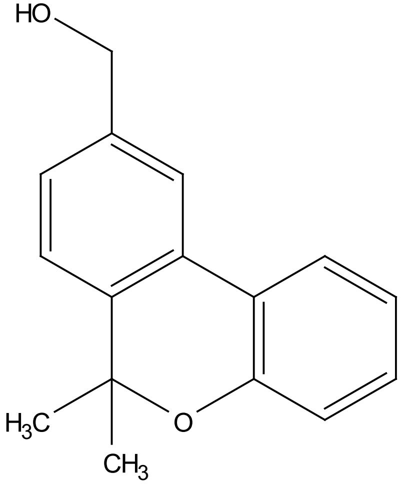

^{13}C NMR (101 MHz, $CDCl_3$) δ 152.89, 140.39, 139.01, 129.66, 128.95, 126.64, 123.60, 123.04, 122.38, 121.68, 120.92, 118.17, 77.62, 65.24 (d, J = 2.9 Hz), 27.67..

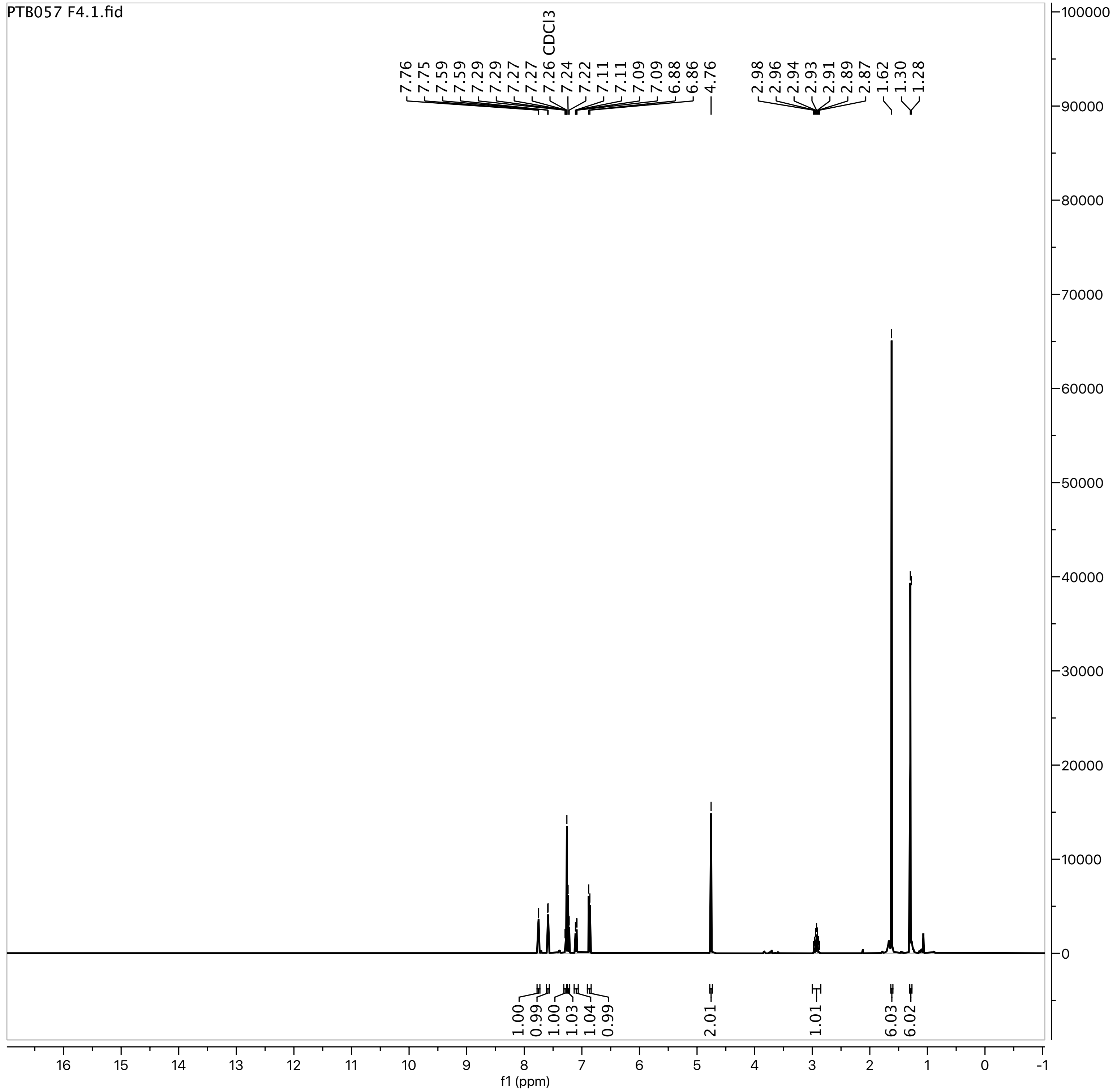

	Parameter	Value
1	Solvent	CDCl3
2	Experiment	1D
3	Spectrometer Frequency	400.13
4	Nucleus	1H

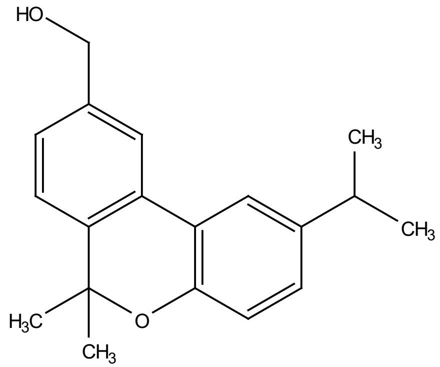

^{1}H NMR (400 MHz, $CDCl_3$) δ 7.75 (d, J = 1.8 Hz, 1H), 7.59 (d, J = 2.1 Hz, 1H), 7.28 (dd, J = 7.9, 1.8 Hz, 1H), 7.23 (d, J = 7.6 Hz, 1H), 7.10 (dd, J = 8.3, 2.2 Hz, 1H), 6.87 (d, J = 8.3 Hz, 1H), 4.76 (s, 2H), 2.93 (hept, J = 6.9 Hz, 1H), 1.62 (s, 6H), 1.29 (d, J = 6.9 Hz, 6H).

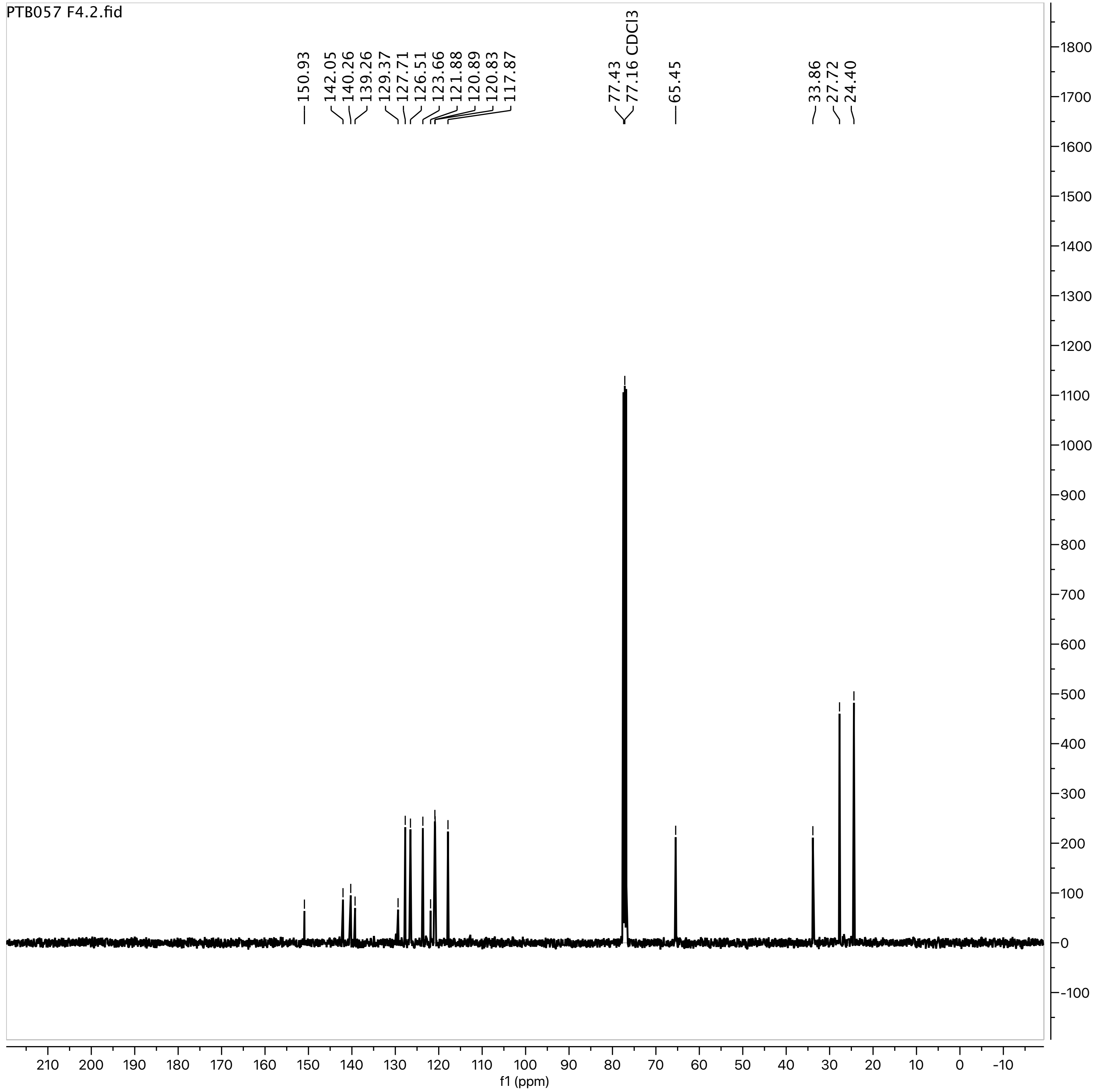

	Parameter	Value
1	Solvent	CDCl3
2	Experiment	1D
3	Spectrometer Frequency	100.62
4	Nucleus	13C

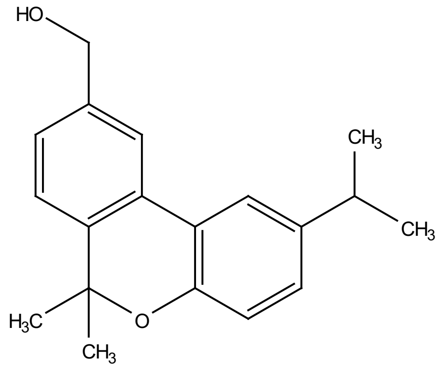

^{13}C NMR (101 MHz, $CDCl_3$) δ 150.93, 142.05, 140.26, 139.26, 129.37, 127.71, 126.51, 123.66, 121.88, 120.89, 120.83, 117.87, 77.43, 65.45, 33.86, 27.72, 24.40.

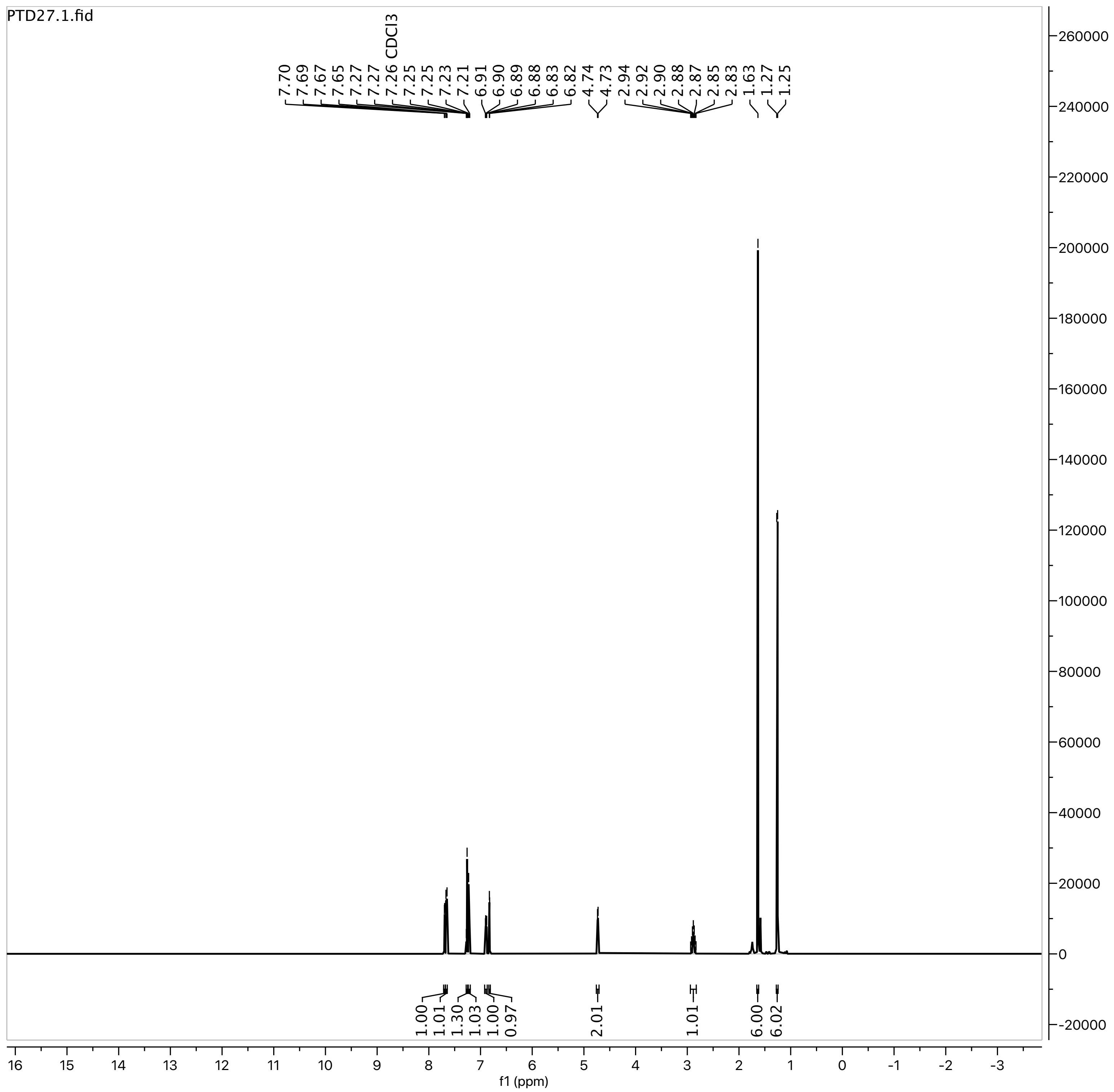

	Parameter	Value
1	Solvent	CDCl3
2	Experiment	1D
3	Spectrometer Frequency	400.13
4	Nucleus	1H

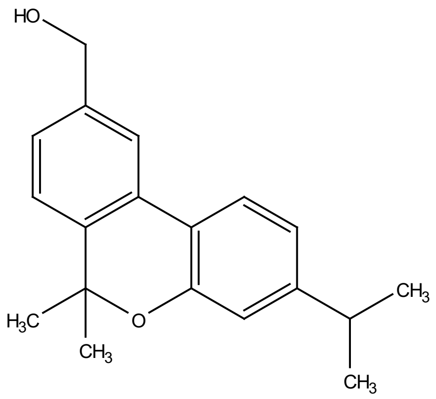

^{1}H NMR (400 MHz, $CDCl_3$) δ 7.70 (d, J = 1.7 Hz, 1H), 7.66 (d, J = 8.1 Hz, 1H), 7.26 (dd, J = 7.9, 1.7 Hz, 1H), 7.22 (d, J = 7.9 Hz, 1H), 6.89 (dd, J = 7.9, 1.8 Hz, 1H), 6.83 (d, J = 2.0 Hz, 1H), 4.73 (d, J = 4.2 Hz, 2H), 2.88 (hept, J = 7.0 Hz, 1H), 1.63 (s, 6H), 1.26 (d, J = 6.8 Hz, 6H).

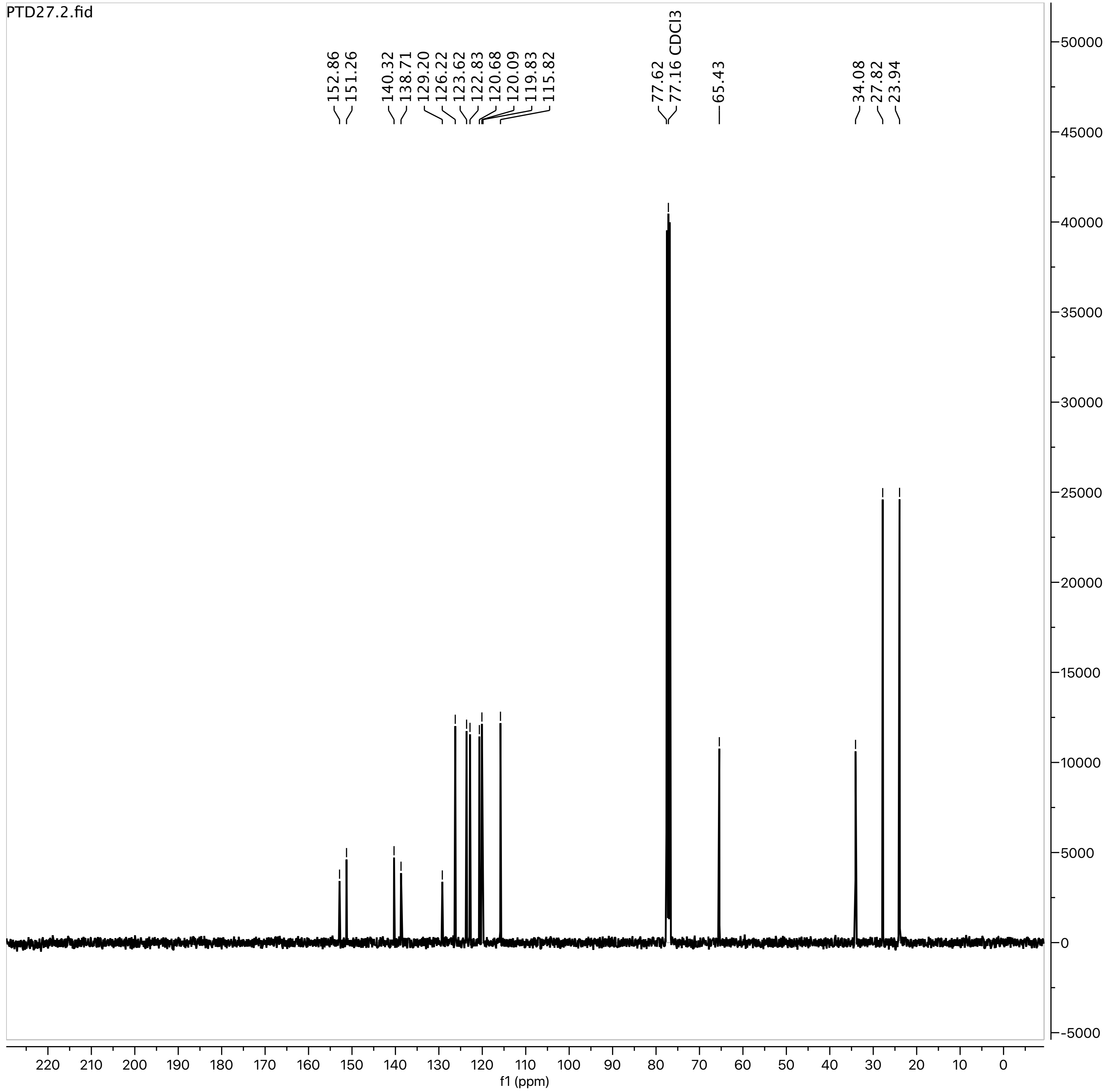

	Parameter	Value
1	Solvent	CDCl3
2	Experiment	1D
3	Spectrometer Frequency	100.62
4	Nucleus	13C

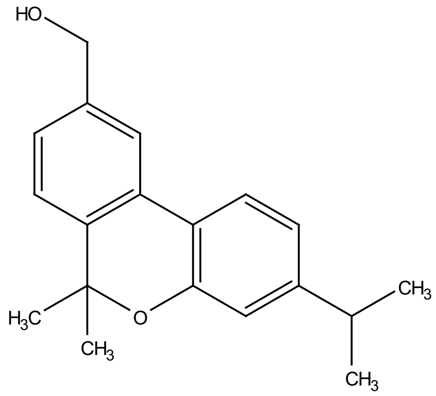

^{13}C NMR (101 MHz, $CDCl_3$) δ 152.86, 151.26, 140.32, 138.71, 129.20, 126.22, 123.62, 122.83, 120.68, 120.09, 119.83, 115.82, 77.62, 65.43, 34.08, 27.82, 23.94.

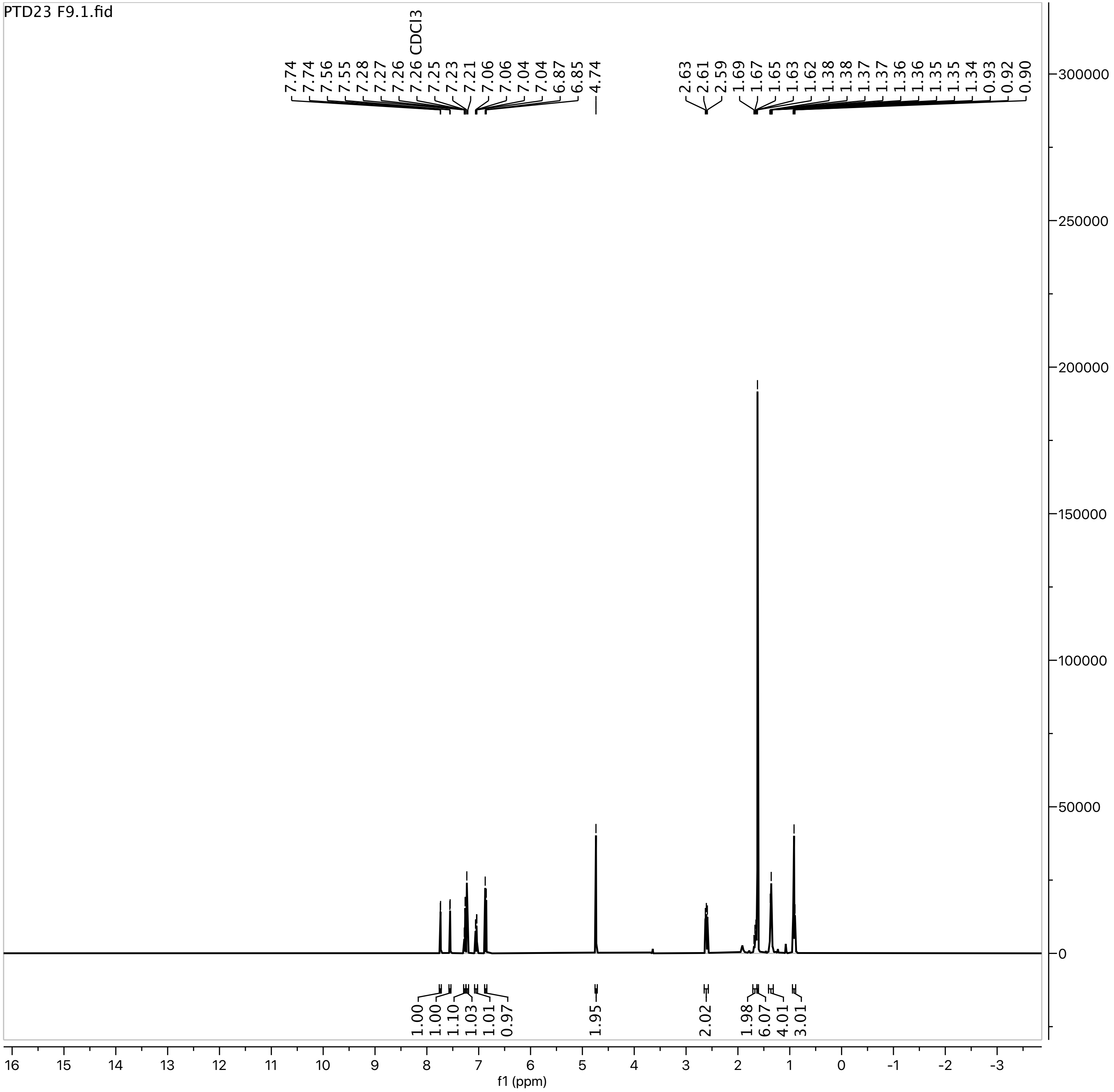

	Parameter	Value
1	Solvent	CDCl3
2	Experiment	1D
3	Spectrometer Frequency	400.13
4	Nucleus	1H

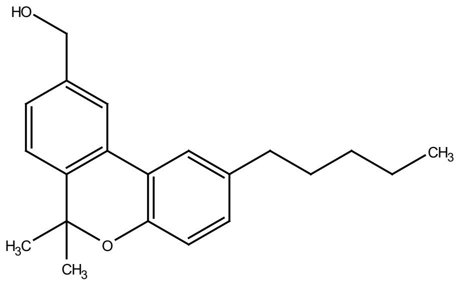

^{1}H NMR (400 MHz, $CDCl_3$) δ 7.74 (d, J = 1.8 Hz, 1H), 7.55 (d, J = 2.2 Hz, 1H), 7.27 (dd, J = 7.6, 2.1 Hz, 1H), 7.22 (d, J = 7.8 Hz, 1H), 7.05 (dd, J = 8.2, 2.1 Hz, 1H), 6.86 (d, J = 8.2 Hz, 1H), 4.74 (s, 2H), 2.65 – 2.57 (m, 2H), 1.71 – 1.63 (m, 2H), 1.62 (s, 6H), 1.41 – 1.32 (m, 4H), 0.95 – 0.89 (m, 3H).

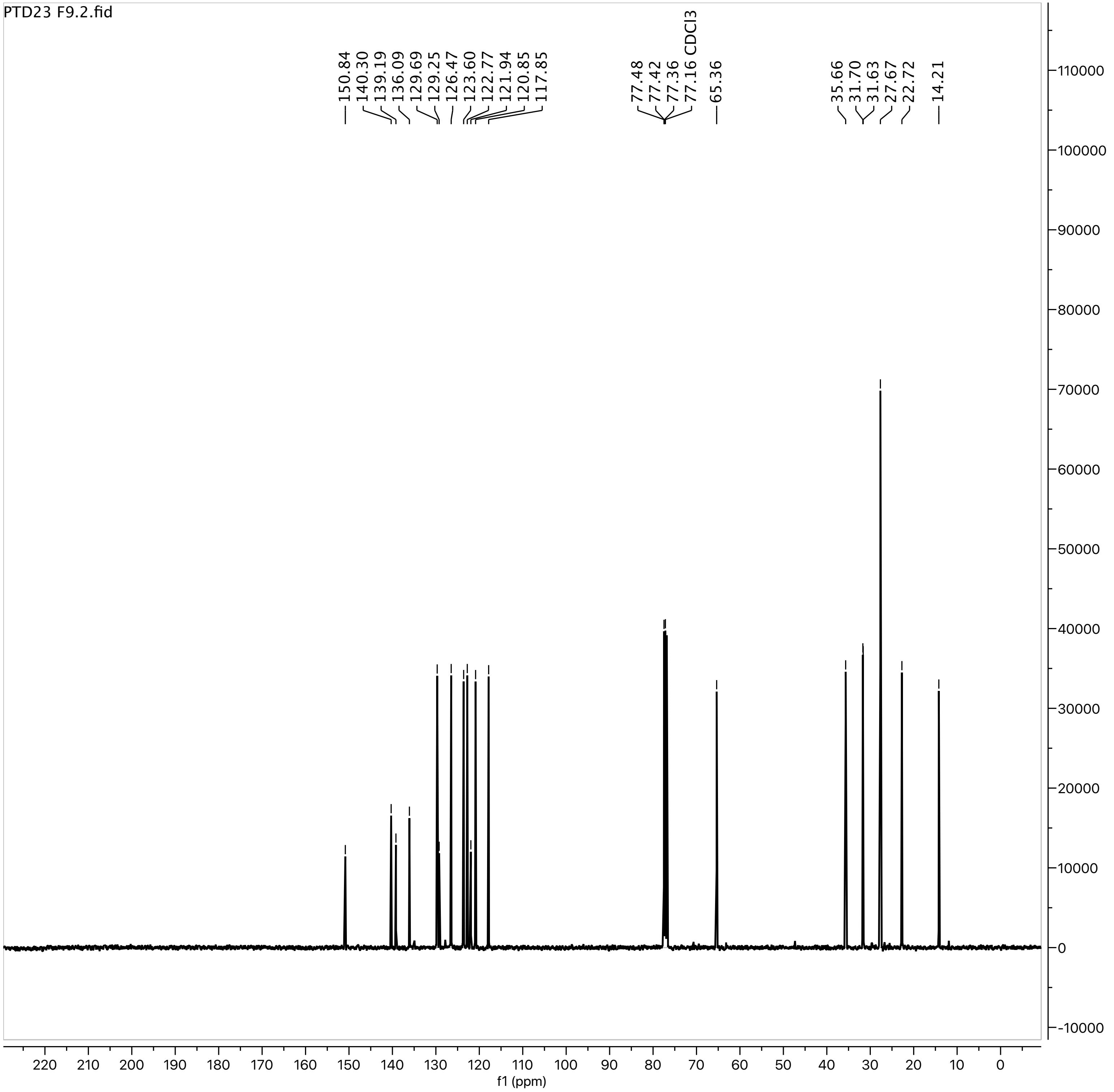

	Parameter	Value
1	Solvent	CDCl3
2	Experiment	1D
3	Spectrometer Frequency	100.62
4	Nucleus	13C

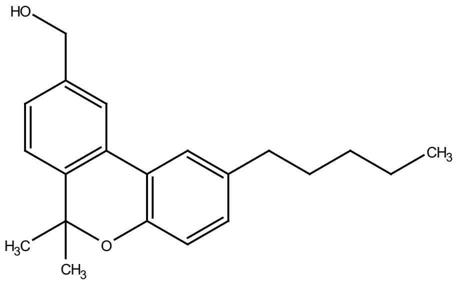

^{13}C NMR (101 MHz, $CDCl_3$) δ 150.84, 140.30, 139.19, 136.09, 129.69, 129.25, 126.47, 123.60, 122.77, 121.94, 120.85, 117.85, 77.45 (d, J = 5.9 Hz), 65.36, 35.66, 31.70, 31.63, 27.67, 22.72, 14.21.
